# Understanding volunteerism among dental students and professionals to reach Saudi Arabia Vision 2030 goals

**DOI:** 10.1371/journal.pone.0296745

**Published:** 2024-01-10

**Authors:** Khalid Aboalshamat, Turki Alayyafi, Ghassan Elaiwa, Mishal Assayegh, Abdulmalk Alqaidi

**Affiliations:** 1 Dental Public Health Division, Preventative Dentistry Department, Faculty of Dental Medicine, Umm Al-Qura University, Makkah, Saudi Arabia; 2 College of Dentistry, Umm Al-Qura University, Makkah, Saudi Arabia; Myanmar Health Network Organization, MYANMAR

## Abstract

**Background:**

Volunteering can be defined as activities a person does for free to help another person or group. Saudi Vision 2030 has a target of one million volunteers from the country by 2030. The aim of this study was to find out the frequencies of the motives, barriers, and experiences of volunteering dental students and dentists in Saudi Arabia.

**Materials and methods:**

In this cross-sectional study, 655 dental students and dentists from 37 cities around Saudi Arabia answered a questionnaire of 59 questions derived from previous studies. The questionnaire was distributed through social media. SPSS software was used to analyze the data, with p-value of 0.05 as significant. Chi-square was used for analytical statistics.

**Results:**

The chance to learn in a health-related field (84.58%) was the most motivating factor to volunteer, and the least motivating factor was financial compensation (46.72%). Females had multiple significantly higher motives percentages than males (p<0.05). The most prominent barrier was time constraints (74.50%), while the least important barrier was parents/family disapproval (28.85%). Lack of transportation was a more significant (p<0.001) barrier for females than males. Of the respondents, 74.50% had previously participated in volunteer work. Among those, 98.36% volunteered in Saudi Arabia and 6.97% volunteered outside of Saudi Arabia. Also, 46.31% volunteered during the COVID-19 pandemic.

**Conclusions:**

A high percentage of dental students and dentists in Saudi Arabia engage in volunteer activities. Nevertheless, various impediments must be addressed to achieved the targeted key performance indicator of Saudi Arabia Vision 2030.

## Introduction

Volunteering can be defined as the activities that someone engages in for free in order to help another person or group [1]. Volunteering becomes a crucial aspect of health care during health disasters and pandemics, such as COVID-19 [2, 3]. This is because the burden of a disaster exceeds the capacity of governments and the normal workforce to meet the high demands for health services [4]. In fact, many medical schools include a volunteering section in their curriculum [3]. Studies have shown that 87.9% to 89% of dental students believe that volunteering is indicative of a competent dentist [5, 6]. Volunteering has a bidirectional benefit to both communities and volunteers. Studies among health-care students have revealed relationships between volunteering and increased self-efficacy, self-esteem, resilience, job consciousness, health profession loyalty, and belonging, along with decreased depression [7–9].

In contrast, other studies have linked volunteering to post-traumatic stress disorder due to the circumstances of the disaster and highlighted the importance of proper training for volunteering [10]. Other studies have also claimed that students do not have enough knowledge about the skills needed during crises [4, 11] and health-care students feel uncertain during pandemics [12]. However, health-care students can volunteer for activities that are directly related to medical aspects, such as patient admission, triage, and conducting diagnostic laboratories [13], or activities that are indirectly related, such as receiving calls, community education, community tracking, transporting of goods, and monitoring disaster victims [13, 14]. Such activities have not been investigated among dental students and dentists, who may help with indirect medical volunteering, particularly during a disaster. Furthermore, there is a knowledge gap with regard to the direct volunteering opportunities dental students and dentists have in their field of study in Saudi Arabia.

Several studies investigated the percentage of health-care students who had volunteered or were willing to volunteer. Many studies have focused on medical students [15–17] or nursing students [18, 19], but few studies have focused on dental students specifically. In fact, many volunteering studies have included dental students under the larger umbrella of other specialties, such as medicine and others, instead of investigating dental professionals in depth. These studies have shown the percentages of dental students in their studies who volunteered in Japan (43%) [13], Pakistan (64%) [20], and Saudi Arabia (30.31%) [21]. In addition, studies have shown high levels of interest and willingness to participate in volunteer activities among medical and dental students during disasters in Japan (89.9%) [13] and Nigeria (82.9%) [22]. The data in these studies did not clearly define the percentages related to dental students separate from other specialties, which highlights a gap in knowledge.

Also, there is a very limited number of studies investigating volunteer work among practicing dentists. One national-scale study in the United States found that 41.9% of dentists of had volunteered outside of the United States [23]. Such a study investigating volunteering among working dentists is lacking elsewhere, particularly in Saudi Arabia.

A recent systematic review noted the importance of researching the motivations for and barriers to volunteering among health-care professionals for better support and utilization of such a group [24]. The extant studies are varied in focus, with some investigating local volunteering in the same city or country [16, 20, 21]; volunteering abroad, in a different country [23, 25]; and volunteering in the case of a disaster, such as COVID-19 [13, 19, 22]. This might explain the differences in the main motivations found in these studies. A systematic review indicated that the main motivation for volunteering among health-care students was moral responsibility (including a sense of duty and social dedication), personal interest, chance to learn, and monetary compensation [24]. One interesting study in Pakistan found that friends were the main motivation for volunteering among medical and dental students [20]. This factor might be important when studying highly social communities, such as in Saudi Arabia. Studies investigated health-care students (including dental students) in Saudi Arabia have found the students motivated to volunteer for religious reasons, to gain experience, and out of a sense of patriotism [21]. However, these factors were tempered by lack of available transportation, the distance between the volunteer site and home, and the inability to use the health care for family members [16]. One study investigating volunteering on medical mission trips found that meeting people from different backgrounds and gaining clinical experience were the strongest motivations among medical students [25]. Conversely, studies investigating volunteering during a disaster found that disaster-related media coverage, professional obligations, governmental requests, medical workforce shortages, and priority access to vaccines were most important factors influencing motivation [13, 19, 22].

Research has reported a wide array of barriers to volunteering among health-care students, including lack of interest, lack of knowledge, transportation issues [21], time constraints [26], workload, treatment complications, feeling that volunteering is not a solution to the problem of access to dental care [27], fear of infection (COVID-19), and fear of transmitting an infection to family or friends [15, 24, 28]. It should be highlighted that most of the prior studies investigated motivation and barriers to volunteering among health-care students, and extrapolation of the results to health-care workers or dentists might not be applicable. Furthermore, it is worth noting that varying types of volunteer projects are required under different circumstances and at different points in time. This variability adds complexity to the task of comparing studies when reviewing the literature.

Saudi Arabia Vision 2030 set a goal to reach one million volunteers by 2030 [29]. Understanding the motivations, barriers, and practices will enable stakeholders to craft a road map to improve the infrastructure for volunteering by dental professionals.

## Aim

The aim of this study was to find out the frequencies of the motivations, barriers, and experiences of volunteering among dental students and dentists in Saudi Arabia.

## Materials and methods

This cross-sectional study was conducted with dental students and dentists in Saudi Arabia from 26 December 2022 to 30 January 2023. Sample size calculation was used to calculate the minimum sample necessary and was set to 385 participants by calculating the alpha of 0.05, expected proportion of motivational behaviors of 50% (chosen to provide a safety margin for generating the maximum sample size), and confidence level of 95%. The participants in this study were recruited via an online survey distributed via different social media platforms, including WhatsApp, Twitter, LinkedIn, TikTok, Snapchat, and Instagram, using a convenience sampling method. This method was used due to the impracticality of constructing a sample frame due to the unavailability of contact details or information for the specified population. The research team utilized social media platforms to reach out to dental students and dentists accounts and invite them to participate in the study. Additionally, invitations were disseminated within groups of dental students and dentists, which typically comprise a substantial number of members. This approach was particularly emphasized on the WhatsApp platform, given its extensive usage in Saudi Arabia. Inclusion criteria included dental students or working dentists in Saudi Arabia who approved the study’s informed consent. Exclusion criteria were students in the first year of dentistry, because the first year is a preparatory year for all health students in most Saudi universities. To maintain the confidentiality of participants’ information, each questionnaire was given an identification number so that the respondent’s identity remained protected. The study was approved by the institutional review board of Umm Al-Qura University with approval number HAPO-02-K-012-2022-11-1353. Informed consent was administered electronically, requiring each participant to click the "approve" button on the consent form before proceeding to the questionnaire pages. If a participant did not agree to the terms outlined in the consent form, they were considered to have declined to provide consent.

The questionnaire consisted of 59 questions divided into four sections. The first section was composed of demographic questions investigating participants’ gender, age, academic year or work status, region of residence, city, university or hospital, sector, and nationality. Questions about age, city and university/hospital were the only open-ended questions. Other questions were close-ended. Section two included 15 questions assessing potential motivations and 14 potential barriers. Section three asked six questions about volunteering opportunities. The last section was only given to participants who had previously participated in volunteer work and composed of nine questions about their experiences and seven questions about the compensation they received. The questions in sections two to four were close-ended with yes/no answers. The questionnaire was administered in English because dental students and dentists in Saudi Arabia undergo their education and licensing examinations in the English language. Questions were derived from multiple studies [13–16, 19, 21–24, 26–28] with modifications, and the questionnaire underwent a pilot with 12 participants to ensure face validation with language, syntax, logic, flow, and understanding.

In this research, data analysis was conducted using SPSS (IBM, Armonk, NY, USA) and Microsoft Excel software. Descriptive statistics, including counts, percentages, means, and standard deviations, were employed for data summarization. The Chi-square test, with a significance level set at p < 0.05, was utilized as the statistical test.

## Results

A total of 655 dental students and dentists answered the study questionnaire. The mean age of the participants was 23.74 with SD of 4.04 years. There were 436 (66.56%) dental students and 219 (33.43%) interns/dentists in this study. The participants were from 37 cities in Saudi Arabia: Abha, Aboarish, Alahsa, Albaha, Alhafouf, Aljouf, Alkharj, AlKhobar, AlQunfudah, Alrass, Aseer, Buraidah, Dammam, Dhahran, Dowmat Al-jandal, Hafer Albatin, Hail, Jazan, Jeddah, Khamis Mushait, Madinah, Majmaah, Makkah, Najran, Northern Border, Qassim, Qatif, Rafha, Riyadh, Sabya, Safwa, Sakaka, Samtah, Sharora, Tabuk, Taif, and Yanbu. In addition, participants came from different private clinics and 32 organizations, including Jouf University, Armed Forces Hospital in Southern Region, Batterjee Medical College, Dar Al Uloom University, Hail University, Ibn Sina National College, Imam Abdulrahman Bin Faisal University, Jazan University, King Abdulaziz University, King Fahad Armed Forces Hospital, King Faisal University, King Khalid University, King Saud Bin Abdulaziz University for Health Sciences, King Saud University, Majmaah University, Ministry of Health, Najran University, National Guard Health Affair, Prince Abdulrahman Institute of Higher Studies Dental, Prince Sattam Bin Abdulaziz University, Princess Nourah Bint Abdul Rahman University, Qassim University, Riyadh Elm University, Sattam University, Taibah University, Taif University, Umm Al-Qura University, University of Hail, Vision College, Vision Colleges in Jeddah, and Vision Colleges in Riyadh. Details of the participants’ demographic data are provided in Table 1.

**Table 1:** Participant demographic data (n  =  655).

Variable	Category	n	%
Gender	Male	317	48.40
	Female	338	51.60
Academic year/work status	2nd year	77	11.76
	3rd year	96	14.66
	4th year	81	12.37
	5th year	84	12.82
	6th year	98	14.96
	Intern	62	9.47
	General dentist or resident	126	19.24
	Specialist	11	1.68
	Consultant	20	3.05
Main study/work sector	Governmental	524	80.00
	Private	131	20.00
Region of current location in Saudi Arabia	Western	255	38.93
	Central	154	23.51
	Southern	73	11.15
	Eastern	99	15.11
	Northern	74	11.30
Nationality	Saudi	624	95.27
	Non-Saudi	31	4.73

### Motivations, barriers, and volunteering preferences (n  =  655)

Regarding the motivations for volunteering, participants had a variety of answers, as shown in Table 2. Female participants and dental students reported significantly higher percentages of motivations than male and dental interns/dentists in several items, as shown in Table 2.

**Table 2:** Participant motivations for volunteering (n  =  655).

Motive	Total n (%)	Gender			Working status		
		Male %	Female %	p-value	Dental student %	Intern or dentist %	p-value
Chance to learn in health-related field	554 (84.58)	80.76	88.17	0.009	88.99	75.8	<0.001
Gaining points for Saudi Commission of Health Specialty that allows for future training application	541 (82.60)	77.29	87.57	<0.001	85.55	76.71	0.005
Personal moral obligation to volunteer (including sense of duty and social dedication)	536 (81.83)	78.23	85.21	0.021	81.65	82.19	0.866
Religious reward for doing good deeds	531 (81.07)	82.33	79.88	0.423	80.73	81.74	0.758
Personal interest	531 (81.07)	79.18	82.84	0.232	84.17	74.89	0.004
Professional moral obligation to volunteer	506 (77.25)	73.19	81.07	0.016	78.67	74.43	0.222
Friend’s encouragement or to accompany friend	508 (77.56)	75.71	79.29	0.272	79.36	73.97	0.119
Good experience with previous volunteering	492 (75.11)	71.92	78.11	0.067	76.61	72.15	0.213
Networking with other people	480 (73.28)	71.29	75.15	0.265	75.23	69.41	0.112
Non-financial compensation (recognition, certificate, etc.)	473 (72.21)	68.14	76.04	0.024	72.94	70.78	0.561
Chance to learn in non-health area (organization, leadership, or others)	432 (65.95)	61.51	70.12	0.02	67.66	62.56	0.193
Patriotism (giving back to the country)	418 (63.82)	63.41	64.2	0.833	63.53	64.38	0.831
Ability to use the health-care services for family member	379 (57.86)	58.04	57.69	0.927	65.37	42.92	< .001
Media coverage during pandemic or disaster	338 (51.60)	47.32	55.62	0.034	51.15	52.51	0.742
Financial compensation	306 (46.72)	44.79	48.52	0.34	50.23	39.73	0.011

*statistical test: chi-square, with p<0.05 of significance level.

Participants reported several barriers to volunteering, which are shown in Table 3. There were also differences in volunteering preferences, as shown in Table 4. Some of the barriers and volunteering preferences were significantly different according to gender and working status. For example, the percentages of "Lack of academic/work support for time off to volunteer," "Lack of transportation," "Bad experience with previous volunteering," and "Parents/family disapproval" were significantly higher (p < 0.05) among females. However, the barrier of "Feeling that volunteering is not real and it is just to show off" was significantly higher among males (p = 0.007). Additionally, dental students reported significantly higher percentages (p < 0.05) for the barriers "Feeling unprepared to volunteer," "Fear of personal or family infection," and "Parents/family disapproval" compared to dentists. Conversely, dentists reported significantly higher percentages (p < 0.05) for the barriers "Feeling that volunteering is not the solution for the inadequacy of access to dental services" and "Feeling that volunteering is not real and it is just to show off" compared to dental students. This is shown in Table 3.

**Table 3:** Participants’ barriers to volunteering (n  =  655).

Barrier	Total n (%)	Gender			Working status		
		Male %	Female %	p-value	Dental student %	Intern or dentist %	p-value
Time constraints	488 (74.50)	71.92	76.92	0.142	75.46	72.6	0.429
Lack of opportunities related to dentistry	447 (68.24)	66.25	70.12	0.287	70.18	64.38	0.133
Lack of academic/work support for time off to volunteer	382 (58.32)	53.31	63.02	0.012	59.86	55.25	0.259
Feeling unprepared to volunteer	367 (56.03)	56.47	55.62	0.828	60.09	47.95	0.003
Feeling that volunteering is not the solution for the inadequacy of access to dental services	367 (56.03)	57.1	55.03	0.594	48.62	70.78	<0.001
Fear of transmitting the infection to family or friends	303 (46.26)	44.16	48.22	0.298	48.62	41.55	0.087
Lack of personal interest	302 (46.11)	48.9	43.49	0.166	46.56	45.21	0.743
Volunteering is in unfriendly environment	302 (46.11)	42.59	49.41	0.08	44.95	48.4	0.404
Lack of transportation	298 (45.50)	37.22	53.25	<0.001	45.87	44.75	0.785
Feeling that volunteering is not real and it is just to show off	293 (44.73)	50.16	39.64	0.007	38.99	56.16	<0.001
Fear of personal or family infection	262 (40.00)	36.59	43.2	0.085	43.12	33.79	0.021
Lack of personal protective equipment (PPE)	253 (38.63)	38.17	39.05	0.817	36.47	42.92	0.109
Bad experience with previous volunteering	210 (32.06)	28.08	35.8	0.034	31.19	33.79	0.502
Parents/family disapproval	189 (28.85)	22.4	34.91	<0.001	31.65	23.29	0.026

*statistical test: chi-square, with p<0.05 of significance level.

**Table 4:** Volunteering preferences (n  =  655).

Volunteering preferences	Total n (%)	Gender			Working status		
		Male %	Female %	p-value	Dental student %	Intern or dentist %	p-value
Assistance under the supervision of another health practitioner (medical doctor or dentist)	492 (75.11)	71.61	78.4	0.044	81.19	63.01	<0.001
Manage non-invasive dental cases (such as fissure sealant or teledentistry)	478 (72.98)	68.14	77.51	0.007	75.92	67.12	0.017
Manage invasive dental cases (such as severe oral trauma)	414 (63.21)	64.98	61.54	0.361	67.66	54.34	<0.001
Manage non-invasive medical cases (such as hypoglycemia, triage, or taking vital signs)	382 (58.32)	58.99	57.69	0.736	63.76	47.49	<0.001
Manage invasive medical cases (such as severe injury)	356 (54.35)	59.31	49.7	0.014	57.8	47.49	0.012
Non-medical volunteering (such as transportation of supplies, call center)	262 (40.00)	40.06	39.94	0.975	42.66	34.7	0.05

*statistical test: chi-square, with p<0.05 of significance level.

### Previous volunteering experience (n  =  488)

Among the participants, there were 488 (74.50%) who had previously participated in volunteer work. The participation in volunteering was significantly higher (p < 0.001) among interns and dentists (94.52%) than dental students (64.44%) and significantly higher (p < 0.001) among participants from the private sector (90.83%) than those in the governmental sector (70.41%). However, the participation in volunteer work was not statistically significant when tested against gender, region, or nationality. Among those who had participated in volunteering (n = 488), there were 480 (98.36%) who volunteered in Saudi Arabia and 34 (6.97%) who volunteered outside of Saudi Arabia. In addition, among those participants, 226 (46.31%) volunteered during the COVID-19 pandemic.

The participants who had volunteered did so in a variety of activities; these previous experiences are shown in Table 5. Also, those participants had different actual compensations when they volunteered before, which is shown in Table 6.

**Table 5:** Participants’ previous experiences with volunteering (n  =  488).

Previous experience in volunteering	n	%
Assistance under the supervision of another health-care practitioner (medical doctor or a dentist)	242	49.59
Manage non-invasive dental cases (such as fissure sealant or teledentistry)	238	48.77
Manage non-invasive medical cases (such as hypoglycemia, triage, vaccination, or taking vital signs)	212	43.44
Non-medical volunteering (such as transportation of supplies, call center)	203	41.60
Manage invasive dental cases (such as severe oral trauma)	104	21.31
Manage invasive medical cases (such as severe injury)	87	17.83

**Table 6:** Participants’ previous compensation received for volunteering (n  =  488).

Previous compensation for volunteering	n	%
Certificate	408	83.61
Continuing medical education hours (CME)	215	44.06
Public recognition	176	36.07
Personal or family priority access to health care	89	18.24
Did not get any compensation at all	60	12.3
I only got reimbursed for personal expenses (calls, transportation, meals, etc.)	54	11.07
I got financial compensation for volunteering	50	10.25

Regarding gender, males had significantly participated more in managed invasive dental cases (28.16%, p < 0.001) and invasive medical cases (22.86%, p  =  0.004) than females (14.40% and 12.76%, respectively). Additionally, there were significantly more (p  =  0.005) male participants who volunteered outside of Saudi Arabia (10.20%) than females (3.70%). Other previous experience included volunteering in Saudi Arabia and volunteering during COVID-19, and none of the compensation types from previous volunteer work were significantly different between males and females.

Regarding studying or work status, interns/dentists participated in non-invasive dental cases (62.80%) significantly more (p > 0.001) than dental students (38.43%). However, dental students volunteered in non-medical circumstances (45.55%) significantly more (p  =  0.039) than dental interns/dentists (36.23%). Also interns/dentists received a certificate as compensation (92.27%) significantly more (p < 0.001) than dental students (77.22%). In fact, dental students did not receive any compensation at all (15.30%) significantly more (p  =  0.018) than dental interns/dentists (0.018%). Other previous experience volunteering in Saudi Arabia, volunteering outside of Saudi Arabia, and volunteering during COVID-19, as well as other forms of compensation from previous volunteer work, were not significantly different between males and females.

## Discussion

This study aimed to assess the motivations for, barriers to, and experiences of volunteering among dental students and dentists in Saudi Arabia. The data were collected from multiple cities and organization types across Saudi Arabia. The most motivating factor for volunteering was the chance to learn in a health-related field, and the least motivating factor was monetary compensation. Females had significantly higher scores on motivations than male participants. The most prominent barrier was time constraints, while the least important barrier was parental or family disapproval. The most preferred area for volunteering was assistance under the supervision of another health-care practitioner, while the least preferred area for volunteering was non-medical work. There was 74.50% of respondents who had previously participated in volunteer work. Among those, 98.36% volunteered in Saudi Arabia, and 6.97% volunteered outside of Saudi Arabia, with 46.31% volunteering during the COVID-19 pandemic.

In our study, the highest ranking reported motivations (81.07% to 84.58%) were the chance to learn in a health-related field, gaining points for the Saudi Commission of Health Specialty that allows for future training applications, personal moral obligations to volunteer, religious rewards, and personal interest. This was very similar to the previous systematic review of health-care students [24]. However, the systematic review reported financial compensation as a main motivation, but our results found it to be the least motivating (46.72%). This may be due to cultural factors and beliefs in Saudi Arabia and Islamic countries. This was explained by the fact that volunteering is a form of charity. Among Muslims, there are two economic theologies related to charity, which are economy of thawab (rewards by God) and economy of baraka (blessing of one’s own money after volunteering) [30]. In fact, the religious motivation was previously noted in a recent study in Saudi Arabia [21]. One of the factors that increases the importance of religious motivations among participants in Saudi Arabia is that the country hosts Umrah and Hajj religious events every year, which are opportunities for many health-care volunteers to participate [31]. It is important to emphasize that distinct types of volunteer projects become necessary in response to varying circumstances and changing temporal factors, which may be influenced by the country or geographic region. This diversity introduces an additional layer of complexity when undertaking literature reviews and attempting to make comparisons across studies with our results.

Gaining the Saudi Commission of Health Specialty application points was another main motivation. These points improve an applicant’s chances of acceptance to highly competitive dental residency programs in Saudi Arabia. A previous study in the United States found that continuing education credits was a main motivation for volunteering [32]. While this could lead us to speculate that motivations among dental students and dentists have similarities across countries, some local motives related to religion and career path development are important to address in relation to the population and country. It is important to understand dental volunteers’ motives so that understanding can be the grounds upon which to base enhancements to volunteerism in Saudi Arabia.

The most reported barriers in our study were time constraints, lack of opportunities related to dentistry, lack of academic or work support while taking time off to volunteer, and feeling unprepared for volunteering. Some of these factors have been reported in previous studies [5, 21, 26], however, there was a difference in the most reported barriers. In other words, the most frequently reported barriers differed in ranking from one study to another. In many studies, fear of infection and fear of disease transmission were reported as major barriers [14, 33, 34]; however, in our study, they had lower prevalence. The reason for the difference might be the fact that previous studies were conducted in 2020 to 2021, when COVID-19 was at its peak and quarantines had been instituted in many countries [35]. Conversely, our study was conducted in 2023, after most of the COVID-19 related restrictions were lifted.

Our study’s results showed that a lack of opportunities related to dentistry was an important barrier. This was not reported in previous studies, and one reason for the difference might be that the earlier studies investigated medical or health-care participants in general [15–17]. It is important to emphasize that we need more studies related to the field of dentistry specifically in order to address problems and issues related to the dental field.

One of the interesting points in our results is that transportation was one of the reported barriers. This barrier was reported significantly more by female participants (53.25%) than males (37.22%), a result that was also found in a previous study in Saudi Arabia [21]. This was explained by the fact that women were only recently allowed to drive in Saudi Arabia [21]. Nevertheless, the percentage of males who reported transportation as a barrier was 37.22%. This is a high percentage and should be investigated more because our current data cannot explain this result.

A surprising result was that parental or family disapproval was the least frequently reported barrier (28.85%), which contrasts with the research team’s expectations. We had assumed the percentage would be higher, as reported in a previous study in Nigeria (58.1%) [22]. Both studies found that females reported this barrier significantly more than males. It is very important to address these barriers during the journey to reach Saudi Vision 2030 for volunteering.

Our results indicated that 74.50% of our respondents had volunteered previously, which is higher than studies conducted in Japan (43%) [13], Pakistan (64%) [20], and even the previous study from Saudi Arabia (30.31%) [21] that investigated dental students. The reason for this difference could be due to our study including working dentists, who had a significantly higher percentage of previous volunteers than did the students. Our study might be the first to mention the percentage of dentists and dental students who volunteered outside of Saudi Arabia (6.97%), although the percentage is lower than that reported in the United States (41.9%) [23]. This may be due to opportunities in Saudi Arabia being not as widely seen and available as is common in the United States.

Most of the participants who had previously volunteered received a certificate as compensation (83.61%), with only 11.07% being compensated for personal expenses while volunteering and 12.3% receiving no compensation at all. We think that volunteers should be compensated by authorities for their personal expenses during volunteering, if possible, because they are offering their time, efforts, and experience. In fact, those who volunteer for health research projects are compensated or reimbursed [36], which is somewhat similar. We believe that ignoring this point might lead to exploitation of volunteers, especially when there is money allocated by authorities for volunteering. Thus, we recommend that health volunteering authorities in Saudi Arabia address this issue in some way during policymaking to achieve the Saudi Vision 2023 of one million volunteers [29].

Although this study encompassed participants from various cities across Saudi Arabia, the sampling method employed (convenience) is a non-probability sampling technique. The questionnaire was self-administered, rendering the results susceptible to self-reported bias. This raises concerns regarding the external validity, or generalizability, of the results. It is advisable that future studies seek to validate these findings through the utilization of random sampling methods. Also, future studies could consider employing qualitative interviews and focus groups as research methods to delve into various aspects of motives and barriers related to volunteering among dental professionals.

## Conclusion

There is a high percentage of dental students and dentists who participate in volunteering in Saudi Arabia. The most motivating factors that should be highlighted to promote volunteering are the chance to learn in a health-related field, gaining points to the Saudi Commission of Health Specialty, personal moral obligations to volunteer, and religious rewards. However, there are some barriers that need to be addressed: time constraints, lack of opportunities related to dentistry, lack of academic or work support while away for volunteering, feeling unprepared to volunteer, and transportation problems, particularly among females. These factors need to be addressed to improve volunteering in light of Saudi Arabia Vision 2030’s targeted key performance indicator.

## Supporting information

S1 File Study raw data. (PDF)

## List of abbreviations

CME: Continuing medical education hours
PPE: personal protective equipment
SD: Standard deviation
