# Supplementary material for: Understanding volunteerism among dental students and professionals to reach Saudi Arabia Vision 2030 goals
Source: PLoS One. 2024 Jan 10;19(1):e0296745. doi: 10.1371/journal.pone.0296745 (PMC10781196; doi:10.1371/journal.pone.0296745)
Supplement: S1 File — (PDF) [file pone.0296745.s001.pdf]

K68 SPSS v14.sav

	SN	Group	Gender	Age	Work	Work2	Work3	Sector
1	1.00	1.00	1.00	20.00	2.00	1.00	1.00	1.00
2	2.00	1.00	1.00	21.00	2.00	1.00	1.00	1.00
3	6.00	1.00	1.00	21.00	4.00	1.00	1.00	1.00
4	56.00	1.00	2.00	22.00	6.00	1.00	1.00	1.00
5	111.00	1.00	1.00	22.00	6.00	1.00	1.00	1.00
6	157.00	1.00	1.00	22.00	4.00	1.00	1.00	2.00
7	311.00	1.00	1.00	20.00	3.00	1.00	1.00	1.00
8	480.00	1.00	1.00	36.00	9.00	3.00	2.00	2.00
9	490.00	1.00	1.00	28.00	8.00	2.00	2.00	2.00
10	603.00	2.00	2.00	24.00	6.00	1.00	1.00	1.00
11	3.00	1.00	1.00	20.00	2.00	1.00	1.00	1.00
12	4.00	1.00	1.00	23.00	4.00	1.00	1.00	1.00
13	5.00	1.00	1.00	22.00	4.00	1.00	1.00	1.00
14	7.00	1.00	1.00	24.00	5.00	1.00	1.00	1.00
15	10.00	1.00	1.00	24.00	6.00	1.00	1.00	1.00
16	11.00	1.00	2.00	50.00	10.00	3.00	2.00	1.00
17	12.00	1.00	2.00	34.00	10.00	3.00	2.00	1.00
18	13.00	1.00	2.00	34.00	9.00	3.00	2.00	1.00
19	14.00	1.00	1.00	38.00	10.00	3.00	2.00	1.00
20	16.00	1.00	1.00	34.00	9.00	3.00	2.00	1.00
21	17.00	1.00	1.00	22.00	5.00	1.00	1.00	1.00
22	18.00	1.00	1.00	25.00	6.00	1.00	1.00	1.00
23	20.00	1.00	1.00	25.00	6.00	1.00	1.00	1.00
24	21.00	1.00	1.00	23.00	6.00	1.00	1.00	1.00
25	22.00	1.00	1.00	20.00	2.00	1.00	1.00	1.00
26	25.00	1.00	1.00	18.00	2.00	1.00	1.00	2.00
27	26.00	1.00	1.00	23.00	5.00	1.00	1.00	1.00
28	27.00	1.00	1.00	23.00	5.00	1.00	1.00	1.00
29	28.00	1.00	2.00	23.00	6.00	1.00	1.00	1.00
30	29.00	1.00	2.00	24.00	6.00	1.00	1.00	1.00
31	31.00	1.00	1.00	21.00	4.00	1.00	1.00	1.00
32	32.00	1.00	1.00	22.00	4.00	1.00	1.00	1.00
33	33.00	1.00	2.00	22.00	4.00	1.00	1.00	2.00
34	34.00	1.00	2.00	23.00	6.00	1.00	1.00	1.00
35	37.00	1.00	2.00	24.00	6.00	1.00	1.00	1.00
36	38.00	1.00	2.00	23.00	5.00	1.00	1.00	1.00
37	39.00	1.00	2.00	22.00	5.00	1.00	1.00	1.00
38	40.00	1.00	2.00	23.00	5.00	1.00	1.00	1.00
39	41.00	1.00	1.00	35.00	10.00	3.00	2.00	1.00
40	42.00	1.00	2.00	20.00	3.00	1.00	1.00	1.00
41	43.00	1.00	1.00	24.00	7.00	2.00	2.00	1.00
42	46.00	1.00	2.00	26.00	8.00	2.00	2.00	1.00

	Region	Nationality	M1	M2	M3	M4	M5	M6
1	1.00	1.00	1.00	1.00	2.00	2.00	1.00	1.00
2	1.00	1.00	1.00	1.00	1.00	1.00	1.00	1.00
3	1.00	1.00	1.00	1.00	1.00	1.00	1.00	2.00
4	1.00	1.00	1.00	1.00	1.00	1.00	1.00	1.00
5	4.00	1.00	1.00	1.00	1.00	1.00	1.00	2.00
6	2.00	2.00	2.00	1.00	2.00	1.00	1.00	1.00
7	4.00	1.00	2.00	2.00	2.00	2.00	2.00	1.00
8	1.00	2.00	1.00	1.00	1.00	1.00	2.00	2.00
9	1.00	1.00	1.00	2.00	2.00	1.00	1.00	1.00
10	1.00	1.00	2.00	2.00	1.00	1.00	1.00	1.00
11	1.00	1.00	1.00	1.00	2.00	1.00	1.00	1.00
12	1.00	1.00	1.00	1.00	1.00	1.00	1.00	1.00
13	1.00	1.00	1.00	1.00	2.00	1.00	1.00	1.00
14	1.00	1.00	1.00	1.00	2.00	1.00	1.00	1.00
15	1.00	1.00	1.00	1.00	1.00	1.00	1.00	2.00
16	2.00	1.00	1.00	2.00	2.00	1.00	1.00	2.00
17	1.00	1.00	1.00	1.00	2.00	1.00	2.00	1.00
18	1.00	1.00	1.00	2.00	2.00	1.00	2.00	1.00
19	1.00	1.00	1.00	1.00	1.00	1.00	2.00	2.00
20	1.00	1.00	1.00	2.00	1.00	1.00	1.00	1.00
21	1.00	1.00	1.00	1.00	2.00	2.00	1.00	1.00
22	1.00	1.00	1.00	1.00	2.00	1.00	1.00	1.00
23	5.00	1.00	1.00	1.00	2.00	1.00	2.00	1.00
24	1.00	1.00	1.00	1.00	1.00	1.00	1.00	1.00
25	5.00	1.00	1.00	1.00	1.00	1.00	1.00	1.00
26	1.00	1.00	2.00	2.00	1.00	2.00	1.00	1.00
27	1.00	1.00	1.00	1.00	2.00	1.00	1.00	1.00
28	1.00	1.00	1.00	1.00	1.00	1.00	1.00	1.00
29	1.00	1.00	1.00	2.00	2.00	1.00	1.00	1.00
30	1.00	1.00	1.00	1.00	1.00	1.00	1.00	1.00
31	1.00	1.00	2.00	2.00	2.00	2.00	2.00	2.00
32	1.00	1.00	1.00	1.00	1.00	2.00	1.00	1.00
33	2.00	2.00	1.00	1.00	1.00	1.00	2.00	1.00
34	4.00	1.00	1.00	2.00	1.00	1.00	1.00	1.00
35	1.00	1.00	1.00	2.00	2.00	1.00	1.00	2.00
36	1.00	1.00	2.00	2.00	2.00	1.00	1.00	1.00
37	1.00	1.00	1.00	1.00	2.00	1.00	1.00	1.00
38	1.00	1.00	1.00	2.00	2.00	1.00	1.00	2.00
39	1.00	1.00	2.00	2.00	2.00	2.00	2.00	2.00
40	1.00	1.00	1.00	1.00	2.00	1.00	1.00	1.00
41	1.00	1.00	1.00	2.00	1.00	1.00	1.00	1.00
42	1.00	1.00	1.00	1.00	1.00	1.00	1.00	1.00

	M7	M8	M9	M10	M11	M12	M13	M14
1	1.00	1.00	1.00	1.00	1.00	1.00	1.00	2.00
2	1.00	1.00	2.00	1.00	2.00	2.00	1.00	2.00
3	1.00	1.00	1.00	1.00	2.00	1.00	1.00	1.00
4	1.00	1.00	1.00	1.00	1.00	1.00	1.00	1.00
5	1.00	1.00	2.00	2.00	1.00	2.00	2.00	2.00
6	1.00	1.00	1.00	1.00	2.00	1.00	1.00	1.00
7	2.00	1.00	1.00	1.00	1.00	1.00	2.00	1.00
8	1.00	1.00	2.00	1.00	2.00	2.00	2.00	1.00
9	2.00	2.00	1.00	1.00	1.00	1.00	2.00	1.00
10	1.00	1.00	1.00	1.00	1.00	1.00	1.00	1.00
11	1.00	1.00	2.00	1.00	1.00	1.00	1.00	1.00
12	2.00	1.00	1.00	1.00	2.00	1.00	1.00	1.00
13	1.00	1.00	2.00	1.00	2.00	1.00	1.00	1.00
14	1.00	1.00	1.00	1.00	1.00	1.00	1.00	1.00
15	1.00	1.00	2.00	1.00	1.00	2.00	2.00	1.00
16	2.00	2.00	2.00	1.00	2.00	2.00	2.00	2.00
17	2.00	2.00	2.00	1.00	2.00	1.00	2.00	2.00
18	1.00	1.00	1.00	1.00	1.00	1.00	2.00	2.00
19	1.00	1.00	1.00	1.00	2.00	2.00	2.00	2.00
20	1.00	1.00	1.00	1.00	1.00	1.00	2.00	1.00
21	1.00	1.00	2.00	1.00	1.00	1.00	1.00	1.00
22	1.00	2.00	2.00	1.00	2.00	2.00	2.00	2.00
23	1.00	1.00	2.00	1.00	1.00	1.00	2.00	1.00
24	1.00	1.00	1.00	2.00	2.00	1.00	2.00	1.00
25	1.00	1.00	1.00	1.00	1.00	1.00	1.00	1.00
26	2.00	1.00	1.00	2.00	2.00	1.00	1.00	2.00
27	2.00	2.00	1.00	1.00	1.00	1.00	1.00	2.00
28	1.00	1.00	1.00	1.00	2.00	2.00	1.00	1.00
29	2.00	1.00	1.00	1.00	2.00	1.00	2.00	1.00
30	1.00	1.00	1.00	1.00	1.00	1.00	1.00	1.00
31	2.00	2.00	2.00	2.00	2.00	2.00	2.00	2.00
32	1.00	2.00	1.00	1.00	1.00	1.00	1.00	1.00
33	2.00	1.00	2.00	1.00	1.00	2.00	1.00	2.00
34	2.00	1.00	1.00	1.00	1.00	1.00	1.00	1.00
35	1.00	1.00	1.00	1.00	2.00	1.00	2.00	1.00
36	2.00	1.00	1.00	2.00	2.00	1.00	2.00	1.00
37	1.00	1.00	1.00	1.00	2.00	1.00	2.00	1.00
38	2.00	1.00	1.00	1.00	1.00	1.00	1.00	1.00
39	2.00	2.00	2.00	2.00	2.00	2.00	2.00	2.00
40	1.00	1.00	2.00	1.00	1.00	1.00	2.00	1.00
41	1.00	2.00	2.00	1.00	1.00	1.00	2.00	1.00
42	1.00	1.00	2.00	1.00	2.00	1.00	2.00	1.00

	M15	B1	B2	B3	B4	B5	B6	B7
1	1.00	2.00	2.00	2.00	2.00	2.00	1.00	1.00
2	1.00	1.00	2.00	2.00	1.00	2.00	1.00	1.00
3	1.00	1.00	2.00	2.00	1.00	2.00	2.00	2.00
4	1.00	2.00	1.00	1.00	2.00	1.00	1.00	1.00
5	1.00	1.00	1.00	2.00	1.00	2.00	2.00	1.00
6	2.00	1.00	2.00	1.00	1.00	2.00	1.00	1.00
7	1.00	1.00	2.00	2.00	1.00	1.00	2.00	2.00
8	1.00	2.00	1.00	1.00	1.00	2.00	2.00	2.00
9	1.00	2.00	1.00	2.00	2.00	2.00	2.00	2.00
10	1.00	1.00	2.00	2.00	1.00	2.00	1.00	1.00
11	1.00	1.00	1.00	2.00	1.00	1.00	1.00	1.00
12	1.00	2.00	1.00	2.00	2.00	2.00	1.00	1.00
13	1.00	2.00	2.00	2.00	2.00	2.00	2.00	2.00
14	1.00	1.00	2.00	1.00	1.00	1.00	1.00	1.00
15	2.00	2.00	1.00	2.00	2.00	1.00	1.00	1.00
16	1.00	1.00	2.00	2.00	1.00	2.00	2.00	2.00
17	1.00	2.00	1.00	1.00	1.00	2.00	2.00	2.00
18	1.00	2.00	2.00	2.00	1.00	1.00	2.00	2.00
19	1.00	1.00	2.00	2.00	1.00	2.00	2.00	2.00
20	1.00	2.00	1.00	2.00	1.00	2.00	2.00	2.00
21	1.00	2.00	2.00	1.00	1.00	2.00	2.00	2.00
22	1.00	2.00	2.00	1.00	1.00	1.00	2.00	2.00
23	1.00	1.00	2.00	1.00	1.00	2.00	1.00	2.00
24	1.00	1.00	2.00	2.00	2.00	1.00	2.00	2.00
25	1.00	2.00	2.00	2.00	2.00	1.00	1.00	1.00
26	1.00	1.00	2.00	2.00	1.00	1.00	1.00	2.00
27	1.00	1.00	2.00	1.00	1.00	1.00	2.00	1.00
28	1.00	1.00	1.00	1.00	1.00	2.00	1.00	2.00
29	1.00	1.00	2.00	2.00	1.00	1.00	2.00	2.00
30	1.00	1.00	1.00	1.00	1.00	1.00	1.00	1.00
31	2.00	2.00	2.00	2.00	2.00	2.00	2.00	2.00
32	1.00	2.00	1.00	1.00	1.00	2.00	2.00	1.00
33	1.00	1.00	2.00	2.00	2.00	2.00	1.00	1.00
34	1.00	1.00	2.00	2.00	1.00	2.00	1.00	1.00
35	1.00	2.00	1.00	2.00	1.00	2.00	2.00	2.00
36	1.00	1.00	2.00	2.00	1.00	1.00	2.00	2.00
37	1.00	1.00	2.00	2.00	1.00	2.00	1.00	1.00
38	2.00	1.00	2.00	1.00	1.00	1.00	1.00	1.00
39	2.00	1.00	1.00	1.00	1.00	1.00	1.00	1.00
40	1.00	1.00	1.00	2.00	2.00	2.00	1.00	1.00
41	1.00	1.00	1.00	1.00	2.00	2.00	2.00	1.00
42	1.00	2.00	2.00	2.00	1.00	2.00	2.00	2.00

	B8	B9	B10	B11	B12	B13	B14	W1
1	1.00	2.00	2.00	2.00	2.00	2.00	2.00	1.00
2	2.00	2.00	2.00	1.00	2.00	2.00	2.00	2.00
3	2.00	2.00	2.00	1.00	2.00	2.00	2.00	1.00
4	2.00	2.00	2.00	1.00	1.00	1.00	1.00	2.00
5	1.00	1.00	1.00	1.00	1.00	1.00	1.00	1.00
6	1.00	2.00	1.00	1.00	2.00	2.00	2.00	2.00
7	2.00	2.00	1.00	2.00	2.00	2.00	2.00	2.00
8	1.00	1.00	2.00	2.00	2.00	2.00	2.00	1.00
9	2.00	2.00	2.00	1.00	2.00	2.00	2.00	2.00
10	1.00	2.00	1.00	1.00	1.00	2.00	2.00	2.00
11	1.00	1.00	1.00	1.00	2.00	2.00	1.00	1.00
12	1.00	2.00	2.00	1.00	2.00	2.00	1.00	2.00
13	1.00	2.00	2.00	1.00	2.00	2.00	1.00	2.00
14	1.00	1.00	1.00	2.00	1.00	1.00	1.00	1.00
15	2.00	1.00	2.00	2.00	2.00	1.00	1.00	2.00
16	2.00	1.00	1.00	1.00	2.00	2.00	2.00	2.00
17	1.00	1.00	1.00	2.00	2.00	2.00	1.00	2.00
18	1.00	1.00	1.00	1.00	1.00	2.00	1.00	1.00
19	2.00	2.00	2.00	2.00	2.00	2.00	2.00	2.00
20	2.00	2.00	2.00	1.00	2.00	2.00	2.00	2.00
21	1.00	1.00	1.00	1.00	2.00	2.00	2.00	2.00
22	2.00	2.00	2.00	2.00	2.00	2.00	2.00	1.00
23	2.00	1.00	1.00	1.00	1.00	2.00	2.00	2.00
24	2.00	1.00	1.00	2.00	2.00	1.00	2.00	1.00
25	2.00	2.00	2.00	2.00	2.00	2.00	2.00	1.00
26	2.00	1.00	1.00	2.00	1.00	1.00	1.00	1.00
27	2.00	2.00	2.00	2.00	1.00	1.00	1.00	2.00
28	2.00	2.00	2.00	2.00	2.00	2.00	2.00	2.00
29	2.00	1.00	1.00	1.00	2.00	2.00	2.00	1.00
30	1.00	1.00	1.00	1.00	1.00	1.00	1.00	1.00
31	2.00	2.00	2.00	2.00	2.00	2.00	2.00	2.00
32	1.00	2.00	2.00	2.00	1.00	2.00	1.00	2.00
33	2.00	2.00	1.00	2.00	2.00	2.00	1.00	2.00
34	2.00	2.00	1.00	2.00	2.00	1.00	1.00	2.00
35	1.00	2.00	1.00	1.00	1.00	1.00	1.00	1.00
36	2.00	1.00	1.00	1.00	2.00	2.00	1.00	2.00
37	2.00	1.00	2.00	1.00	2.00	2.00	2.00	2.00
38	1.00	1.00	1.00	1.00	1.00	1.00	1.00	1.00
39	1.00	1.00	1.00	1.00	1.00	1.00	1.00	1.00
40	2.00	1.00	1.00	1.00	2.00	2.00	2.00	1.00
41	1.00	1.00	1.00	1.00	2.00	1.00	1.00	1.00
42	1.00	1.00	1.00	1.00	2.00	2.00	2.00	1.00

	W2	W3	W4	W5	W6	E1	V1	V2
1	1.00	1.00	1.00	1.00	1.00	2.00	1.00	1.00
2	2.00	2.00	2.00	2.00	2.00	2.00	2.00	2.00
3	1.00	2.00	1.00	1.00	2.00	1.00	2.00	2.00
4	2.00	2.00	1.00	1.00	1.00	1.00	2.00	1.00
5	1.00	1.00	1.00	1.00	1.00	1.00	1.00	1.00
6	1.00	1.00	1.00	1.00	1.00	1.00	2.00	2.00
7	2.00	1.00	1.00	1.00	1.00	1.00	2.00	2.00
8	1.00	2.00	2.00	1.00	2.00	1.00	2.00	1.00
9	1.00	2.00	2.00	1.00	1.00	1.00	2.00	2.00
10	2.00	1.00	1.00	1.00	1.00	1.00	2.00	2.00
11	2.00	2.00	2.00	1.00	1.00	2.00	2.00	1.00
12	1.00	1.00	1.00	1.00	2.00	1.00	2.00	2.00
13	2.00	1.00	2.00	1.00	2.00	1.00	2.00	2.00
14	1.00	1.00	1.00	1.00	1.00	1.00	2.00	2.00
15	2.00	1.00	1.00	1.00	2.00	1.00	2.00	2.00
16	2.00	1.00	2.00	2.00	2.00	1.00	2.00	2.00
17	2.00	1.00	1.00	1.00	2.00	1.00	2.00	2.00
18	2.00	2.00	2.00	1.00	2.00	1.00	2.00	2.00
19	2.00	1.00	1.00	1.00	2.00	1.00	2.00	2.00
20	2.00	1.00	2.00	1.00	2.00	1.00	2.00	2.00
21	2.00	1.00	2.00	1.00	2.00	1.00	2.00	2.00
22	1.00	2.00	2.00	2.00	2.00	1.00	2.00	2.00
23	1.00	1.00	2.00	2.00	1.00	1.00	1.00	2.00
24	1.00	1.00	1.00	1.00	1.00	1.00	2.00	2.00
25	1.00	1.00	1.00	1.00	1.00	1.00	1.00	1.00
26	2.00	1.00	1.00	2.00	1.00	1.00	1.00	2.00
27	2.00	1.00	1.00	1.00	1.00	1.00	2.00	2.00
28	2.00	2.00	2.00	1.00	2.00	1.00	2.00	2.00
29	1.00	2.00	2.00	1.00	2.00	1.00	2.00	2.00
30	1.00	2.00	2.00	2.00	1.00	1.00	2.00	2.00
31	2.00	2.00	2.00	2.00	2.00	1.00	2.00	2.00
32	1.00	1.00	1.00	1.00	2.00	1.00	2.00	2.00
33	2.00	1.00	1.00	1.00	1.00	1.00	2.00	2.00
34	2.00	1.00	1.00	1.00	1.00	1.00	2.00	2.00
35	2.00	1.00	1.00	1.00	2.00	1.00	2.00	2.00
36	2.00	1.00	2.00	1.00	1.00	1.00	2.00	2.00
37	2.00	1.00	1.00	1.00	2.00	1.00	2.00	2.00
38	1.00	1.00	1.00	2.00	2.00	1.00	2.00	1.00
39	2.00	1.00	2.00	1.00	2.00	1.00	2.00	2.00
40	1.00	2.00	2.00	1.00	2.00	1.00	2.00	2.00
41	1.00	2.00	1.00	1.00	1.00	1.00	1.00	1.00
42	1.00	2.00	2.00	1.00	2.00	1.00	2.00	2.00

	V3	V4	V5	V6	E2	E3	E4	C1
1	1.00	1.00	1.00	1.00	2.00	2.00	2.00	2.00
2	2.00	2.00	2.00	2.00	2.00	2.00	2.00	2.00
3	1.00	2.00	1.00	2.00	2.00	1.00	2.00	2.00
4	1.00	1.00	1.00	2.00	2.00	1.00	2.00	1.00
5	1.00	1.00	1.00	1.00	2.00	2.00	1.00	1.00
6	2.00	2.00	2.00	1.00	2.00	1.00	2.00	2.00
7	2.00	2.00	2.00	2.00	2.00	1.00	2.00	2.00
8	2.00	1.00	1.00	2.00	2.00	1.00	1.00	2.00
9	2.00	2.00	2.00	2.00	2.00	2.00	1.00	2.00
10	2.00	2.00	2.00	2.00	2.00	2.00	2.00	2.00
11	1.00	2.00	1.00	2.00	1.00	2.00	2.00	1.00
12	2.00	1.00	1.00	1.00	1.00	2.00	2.00	2.00
13	1.00	2.00	1.00	2.00	1.00	2.00	2.00	2.00
14	1.00	1.00	1.00	1.00	1.00	2.00	1.00	2.00
15	1.00	1.00	1.00	2.00	1.00	2.00	1.00	2.00
16	1.00	2.00	2.00	2.00	1.00	2.00	2.00	2.00
17	1.00	2.00	1.00	1.00	1.00	2.00	2.00	2.00
18	2.00	2.00	1.00	2.00	1.00	2.00	1.00	2.00
19	1.00	1.00	2.00	2.00	1.00	2.00	2.00	2.00
20	1.00	2.00	2.00	2.00	1.00	1.00	2.00	2.00
21	2.00	1.00	2.00	2.00	1.00	2.00	2.00	2.00
22	2.00	2.00	1.00	2.00	1.00	2.00	2.00	2.00
23	1.00	1.00	1.00	2.00	1.00	2.00	2.00	2.00
24	2.00	1.00	1.00	1.00	1.00	2.00	1.00	1.00
25	1.00	1.00	1.00	1.00	1.00	2.00	2.00	2.00
26	1.00	1.00	2.00	1.00	1.00	2.00	1.00	2.00
27	1.00	1.00	1.00	2.00	1.00	2.00	2.00	2.00
28	1.00	1.00	2.00	1.00	1.00	2.00	1.00	2.00
29	2.00	2.00	1.00	2.00	1.00	2.00	2.00	2.00
30	2.00	1.00	2.00	2.00	1.00	2.00	2.00	2.00
31	2.00	2.00	2.00	2.00	1.00	1.00	2.00	1.00
32	2.00	2.00	1.00	1.00	1.00	2.00	2.00	2.00
33	2.00	1.00	1.00	2.00	1.00	2.00	2.00	2.00
34	2.00	2.00	1.00	1.00	1.00	2.00	2.00	2.00
35	2.00	1.00	2.00	2.00	1.00	2.00	2.00	2.00
36	2.00	2.00	1.00	1.00	1.00	2.00	1.00	2.00
37	2.00	1.00	1.00	2.00	1.00	2.00	1.00	2.00
38	1.00	2.00	2.00	1.00	1.00	2.00	2.00	2.00
39	1.00	2.00	2.00	2.00	1.00	2.00	2.00	2.00
40	2.00	2.00	2.00	2.00	1.00	2.00	2.00	2.00
41	1.00	2.00	1.00	1.00	1.00	2.00	1.00	2.00
42	1.00	1.00	2.00	1.00	1.00	2.00	2.00	2.00

	C2	C3	C4	C5	C6	C7	filter_$
1	2.00	2.00	2.00	2.00	2.00	2.00	0
2	2.00	2.00	2.00	2.00	2.00	2.00	0
3	2.00	2.00	2.00	2.00	2.00	1.00	1
4	1.00	1.00	1.00	1.00	1.00	2.00	1
5	1.00	1.00	1.00	1.00	1.00	2.00	1
6	2.00	1.00	2.00	2.00	2.00	2.00	1
7	2.00	1.00	2.00	2.00	2.00	2.00	1
8	2.00	2.00	2.00	2.00	2.00	1.00	1
9	2.00	2.00	2.00	2.00	2.00	2.00	1
10	2.00	2.00	2.00	2.00	2.00	2.00	1
11	1.00	1.00	1.00	2.00	1.00	2.00	0
12	1.00	1.00	2.00	2.00	2.00	2.00	1
13	2.00	1.00	1.00	2.00	1.00	2.00	1
14	1.00	1.00	1.00	1.00	2.00	2.00	1
15	2.00	1.00	2.00	2.00	2.00	2.00	1
16	2.00	1.00	2.00	2.00	2.00	2.00	1
17	2.00	1.00	2.00	1.00	2.00	2.00	1
18	1.00	1.00	2.00	1.00	2.00	2.00	1
19	2.00	1.00	1.00	1.00	2.00	2.00	1
20	2.00	1.00	1.00	2.00	2.00	2.00	1
21	2.00	1.00	1.00	2.00	2.00	2.00	1
22	2.00	1.00	2.00	2.00	2.00	2.00	1
23	1.00	2.00	1.00	1.00	2.00	2.00	1
24	2.00	1.00	1.00	2.00	2.00	2.00	1
25	2.00	2.00	1.00	1.00	1.00	2.00	1
26	1.00	2.00	1.00	1.00	1.00	2.00	1
27	2.00	1.00	1.00	2.00	2.00	2.00	1
28	2.00	2.00	2.00	2.00	2.00	1.00	1
29	2.00	1.00	1.00	1.00	2.00	2.00	1
30	2.00	1.00	1.00	1.00	2.00	2.00	1
31	1.00	1.00	1.00	1.00	1.00	2.00	1
32	1.00	1.00	2.00	2.00	1.00	2.00	1
33	2.00	2.00	2.00	2.00	2.00	1.00	1
34	2.00	1.00	2.00	2.00	2.00	2.00	1
35	2.00	1.00	2.00	2.00	2.00	2.00	1
36	2.00	1.00	1.00	1.00	2.00	2.00	1
37	2.00	1.00	2.00	2.00	2.00	2.00	1
38	2.00	2.00	2.00	2.00	2.00	2.00	1
39	2.00	2.00	2.00	2.00	2.00	2.00	1
40	2.00	2.00	2.00	1.00	2.00	2.00	1
41	1.00	1.00	2.00	1.00	1.00	2.00	1
42	2.00	1.00	1.00	1.00	2.00	2.00	1

	SN	Group	Gender	Age	Work	Work2	Work3	Sector
43	47.00	1.00	1.00	36.00	10.00	3.00	2.00	1.00
44	48.00	1.00	1.00	42.00	10.00	3.00	2.00	1.00
45	49.00	1.00	1.00	25.00	8.00	2.00	2.00	1.00
46	51.00	1.00	2.00	37.00	8.00	2.00	2.00	2.00
47	52.00	1.00	2.00	21.00	4.00	1.00	1.00	1.00
48	53.00	1.00	1.00	34.00	10.00	3.00	2.00	1.00
49	54.00	1.00	1.00	37.00	10.00	3.00	2.00	1.00
50	55.00	1.00	2.00	22.00	4.00	1.00	1.00	1.00
51	57.00	1.00	2.00	20.00	2.00	1.00	1.00	1.00
52	64.00	1.00	1.00	25.00	7.00	2.00	2.00	1.00
53	66.00	1.00	1.00	23.00	5.00	1.00	1.00	1.00
54	67.00	1.00	2.00	21.00	9.00	3.00	2.00	1.00
55	69.00	1.00	2.00	24.00	7.00	2.00	2.00	1.00
56	72.00	1.00	2.00	22.00	6.00	1.00	1.00	2.00
57	73.00	1.00	1.00	26.00	8.00	2.00	2.00	1.00
58	75.00	1.00	1.00	19.00	3.00	1.00	1.00	1.00
59	77.00	1.00	1.00	24.00	6.00	1.00	1.00	1.00
60	78.00	1.00	1.00	24.00	6.00	1.00	1.00	1.00
61	79.00	1.00	1.00	24.00	6.00	1.00	1.00	1.00
62	80.00	1.00	1.00	23.00	6.00	1.00	1.00	1.00
63	82.00	1.00	1.00	28.00	8.00	2.00	2.00	2.00
64	84.00	1.00	1.00	20.00	3.00	1.00	1.00	1.00
65	88.00	1.00	1.00	19.00	2.00	1.00	1.00	1.00
66	90.00	1.00	1.00	27.00	5.00	1.00	1.00	1.00
67	91.00	1.00	1.00	22.00	2.00	1.00	1.00	2.00
68	95.00	1.00	2.00	25.00	7.00	2.00	2.00	2.00
69	96.00	1.00	1.00	23.00	5.00	1.00	1.00	1.00
70	98.00	1.00	1.00	23.00	5.00	1.00	1.00	1.00
71	99.00	1.00	2.00	24.00	6.00	1.00	1.00	2.00
72	100.00	1.00	1.00	22.00	5.00	1.00	1.00	1.00
73	104.00	1.00	1.00	21.00	4.00	1.00	1.00	1.00
74	105.00	1.00	2.00	23.00	4.00	1.00	1.00	2.00
75	106.00	1.00	1.00	21.00	4.00	1.00	1.00	1.00
76	108.00	1.00	1.00	23.00	5.00	1.00	1.00	1.00
77	110.00	1.00	1.00	47.00	10.00	3.00	2.00	1.00
78	115.00	1.00	2.00	26.00	8.00	2.00	2.00	2.00
79	116.00	1.00	2.00	22.00	4.00	1.00	1.00	1.00
80	117.00	1.00	2.00	22.00	5.00	1.00	1.00	2.00
81	118.00	1.00	2.00	21.00	4.00	1.00	1.00	1.00
82	120.00	1.00	2.00	26.00	8.00	2.00	2.00	1.00
83	121.00	1.00	1.00	29.00	8.00	2.00	2.00	2.00
84	122.00	1.00	2.00	23.00	6.00	1.00	1.00	1.00

	Region	Nationality	M1	M2	M3	M4	M5	M6
43	1.00	1.00	1.00	1.00	2.00	1.00	2.00	1.00
44	1.00	1.00	1.00	1.00	2.00	2.00	2.00	2.00
45	1.00	1.00	1.00	1.00	2.00	1.00	1.00	1.00
46	1.00	1.00	1.00	1.00	1.00	2.00	2.00	2.00
47	1.00	1.00	1.00	1.00	1.00	1.00	1.00	1.00
48	1.00	1.00	1.00	1.00	1.00	1.00	1.00	1.00
49	1.00	1.00	1.00	1.00	2.00	2.00	1.00	2.00
50	1.00	1.00	1.00	1.00	1.00	1.00	1.00	1.00
51	1.00	2.00	1.00	1.00	1.00	1.00	1.00	1.00
52	1.00	1.00	1.00	1.00	1.00	1.00	1.00	1.00
53	1.00	1.00	1.00	2.00	1.00	1.00	1.00	1.00
54	3.00	1.00	1.00	2.00	1.00	1.00	1.00	1.00
55	1.00	1.00	1.00	2.00	2.00	1.00	1.00	1.00
56	3.00	1.00	1.00	1.00	2.00	2.00	1.00	1.00
57	1.00	1.00	1.00	1.00	1.00	1.00	2.00	1.00
58	1.00	1.00	1.00	1.00	2.00	1.00	1.00	1.00
59	3.00	1.00	1.00	1.00	1.00	1.00	2.00	1.00
60	4.00	1.00	1.00	2.00	1.00	1.00	1.00	2.00
61	1.00	1.00	1.00	1.00	2.00	1.00	2.00	1.00
62	1.00	1.00	1.00	1.00	1.00	1.00	1.00	1.00
63	4.00	1.00	1.00	1.00	1.00	1.00	1.00	1.00
64	4.00	1.00	2.00	2.00	2.00	1.00	1.00	2.00
65	1.00	1.00	1.00	1.00	2.00	1.00	1.00	1.00
66	3.00	1.00	1.00	1.00	1.00	1.00	2.00	1.00
67	4.00	1.00	1.00	1.00	2.00	1.00	1.00	2.00
68	2.00	2.00	1.00	1.00	1.00	1.00	1.00	1.00
69	1.00	1.00	1.00	1.00	1.00	1.00	1.00	1.00
70	1.00	1.00	1.00	1.00	1.00	1.00	1.00	2.00
71	2.00	1.00	1.00	1.00	1.00	1.00	1.00	1.00
72	4.00	1.00	1.00	1.00	2.00	2.00	1.00	2.00
73	1.00	1.00	1.00	1.00	1.00	2.00	1.00	1.00
74	2.00	1.00	1.00	2.00	2.00	1.00	1.00	1.00
75	1.00	1.00	1.00	2.00	1.00	1.00	1.00	2.00
76	1.00	1.00	1.00	1.00	1.00	1.00	1.00	2.00
77	1.00	2.00	1.00	1.00	2.00	1.00	1.00	1.00
78	1.00	1.00	1.00	1.00	2.00	2.00	1.00	1.00
79	1.00	1.00	2.00	1.00	2.00	1.00	1.00	1.00
80	4.00	2.00	1.00	1.00	1.00	1.00	1.00	1.00
81	1.00	1.00	1.00	1.00	1.00	1.00	1.00	1.00
82	2.00	1.00	1.00	1.00	2.00	1.00	1.00	1.00
83	2.00	1.00	1.00	2.00	2.00	1.00	1.00	2.00
84	2.00	1.00	1.00	1.00	2.00	1.00	2.00	2.00

	M7	M8	M9	M10	M11	M12	M13	M14
43	1.00	1.00	1.00	1.00	2.00	1.00	1.00	2.00
44	2.00	1.00	2.00	1.00	2.00	1.00	2.00	2.00
45	1.00	1.00	1.00	1.00	2.00	1.00	1.00	1.00
46	1.00	1.00	1.00	1.00	2.00	1.00	2.00	1.00
47	2.00	1.00	1.00	1.00	2.00	1.00	2.00	1.00
48	1.00	1.00	1.00	1.00	1.00	1.00	1.00	1.00
49	1.00	2.00	2.00	1.00	2.00	2.00	2.00	2.00
50	1.00	1.00	1.00	1.00	2.00	1.00	1.00	1.00
51	1.00	1.00	1.00	1.00	1.00	1.00	1.00	1.00
52	1.00	1.00	1.00	1.00	2.00	1.00	1.00	1.00
53	1.00	1.00	1.00	1.00	2.00	2.00	1.00	1.00
54	1.00	1.00	1.00	1.00	2.00	1.00	1.00	1.00
55	1.00	1.00	2.00	2.00	1.00	1.00	2.00	1.00
56	1.00	2.00	1.00	2.00	1.00	1.00	2.00	1.00
57	1.00	1.00	1.00	1.00	2.00	2.00	2.00	2.00
58	1.00	1.00	1.00	1.00	2.00	1.00	1.00	1.00
59	1.00	1.00	1.00	1.00	1.00	1.00	1.00	1.00
60	1.00	2.00	2.00	2.00	1.00	2.00	2.00	1.00
61	2.00	2.00	2.00	1.00	2.00	2.00	2.00	2.00
62	1.00	1.00	1.00	1.00	1.00	1.00	2.00	1.00
63	1.00	1.00	1.00	1.00	2.00	1.00	1.00	1.00
64	2.00	1.00	2.00	2.00	1.00	1.00	1.00	1.00
65	1.00	1.00	1.00	1.00	2.00	2.00	1.00	1.00
66	1.00	1.00	2.00	1.00	2.00	1.00	1.00	1.00
67	1.00	1.00	1.00	1.00	1.00	1.00	1.00	1.00
68	1.00	1.00	1.00	1.00	2.00	1.00	2.00	2.00
69	2.00	1.00	2.00	1.00	2.00	1.00	1.00	1.00
70	2.00	1.00	2.00	1.00	1.00	2.00	1.00	1.00
71	1.00	1.00	1.00	1.00	1.00	1.00	1.00	1.00
72	1.00	1.00	1.00	2.00	2.00	2.00	1.00	1.00
73	1.00	1.00	1.00	1.00	1.00	1.00	1.00	1.00
74	1.00	1.00	2.00	1.00	1.00	1.00	1.00	1.00
75	2.00	1.00	1.00	2.00	1.00	2.00	1.00	2.00
76	1.00	1.00	2.00	1.00	2.00	1.00	1.00	1.00
77	1.00	1.00	2.00	1.00	2.00	2.00	2.00	2.00
78	1.00	1.00	1.00	1.00	2.00	1.00	2.00	1.00
79	2.00	1.00	2.00	2.00	2.00	1.00	1.00	1.00
80	2.00	1.00	1.00	1.00	1.00	1.00	1.00	1.00
81	1.00	1.00	1.00	1.00	1.00	1.00	1.00	1.00
82	1.00	1.00	1.00	1.00	2.00	2.00	1.00	1.00
83	2.00	2.00	1.00	1.00	2.00	2.00	1.00	1.00
84	2.00	1.00	2.00	1.00	2.00	1.00	2.00	2.00

	M15	B1	B2	B3	B4	B5	B6	B7
43	1.00	1.00	1.00	1.00	1.00	1.00	1.00	1.00
44	1.00	1.00	1.00	2.00	1.00	2.00	1.00	1.00
45	1.00	2.00	1.00	2.00	1.00	1.00	1.00	1.00
46	1.00	1.00	1.00	2.00	2.00	2.00	2.00	2.00
47	2.00	2.00	1.00	2.00	1.00	1.00	1.00	1.00
48	1.00	1.00	1.00	1.00	1.00	1.00	1.00	1.00
49	1.00	1.00	2.00	1.00	1.00	1.00	2.00	2.00
50	1.00	1.00	2.00	2.00	2.00	2.00	1.00	1.00
51	1.00	1.00	1.00	1.00	1.00	1.00	1.00	1.00
52	1.00	1.00	1.00	2.00	2.00	1.00	1.00	1.00
53	1.00	1.00	1.00	1.00	1.00	1.00	1.00	1.00
54	1.00	1.00	1.00	2.00	1.00	1.00	2.00	1.00
55	1.00	1.00	1.00	1.00	2.00	2.00	2.00	2.00
56	2.00	2.00	1.00	2.00	1.00	1.00	2.00	1.00
57	1.00	2.00	2.00	2.00	2.00	2.00	2.00	2.00
58	1.00	2.00	2.00	2.00	1.00	2.00	2.00	2.00
59	2.00	1.00	1.00	2.00	1.00	1.00	1.00	1.00
60	2.00	2.00	1.00	1.00	2.00	1.00	1.00	1.00
61	2.00	2.00	2.00	2.00	1.00	2.00	1.00	2.00
62	1.00	1.00	1.00	1.00	1.00	1.00	1.00	1.00
63	1.00	1.00	1.00	1.00	1.00	1.00	1.00	1.00
64	1.00	1.00	2.00	2.00	2.00	1.00	2.00	2.00
65	1.00	2.00	2.00	2.00	1.00	2.00	2.00	1.00
66	1.00	2.00	1.00	1.00	1.00	2.00	1.00	1.00
67	1.00	2.00	2.00	2.00	1.00	1.00	2.00	2.00
68	1.00	2.00	2.00	2.00	1.00	2.00	2.00	2.00
69	1.00	1.00	1.00	1.00	2.00	2.00	1.00	1.00
70	1.00	1.00	2.00	2.00	1.00	1.00	1.00	1.00
71	1.00	1.00	1.00	1.00	1.00	1.00	1.00	1.00
72	2.00	2.00	2.00	2.00	1.00	1.00	1.00	1.00
73	2.00	1.00	2.00	2.00	1.00	1.00	1.00	1.00
74	1.00	2.00	2.00	2.00	1.00	2.00	1.00	1.00
75	2.00	1.00	1.00	2.00	2.00	1.00	2.00	1.00
76	1.00	1.00	2.00	1.00	1.00	1.00	2.00	1.00
77	2.00	1.00	1.00	2.00	1.00	1.00	2.00	2.00
78	1.00	2.00	1.00	1.00	1.00	2.00	2.00	2.00
79	2.00	2.00	1.00	2.00	1.00	2.00	2.00	2.00
80	1.00	2.00	2.00	2.00	1.00	2.00	2.00	1.00
81	1.00	2.00	2.00	2.00	2.00	2.00	2.00	2.00
82	1.00	2.00	2.00	2.00	1.00	2.00	2.00	2.00
83	2.00	1.00	1.00	1.00	1.00	2.00	2.00	2.00
84	1.00	2.00	1.00	2.00	1.00	2.00	2.00	2.00

	B8	B9	B10	B11	B12	B13	B14	W1
43	2.00	2.00	2.00	2.00	2.00	1.00	1.00	1.00
44	2.00	2.00	2.00	1.00	2.00	2.00	2.00	1.00
45	1.00	1.00	1.00	1.00	2.00	2.00	1.00	1.00
46	2.00	1.00	2.00	1.00	2.00	2.00	2.00	1.00
47	2.00	1.00	1.00	1.00	1.00	2.00	1.00	1.00
48	1.00	1.00	1.00	1.00	1.00	1.00	1.00	1.00
49	2.00	2.00	2.00	1.00	2.00	2.00	1.00	1.00
50	2.00	1.00	1.00	2.00	2.00	2.00	2.00	1.00
51	1.00	1.00	1.00	1.00	1.00	1.00	1.00	1.00
52	2.00	2.00	2.00	1.00	1.00	2.00	2.00	1.00
53	1.00	1.00	1.00	1.00	1.00	1.00	1.00	1.00
54	2.00	1.00	1.00	2.00	1.00	2.00	1.00	2.00
55	2.00	1.00	2.00	1.00	2.00	2.00	2.00	2.00
56	2.00	1.00	1.00	1.00	2.00	2.00	2.00	2.00
57	2.00	2.00	2.00	2.00	2.00	2.00	2.00	1.00
58	2.00	2.00	2.00	1.00	2.00	2.00	1.00	2.00
59	1.00	1.00	1.00	1.00	2.00	1.00	2.00	1.00
60	2.00	2.00	2.00	1.00	1.00	2.00	1.00	1.00
61	2.00	2.00	2.00	1.00	2.00	2.00	2.00	2.00
62	1.00	1.00	1.00	1.00	1.00	1.00	1.00	1.00
63	1.00	1.00	1.00	1.00	1.00	1.00	1.00	2.00
64	2.00	1.00	2.00	2.00	1.00	2.00	2.00	2.00
65	1.00	1.00	2.00	1.00	2.00	2.00	2.00	1.00
66	1.00	2.00	1.00	1.00	2.00	2.00	2.00	1.00
67	2.00	1.00	1.00	1.00	2.00	1.00	1.00	1.00
68	2.00	2.00	2.00	1.00	2.00	2.00	2.00	2.00
69	1.00	1.00	1.00	1.00	2.00	1.00	2.00	1.00
70	2.00	1.00	1.00	2.00	1.00	2.00	2.00	1.00
71	1.00	1.00	1.00	1.00	1.00	1.00	1.00	1.00
72	2.00	2.00	2.00	1.00	2.00	2.00	2.00	2.00
73	1.00	1.00	1.00	2.00	1.00	1.00	2.00	1.00
74	2.00	2.00	1.00	1.00	2.00	2.00	1.00	1.00
75	2.00	1.00	2.00	2.00	2.00	1.00	1.00	2.00
76	1.00	2.00	1.00	1.00	1.00	1.00	1.00	1.00
77	2.00	2.00	1.00	2.00	2.00	2.00	2.00	2.00
78	2.00	2.00	2.00	1.00	2.00	2.00	1.00	1.00
79	2.00	1.00	1.00	1.00	2.00	2.00	1.00	1.00
80	1.00	1.00	2.00	2.00	2.00	2.00	2.00	1.00
81	2.00	2.00	2.00	2.00	2.00	2.00	2.00	2.00
82	1.00	1.00	1.00	1.00	2.00	2.00	1.00	2.00
83	1.00	1.00	1.00	1.00	1.00	2.00	1.00	2.00
84	2.00	1.00	2.00	1.00	2.00	2.00	2.00	1.00

	W2	W3	W4	W5	W6	E1	V1	V2
43	2.00	2.00	2.00	1.00	1.00	1.00	2.00	2.00
44	1.00	2.00	2.00	1.00	1.00	1.00	2.00	2.00
45	2.00	1.00	2.00	1.00	2.00	1.00	2.00	2.00
46	2.00	1.00	1.00	1.00	2.00	1.00	2.00	2.00
47	1.00	2.00	2.00	1.00	2.00	1.00	2.00	2.00
48	1.00	1.00	1.00	1.00	1.00	1.00	1.00	1.00
49	1.00	2.00	2.00	1.00	2.00	1.00	2.00	1.00
50	1.00	1.00	1.00	1.00	1.00	1.00	2.00	2.00
51	1.00	1.00	1.00	1.00	1.00	1.00	1.00	1.00
52	1.00	1.00	2.00	2.00	2.00	1.00	2.00	2.00
53	1.00	2.00	2.00	1.00	2.00	1.00	2.00	2.00
54	2.00	2.00	2.00	1.00	1.00	1.00	1.00	1.00
55	2.00	1.00	2.00	1.00	2.00	1.00	2.00	2.00
56	2.00	2.00	2.00	2.00	2.00	1.00	1.00	1.00
57	1.00	1.00	1.00	2.00	2.00	1.00	2.00	2.00
58	1.00	2.00	2.00	1.00	1.00	1.00	2.00	2.00
59	1.00	1.00	1.00	1.00	1.00	1.00	1.00	2.00
60	1.00	2.00	1.00	1.00	1.00	1.00	1.00	1.00
61	2.00	1.00	1.00	2.00	2.00	1.00	2.00	2.00
62	1.00	1.00	2.00	1.00	2.00	1.00	1.00	2.00
63	2.00	1.00	2.00	2.00	2.00	1.00	2.00	2.00
64	2.00	1.00	1.00	1.00	1.00	1.00	2.00	2.00
65	1.00	1.00	1.00	1.00	2.00	1.00	2.00	2.00
66	1.00	1.00	1.00	1.00	1.00	1.00	2.00	2.00
67	1.00	1.00	1.00	1.00	1.00	1.00	2.00	2.00
68	2.00	1.00	1.00	1.00	2.00	1.00	2.00	2.00
69	2.00	1.00	1.00	1.00	2.00	1.00	1.00	2.00
70	1.00	2.00	1.00	1.00	2.00	1.00	2.00	2.00
71	1.00	1.00	1.00	1.00	1.00	1.00	1.00	1.00
72	2.00	1.00	1.00	1.00	2.00	1.00	1.00	1.00
73	2.00	1.00	1.00	2.00	1.00	1.00	1.00	2.00
74	1.00	1.00	1.00	1.00	2.00	1.00	2.00	2.00
75	1.00	2.00	1.00	1.00	1.00	1.00	1.00	1.00
76	1.00	2.00	2.00	1.00	2.00	1.00	2.00	2.00
77	2.00	1.00	1.00	2.00	1.00	1.00	2.00	2.00
78	2.00	2.00	2.00	1.00	2.00	1.00	2.00	2.00
79	1.00	1.00	1.00	1.00	2.00	1.00	2.00	2.00
80	1.00	1.00	1.00	1.00	2.00	1.00	2.00	2.00
81	2.00	1.00	1.00	1.00	2.00	1.00	2.00	2.00
82	2.00	1.00	1.00	1.00	1.00	1.00	2.00	2.00
83	2.00	2.00	2.00	2.00	2.00	1.00	2.00	2.00
84	1.00	1.00	1.00	1.00	1.00	1.00	2.00	2.00

	V3	V4	V5	V6	E2	E3	E4	C1
43	1.00	2.00	2.00	2.00	1.00	2.00	1.00	2.00
44	1.00	1.00	2.00	2.00	1.00	2.00	1.00	2.00
45	1.00	1.00	1.00	2.00	1.00	2.00	2.00	2.00
46	1.00	1.00	1.00	2.00	1.00	2.00	1.00	2.00
47	2.00	1.00	1.00	1.00	1.00	2.00	1.00	2.00
48	1.00	1.00	1.00	1.00	1.00	1.00	1.00	1.00
49	1.00	1.00	2.00	2.00	1.00	1.00	2.00	2.00
50	1.00	2.00	2.00	2.00	1.00	2.00	2.00	2.00
51	1.00	1.00	1.00	1.00	1.00	2.00	1.00	1.00
52	1.00	2.00	2.00	2.00	1.00	2.00	2.00	2.00
53	2.00	2.00	2.00	2.00	1.00	2.00	1.00	2.00
54	1.00	1.00	1.00	2.00	1.00	2.00	1.00	1.00
55	1.00	2.00	2.00	1.00	1.00	2.00	1.00	2.00
56	1.00	1.00	1.00	1.00	1.00	2.00	1.00	1.00
57	1.00	1.00	2.00	2.00	1.00	1.00	1.00	1.00
58	2.00	1.00	1.00	1.00	1.00	2.00	1.00	2.00
59	1.00	1.00	1.00	1.00	1.00	1.00	2.00	2.00
60	2.00	2.00	1.00	1.00	1.00	2.00	2.00	1.00
61	1.00	1.00	2.00	2.00	1.00	2.00	2.00	2.00
62	1.00	1.00	2.00	2.00	1.00	2.00	1.00	2.00
63	1.00	1.00	1.00	1.00	1.00	2.00	2.00	2.00
64	1.00	2.00	2.00	1.00	1.00	2.00	2.00	2.00
65	2.00	2.00	2.00	1.00	1.00	2.00	2.00	2.00
66	2.00	1.00	1.00	1.00	1.00	2.00	1.00	2.00
67	2.00	2.00	2.00	1.00	1.00	2.00	1.00	2.00
68	1.00	2.00	2.00	2.00	1.00	1.00	1.00	2.00
69	1.00	1.00	1.00	2.00	1.00	2.00	1.00	2.00
70	2.00	2.00	1.00	2.00	1.00	2.00	1.00	2.00
71	1.00	1.00	1.00	1.00	1.00	2.00	2.00	2.00
72	1.00	2.00	2.00	2.00	1.00	2.00	1.00	1.00
73	1.00	1.00	1.00	1.00	1.00	1.00	1.00	2.00
74	2.00	2.00	1.00	1.00	1.00	2.00	2.00	2.00
75	2.00	2.00	1.00	2.00	1.00	2.00	2.00	1.00
76	2.00	1.00	2.00	1.00	1.00	2.00	1.00	2.00
77	2.00	1.00	2.00	1.00	1.00	1.00	2.00	2.00
78	2.00	2.00	2.00	2.00	1.00	2.00	1.00	2.00
79	2.00	2.00	2.00	1.00	1.00	2.00	1.00	2.00
80	2.00	2.00	2.00	1.00	1.00	2.00	1.00	2.00
81	2.00	2.00	2.00	1.00	1.00	2.00	2.00	2.00
82	1.00	1.00	1.00	2.00	1.00	2.00	2.00	2.00
83	2.00	2.00	2.00	2.00	1.00	2.00	2.00	2.00
84	1.00	1.00	2.00	2.00	1.00	2.00	2.00	2.00

	C2	C3	C4	C5	C6	C7	filter_$
43	2.00	1.00	2.00	2.00	2.00	2.00	1
44	2.00	1.00	2.00	1.00	2.00	2.00	1
45	2.00	1.00	1.00	1.00	2.00	2.00	1
46	2.00	1.00	1.00	1.00	2.00	2.00	1
47	2.00	1.00	2.00	2.00	2.00	2.00	1
48	1.00	1.00	1.00	1.00	1.00	2.00	1
49	2.00	1.00	2.00	1.00	2.00	2.00	1
50	2.00	2.00	1.00	2.00	1.00	2.00	1
51	1.00	1.00	1.00	1.00	1.00	2.00	1
52	2.00	1.00	1.00	2.00	2.00	2.00	1
53	2.00	1.00	1.00	1.00	1.00	2.00	1
54	2.00	1.00	2.00	1.00	2.00	2.00	1
55	2.00	1.00	2.00	2.00	2.00	2.00	1
56	1.00	1.00	1.00	1.00	1.00	2.00	1
57	1.00	1.00	1.00	1.00	1.00	2.00	1
58	2.00	1.00	1.00	2.00	2.00	2.00	1
59	1.00	2.00	1.00	1.00	1.00	2.00	1
60	2.00	1.00	1.00	1.00	1.00	2.00	1
61	2.00	1.00	1.00	1.00	2.00	2.00	1
62	2.00	1.00	1.00	2.00	2.00	2.00	1
63	2.00	1.00	1.00	1.00	1.00	2.00	1
64	2.00	1.00	2.00	2.00	2.00	2.00	1
65	2.00	2.00	2.00	2.00	2.00	1.00	1
66	2.00	1.00	2.00	2.00	2.00	2.00	1
67	2.00	1.00	2.00	2.00	2.00	2.00	1
68	2.00	2.00	2.00	2.00	2.00	1.00	1
69	2.00	1.00	1.00	2.00	2.00	2.00	1
70	2.00	1.00	2.00	2.00	2.00	2.00	1
71	2.00	1.00	1.00	1.00	2.00	2.00	1
72	2.00	1.00	2.00	1.00	1.00	2.00	1
73	1.00	1.00	1.00	1.00	2.00	2.00	1
74	2.00	2.00	2.00	1.00	2.00	2.00	1
75	2.00	2.00	1.00	2.00	1.00	2.00	1
76	2.00	1.00	1.00	1.00	1.00	2.00	1
77	2.00	1.00	2.00	2.00	2.00	2.00	1
78	2.00	1.00	1.00	1.00	2.00	2.00	1
79	2.00	1.00	2.00	2.00	2.00	2.00	1
80	2.00	1.00	1.00	1.00	2.00	2.00	1
81	2.00	1.00	2.00	2.00	2.00	2.00	1
82	2.00	1.00	2.00	1.00	2.00	2.00	1
83	2.00	1.00	2.00	2.00	2.00	2.00	1
84	2.00	1.00	2.00	2.00	2.00	2.00	1

	SN	Group	Gender	Age	Work	Work2	Work3	Sector
85	124.00	1.00	2.00	21.00	3.00	1.00	1.00	2.00
86	125.00	1.00	1.00	32.00	9.00	3.00	2.00	1.00
87	126.00	1.00	1.00	24.00	7.00	2.00	2.00	1.00
88	127.00	1.00	1.00	25.00	7.00	2.00	2.00	1.00
89	128.00	1.00	1.00	27.00	8.00	2.00	2.00	2.00
90	129.00	1.00	2.00	25.00	7.00	2.00	2.00	1.00
91	130.00	1.00	2.00	24.00	7.00	2.00	2.00	1.00
92	131.00	1.00	2.00	25.00	6.00	1.00	1.00	1.00
93	133.00	1.00	1.00	27.00	8.00	2.00	2.00	2.00
94	134.00	1.00	1.00	25.00	7.00	2.00	2.00	1.00
95	136.00	1.00	2.00	28.00	8.00	2.00	2.00	1.00
96	137.00	1.00	1.00	25.00	7.00	2.00	2.00	1.00
97	138.00	1.00	1.00	24.00	7.00	2.00	2.00	1.00
98	139.00	1.00	2.00	28.00	8.00	2.00	2.00	1.00
99	141.00	1.00	1.00	24.00	7.00	2.00	2.00	1.00
100	142.00	1.00	1.00	25.00	7.00	2.00	2.00	1.00
101	144.00	1.00	1.00	23.00	6.00	1.00	1.00	1.00
102	146.00	1.00	2.00	27.00	8.00	2.00	2.00	1.00
103	147.00	1.00	1.00	27.00	8.00	2.00	2.00	2.00
104	148.00	1.00	2.00	24.00	8.00	2.00	2.00	2.00
105	149.00	1.00	2.00	25.00	6.00	1.00	1.00	1.00
106	150.00	1.00	1.00	24.00	6.00	1.00	1.00	1.00
107	151.00	1.00	1.00	25.00	6.00	1.00	1.00	1.00
108	153.00	1.00	1.00	23.00	6.00	1.00	1.00	1.00
109	154.00	1.00	1.00	25.00	6.00	1.00	1.00	2.00
110	155.00	1.00	1.00	23.00	5.00	1.00	1.00	2.00
111	158.00	1.00	1.00	25.00	7.00	2.00	2.00	1.00
112	159.00	1.00	1.00	23.00	5.00	1.00	1.00	2.00
113	160.00	1.00	1.00	22.00	5.00	1.00	1.00	2.00
114	161.00	1.00	1.00	24.00	7.00	2.00	2.00	1.00
115	163.00	1.00	1.00	25.00	7.00	2.00	2.00	1.00
116	164.00	1.00	1.00	26.00	8.00	2.00	2.00	1.00
117	165.00	1.00	1.00	27.00	8.00	2.00	2.00	2.00
118	166.00	1.00	1.00	31.00	8.00	2.00	2.00	2.00
119	167.00	1.00	2.00	29.00	8.00	2.00	2.00	1.00
120	168.00	1.00	1.00	26.00	8.00	2.00	2.00	2.00
121	169.00	1.00	2.00	26.00	8.00	2.00	2.00	1.00
122	170.00	1.00	2.00	26.00	8.00	2.00	2.00	2.00
123	171.00	1.00	1.00	23.00	6.00	1.00	1.00	2.00
124	172.00	1.00	1.00	24.00	7.00	2.00	2.00	1.00
125	173.00	1.00	2.00	28.00	9.00	3.00	2.00	1.00
126	174.00	1.00	2.00	25.00	8.00	2.00	2.00	2.00

	Region	Nationality	M1	M2	M3	M4	M5	M6
85	2.00	1.00	1.00	2.00	1.00	2.00	2.00	1.00
86	4.00	1.00	1.00	1.00	2.00	1.00	2.00	2.00
87	3.00	1.00	2.00	1.00	1.00	1.00	1.00	1.00
88	3.00	1.00	1.00	1.00	1.00	1.00	1.00	1.00
89	1.00	1.00	1.00	1.00	2.00	1.00	1.00	2.00
90	3.00	1.00	2.00	2.00	2.00	2.00	2.00	2.00
91	3.00	1.00	2.00	2.00	2.00	1.00	2.00	1.00
92	3.00	1.00	1.00	1.00	1.00	1.00	1.00	2.00
93	1.00	1.00	1.00	1.00	2.00	1.00	1.00	1.00
94	3.00	1.00	1.00	1.00	2.00	1.00	1.00	1.00
95	1.00	2.00	1.00	1.00	1.00	1.00	1.00	1.00
96	1.00	1.00	1.00	2.00	2.00	2.00	1.00	1.00
97	1.00	1.00	2.00	1.00	2.00	2.00	1.00	1.00
98	1.00	1.00	1.00	1.00	1.00	1.00	1.00	1.00
99	1.00	1.00	1.00	2.00	1.00	2.00	2.00	2.00
100	1.00	1.00	2.00	1.00	1.00	2.00	1.00	1.00
101	1.00	1.00	1.00	1.00	2.00	2.00	2.00	1.00
102	1.00	1.00	1.00	1.00	2.00	1.00	1.00	2.00
103	1.00	1.00	1.00	1.00	1.00	1.00	1.00	1.00
104	2.00	1.00	2.00	2.00	2.00	1.00	1.00	1.00
105	3.00	1.00	2.00	1.00	1.00	1.00	1.00	1.00
106	4.00	1.00	1.00	1.00	1.00	1.00	1.00	2.00
107	4.00	1.00	1.00	1.00	1.00	1.00	1.00	1.00
108	4.00	1.00	2.00	2.00	2.00	2.00	1.00	2.00
109	2.00	1.00	2.00	2.00	2.00	1.00	2.00	2.00
110	2.00	2.00	1.00	1.00	2.00	1.00	1.00	1.00
111	3.00	1.00	1.00	2.00	1.00	1.00	1.00	1.00
112	2.00	2.00	1.00	2.00	1.00	1.00	1.00	2.00
113	2.00	2.00	1.00	1.00	2.00	1.00	1.00	2.00
114	4.00	1.00	1.00	1.00	2.00	1.00	1.00	1.00
115	4.00	1.00	1.00	2.00	1.00	2.00	2.00	1.00
116	3.00	1.00	1.00	1.00	1.00	1.00	1.00	1.00
117	3.00	1.00	1.00	1.00	1.00	1.00	1.00	1.00
118	4.00	1.00	1.00	2.00	1.00	1.00	1.00	1.00
119	3.00	1.00	1.00	1.00	2.00	1.00	1.00	2.00
120	1.00	1.00	2.00	2.00	2.00	1.00	1.00	1.00
121	1.00	1.00	1.00	1.00	2.00	1.00	1.00	1.00
122	1.00	1.00	1.00	1.00	1.00	2.00	1.00	1.00
123	2.00	2.00	1.00	2.00	2.00	1.00	1.00	1.00
124	4.00	1.00	1.00	1.00	2.00	1.00	1.00	1.00
125	1.00	1.00	1.00	1.00	1.00	1.00	1.00	1.00
126	1.00	2.00	1.00	1.00	1.00	1.00	1.00	1.00

	M7	M8	M9	M10	M11	M12	M13	M14
85	2.00	2.00	1.00	2.00	2.00	1.00	2.00	2.00
86	1.00	2.00	2.00	1.00	2.00	2.00	2.00	2.00
87	1.00	2.00	1.00	1.00	2.00	1.00	1.00	1.00
88	2.00	1.00	1.00	1.00	1.00	1.00	2.00	1.00
89	1.00	1.00	2.00	1.00	2.00	2.00	2.00	2.00
90	2.00	2.00	2.00	2.00	2.00	1.00	2.00	1.00
91	1.00	1.00	1.00	1.00	1.00	1.00	2.00	1.00
92	1.00	1.00	1.00	1.00	2.00	1.00	1.00	2.00
93	1.00	1.00	1.00	1.00	1.00	2.00	1.00	1.00
94	1.00	2.00	2.00	1.00	1.00	2.00	1.00	1.00
95	1.00	1.00	1.00	1.00	1.00	1.00	1.00	1.00
96	1.00	2.00	2.00	1.00	2.00	1.00	1.00	1.00
97	2.00	1.00	2.00	2.00	2.00	2.00	1.00	1.00
98	1.00	1.00	1.00	1.00	1.00	1.00	1.00	1.00
99	2.00	2.00	1.00	1.00	2.00	2.00	2.00	1.00
100	1.00	1.00	2.00	1.00	2.00	1.00	1.00	1.00
101	2.00	2.00	2.00	1.00	1.00	2.00	1.00	1.00
102	2.00	2.00	2.00	1.00	2.00	1.00	2.00	1.00
103	1.00	1.00	2.00	1.00	1.00	1.00	1.00	1.00
104	1.00	1.00	1.00	1.00	1.00	1.00	1.00	1.00
105	1.00	1.00	1.00	1.00	1.00	1.00	2.00	1.00
106	1.00	1.00	2.00	1.00	1.00	2.00	1.00	1.00
107	1.00	1.00	1.00	1.00	2.00	2.00	2.00	1.00
108	2.00	2.00	2.00	2.00	2.00	1.00	2.00	2.00
109	1.00	1.00	2.00	2.00	1.00	1.00	2.00	1.00
110	1.00	1.00	1.00	1.00	2.00	1.00	1.00	1.00
111	1.00	1.00	1.00	1.00	2.00	2.00	2.00	2.00
112	2.00	1.00	1.00	2.00	2.00	2.00	1.00	1.00
113	2.00	1.00	1.00	1.00	1.00	1.00	1.00	2.00
114	1.00	1.00	1.00	1.00	2.00	1.00	2.00	1.00
115	1.00	1.00	2.00	2.00	1.00	2.00	2.00	1.00
116	1.00	1.00	1.00	1.00	2.00	1.00	2.00	1.00
117	1.00	1.00	1.00	1.00	1.00	1.00	1.00	1.00
118	1.00	1.00	2.00	1.00	1.00	2.00	1.00	1.00
119	1.00	1.00	1.00	1.00	2.00	1.00	1.00	1.00
120	2.00	1.00	2.00	1.00	1.00	1.00	1.00	1.00
121	2.00	1.00	1.00	2.00	2.00	1.00	2.00	1.00
122	1.00	1.00	1.00	1.00	1.00	1.00	1.00	1.00
123	2.00	1.00	1.00	1.00	2.00	1.00	2.00	1.00
124	1.00	1.00	2.00	1.00	2.00	1.00	1.00	1.00
125	1.00	1.00	1.00	1.00	2.00	1.00	2.00	2.00
126	2.00	2.00	1.00	2.00	2.00	2.00	1.00	1.00

	M15	B1	B2	B3	B4	B5	B6	B7
85	2.00	1.00	1.00	2.00	2.00	2.00	2.00	1.00
86	2.00	2.00	1.00	1.00	2.00	1.00	2.00	2.00
87	1.00	1.00	1.00	1.00	1.00	1.00	2.00	2.00
88	1.00	1.00	1.00	2.00	1.00	1.00	2.00	2.00
89	1.00	2.00	1.00	1.00	2.00	2.00	2.00	2.00
90	2.00	1.00	1.00	1.00	2.00	2.00	2.00	2.00
91	1.00	1.00	1.00	2.00	1.00	2.00	1.00	1.00
92	1.00	1.00	2.00	2.00	1.00	2.00	2.00	2.00
93	2.00	2.00	1.00	1.00	1.00	2.00	2.00	2.00
94	1.00	1.00	1.00	1.00	1.00	1.00	2.00	1.00
95	1.00	1.00	2.00	2.00	2.00	1.00	1.00	1.00
96	2.00	1.00	1.00	1.00	1.00	1.00	2.00	2.00
97	1.00	2.00	1.00	1.00	1.00	1.00	1.00	1.00
98	1.00	2.00	2.00	2.00	1.00	2.00	2.00	2.00
99	2.00	2.00	2.00	2.00	1.00	1.00	1.00	1.00
100	1.00	2.00	1.00	1.00	1.00	1.00	1.00	2.00
101	2.00	2.00	1.00	1.00	1.00	2.00	2.00	2.00
102	2.00	2.00	1.00	1.00	1.00	1.00	2.00	2.00
103	2.00	1.00	1.00	1.00	1.00	1.00	2.00	1.00
104	1.00	1.00	2.00	2.00	1.00	1.00	1.00	1.00
105	1.00	2.00	1.00	1.00	2.00	2.00	2.00	2.00
106	1.00	2.00	1.00	2.00	2.00	1.00	2.00	1.00
107	1.00	1.00	1.00	1.00	2.00	2.00	1.00	1.00
108	2.00	1.00	2.00	1.00	1.00	1.00	2.00	2.00
109	2.00	2.00	1.00	2.00	1.00	1.00	1.00	1.00
110	1.00	2.00	2.00	2.00	1.00	2.00	2.00	2.00
111	1.00	2.00	1.00	1.00	1.00	2.00	2.00	2.00
112	1.00	1.00	1.00	1.00	1.00	1.00	1.00	1.00
113	2.00	1.00	2.00	1.00	1.00	1.00	2.00	2.00
114	1.00	2.00	1.00	2.00	2.00	2.00	2.00	2.00
115	2.00	2.00	1.00	1.00	2.00	2.00	2.00	2.00
116	1.00	2.00	2.00	2.00	2.00	2.00	2.00	2.00
117	1.00	2.00	2.00	2.00	2.00	2.00	2.00	2.00
118	1.00	1.00	1.00	1.00	2.00	1.00	2.00	2.00
119	1.00	2.00	2.00	2.00	2.00	2.00	2.00	2.00
120	1.00	1.00	1.00	1.00	1.00	1.00	1.00	1.00
121	1.00	1.00	2.00	2.00	2.00	2.00	2.00	2.00
122	1.00	2.00	1.00	1.00	1.00	1.00	1.00	1.00
123	1.00	1.00	2.00	2.00	1.00	2.00	1.00	1.00
124	1.00	2.00	2.00	2.00	2.00	1.00	2.00	2.00
125	1.00	2.00	1.00	1.00	1.00	2.00	1.00	1.00
126	1.00	1.00	1.00	2.00	2.00	2.00	2.00	1.00

	B8	B9	B10	B11	B12	B13	B14	W1
85	1.00	1.00	2.00	2.00	2.00	2.00	2.00	1.00
86	1.00	1.00	1.00	1.00	2.00	2.00	1.00	1.00
87	2.00	2.00	2.00	1.00	1.00	2.00	1.00	1.00
88	2.00	2.00	2.00	2.00	2.00	2.00	2.00	1.00
89	2.00	2.00	1.00	1.00	2.00	2.00	1.00	1.00
90	2.00	1.00	2.00	1.00	1.00	2.00	2.00	2.00
91	1.00	2.00	1.00	1.00	2.00	2.00	1.00	1.00
92	1.00	2.00	2.00	1.00	2.00	2.00	2.00	1.00
93	2.00	1.00	1.00	2.00	1.00	2.00	1.00	1.00
94	1.00	2.00	1.00	1.00	2.00	2.00	2.00	1.00
95	1.00	1.00	1.00	1.00	1.00	1.00	1.00	1.00
96	1.00	1.00	1.00	1.00	2.00	2.00	2.00	1.00
97	1.00	1.00	1.00	1.00	2.00	2.00	1.00	1.00
98	2.00	2.00	2.00	2.00	2.00	2.00	2.00	2.00
99	1.00	1.00	1.00	2.00	1.00	2.00	1.00	1.00
100	2.00	2.00	2.00	2.00	2.00	2.00	2.00	2.00
101	2.00	2.00	2.00	2.00	1.00	2.00	1.00	2.00
102	2.00	1.00	2.00	1.00	1.00	1.00	2.00	1.00
103	1.00	1.00	1.00	1.00	2.00	1.00	1.00	1.00
104	1.00	1.00	1.00	1.00	1.00	1.00	1.00	1.00
105	1.00	1.00	1.00	1.00	1.00	2.00	1.00	1.00
106	2.00	2.00	2.00	2.00	2.00	2.00	2.00	1.00
107	1.00	1.00	1.00	1.00	2.00	2.00	1.00	1.00
108	2.00	2.00	1.00	2.00	2.00	2.00	1.00	2.00
109	1.00	2.00	2.00	1.00	2.00	2.00	2.00	2.00
110	2.00	2.00	2.00	2.00	2.00	2.00	2.00	1.00
111	2.00	2.00	1.00	2.00	2.00	2.00	2.00	2.00
112	1.00	1.00	1.00	1.00	1.00	1.00	1.00	1.00
113	2.00	2.00	2.00	2.00	2.00	2.00	2.00	2.00
114	2.00	2.00	2.00	1.00	2.00	2.00	2.00	1.00
115	2.00	2.00	2.00	2.00	2.00	2.00	2.00	1.00
116	2.00	2.00	2.00	2.00	2.00	2.00	2.00	1.00
117	2.00	2.00	2.00	2.00	2.00	2.00	2.00	1.00
118	1.00	2.00	1.00	1.00	2.00	2.00	2.00	1.00
119	2.00	2.00	1.00	2.00	2.00	2.00	2.00	1.00
120	2.00	2.00	2.00	2.00	2.00	2.00	2.00	2.00
121	2.00	2.00	2.00	2.00	2.00	2.00	2.00	1.00
122	1.00	1.00	1.00	1.00	2.00	2.00	2.00	1.00
123	1.00	2.00	2.00	1.00	2.00	2.00	2.00	2.00
124	2.00	2.00	1.00	1.00	2.00	2.00	2.00	1.00
125	1.00	2.00	1.00	1.00	2.00	2.00	1.00	1.00
126	2.00	1.00	1.00	2.00	2.00	2.00	2.00	2.00

	W2	W3	W4	W5	W6	E1	V1	V2
85	2.00	1.00	2.00	1.00	2.00	1.00	1.00	2.00
86	1.00	2.00	2.00	2.00	2.00	1.00	2.00	2.00
87	1.00	2.00	2.00	1.00	1.00	1.00	2.00	2.00
88	1.00	1.00	2.00	2.00	2.00	1.00	2.00	2.00
89	1.00	2.00	2.00	2.00	2.00	1.00	2.00	2.00
90	2.00	1.00	2.00	1.00	2.00	1.00	2.00	2.00
91	1.00	2.00	2.00	1.00	2.00	1.00	2.00	2.00
92	1.00	1.00	2.00	2.00	2.00	1.00	1.00	2.00
93	1.00	1.00	1.00	2.00	1.00	1.00	1.00	2.00
94	1.00	2.00	2.00	2.00	2.00	1.00	2.00	2.00
95	1.00	2.00	1.00	1.00	2.00	1.00	2.00	2.00
96	2.00	1.00	2.00	2.00	2.00	1.00	2.00	2.00
97	2.00	1.00	2.00	2.00	2.00	1.00	2.00	2.00
98	2.00	1.00	2.00	2.00	2.00	1.00	2.00	2.00
99	2.00	1.00	2.00	1.00	1.00	1.00	1.00	2.00
100	2.00	1.00	1.00	1.00	1.00	1.00	2.00	1.00
101	2.00	1.00	1.00	1.00	1.00	1.00	2.00	2.00
102	1.00	1.00	2.00	2.00	2.00	1.00	2.00	2.00
103	1.00	1.00	1.00	1.00	1.00	1.00	2.00	2.00
104	1.00	1.00	2.00	1.00	1.00	1.00	2.00	2.00
105	2.00	1.00	2.00	1.00	2.00	1.00	2.00	2.00
106	1.00	1.00	1.00	2.00	2.00	1.00	1.00	1.00
107	1.00	1.00	2.00	1.00	1.00	1.00	2.00	2.00
108	2.00	1.00	2.00	1.00	2.00	1.00	2.00	2.00
109	2.00	2.00	2.00	1.00	2.00	1.00	2.00	2.00
110	1.00	1.00	1.00	1.00	1.00	1.00	2.00	2.00
111	2.00	1.00	2.00	2.00	2.00	1.00	2.00	2.00
112	1.00	1.00	1.00	2.00	2.00	1.00	1.00	1.00
113	1.00	1.00	2.00	2.00	1.00	1.00	2.00	2.00
114	1.00	1.00	2.00	2.00	2.00	1.00	2.00	2.00
115	2.00	1.00	2.00	1.00	2.00	1.00	2.00	2.00
116	2.00	1.00	2.00	1.00	1.00	1.00	2.00	2.00
117	1.00	1.00	1.00	1.00	1.00	1.00	1.00	1.00
118	1.00	2.00	1.00	1.00	1.00	1.00	1.00	1.00
119	2.00	1.00	2.00	2.00	2.00	1.00	2.00	2.00
120	2.00	1.00	2.00	2.00	2.00	1.00	2.00	2.00
121	1.00	1.00	1.00	1.00	2.00	1.00	1.00	1.00
122	1.00	2.00	1.00	1.00	2.00	1.00	2.00	2.00
123	2.00	1.00	1.00	1.00	2.00	1.00	2.00	2.00
124	2.00	1.00	2.00	1.00	2.00	1.00	2.00	2.00
125	2.00	1.00	2.00	1.00	2.00	1.00	2.00	2.00
126	2.00	2.00	2.00	1.00	1.00	1.00	1.00	2.00

	V3	V4	V5	V6	E2	E3	E4	C1
85	1.00	2.00	1.00	2.00	1.00	2.00	1.00	1.00
86	1.00	1.00	2.00	1.00	1.00	2.00	2.00	1.00
87	2.00	2.00	2.00	2.00	1.00	2.00	1.00	2.00
88	2.00	2.00	2.00	1.00	1.00	2.00	2.00	2.00
89	1.00	1.00	2.00	2.00	1.00	2.00	2.00	2.00
90	2.00	2.00	2.00	1.00	1.00	2.00	2.00	2.00
91	1.00	1.00	1.00	2.00	1.00	2.00	1.00	2.00
92	1.00	2.00	2.00	2.00	1.00	2.00	1.00	2.00
93	2.00	1.00	1.00	1.00	1.00	2.00	1.00	1.00
94	1.00	2.00	2.00	2.00	1.00	2.00	2.00	2.00
95	1.00	2.00	1.00	1.00	1.00	2.00	2.00	2.00
96	1.00	2.00	2.00	2.00	1.00	2.00	2.00	2.00
97	1.00	2.00	2.00	2.00	1.00	2.00	2.00	2.00
98	1.00	1.00	1.00	2.00	1.00	2.00	2.00	2.00
99	2.00	1.00	1.00	2.00	1.00	2.00	2.00	2.00
100	1.00	1.00	1.00	1.00	1.00	2.00	1.00	1.00
101	2.00	2.00	2.00	1.00	1.00	2.00	1.00	2.00
102	2.00	2.00	2.00	2.00	1.00	2.00	2.00	2.00
103	2.00	2.00	2.00	2.00	1.00	2.00	2.00	2.00
104	2.00	1.00	2.00	1.00	1.00	2.00	2.00	2.00
105	1.00	2.00	2.00	2.00	1.00	2.00	2.00	2.00
106	1.00	1.00	2.00	2.00	1.00	2.00	1.00	2.00
107	2.00	2.00	2.00	1.00	1.00	2.00	1.00	2.00
108	1.00	2.00	2.00	2.00	1.00	2.00	2.00	2.00
109	2.00	2.00	1.00	1.00	1.00	2.00	2.00	2.00
110	2.00	1.00	1.00	1.00	1.00	2.00	2.00	2.00
111	2.00	2.00	2.00	2.00	1.00	2.00	2.00	2.00
112	1.00	1.00	2.00	2.00	1.00	1.00	1.00	2.00
113	2.00	2.00	1.00	1.00	1.00	1.00	1.00	2.00
114	1.00	2.00	1.00	2.00	1.00	2.00	2.00	2.00
115	2.00	2.00	1.00	1.00	1.00	2.00	1.00	2.00
116	2.00	2.00	1.00	1.00	1.00	2.00	1.00	2.00
117	1.00	1.00	1.00	1.00	1.00	2.00	1.00	2.00
118	1.00	1.00	1.00	2.00	1.00	2.00	2.00	2.00
119	1.00	2.00	2.00	2.00	1.00	2.00	2.00	2.00
120	1.00	1.00	2.00	2.00	1.00	2.00	2.00	1.00
121	1.00	1.00	1.00	2.00	1.00	2.00	1.00	2.00
122	2.00	2.00	1.00	1.00	1.00	2.00	1.00	2.00
123	1.00	1.00	2.00	1.00	1.00	2.00	2.00	2.00
124	2.00	2.00	2.00	1.00	1.00	2.00	2.00	2.00
125	2.00	2.00	2.00	1.00	1.00	2.00	2.00	2.00
126	2.00	2.00	2.00	2.00	1.00	2.00	2.00	2.00

	C2	C3	C4	C5	C6	C7	filter_$
85	2.00	2.00	1.00	2.00	2.00	1.00	1
86	1.00	1.00	2.00	2.00	2.00	2.00	1
87	2.00	1.00	2.00	2.00	2.00	2.00	1
88	2.00	1.00	1.00	1.00	2.00	2.00	1
89	2.00	1.00	1.00	2.00	2.00	2.00	1
90	2.00	1.00	2.00	2.00	2.00	2.00	1
91	2.00	1.00	1.00	2.00	2.00	2.00	1
92	2.00	1.00	2.00	2.00	2.00	2.00	1
93	2.00	1.00	2.00	2.00	2.00	2.00	1
94	2.00	1.00	1.00	2.00	2.00	2.00	1
95	2.00	1.00	1.00	1.00	1.00	2.00	1
96	2.00	1.00	1.00	2.00	2.00	2.00	1
97	2.00	1.00	2.00	2.00	2.00	2.00	1
98	2.00	1.00	2.00	1.00	2.00	2.00	1
99	2.00	1.00	2.00	2.00	2.00	2.00	1
100	1.00	1.00	2.00	2.00	2.00	2.00	1
101	2.00	2.00	2.00	2.00	2.00	2.00	1
102	2.00	1.00	2.00	2.00	2.00	2.00	1
103	2.00	1.00	2.00	1.00	2.00	2.00	1
104	2.00	1.00	1.00	2.00	2.00	2.00	1
105	2.00	2.00	2.00	2.00	2.00	1.00	1
106	2.00	1.00	2.00	2.00	2.00	2.00	1
107	2.00	1.00	2.00	1.00	2.00	2.00	1
108	2.00	1.00	2.00	2.00	2.00	2.00	1
109	2.00	1.00	2.00	1.00	2.00	2.00	1
110	2.00	1.00	1.00	2.00	2.00	2.00	1
111	2.00	2.00	2.00	2.00	2.00	1.00	1
112	2.00	1.00	1.00	2.00	2.00	2.00	1
113	2.00	1.00	2.00	1.00	2.00	2.00	1
114	2.00	1.00	2.00	1.00	2.00	2.00	1
115	2.00	1.00	1.00	2.00	2.00	2.00	1
116	2.00	1.00	2.00	1.00	2.00	2.00	1
117	2.00	2.00	2.00	2.00	2.00	2.00	1
118	2.00	1.00	2.00	2.00	2.00	2.00	1
119	2.00	1.00	1.00	1.00	1.00	2.00	1
120	2.00	1.00	1.00	2.00	2.00	2.00	1
121	2.00	1.00	1.00	2.00	2.00	2.00	1
122	2.00	1.00	1.00	1.00	1.00	2.00	1
123	2.00	1.00	2.00	1.00	2.00	2.00	1
124	2.00	1.00	2.00	1.00	2.00	2.00	1
125	2.00	1.00	2.00	2.00	2.00	2.00	1
126	2.00	2.00	2.00	2.00	2.00	1.00	1

	SN	Group	Gender	Age	Work	Work2	Work3	Sector
127	175.00	1.00	2.00	27.00	8.00	2.00	2.00	2.00
128	176.00	1.00	2.00	27.00	8.00	2.00	2.00	2.00
129	177.00	1.00	2.00	27.00	8.00	2.00	2.00	2.00
130	179.00	1.00	1.00	23.00	5.00	1.00	1.00	1.00
131	180.00	1.00	1.00	25.00	7.00	2.00	2.00	1.00
132	181.00	1.00	2.00	27.00	8.00	2.00	2.00	2.00
133	182.00	1.00	1.00	23.00	6.00	1.00	1.00	2.00
134	183.00	1.00	1.00	24.00	7.00	2.00	2.00	1.00
135	184.00	1.00	1.00	24.00	7.00	2.00	2.00	1.00
136	185.00	1.00	2.00	23.00	6.00	1.00	1.00	1.00
137	186.00	1.00	1.00	25.00	7.00	2.00	2.00	1.00
138	187.00	1.00	1.00	26.00	8.00	2.00	2.00	2.00
139	188.00	1.00	1.00	25.00	7.00	2.00	2.00	1.00
140	189.00	1.00	1.00	24.00	6.00	1.00	1.00	2.00
141	190.00	1.00	1.00	24.00	7.00	2.00	2.00	1.00
142	191.00	1.00	1.00	25.00	7.00	2.00	2.00	1.00
143	192.00	1.00	1.00	26.00	6.00	1.00	1.00	1.00
144	193.00	1.00	1.00	25.00	7.00	2.00	2.00	1.00
145	195.00	1.00	1.00	27.00	8.00	2.00	2.00	2.00
146	196.00	1.00	1.00	26.00	7.00	2.00	2.00	1.00
147	197.00	1.00	2.00	28.00	8.00	2.00	2.00	2.00
148	198.00	1.00	1.00	26.00	7.00	2.00	2.00	1.00
149	200.00	1.00	1.00	24.00	7.00	2.00	2.00	1.00
150	202.00	1.00	2.00	28.00	8.00	2.00	2.00	1.00
151	203.00	1.00	2.00	28.00	8.00	2.00	2.00	2.00
152	204.00	1.00	1.00	28.00	8.00	2.00	2.00	1.00
153	205.00	1.00	1.00	23.00	5.00	1.00	1.00	1.00
154	206.00	1.00	2.00	25.00	7.00	2.00	2.00	1.00
155	207.00	1.00	1.00	23.00	7.00	2.00	2.00	1.00
156	208.00	1.00	2.00	27.00	8.00	2.00	2.00	2.00
157	209.00	1.00	1.00	24.00	6.00	1.00	1.00	1.00
158	210.00	1.00	1.00	26.00	6.00	1.00	1.00	2.00
159	211.00	1.00	1.00	23.00	6.00	1.00	1.00	1.00
160	212.00	1.00	1.00	25.00	6.00	1.00	1.00	1.00
161	214.00	1.00	1.00	26.00	8.00	2.00	2.00	2.00
162	215.00	1.00	1.00	24.00	7.00	2.00	2.00	1.00
163	216.00	1.00	2.00	19.00	2.00	1.00	1.00	1.00
164	217.00	1.00	2.00	23.00	3.00	1.00	1.00	1.00
165	219.00	1.00	1.00	21.00	3.00	1.00	1.00	1.00
166	220.00	1.00	2.00	21.00	3.00	1.00	1.00	1.00
167	221.00	1.00	2.00	22.00	3.00	1.00	1.00	1.00
168	222.00	1.00	2.00	21.00	3.00	1.00	1.00	1.00

	Region	Nationality	M1	M2	M3	M4	M5	M6
127	1.00	1.00	1.00	1.00	1.00	2.00	1.00	1.00
128	2.00	2.00	1.00	1.00	1.00	1.00	1.00	1.00
129	1.00	2.00	1.00	1.00	1.00	2.00	1.00	1.00
130	3.00	1.00	1.00	1.00	2.00	2.00	2.00	2.00
131	4.00	1.00	1.00	1.00	1.00	1.00	1.00	1.00
132	1.00	1.00	1.00	1.00	1.00	1.00	1.00	1.00
133	2.00	1.00	1.00	1.00	2.00	1.00	1.00	2.00
134	4.00	1.00	1.00	1.00	1.00	1.00	1.00	1.00
135	4.00	1.00	1.00	1.00	2.00	1.00	1.00	1.00
136	3.00	1.00	1.00	1.00	2.00	1.00	1.00	1.00
137	1.00	1.00	2.00	2.00	2.00	2.00	2.00	1.00
138	2.00	1.00	1.00	1.00	1.00	1.00	1.00	1.00
139	1.00	1.00	1.00	1.00	2.00	1.00	1.00	1.00
140	4.00	2.00	1.00	2.00	1.00	1.00	1.00	1.00
141	3.00	1.00	1.00	1.00	1.00	2.00	1.00	1.00
142	3.00	1.00	2.00	2.00	2.00	2.00	2.00	2.00
143	3.00	1.00	1.00	2.00	2.00	1.00	1.00	1.00
144	3.00	1.00	2.00	2.00	2.00	2.00	2.00	2.00
145	4.00	1.00	1.00	1.00	1.00	1.00	2.00	1.00
146	3.00	1.00	1.00	1.00	2.00	1.00	1.00	2.00
147	4.00	1.00	1.00	1.00	1.00	1.00	1.00	1.00
148	3.00	1.00	1.00	1.00	2.00	1.00	1.00	1.00
149	3.00	1.00	1.00	1.00	1.00	2.00	2.00	1.00
150	1.00	1.00	2.00	1.00	2.00	1.00	1.00	1.00
151	1.00	1.00	1.00	1.00	1.00	1.00	1.00	1.00
152	5.00	1.00	1.00	1.00	1.00	1.00	1.00	1.00
153	1.00	1.00	2.00	2.00	2.00	1.00	1.00	1.00
154	4.00	1.00	1.00	1.00	1.00	1.00	1.00	2.00
155	1.00	1.00	2.00	1.00	1.00	2.00	1.00	1.00
156	1.00	1.00	2.00	2.00	2.00	1.00	1.00	1.00
157	1.00	1.00	1.00	2.00	2.00	2.00	2.00	1.00
158	2.00	1.00	2.00	1.00	2.00	1.00	2.00	1.00
159	1.00	1.00	2.00	2.00	1.00	1.00	1.00	2.00
160	1.00	1.00	1.00	1.00	1.00	1.00	1.00	2.00
161	1.00	1.00	1.00	1.00	2.00	1.00	1.00	1.00
162	1.00	1.00	1.00	1.00	1.00	1.00	1.00	1.00
163	2.00	1.00	1.00	1.00	1.00	1.00	1.00	1.00
164	2.00	1.00	1.00	1.00	1.00	1.00	1.00	1.00
165	2.00	1.00	1.00	1.00	2.00	1.00	1.00	2.00
166	2.00	1.00	1.00	1.00	1.00	1.00	1.00	1.00
167	2.00	1.00	1.00	1.00	1.00	1.00	2.00	2.00
168	2.00	1.00	1.00	1.00	2.00	1.00	1.00	1.00

	M7	M8	M9	M10	M11	M12	M13	M14
127	1.00	1.00	1.00	1.00	1.00	1.00	1.00	1.00
128	1.00	1.00	1.00	1.00	1.00	1.00	1.00	1.00
129	1.00	1.00	1.00	1.00	2.00	1.00	1.00	1.00
130	2.00	1.00	2.00	2.00	1.00	1.00	2.00	1.00
131	1.00	1.00	2.00	1.00	2.00	1.00	2.00	1.00
132	1.00	1.00	2.00	1.00	2.00	1.00	1.00	1.00
133	1.00	1.00	2.00	1.00	2.00	1.00	1.00	1.00
134	1.00	1.00	1.00	1.00	1.00	1.00	1.00	1.00
135	1.00	1.00	1.00	1.00	1.00	2.00	1.00	1.00
136	2.00	1.00	1.00	1.00	1.00	1.00	2.00	1.00
137	2.00	2.00	2.00	1.00	2.00	1.00	2.00	1.00
138	1.00	1.00	1.00	1.00	2.00	1.00	2.00	2.00
139	1.00	1.00	1.00	1.00	1.00	1.00	2.00	1.00
140	1.00	2.00	2.00	1.00	1.00	2.00	2.00	2.00
141	1.00	1.00	2.00	1.00	2.00	1.00	2.00	1.00
142	2.00	2.00	2.00	2.00	2.00	2.00	2.00	1.00
143	1.00	1.00	2.00	2.00	1.00	1.00	1.00	1.00
144	2.00	2.00	2.00	2.00	2.00	2.00	2.00	2.00
145	2.00	1.00	1.00	2.00	2.00	2.00	2.00	1.00
146	2.00	1.00	2.00	2.00	1.00	2.00	1.00	1.00
147	1.00	1.00	1.00	1.00	1.00	1.00	1.00	1.00
148	1.00	1.00	1.00	1.00	2.00	1.00	1.00	1.00
149	1.00	1.00	1.00	1.00	1.00	2.00	1.00	1.00
150	2.00	1.00	1.00	1.00	1.00	1.00	1.00	1.00
151	2.00	2.00	1.00	1.00	2.00	1.00	2.00	1.00
152	1.00	1.00	1.00	1.00	1.00	1.00	1.00	1.00
153	2.00	1.00	1.00	1.00	1.00	1.00	1.00	1.00
154	2.00	2.00	2.00	1.00	2.00	1.00	2.00	1.00
155	2.00	1.00	1.00	1.00	2.00	1.00	1.00	1.00
156	2.00	1.00	2.00	2.00	1.00	2.00	1.00	1.00
157	2.00	1.00	1.00	1.00	2.00	2.00	2.00	1.00
158	2.00	1.00	2.00	1.00	2.00	1.00	2.00	1.00
159	2.00	1.00	2.00	2.00	2.00	1.00	2.00	1.00
160	2.00	1.00	1.00	1.00	2.00	1.00	1.00	1.00
161	1.00	1.00	1.00	1.00	1.00	1.00	2.00	1.00
162	1.00	1.00	1.00	1.00	1.00	1.00	1.00	1.00
163	2.00	1.00	1.00	1.00	2.00	1.00	1.00	1.00
164	1.00	1.00	1.00	1.00	1.00	1.00	1.00	1.00
165	2.00	1.00	2.00	1.00	1.00	1.00	1.00	1.00
166	1.00	1.00	1.00	1.00	2.00	1.00	1.00	1.00
167	1.00	1.00	1.00	1.00	2.00	1.00	1.00	1.00
168	1.00	1.00	1.00	1.00	1.00	1.00	1.00	1.00

	M15	B1	B2	B3	B4	B5	B6	B7
127	2.00	2.00	1.00	1.00	1.00	1.00	1.00	1.00
128	1.00	2.00	2.00	2.00	2.00	2.00	2.00	2.00
129	1.00	2.00	2.00	2.00	2.00	2.00	2.00	2.00
130	1.00	2.00	2.00	2.00	1.00	1.00	2.00	2.00
131	1.00	2.00	2.00	1.00	2.00	2.00	2.00	2.00
132	1.00	2.00	2.00	2.00	1.00	1.00	1.00	1.00
133	2.00	2.00	1.00	2.00	1.00	1.00	2.00	2.00
134	1.00	1.00	1.00	1.00	1.00	1.00	1.00	1.00
135	1.00	2.00	1.00	1.00	1.00	2.00	1.00	1.00
136	1.00	2.00	2.00	2.00	1.00	2.00	2.00	2.00
137	2.00	2.00	1.00	2.00	1.00	1.00	2.00	2.00
138	1.00	1.00	1.00	1.00	2.00	2.00	1.00	1.00
139	1.00	1.00	1.00	1.00	1.00	2.00	2.00	2.00
140	2.00	2.00	2.00	2.00	1.00	2.00	2.00	2.00
141	1.00	2.00	1.00	2.00	1.00	1.00	2.00	2.00
142	2.00	2.00	2.00	1.00	1.00	1.00	2.00	2.00
143	1.00	1.00	1.00	2.00	1.00	2.00	1.00	1.00
144	2.00	2.00	2.00	2.00	2.00	2.00	2.00	2.00
145	2.00	2.00	1.00	2.00	2.00	2.00	2.00	2.00
146	1.00	1.00	1.00	1.00	1.00	1.00	1.00	1.00
147	1.00	1.00	1.00	1.00	1.00	1.00	1.00	1.00
148	1.00	1.00	1.00	2.00	1.00	2.00	1.00	1.00
149	1.00	2.00	2.00	2.00	1.00	1.00	1.00	1.00
150	1.00	2.00	2.00	2.00	1.00	1.00	1.00	1.00
151	1.00	2.00	2.00	1.00	2.00	2.00	2.00	2.00
152	1.00	1.00	1.00	1.00	1.00	1.00	1.00	1.00
153	2.00	1.00	1.00	1.00	1.00	2.00	2.00	2.00
154	1.00	1.00	1.00	2.00	2.00	1.00	2.00	2.00
155	2.00	1.00	1.00	1.00	1.00	1.00	1.00	1.00
156	2.00	1.00	1.00	1.00	1.00	1.00	1.00	1.00
157	2.00	2.00	1.00	1.00	2.00	2.00	2.00	2.00
158	2.00	1.00	2.00	1.00	2.00	1.00	2.00	1.00
159	2.00	2.00	1.00	1.00	1.00	1.00	2.00	2.00
160	1.00	2.00	2.00	2.00	1.00	2.00	2.00	2.00
161	1.00	1.00	1.00	2.00	1.00	1.00	1.00	1.00
162	1.00	1.00	1.00	1.00	1.00	1.00	1.00	1.00
163	1.00	2.00	1.00	2.00	1.00	2.00	1.00	1.00
164	1.00	2.00	2.00	2.00	1.00	1.00	1.00	1.00
165	1.00	1.00	1.00	1.00	1.00	1.00	1.00	1.00
166	1.00	2.00	2.00	2.00	1.00	2.00	2.00	2.00
167	1.00	2.00	2.00	2.00	1.00	2.00	2.00	2.00
168	2.00	1.00	1.00	2.00	1.00	2.00	2.00	2.00

	B8	B9	B10	B11	B12	B13	B14	W1
127	1.00	1.00	1.00	1.00	1.00	2.00	2.00	2.00
128	2.00	2.00	2.00	2.00	2.00	2.00	2.00	1.00
129	2.00	2.00	2.00	2.00	2.00	2.00	2.00	1.00
130	2.00	2.00	2.00	2.00	2.00	2.00	2.00	1.00
131	2.00	2.00	1.00	1.00	2.00	2.00	1.00	1.00
132	1.00	2.00	1.00	1.00	1.00	1.00	2.00	2.00
133	2.00	2.00	2.00	1.00	2.00	2.00	1.00	1.00
134	1.00	1.00	1.00	1.00	1.00	1.00	1.00	1.00
135	1.00	1.00	1.00	1.00	2.00	2.00	2.00	1.00
136	2.00	1.00	1.00	1.00	2.00	1.00	2.00	2.00
137	2.00	2.00	2.00	2.00	2.00	2.00	2.00	1.00
138	1.00	2.00	1.00	2.00	1.00	2.00	1.00	2.00
139	2.00	2.00	2.00	2.00	2.00	2.00	2.00	1.00
140	2.00	2.00	1.00	1.00	2.00	2.00	1.00	1.00
141	2.00	2.00	2.00	1.00	2.00	2.00	2.00	2.00
142	2.00	2.00	1.00	1.00	2.00	2.00	2.00	2.00
143	1.00	1.00	1.00	1.00	2.00	2.00	2.00	1.00
144	2.00	2.00	2.00	2.00	2.00	2.00	2.00	2.00
145	1.00	2.00	2.00	1.00	2.00	2.00	2.00	2.00
146	1.00	1.00	2.00	1.00	2.00	2.00	1.00	1.00
147	1.00	1.00	1.00	1.00	1.00	1.00	1.00	2.00
148	1.00	1.00	1.00	1.00	2.00	2.00	2.00	1.00
149	1.00	1.00	2.00	2.00	1.00	1.00	1.00	1.00
150	1.00	1.00	1.00	2.00	2.00	1.00	1.00	2.00
151	1.00	2.00	2.00	1.00	2.00	2.00	1.00	1.00
152	1.00	1.00	1.00	1.00	1.00	1.00	1.00	1.00
153	2.00	2.00	2.00	1.00	1.00	2.00	1.00	1.00
154	2.00	2.00	2.00	2.00	1.00	1.00	1.00	2.00
155	2.00	2.00	1.00	1.00	1.00	2.00	1.00	1.00
156	1.00	2.00	2.00	1.00	2.00	1.00	1.00	1.00
157	2.00	2.00	2.00	2.00	2.00	2.00	2.00	1.00
158	2.00	1.00	2.00	1.00	2.00	1.00	2.00	2.00
159	1.00	2.00	1.00	1.00	2.00	2.00	2.00	1.00
160	2.00	2.00	1.00	2.00	2.00	2.00	2.00	1.00
161	1.00	1.00	1.00	1.00	2.00	2.00	1.00	1.00
162	1.00	1.00	1.00	1.00	1.00	1.00	1.00	1.00
163	2.00	2.00	1.00	1.00	1.00	2.00	2.00	1.00
164	2.00	1.00	1.00	1.00	2.00	1.00	2.00	1.00
165	1.00	1.00	1.00	1.00	1.00	1.00	1.00	1.00
166	2.00	2.00	2.00	1.00	2.00	2.00	2.00	1.00
167	2.00	2.00	2.00	1.00	2.00	2.00	2.00	1.00
168	2.00	1.00	1.00	1.00	2.00	2.00	1.00	2.00

	W2	W3	W4	W5	W6	E1	V1	V2
127	2.00	1.00	2.00	2.00	2.00	1.00	2.00	2.00
128	1.00	1.00	1.00	1.00	1.00	1.00	2.00	2.00
129	1.00	1.00	1.00	1.00	1.00	1.00	2.00	2.00
130	1.00	2.00	1.00	1.00	2.00	1.00	2.00	2.00
131	1.00	1.00	1.00	1.00	2.00	1.00	2.00	2.00
132	2.00	1.00	1.00	1.00	2.00	1.00	2.00	2.00
133	1.00	1.00	2.00	2.00	2.00	1.00	2.00	2.00
134	1.00	1.00	1.00	1.00	1.00	1.00	1.00	1.00
135	1.00	2.00	1.00	1.00	2.00	1.00	2.00	2.00
136	2.00	1.00	1.00	1.00	2.00	1.00	2.00	2.00
137	1.00	2.00	2.00	1.00	2.00	1.00	1.00	1.00
138	2.00	1.00	2.00	1.00	1.00	1.00	2.00	2.00
139	2.00	1.00	1.00	1.00	2.00	1.00	1.00	2.00
140	2.00	1.00	2.00	2.00	2.00	1.00	2.00	2.00
141	1.00	1.00	2.00	1.00	2.00	1.00	2.00	2.00
142	2.00	2.00	2.00	1.00	2.00	1.00	2.00	2.00
143	1.00	1.00	1.00	1.00	2.00	1.00	2.00	2.00
144	2.00	2.00	2.00	2.00	2.00	1.00	1.00	2.00
145	2.00	1.00	2.00	2.00	2.00	1.00	2.00	2.00
146	1.00	1.00	1.00	1.00	2.00	1.00	1.00	1.00
147	1.00	2.00	1.00	1.00	1.00	1.00	2.00	2.00
148	1.00	1.00	1.00	1.00	1.00	1.00	2.00	2.00
149	1.00	1.00	1.00	1.00	1.00	1.00	1.00	2.00
150	2.00	1.00	1.00	1.00	1.00	1.00	2.00	2.00
151	1.00	1.00	1.00	1.00	1.00	1.00	2.00	1.00
152	1.00	1.00	1.00	1.00	1.00	1.00	1.00	1.00
153	1.00	1.00	1.00	1.00	1.00	1.00	2.00	2.00
154	2.00	1.00	1.00	1.00	2.00	1.00	2.00	2.00
155	1.00	2.00	1.00	1.00	2.00	1.00	1.00	2.00
156	2.00	1.00	1.00	1.00	2.00	1.00	2.00	2.00
157	1.00	1.00	1.00	1.00	1.00	1.00	2.00	2.00
158	1.00	2.00	1.00	2.00	1.00	1.00	1.00	2.00
159	2.00	1.00	2.00	1.00	2.00	1.00	2.00	2.00
160	1.00	1.00	1.00	1.00	1.00	1.00	2.00	2.00
161	1.00	1.00	2.00	1.00	2.00	1.00	1.00	1.00
162	1.00	1.00	1.00	1.00	1.00	1.00	1.00	1.00
163	2.00	1.00	2.00	1.00	1.00	1.00	2.00	2.00
164	2.00	1.00	1.00	1.00	2.00	1.00	2.00	2.00
165	1.00	2.00	2.00	1.00	2.00	1.00	1.00	1.00
166	1.00	1.00	1.00	1.00	2.00	1.00	2.00	2.00
167	1.00	1.00	1.00	1.00	1.00	1.00	2.00	2.00
168	2.00	1.00	1.00	1.00	1.00	1.00	2.00	2.00

	V3	V4	V5	V6	E2	E3	E4	C1
127	1.00	2.00	2.00	2.00	1.00	2.00	1.00	2.00
128	2.00	1.00	2.00	2.00	1.00	2.00	1.00	2.00
129	1.00	1.00	1.00	1.00	1.00	2.00	1.00	2.00
130	2.00	2.00	1.00	2.00	1.00	2.00	1.00	2.00
131	2.00	2.00	1.00	1.00	1.00	2.00	1.00	2.00
132	2.00	1.00	2.00	2.00	1.00	2.00	1.00	2.00
133	1.00	2.00	1.00	1.00	1.00	2.00	2.00	2.00
134	1.00	1.00	1.00	1.00	1.00	2.00	1.00	1.00
135	1.00	2.00	1.00	2.00	1.00	2.00	2.00	2.00
136	1.00	2.00	2.00	2.00	1.00	2.00	2.00	2.00
137	2.00	2.00	1.00	1.00	1.00	2.00	2.00	2.00
138	2.00	2.00	1.00	1.00	1.00	2.00	2.00	2.00
139	1.00	1.00	1.00	2.00	1.00	2.00	2.00	2.00
140	2.00	2.00	1.00	1.00	1.00	2.00	2.00	2.00
141	2.00	1.00	1.00	1.00	1.00	2.00	1.00	2.00
142	1.00	2.00	1.00	2.00	1.00	2.00	1.00	2.00
143	1.00	1.00	1.00	2.00	1.00	2.00	1.00	2.00
144	1.00	2.00	1.00	2.00	1.00	2.00	1.00	2.00
145	2.00	2.00	2.00	2.00	1.00	2.00	2.00	2.00
146	1.00	1.00	1.00	1.00	1.00	2.00	1.00	1.00
147	2.00	1.00	2.00	2.00	1.00	2.00	1.00	1.00
148	1.00	1.00	2.00	2.00	1.00	2.00	2.00	2.00
149	2.00	2.00	2.00	2.00	1.00	2.00	2.00	2.00
150	2.00	1.00	1.00	1.00	1.00	2.00	2.00	2.00
151	1.00	1.00	2.00	1.00	1.00	1.00	1.00	2.00
152	1.00	1.00	1.00	1.00	1.00	2.00	1.00	2.00
153	2.00	2.00	2.00	2.00	1.00	2.00	2.00	2.00
154	2.00	1.00	2.00	2.00	1.00	2.00	2.00	2.00
155	1.00	1.00	2.00	1.00	1.00	2.00	2.00	2.00
156	1.00	2.00	1.00	2.00	1.00	2.00	2.00	2.00
157	2.00	2.00	1.00	1.00	1.00	2.00	2.00	2.00
158	1.00	2.00	1.00	2.00	1.00	2.00	1.00	1.00
159	2.00	2.00	2.00	1.00	1.00	2.00	1.00	2.00
160	2.00	1.00	1.00	2.00	1.00	2.00	2.00	2.00
161	1.00	2.00	1.00	1.00	1.00	2.00	2.00	2.00
162	1.00	1.00	1.00	1.00	1.00	2.00	1.00	1.00
163	2.00	2.00	2.00	1.00	1.00	2.00	2.00	2.00
164	2.00	2.00	2.00	1.00	1.00	2.00	1.00	2.00
165	2.00	2.00	1.00	2.00	1.00	2.00	1.00	2.00
166	2.00	1.00	2.00	1.00	1.00	2.00	1.00	2.00
167	2.00	1.00	2.00	2.00	1.00	2.00	1.00	2.00
168	2.00	2.00	2.00	1.00	1.00	2.00	1.00	2.00

	C2	C3	C4	C5	C6	C7	filter_$
127	2.00	1.00	1.00	2.00	2.00	2.00	1
128	2.00	1.00	2.00	1.00	2.00	2.00	1
129	2.00	1.00	1.00	1.00	2.00	2.00	1
130	2.00	1.00	1.00	1.00	2.00	2.00	1
131	2.00	1.00	2.00	2.00	2.00	2.00	1
132	2.00	1.00	1.00	1.00	2.00	2.00	1
133	2.00	1.00	2.00	2.00	2.00	2.00	1
134	1.00	1.00	1.00	1.00	1.00	2.00	1
135	2.00	1.00	1.00	2.00	2.00	2.00	1
136	2.00	1.00	2.00	2.00	2.00	2.00	1
137	2.00	1.00	2.00	2.00	2.00	2.00	1
138	2.00	1.00	2.00	2.00	2.00	2.00	1
139	2.00	1.00	1.00	1.00	2.00	2.00	1
140	2.00	1.00	2.00	1.00	2.00	2.00	1
141	1.00	1.00	2.00	2.00	1.00	2.00	1
142	2.00	1.00	2.00	2.00	2.00	2.00	1
143	2.00	1.00	2.00	2.00	2.00	2.00	1
144	2.00	2.00	2.00	2.00	2.00	1.00	1
145	2.00	1.00	1.00	2.00	2.00	2.00	1
146	1.00	1.00	1.00	1.00	1.00	2.00	1
147	2.00	1.00	2.00	2.00	2.00	2.00	1
148	2.00	1.00	1.00	2.00	1.00	2.00	1
149	2.00	1.00	1.00	1.00	1.00	2.00	1
150	2.00	1.00	1.00	1.00	2.00	2.00	1
151	2.00	1.00	1.00	2.00	2.00	2.00	1
152	2.00	2.00	2.00	2.00	2.00	1.00	1
153	2.00	1.00	1.00	1.00	2.00	2.00	1
154	2.00	1.00	1.00	2.00	2.00	2.00	1
155	2.00	1.00	1.00	1.00	2.00	2.00	1
156	2.00	1.00	2.00	2.00	2.00	2.00	1
157	2.00	1.00	2.00	2.00	2.00	2.00	1
158	2.00	1.00	2.00	1.00	2.00	2.00	1
159	2.00	1.00	2.00	2.00	2.00	2.00	1
160	2.00	1.00	2.00	2.00	2.00	2.00	1
161	1.00	1.00	1.00	2.00	2.00	2.00	1
162	1.00	1.00	1.00	1.00	1.00	2.00	1
163	2.00	1.00	1.00	1.00	2.00	2.00	1
164	2.00	2.00	2.00	2.00	2.00	2.00	1
165	2.00	1.00	1.00	2.00	2.00	2.00	1
166	2.00	1.00	1.00	1.00	2.00	2.00	1
167	2.00	1.00	2.00	2.00	2.00	2.00	1
168	2.00	1.00	2.00	2.00	2.00	2.00	1

	SN	Group	Gender	Age	Work	Work2	Work3	Sector
169	223.00	1.00	1.00	21.00	3.00	1.00	1.00	1.00
170	227.00	1.00	1.00	24.00	6.00	1.00	1.00	1.00
171	228.00	1.00	1.00	23.00	6.00	1.00	1.00	1.00
172	229.00	1.00	1.00	24.00	6.00	1.00	1.00	1.00
173	230.00	1.00	1.00	23.00	6.00	1.00	1.00	1.00
174	231.00	1.00	1.00	23.00	6.00	1.00	1.00	1.00
175	232.00	1.00	1.00	24.00	6.00	1.00	1.00	1.00
176	233.00	1.00	2.00	23.00	6.00	1.00	1.00	1.00
177	235.00	1.00	2.00	23.00	5.00	1.00	1.00	1.00
178	236.00	1.00	1.00	22.00	6.00	1.00	1.00	1.00
179	237.00	1.00	1.00	24.00	6.00	1.00	1.00	1.00
180	238.00	1.00	2.00	22.00	4.00	1.00	1.00	1.00
181	239.00	1.00	1.00	24.00	6.00	1.00	1.00	1.00
182	240.00	1.00	1.00	22.00	4.00	1.00	1.00	1.00
183	241.00	1.00	1.00	23.00	4.00	1.00	1.00	1.00
184	243.00	1.00	1.00	20.00	2.00	1.00	1.00	1.00
185	244.00	1.00	2.00	23.00	6.00	1.00	1.00	1.00
186	245.00	1.00	2.00	23.00	5.00	1.00	1.00	1.00
187	246.00	1.00	1.00	24.00	7.00	2.00	2.00	1.00
188	248.00	1.00	2.00	23.00	6.00	1.00	1.00	1.00
189	249.00	1.00	1.00	21.00	4.00	1.00	1.00	1.00
190	251.00	1.00	1.00	20.00	2.00	1.00	1.00	1.00
191	254.00	1.00	2.00	20.00	3.00	1.00	1.00	1.00
192	255.00	1.00	1.00	20.00	3.00	1.00	1.00	1.00
193	257.00	1.00	1.00	25.00	5.00	1.00	1.00	1.00
194	258.00	1.00	1.00	22.00	3.00	1.00	1.00	1.00
195	259.00	1.00	2.00	21.00	2.00	1.00	1.00	1.00
196	260.00	1.00	1.00	21.00	3.00	1.00	1.00	1.00
197	261.00	1.00	1.00	20.00	3.00	1.00	1.00	1.00
198	264.00	1.00	1.00	28.00	8.00	2.00	2.00	2.00
199	267.00	1.00	1.00	25.00	8.00	2.00	2.00	1.00
200	268.00	1.00	1.00	25.00	8.00	2.00	2.00	1.00
201	269.00	1.00	2.00	25.00	8.00	2.00	2.00	1.00
202	270.00	1.00	2.00	25.00	8.00	2.00	2.00	1.00
203	271.00	1.00	1.00	23.00	6.00	1.00	1.00	1.00
204	272.00	1.00	2.00	23.00	6.00	1.00	1.00	1.00
205	273.00	1.00	1.00	23.00	6.00	1.00	1.00	1.00
206	274.00	1.00	2.00	23.00	6.00	1.00	1.00	1.00
207	275.00	1.00	1.00	23.00	6.00	1.00	1.00	1.00
208	276.00	1.00	2.00	23.00	6.00	1.00	1.00	1.00
209	277.00	1.00	1.00	24.00	6.00	1.00	1.00	1.00
210	278.00	1.00	2.00	24.00	6.00	1.00	1.00	1.00

	Region	Nationality	M1	M2	M3	M4	M5	M6
169	2.00	1.00	2.00	1.00	2.00	1.00	1.00	1.00
170	2.00	1.00	2.00	1.00	2.00	1.00	2.00	1.00
171	2.00	1.00	2.00	1.00	2.00	2.00	2.00	2.00
172	2.00	1.00	1.00	1.00	2.00	1.00	1.00	1.00
173	2.00	1.00	1.00	2.00	1.00	1.00	1.00	2.00
174	1.00	1.00	1.00	1.00	2.00	2.00	1.00	1.00
175	1.00	1.00	1.00	1.00	1.00	1.00	1.00	2.00
176	1.00	1.00	1.00	1.00	1.00	1.00	1.00	1.00
177	5.00	1.00	1.00	1.00	2.00	2.00	1.00	1.00
178	2.00	1.00	1.00	1.00	2.00	1.00	1.00	1.00
179	2.00	1.00	1.00	1.00	2.00	1.00	1.00	2.00
180	1.00	1.00	1.00	2.00	1.00	2.00	1.00	2.00
181	2.00	1.00	1.00	2.00	1.00	1.00	1.00	1.00
182	1.00	1.00	2.00	1.00	1.00	1.00	1.00	1.00
183	4.00	1.00	1.00	1.00	1.00	1.00	2.00	1.00
184	2.00	1.00	2.00	2.00	2.00	2.00	2.00	2.00
185	5.00	1.00	1.00	2.00	1.00	1.00	1.00	1.00
186	5.00	1.00	1.00	1.00	1.00	1.00	1.00	1.00
187	1.00	1.00	1.00	1.00	1.00	1.00	1.00	2.00
188	4.00	1.00	1.00	1.00	1.00	1.00	1.00	1.00
189	2.00	1.00	2.00	2.00	1.00	2.00	2.00	2.00
190	5.00	1.00	2.00	1.00	2.00	1.00	2.00	1.00
191	4.00	1.00	1.00	2.00	2.00	1.00	2.00	1.00
192	1.00	1.00	1.00	1.00	1.00	1.00	1.00	1.00
193	5.00	1.00	1.00	1.00	1.00	1.00	1.00	1.00
194	1.00	1.00	1.00	1.00	2.00	2.00	1.00	1.00
195	5.00	1.00	1.00	1.00	2.00	1.00	2.00	1.00
196	1.00	1.00	1.00	1.00	1.00	1.00	1.00	1.00
197	1.00	1.00	2.00	2.00	2.00	1.00	2.00	2.00
198	3.00	1.00	1.00	1.00	1.00	1.00	1.00	1.00
199	1.00	1.00	1.00	1.00	1.00	1.00	2.00	2.00
200	1.00	1.00	2.00	2.00	2.00	1.00	1.00	1.00
201	1.00	1.00	2.00	2.00	2.00	1.00	1.00	1.00
202	1.00	1.00	1.00	1.00	2.00	1.00	1.00	1.00
203	4.00	1.00	1.00	1.00	1.00	1.00	1.00	2.00
204	1.00	1.00	2.00	1.00	2.00	1.00	1.00	2.00
205	4.00	1.00	1.00	1.00	2.00	1.00	1.00	2.00
206	4.00	1.00	2.00	2.00	2.00	1.00	1.00	2.00
207	4.00	1.00	2.00	1.00	2.00	1.00	2.00	1.00
208	4.00	1.00	1.00	2.00	1.00	1.00	2.00	2.00
209	4.00	1.00	1.00	1.00	2.00	2.00	1.00	1.00
210	4.00	1.00	1.00	1.00	2.00	2.00	1.00	1.00

K68 SPSS v14.sav

	M7	M8	M9	M10	M11	M12	M13	M14
169	2.00	1.00	2.00	2.00	1.00	2.00	1.00	1.00
170	2.00	1.00	2.00	1.00	2.00	1.00	2.00	1.00
171	2.00	2.00	2.00	2.00	2.00	1.00	2.00	1.00
172	2.00	2.00	2.00	1.00	1.00	2.00	1.00	1.00
173	1.00	1.00	2.00	2.00	2.00	2.00	2.00	1.00
174	1.00	1.00	1.00	2.00	2.00	1.00	1.00	1.00
175	2.00	1.00	1.00	1.00	1.00	1.00	1.00	1.00
176	1.00	1.00	1.00	1.00	1.00	1.00	1.00	1.00
177	2.00	1.00	1.00	1.00	2.00	1.00	1.00	1.00
178	1.00	1.00	1.00	1.00	1.00	1.00	2.00	1.00
179	2.00	1.00	1.00	1.00	2.00	2.00	2.00	1.00
180	1.00	2.00	1.00	2.00	1.00	2.00	1.00	2.00
181	2.00	1.00	1.00	2.00	2.00	1.00	1.00	1.00
182	2.00	1.00	1.00	1.00	2.00	1.00	1.00	1.00
183	1.00	2.00	1.00	1.00	1.00	1.00	1.00	2.00
184	1.00	2.00	2.00	1.00	1.00	1.00	2.00	1.00
185	2.00	1.00	2.00	1.00	2.00	1.00	2.00	1.00
186	1.00	1.00	1.00	1.00	1.00	2.00	1.00	1.00
187	2.00	2.00	2.00	1.00	1.00	2.00	2.00	1.00
188	1.00	1.00	1.00	2.00	2.00	1.00	2.00	1.00
189	2.00	2.00	2.00	2.00	1.00	2.00	2.00	2.00
190	1.00	1.00	1.00	1.00	1.00	1.00	2.00	1.00
191	2.00	1.00	2.00	2.00	2.00	2.00	2.00	2.00
192	2.00	1.00	1.00	1.00	2.00	1.00	1.00	1.00
193	1.00	1.00	1.00	1.00	2.00	1.00	1.00	1.00
194	1.00	1.00	1.00	1.00	2.00	1.00	2.00	1.00
195	2.00	1.00	2.00	1.00	2.00	1.00	1.00	2.00
196	1.00	1.00	1.00	1.00	1.00	1.00	1.00	1.00
197	2.00	1.00	1.00	2.00	2.00	2.00	2.00	1.00
198	1.00	1.00	1.00	1.00	2.00	1.00	2.00	1.00
199	1.00	1.00	1.00	2.00	1.00	1.00	2.00	2.00
200	1.00	2.00	2.00	1.00	1.00	2.00	2.00	1.00
201	1.00	1.00	1.00	1.00	2.00	1.00	2.00	1.00
202	1.00	1.00	1.00	1.00	1.00	1.00	1.00	1.00
203	2.00	1.00	1.00	1.00	2.00	1.00	1.00	2.00
204	1.00	1.00	1.00	2.00	2.00	1.00	2.00	1.00
205	1.00	1.00	1.00	1.00	2.00	2.00	2.00	1.00
206	2.00	1.00	2.00	1.00	1.00	1.00	1.00	1.00
207	2.00	1.00	1.00	1.00	1.00	1.00	2.00	1.00
208	1.00	1.00	2.00	2.00	2.00	1.00	2.00	1.00
209	1.00	1.00	1.00	1.00	2.00	1.00	1.00	1.00
210	2.00	1.00	1.00	1.00	2.00	1.00	1.00	1.00

	M15	B1	B2	B3	B4	B5	B6	B7
169	1.00	2.00	2.00	2.00	1.00	2.00	2.00	2.00
170	1.00	1.00	2.00	2.00	2.00	2.00	2.00	1.00
171	2.00	2.00	1.00	1.00	1.00	1.00	2.00	2.00
172	1.00	1.00	1.00	1.00	1.00	1.00	1.00	1.00
173	1.00	1.00	1.00	1.00	2.00	2.00	2.00	1.00
174	1.00	1.00	1.00	1.00	2.00	2.00	2.00	2.00
175	1.00	1.00	1.00	2.00	1.00	2.00	2.00	2.00
176	1.00	2.00	1.00	1.00	1.00	2.00	2.00	2.00
177	1.00	2.00	1.00	1.00	1.00	1.00	1.00	1.00
178	1.00	2.00	1.00	2.00	2.00	2.00	2.00	2.00
179	1.00	1.00	1.00	1.00	1.00	1.00	1.00	1.00
180	1.00	2.00	1.00	2.00	1.00	2.00	1.00	2.00
181	1.00	2.00	1.00	2.00	2.00	2.00	1.00	1.00
182	2.00	2.00	1.00	2.00	1.00	2.00	1.00	1.00
183	1.00	1.00	2.00	2.00	2.00	1.00	1.00	1.00
184	2.00	1.00	2.00	2.00	2.00	2.00	2.00	2.00
185	2.00	2.00	1.00	1.00	1.00	1.00	1.00	1.00
186	1.00	1.00	2.00	2.00	1.00	2.00	2.00	2.00
187	1.00	1.00	1.00	1.00	1.00	1.00	2.00	2.00
188	1.00	1.00	2.00	2.00	2.00	2.00	1.00	1.00
189	2.00	1.00	1.00	1.00	2.00	2.00	2.00	2.00
190	1.00	2.00	1.00	2.00	1.00	1.00	2.00	2.00
191	2.00	1.00	1.00	2.00	2.00	2.00	2.00	2.00
192	1.00	1.00	1.00	1.00	1.00	1.00	2.00	2.00
193	2.00	1.00	1.00	1.00	1.00	1.00	1.00	1.00
194	1.00	2.00	1.00	1.00	1.00	2.00	2.00	2.00
195	2.00	1.00	1.00	1.00	1.00	1.00	1.00	1.00
196	2.00	1.00	2.00	1.00	1.00	1.00	2.00	2.00
197	1.00	2.00	1.00	1.00	2.00	2.00	2.00	2.00
198	1.00	2.00	2.00	2.00	2.00	2.00	2.00	2.00
199	2.00	1.00	1.00	2.00	2.00	1.00	1.00	1.00
200	2.00	2.00	1.00	1.00	2.00	2.00	2.00	2.00
201	1.00	2.00	2.00	2.00	2.00	1.00	2.00	1.00
202	2.00	2.00	2.00	2.00	1.00	2.00	2.00	2.00
203	1.00	2.00	2.00	2.00	2.00	2.00	2.00	2.00
204	2.00	1.00	1.00	2.00	1.00	1.00	1.00	1.00
205	1.00	2.00	1.00	2.00	1.00	2.00	2.00	2.00
206	1.00	1.00	2.00	1.00	1.00	1.00	2.00	2.00
207	2.00	2.00	1.00	1.00	1.00	2.00	2.00	2.00
208	2.00	2.00	1.00	1.00	1.00	2.00	1.00	1.00
209	1.00	1.00	1.00	2.00	2.00	2.00	2.00	2.00
210	2.00	1.00	1.00	2.00	2.00	1.00	2.00	2.00

	B8	B9	B10	B11	B12	B13	B14	W1
169	1.00	1.00	1.00	1.00	2.00	2.00	2.00	1.00
170	1.00	2.00	1.00	2.00	1.00	1.00	2.00	2.00
171	1.00	2.00	2.00	2.00	2.00	2.00	2.00	2.00
172	1.00	1.00	1.00	1.00	1.00	1.00	1.00	1.00
173	2.00	2.00	1.00	1.00	2.00	2.00	2.00	1.00
174	2.00	2.00	2.00	2.00	2.00	1.00	1.00	2.00
175	2.00	2.00	2.00	2.00	2.00	2.00	2.00	2.00
176	2.00	2.00	1.00	1.00	2.00	2.00	2.00	2.00
177	1.00	1.00	1.00	1.00	2.00	1.00	1.00	2.00
178	2.00	1.00	1.00	2.00	2.00	2.00	1.00	1.00
179	1.00	2.00	2.00	1.00	2.00	1.00	1.00	1.00
180	1.00	2.00	1.00	2.00	1.00	2.00	1.00	1.00
181	2.00	2.00	1.00	1.00	2.00	2.00	1.00	2.00
182	1.00	2.00	1.00	1.00	2.00	2.00	2.00	1.00
183	1.00	1.00	1.00	1.00	1.00	1.00	1.00	1.00
184	2.00	2.00	1.00	1.00	1.00	2.00	1.00	2.00
185	2.00	1.00	1.00	1.00	1.00	2.00	1.00	2.00
186	2.00	2.00	2.00	2.00	2.00	2.00	2.00	1.00
187	2.00	2.00	1.00	1.00	2.00	2.00	2.00	2.00
188	1.00	1.00	1.00	1.00	1.00	2.00	1.00	1.00
189	2.00	2.00	1.00	1.00	2.00	2.00	2.00	2.00
190	1.00	2.00	1.00	1.00	2.00	2.00	2.00	1.00
191	2.00	2.00	2.00	2.00	2.00	2.00	2.00	2.00
192	2.00	1.00	1.00	2.00	2.00	2.00	2.00	1.00
193	1.00	1.00	1.00	1.00	1.00	2.00	2.00	1.00
194	2.00	2.00	1.00	1.00	2.00	2.00	2.00	1.00
195	1.00	1.00	1.00	1.00	2.00	2.00	1.00	1.00
196	2.00	2.00	2.00	1.00	2.00	2.00	2.00	2.00
197	2.00	2.00	2.00	1.00	2.00	2.00	1.00	2.00
198	2.00	2.00	2.00	2.00	2.00	2.00	2.00	1.00
199	1.00	1.00	1.00	1.00	2.00	2.00	1.00	1.00
200	1.00	1.00	1.00	2.00	1.00	2.00	1.00	2.00
201	1.00	1.00	1.00	1.00	1.00	1.00	1.00	1.00
202	2.00	2.00	1.00	1.00	1.00	2.00	2.00	2.00
203	2.00	2.00	2.00	2.00	2.00	2.00	2.00	1.00
204	2.00	1.00	1.00	1.00	2.00	1.00	1.00	2.00
205	2.00	2.00	2.00	1.00	2.00	2.00	2.00	1.00
206	2.00	1.00	1.00	1.00	2.00	2.00	1.00	2.00
207	2.00	2.00	2.00	2.00	2.00	2.00	2.00	1.00
208	1.00	1.00	1.00	1.00	2.00	2.00	1.00	1.00
209	2.00	2.00	2.00	2.00	2.00	2.00	2.00	1.00
210	2.00	2.00	1.00	1.00	2.00	2.00	1.00	2.00

	W2	W3	W4	W5	W6	E1	V1	V2
169	1.00	1.00	1.00	1.00	1.00	1.00	1.00	1.00
170	1.00	2.00	1.00	2.00	1.00	1.00	1.00	1.00
171	2.00	2.00	2.00	1.00	2.00	1.00	2.00	2.00
172	1.00	2.00	2.00	1.00	2.00	1.00	2.00	2.00
173	1.00	2.00	2.00	1.00	1.00	1.00	2.00	2.00
174	2.00	2.00	2.00	1.00	1.00	1.00	2.00	2.00
175	2.00	1.00	1.00	1.00	1.00	1.00	2.00	2.00
176	2.00	1.00	2.00	2.00	2.00	1.00	2.00	2.00
177	2.00	1.00	1.00	1.00	2.00	1.00	2.00	2.00
178	2.00	2.00	2.00	1.00	2.00	1.00	2.00	2.00
179	1.00	1.00	1.00	1.00	2.00	1.00	2.00	1.00
180	2.00	1.00	2.00	1.00	2.00	1.00	1.00	2.00
181	1.00	1.00	2.00	1.00	2.00	1.00	2.00	2.00
182	1.00	2.00	2.00	1.00	2.00	1.00	2.00	2.00
183	1.00	2.00	1.00	1.00	1.00	1.00	1.00	1.00
184	2.00	2.00	2.00	1.00	1.00	1.00	2.00	2.00
185	2.00	1.00	1.00	2.00	1.00	1.00	2.00	2.00
186	2.00	1.00	2.00	1.00	2.00	1.00	2.00	2.00
187	1.00	2.00	2.00	1.00	2.00	1.00	2.00	2.00
188	1.00	1.00	1.00	1.00	2.00	1.00	2.00	2.00
189	2.00	1.00	2.00	1.00	1.00	1.00	2.00	2.00
190	1.00	1.00	2.00	1.00	2.00	1.00	2.00	2.00
191	2.00	1.00	2.00	1.00	2.00	1.00	2.00	2.00
192	1.00	1.00	1.00	1.00	2.00	1.00	2.00	2.00
193	1.00	1.00	1.00	1.00	2.00	1.00	2.00	2.00
194	1.00	1.00	1.00	1.00	1.00	1.00	1.00	2.00
195	2.00	1.00	2.00	1.00	2.00	1.00	1.00	1.00
196	2.00	2.00	2.00	2.00	2.00	1.00	2.00	2.00
197	1.00	2.00	1.00	2.00	2.00	1.00	2.00	2.00
198	1.00	1.00	1.00	1.00	1.00	1.00	2.00	2.00
199	1.00	1.00	1.00	2.00	1.00	1.00	2.00	2.00
200	2.00	2.00	2.00	2.00	2.00	1.00	1.00	1.00
201	1.00	1.00	1.00	1.00	1.00	1.00	1.00	2.00
202	2.00	1.00	1.00	1.00	1.00	1.00	2.00	2.00
203	1.00	1.00	1.00	2.00	2.00	1.00	2.00	1.00
204	2.00	1.00	2.00	1.00	1.00	1.00	2.00	2.00
205	2.00	1.00	1.00	1.00	2.00	1.00	2.00	2.00
206	2.00	1.00	1.00	1.00	2.00	1.00	2.00	2.00
207	1.00	1.00	1.00	2.00	2.00	1.00	2.00	2.00
208	1.00	2.00	2.00	2.00	2.00	1.00	2.00	2.00
209	2.00	1.00	2.00	1.00	2.00	1.00	2.00	2.00
210	2.00	1.00	1.00	1.00	2.00	1.00	2.00	2.00

	V3	V4	V5	V6	E2	E3	E4	C1
169	1.00	1.00	1.00	1.00	1.00	2.00	2.00	2.00
170	2.00	2.00	1.00	2.00	1.00	1.00	2.00	1.00
171	2.00	2.00	2.00	1.00	1.00	2.00	2.00	2.00
172	1.00	2.00	1.00	1.00	1.00	2.00	2.00	2.00
173	2.00	2.00	2.00	1.00	1.00	2.00	2.00	2.00
174	2.00	1.00	2.00	2.00	1.00	2.00	1.00	2.00
175	2.00	2.00	1.00	1.00	1.00	2.00	2.00	2.00
176	2.00	2.00	2.00	2.00	1.00	2.00	1.00	2.00
177	2.00	1.00	1.00	1.00	1.00	2.00	2.00	2.00
178	1.00	2.00	1.00	2.00	1.00	2.00	1.00	2.00
179	1.00	2.00	2.00	2.00	1.00	2.00	1.00	2.00
180	1.00	2.00	1.00	2.00	1.00	2.00	1.00	2.00
181	2.00	1.00	1.00	2.00	1.00	2.00	1.00	2.00
182	2.00	1.00	2.00	2.00	1.00	2.00	2.00	2.00
183	1.00	2.00	1.00	2.00	1.00	2.00	1.00	1.00
184	2.00	1.00	1.00	1.00	1.00	2.00	2.00	2.00
185	2.00	1.00	2.00	2.00	1.00	2.00	1.00	2.00
186	2.00	1.00	1.00	2.00	1.00	2.00	1.00	2.00
187	2.00	2.00	1.00	2.00	1.00	2.00	2.00	2.00
188	2.00	1.00	2.00	2.00	1.00	2.00	2.00	2.00
189	2.00	1.00	1.00	2.00	1.00	2.00	1.00	2.00
190	2.00	1.00	1.00	2.00	1.00	2.00	2.00	2.00
191	1.00	1.00	2.00	1.00	1.00	2.00	2.00	2.00
192	2.00	1.00	2.00	1.00	1.00	2.00	2.00	2.00
193	2.00	1.00	2.00	2.00	1.00	2.00	2.00	2.00
194	1.00	1.00	2.00	1.00	1.00	2.00	2.00	2.00
195	2.00	1.00	2.00	1.00	1.00	1.00	1.00	1.00
196	2.00	2.00	2.00	2.00	1.00	2.00	2.00	2.00
197	1.00	2.00	2.00	2.00	1.00	2.00	2.00	2.00
198	1.00	1.00	1.00	1.00	1.00	2.00	1.00	2.00
199	1.00	1.00	2.00	1.00	1.00	1.00	1.00	2.00
200	2.00	2.00	2.00	2.00	1.00	2.00	1.00	2.00
201	1.00	1.00	1.00	2.00	1.00	2.00	1.00	2.00
202	2.00	2.00	2.00	2.00	1.00	2.00	1.00	2.00
203	1.00	1.00	1.00	2.00	1.00	2.00	1.00	2.00
204	2.00	2.00	2.00	2.00	1.00	2.00	2.00	1.00
205	2.00	2.00	1.00	1.00	1.00	2.00	1.00	2.00
206	2.00	2.00	1.00	2.00	1.00	2.00	2.00	2.00
207	1.00	2.00	1.00	2.00	1.00	2.00	2.00	2.00
208	2.00	2.00	1.00	2.00	1.00	2.00	2.00	2.00
209	1.00	2.00	2.00	2.00	1.00	2.00	2.00	2.00
210	1.00	1.00	2.00	2.00	1.00	2.00	2.00	2.00

	C2	C3	C4	C5	C6	C7	filter_$
169	1.00	1.00	2.00	1.00	1.00	2.00	1
170	2.00	1.00	2.00	1.00	1.00	2.00	1
171	2.00	1.00	2.00	2.00	2.00	2.00	1
172	2.00	1.00	1.00	2.00	1.00	2.00	1
173	2.00	1.00	1.00	1.00	1.00	2.00	1
174	2.00	1.00	2.00	2.00	2.00	2.00	1
175	2.00	2.00	2.00	2.00	2.00	2.00	1
176	2.00	1.00	2.00	1.00	2.00	2.00	1
177	2.00	1.00	2.00	2.00	2.00	2.00	1
178	2.00	1.00	2.00	2.00	2.00	2.00	1
179	2.00	1.00	1.00	2.00	2.00	2.00	1
180	1.00	2.00	1.00	2.00	1.00	2.00	1
181	2.00	1.00	1.00	1.00	1.00	2.00	1
182	2.00	1.00	1.00	2.00	1.00	2.00	1
183	1.00	1.00	1.00	1.00	1.00	2.00	1
184	2.00	1.00	1.00	2.00	2.00	2.00	1
185	2.00	1.00	2.00	2.00	2.00	2.00	1
186	2.00	1.00	1.00	2.00	2.00	2.00	1
187	2.00	1.00	2.00	2.00	2.00	2.00	1
188	2.00	1.00	2.00	2.00	2.00	2.00	1
189	2.00	1.00	2.00	2.00	2.00	2.00	1
190	2.00	2.00	2.00	2.00	2.00	2.00	1
191	2.00	1.00	1.00	2.00	2.00	2.00	1
192	2.00	2.00	2.00	2.00	2.00	1.00	1
193	2.00	2.00	2.00	1.00	2.00	2.00	1
194	2.00	1.00	1.00	1.00	2.00	2.00	1
195	1.00	1.00	1.00	2.00	1.00	2.00	1
196	2.00	2.00	2.00	2.00	2.00	1.00	1
197	2.00	1.00	2.00	2.00	2.00	2.00	1
198	2.00	1.00	1.00	1.00	2.00	2.00	1
199	2.00	1.00	2.00	2.00	2.00	2.00	1
200	2.00	1.00	2.00	2.00	2.00	2.00	1
201	2.00	1.00	1.00	2.00	2.00	2.00	1
202	2.00	1.00	1.00	2.00	2.00	2.00	1
203	2.00	1.00	1.00	2.00	2.00	2.00	1
204	2.00	1.00	1.00	2.00	2.00	2.00	1
205	2.00	1.00	2.00	2.00	2.00	2.00	1
206	2.00	1.00	2.00	2.00	2.00	2.00	1
207	2.00	1.00	1.00	2.00	2.00	2.00	1
208	2.00	1.00	1.00	2.00	2.00	2.00	1
209	2.00	1.00	2.00	2.00	2.00	2.00	1
210	2.00	1.00	1.00	2.00	1.00	2.00	1

	SN	Group	Gender	Age	Work	Work2	Work3	Sector
211	279.00	1.00	2.00	21.00	3.00	1.00	1.00	1.00
212	280.00	1.00	1.00	24.00	6.00	1.00	1.00	1.00
213	281.00	1.00	2.00	23.00	6.00	1.00	1.00	1.00
214	282.00	1.00	1.00	37.00	10.00	3.00	2.00	1.00
215	283.00	1.00	2.00	23.00	6.00	1.00	1.00	1.00
216	284.00	1.00	1.00	21.00	2.00	1.00	1.00	1.00
217	285.00	1.00	2.00	22.00	5.00	1.00	1.00	2.00
218	286.00	1.00	2.00	23.00	5.00	1.00	1.00	2.00
219	287.00	1.00	2.00	28.00	6.00	1.00	1.00	2.00
220	288.00	1.00	2.00	25.00	5.00	1.00	1.00	2.00
221	289.00	1.00	2.00	24.00	6.00	1.00	1.00	1.00
222	290.00	1.00	2.00	23.00	6.00	1.00	1.00	1.00
223	291.00	1.00	2.00	23.00	5.00	1.00	1.00	2.00
224	293.00	1.00	2.00	22.00	5.00	1.00	1.00	2.00
225	296.00	1.00	2.00	27.00	8.00	2.00	2.00	2.00
226	297.00	1.00	2.00	22.00	3.00	1.00	1.00	1.00
227	298.00	1.00	2.00	22.00	4.00	1.00	1.00	1.00
228	299.00	1.00	2.00	25.00	8.00	2.00	2.00	2.00
229	301.00	1.00	2.00	27.00	7.00	2.00	2.00	2.00
230	302.00	1.00	2.00	25.00	8.00	2.00	2.00	2.00
231	304.00	1.00	2.00	21.00	4.00	1.00	1.00	1.00
232	305.00	1.00	2.00	21.00	3.00	1.00	1.00	1.00
233	306.00	1.00	2.00	21.00	3.00	1.00	1.00	1.00
234	308.00	1.00	2.00	20.00	3.00	1.00	1.00	1.00
235	309.00	1.00	1.00	18.00	2.00	1.00	1.00	1.00
236	310.00	1.00	1.00	20.00	2.00	1.00	1.00	1.00
237	312.00	1.00	2.00	30.00	8.00	2.00	2.00	2.00
238	313.00	1.00	2.00	28.00	8.00	2.00	2.00	2.00
239	314.00	1.00	2.00	24.00	5.00	1.00	1.00	2.00
240	315.00	1.00	2.00	19.00	2.00	1.00	1.00	1.00
241	316.00	1.00	2.00	23.00	5.00	1.00	1.00	2.00
242	317.00	1.00	2.00	19.00	2.00	1.00	1.00	1.00
243	318.00	1.00	1.00	22.00	5.00	1.00	1.00	1.00
244	319.00	1.00	2.00	23.00	6.00	1.00	1.00	1.00
245	320.00	1.00	2.00	23.00	6.00	1.00	1.00	1.00
246	321.00	1.00	1.00	25.00	5.00	1.00	1.00	1.00
247	322.00	1.00	2.00	21.00	5.00	1.00	1.00	2.00
248	323.00	1.00	2.00	26.00	8.00	2.00	2.00	1.00
249	324.00	1.00	1.00	26.00	8.00	2.00	2.00	2.00
250	325.00	1.00	2.00	20.00	3.00	1.00	1.00	1.00
251	326.00	1.00	2.00	20.00	3.00	1.00	1.00	1.00
252	330.00	1.00	1.00	22.00	4.00	1.00	1.00	1.00

	Region	Nationality	M1	M2	M3	M4	M5	M6
211	2.00	1.00	1.00	1.00	1.00	1.00	1.00	1.00
212	4.00	1.00	1.00	1.00	1.00	1.00	1.00	1.00
213	4.00	1.00	1.00	1.00	1.00	1.00	1.00	1.00
214	5.00	1.00	1.00	1.00	2.00	1.00	2.00	2.00
215	4.00	1.00	1.00	1.00	1.00	1.00	1.00	1.00
216	2.00	1.00	1.00	2.00	1.00	1.00	1.00	1.00
217	2.00	1.00	1.00	1.00	1.00	1.00	1.00	2.00
218	1.00	1.00	1.00	1.00	1.00	1.00	1.00	1.00
219	1.00	1.00	1.00	1.00	1.00	1.00	1.00	1.00
220	5.00	1.00	2.00	1.00	2.00	1.00	1.00	1.00
221	4.00	1.00	1.00	2.00	2.00	1.00	2.00	2.00
222	4.00	1.00	2.00	2.00	2.00	2.00	1.00	2.00
223	1.00	1.00	1.00	2.00	2.00	2.00	1.00	2.00
224	1.00	1.00	1.00	1.00	1.00	1.00	1.00	1.00
225	1.00	1.00	2.00	2.00	2.00	1.00	2.00	1.00
226	2.00	1.00	2.00	1.00	2.00	2.00	1.00	1.00
227	3.00	1.00	1.00	2.00	1.00	1.00	1.00	1.00
228	1.00	1.00	2.00	1.00	2.00	1.00	1.00	1.00
229	1.00	1.00	2.00	1.00	2.00	2.00	1.00	1.00
230	1.00	1.00	1.00	1.00	1.00	2.00	1.00	2.00
231	4.00	1.00	1.00	1.00	1.00	1.00	1.00	1.00
232	4.00	1.00	1.00	1.00	1.00	1.00	1.00	1.00
233	4.00	1.00	2.00	2.00	1.00	1.00	1.00	1.00
234	4.00	1.00	1.00	1.00	1.00	1.00	1.00	1.00
235	4.00	1.00	1.00	2.00	2.00	1.00	1.00	1.00
236	4.00	1.00	1.00	2.00	1.00	1.00	1.00	2.00
237	2.00	1.00	1.00	1.00	2.00	1.00	2.00	1.00
238	1.00	1.00	1.00	1.00	2.00	2.00	2.00	2.00
239	1.00	1.00	1.00	1.00	1.00	1.00	1.00	1.00
240	4.00	1.00	1.00	1.00	2.00	1.00	1.00	1.00
241	4.00	1.00	1.00	1.00	1.00	2.00	2.00	1.00
242	1.00	1.00	1.00	1.00	1.00	1.00	1.00	1.00
243	1.00	1.00	1.00	1.00	1.00	1.00	2.00	1.00
244	3.00	1.00	1.00	1.00	2.00	1.00	1.00	2.00
245	3.00	1.00	1.00	1.00	1.00	1.00	1.00	1.00
246	5.00	1.00	2.00	2.00	2.00	2.00	2.00	1.00
247	1.00	1.00	1.00	1.00	2.00	2.00	1.00	2.00
248	2.00	1.00	1.00	1.00	1.00	1.00	1.00	2.00
249	5.00	1.00	1.00	1.00	1.00	1.00	1.00	1.00
250	1.00	1.00	2.00	1.00	1.00	1.00	1.00	1.00
251	5.00	1.00	2.00	1.00	2.00	1.00	1.00	1.00
252	2.00	1.00	1.00	1.00	2.00	1.00	2.00	2.00

	M7	M8	M9	M10	M11	M12	M13	M14
211	1.00	1.00	1.00	1.00	1.00	1.00	1.00	1.00
212	1.00	1.00	2.00	1.00	1.00	1.00	1.00	1.00
213	1.00	1.00	1.00	1.00	2.00	1.00	1.00	1.00
214	2.00	2.00	2.00	1.00	2.00	2.00	2.00	2.00
215	1.00	1.00	1.00	1.00	2.00	1.00	1.00	1.00
216	2.00	1.00	2.00	1.00	1.00	2.00	1.00	1.00
217	2.00	1.00	1.00	1.00	1.00	2.00	1.00	1.00
218	1.00	1.00	1.00	1.00	1.00	1.00	1.00	1.00
219	1.00	1.00	1.00	1.00	1.00	1.00	1.00	1.00
220	2.00	1.00	2.00	1.00	1.00	2.00	1.00	2.00
221	2.00	1.00	1.00	1.00	2.00	1.00	1.00	1.00
222	2.00	1.00	2.00	2.00	2.00	1.00	2.00	1.00
223	2.00	1.00	1.00	1.00	2.00	1.00	1.00	1.00
224	1.00	1.00	2.00	1.00	2.00	1.00	1.00	1.00
225	2.00	1.00	2.00	1.00	2.00	2.00	1.00	1.00
226	1.00	1.00	1.00	1.00	2.00	2.00	2.00	1.00
227	1.00	1.00	1.00	1.00	2.00	1.00	1.00	1.00
228	2.00	1.00	1.00	1.00	2.00	1.00	2.00	1.00
229	2.00	1.00	1.00	1.00	2.00	1.00	2.00	1.00
230	1.00	1.00	1.00	1.00	2.00	1.00	2.00	1.00
231	2.00	1.00	1.00	1.00	1.00	1.00	2.00	2.00
232	1.00	1.00	1.00	1.00	1.00	1.00	1.00	1.00
233	2.00	1.00	1.00	1.00	1.00	2.00	1.00	1.00
234	1.00	1.00	1.00	1.00	2.00	1.00	2.00	1.00
235	2.00	1.00	1.00	1.00	1.00	2.00	1.00	1.00
236	2.00	1.00	1.00	1.00	1.00	1.00	1.00	1.00
237	2.00	1.00	1.00	1.00	1.00	2.00	2.00	1.00
238	2.00	2.00	2.00	2.00	1.00	2.00	2.00	2.00
239	1.00	1.00	1.00	1.00	1.00	1.00	1.00	1.00
240	1.00	1.00	1.00	1.00	2.00	2.00	1.00	1.00
241	1.00	1.00	1.00	1.00	2.00	1.00	1.00	1.00
242	1.00	1.00	1.00	1.00	1.00	1.00	1.00	1.00
243	1.00	1.00	2.00	1.00	1.00	2.00	1.00	1.00
244	1.00	1.00	1.00	1.00	2.00	1.00	2.00	2.00
245	2.00	1.00	1.00	1.00	2.00	1.00	2.00	1.00
246	2.00	1.00	2.00	2.00	2.00	2.00	2.00	2.00
247	1.00	2.00	2.00	2.00	2.00	2.00	2.00	1.00
248	2.00	2.00	1.00	2.00	2.00	1.00	2.00	1.00
249	1.00	1.00	1.00	1.00	1.00	1.00	1.00	1.00
250	1.00	1.00	1.00	1.00	2.00	1.00	2.00	1.00
251	1.00	1.00	1.00	1.00	2.00	1.00	1.00	1.00
252	1.00	1.00	2.00	2.00	2.00	2.00	1.00	2.00

	M15	B1	B2	B3	B4	B5	B6	B7
211	1.00	1.00	1.00	2.00	1.00	2.00	2.00	2.00
212	1.00	2.00	1.00	1.00	2.00	1.00	1.00	1.00
213	1.00	2.00	2.00	2.00	1.00	2.00	2.00	2.00
214	2.00	2.00	2.00	2.00	1.00	2.00	2.00	2.00
215	1.00	2.00	2.00	2.00	2.00	1.00	2.00	2.00
216	1.00	1.00	2.00	2.00	1.00	1.00	1.00	1.00
217	1.00	1.00	2.00	2.00	2.00	2.00	2.00	2.00
218	1.00	1.00	1.00	1.00	1.00	2.00	2.00	2.00
219	1.00	2.00	2.00	1.00	1.00	2.00	2.00	2.00
220	1.00	2.00	1.00	2.00	1.00	2.00	1.00	1.00
221	1.00	2.00	2.00	2.00	1.00	2.00	2.00	1.00
222	2.00	1.00	1.00	2.00	1.00	2.00	2.00	2.00
223	1.00	2.00	2.00	2.00	2.00	2.00	2.00	2.00
224	1.00	1.00	2.00	2.00	2.00	1.00	2.00	1.00
225	2.00	1.00	1.00	1.00	1.00	1.00	1.00	1.00
226	1.00	1.00	1.00	1.00	1.00	1.00	2.00	2.00
227	1.00	1.00	1.00	2.00	1.00	1.00	1.00	1.00
228	1.00	2.00	1.00	1.00	1.00	2.00	2.00	2.00
229	2.00	1.00	1.00	2.00	1.00	1.00	2.00	2.00
230	2.00	1.00	1.00	1.00	1.00	2.00	1.00	1.00
231	2.00	1.00	1.00	1.00	1.00	1.00	1.00	1.00
232	1.00	2.00	2.00	2.00	1.00	2.00	1.00	1.00
233	1.00	2.00	1.00	2.00	1.00	1.00	2.00	2.00
234	1.00	1.00	2.00	2.00	1.00	1.00	2.00	2.00
235	1.00	2.00	1.00	1.00	1.00	1.00	2.00	1.00
236	1.00	1.00	1.00	2.00	1.00	2.00	2.00	2.00
237	2.00	1.00	1.00	1.00	1.00	2.00	1.00	1.00
238	2.00	2.00	1.00	1.00	1.00	1.00	2.00	2.00
239	1.00	1.00	2.00	2.00	1.00	2.00	2.00	2.00
240	2.00	2.00	2.00	2.00	1.00	2.00	2.00	2.00
241	1.00	1.00	2.00	2.00	1.00	1.00	2.00	2.00
242	1.00	2.00	2.00	2.00	2.00	2.00	2.00	2.00
243	2.00	1.00	1.00	1.00	2.00	2.00	2.00	2.00
244	2.00	1.00	1.00	2.00	1.00	2.00	1.00	1.00
245	1.00	2.00	2.00	1.00	1.00	2.00	2.00	2.00
246	2.00	1.00	1.00	1.00	2.00	1.00	2.00	2.00
247	2.00	1.00	2.00	2.00	1.00	1.00	2.00	2.00
248	1.00	2.00	1.00	1.00	1.00	1.00	1.00	1.00
249	1.00	2.00	1.00	1.00	1.00	2.00	1.00	1.00
250	1.00	2.00	1.00	1.00	1.00	2.00	2.00	2.00
251	1.00	2.00	2.00	2.00	1.00	2.00	2.00	2.00
252	1.00	1.00	1.00	2.00	2.00	1.00	1.00	1.00

	B8	B9	B10	B11	B12	B13	B14	W1
211	2.00	2.00	1.00	1.00	2.00	2.00	2.00	2.00
212	2.00	2.00	2.00	2.00	2.00	2.00	2.00	2.00
213	2.00	2.00	2.00	2.00	2.00	2.00	2.00	2.00
214	2.00	2.00	2.00	1.00	2.00	2.00	2.00	2.00
215	2.00	1.00	2.00	1.00	2.00	2.00	1.00	2.00
216	1.00	2.00	2.00	1.00	1.00	1.00	1.00	1.00
217	2.00	2.00	2.00	2.00	2.00	1.00	2.00	1.00
218	2.00	2.00	2.00	2.00	2.00	2.00	2.00	1.00
219	2.00	2.00	1.00	1.00	1.00	2.00	2.00	1.00
220	2.00	1.00	2.00	2.00	1.00	1.00	1.00	1.00
221	1.00	2.00	1.00	2.00	2.00	2.00	1.00	2.00
222	2.00	1.00	2.00	2.00	2.00	2.00	2.00	1.00
223	2.00	2.00	2.00	2.00	2.00	2.00	2.00	2.00
224	1.00	1.00	1.00	1.00	2.00	2.00	2.00	1.00
225	1.00	1.00	1.00	1.00	1.00	1.00	1.00	2.00
226	2.00	1.00	1.00	1.00	2.00	1.00	1.00	1.00
227	1.00	1.00	1.00	1.00	1.00	2.00	1.00	1.00
228	2.00	2.00	2.00	1.00	2.00	1.00	1.00	2.00
229	2.00	2.00	2.00	2.00	2.00	1.00	1.00	2.00
230	1.00	1.00	1.00	1.00	1.00	2.00	1.00	1.00
231	1.00	1.00	1.00	1.00	1.00	2.00	1.00	1.00
232	2.00	2.00	2.00	1.00	2.00	2.00	2.00	1.00
233	1.00	2.00	1.00	1.00	2.00	2.00	2.00	1.00
234	1.00	1.00	2.00	2.00	2.00	2.00	2.00	1.00
235	2.00	1.00	1.00	1.00	2.00	2.00	1.00	1.00
236	2.00	1.00	1.00	1.00	2.00	2.00	1.00	2.00
237	2.00	1.00	1.00	2.00	2.00	2.00	1.00	1.00
238	2.00	2.00	2.00	2.00	2.00	2.00	2.00	2.00
239	2.00	2.00	2.00	1.00	2.00	2.00	2.00	1.00
240	1.00	1.00	1.00	1.00	2.00	2.00	2.00	1.00
241	2.00	2.00	1.00	2.00	2.00	2.00	2.00	2.00
242	2.00	2.00	2.00	2.00	2.00	2.00	2.00	1.00
243	1.00	1.00	1.00	1.00	2.00	1.00	2.00	1.00
244	2.00	1.00	1.00	1.00	1.00	2.00	1.00	1.00
245	2.00	2.00	2.00	1.00	1.00	2.00	2.00	1.00
246	2.00	2.00	1.00	2.00	1.00	2.00	1.00	1.00
247	2.00	1.00	1.00	1.00	1.00	2.00	1.00	1.00
248	1.00	1.00	1.00	1.00	2.00	2.00	2.00	1.00
249	1.00	1.00	1.00	1.00	1.00	1.00	1.00	1.00
250	2.00	2.00	1.00	1.00	1.00	2.00	2.00	1.00
251	2.00	1.00	1.00	1.00	2.00	2.00	2.00	2.00
252	2.00	2.00	1.00	1.00	1.00	1.00	1.00	1.00

	W2	W3	W4	W5	W6	E1	V1	V2
211	2.00	1.00	1.00	1.00	2.00	1.00	2.00	2.00
212	2.00	1.00	1.00	1.00	2.00	1.00	2.00	1.00
213	1.00	1.00	1.00	1.00	2.00	1.00	2.00	2.00
214	2.00	1.00	2.00	2.00	2.00	1.00	2.00	2.00
215	2.00	1.00	1.00	1.00	2.00	1.00	2.00	2.00
216	1.00	1.00	2.00	1.00	2.00	1.00	2.00	2.00
217	2.00	1.00	1.00	1.00	2.00	1.00	2.00	2.00
218	1.00	1.00	1.00	1.00	1.00	1.00	2.00	2.00
219	1.00	1.00	1.00	1.00	2.00	1.00	2.00	2.00
220	1.00	2.00	1.00	1.00	2.00	1.00	2.00	1.00
221	2.00	1.00	1.00	1.00	2.00	1.00	2.00	2.00
222	2.00	1.00	2.00	2.00	2.00	1.00	2.00	2.00
223	2.00	1.00	2.00	1.00	1.00	1.00	2.00	2.00
224	2.00	1.00	2.00	1.00	2.00	1.00	1.00	2.00
225	2.00	1.00	2.00	1.00	2.00	1.00	2.00	1.00
226	1.00	1.00	1.00	1.00	2.00	1.00	2.00	2.00
227	1.00	1.00	1.00	1.00	1.00	1.00	2.00	2.00
228	2.00	1.00	1.00	1.00	2.00	1.00	2.00	2.00
229	1.00	1.00	2.00	1.00	2.00	1.00	2.00	2.00
230	1.00	1.00	2.00	1.00	1.00	1.00	2.00	2.00
231	2.00	1.00	2.00	1.00	1.00	1.00	2.00	2.00
232	1.00	1.00	1.00	1.00	2.00	1.00	2.00	2.00
233	1.00	1.00	1.00	1.00	2.00	1.00	2.00	2.00
234	2.00	1.00	2.00	1.00	2.00	1.00	2.00	2.00
235	2.00	1.00	2.00	1.00	2.00	1.00	2.00	2.00
236	2.00	1.00	1.00	1.00	1.00	1.00	2.00	2.00
237	1.00	1.00	1.00	1.00	2.00	1.00	2.00	2.00
238	2.00	2.00	2.00	2.00	2.00	1.00	2.00	2.00
239	1.00	1.00	1.00	1.00	2.00	1.00	2.00	2.00
240	1.00	1.00	1.00	1.00	1.00	1.00	2.00	2.00
241	1.00	1.00	1.00	2.00	1.00	1.00	2.00	2.00
242	1.00	1.00	1.00	1.00	1.00	1.00	1.00	1.00
243	1.00	2.00	1.00	1.00	2.00	1.00	1.00	1.00
244	2.00	1.00	2.00	2.00	2.00	1.00	2.00	2.00
245	1.00	1.00	1.00	1.00	1.00	1.00	1.00	2.00
246	2.00	1.00	1.00	1.00	1.00	1.00	2.00	2.00
247	1.00	1.00	2.00	1.00	1.00	1.00	2.00	2.00
248	1.00	2.00	1.00	2.00	1.00	1.00	1.00	2.00
249	1.00	2.00	2.00	2.00	2.00	1.00	1.00	1.00
250	1.00	1.00	1.00	1.00	2.00	1.00	1.00	2.00
251	2.00	2.00	2.00	2.00	2.00	1.00	2.00	2.00
252	1.00	1.00	2.00	1.00	1.00	1.00	1.00	1.00

	V3	V4	V5	V6	E2	E3	E4	C1
211	2.00	2.00	1.00	1.00	1.00	2.00	1.00	2.00
212	2.00	2.00	1.00	2.00	1.00	2.00	2.00	2.00
213	1.00	1.00	1.00	2.00	1.00	2.00	1.00	2.00
214	1.00	2.00	1.00	2.00	1.00	2.00	2.00	2.00
215	1.00	1.00	1.00	2.00	1.00	2.00	2.00	2.00
216	2.00	2.00	2.00	2.00	1.00	2.00	1.00	2.00
217	2.00	2.00	1.00	2.00	1.00	2.00	1.00	2.00
218	1.00	1.00	2.00	2.00	1.00	2.00	2.00	2.00
219	2.00	2.00	1.00	2.00	1.00	2.00	2.00	2.00
220	1.00	2.00	1.00	1.00	1.00	2.00	1.00	2.00
221	1.00	1.00	1.00	2.00	1.00	2.00	1.00	2.00
222	1.00	2.00	2.00	1.00	1.00	2.00	1.00	2.00
223	1.00	1.00	1.00	1.00	1.00	2.00	1.00	2.00
224	1.00	2.00	1.00	2.00	1.00	2.00	2.00	2.00
225	1.00	1.00	1.00	2.00	1.00	2.00	1.00	2.00
226	2.00	1.00	2.00	2.00	1.00	2.00	2.00	2.00
227	2.00	1.00	2.00	2.00	1.00	2.00	2.00	2.00
228	2.00	1.00	1.00	2.00	1.00	2.00	2.00	2.00
229	2.00	1.00	1.00	1.00	1.00	2.00	2.00	2.00
230	2.00	2.00	2.00	1.00	1.00	2.00	2.00	2.00
231	2.00	2.00	2.00	1.00	1.00	2.00	1.00	2.00
232	2.00	2.00	1.00	1.00	1.00	2.00	2.00	2.00
233	2.00	2.00	2.00	2.00	1.00	2.00	2.00	2.00
234	2.00	2.00	2.00	2.00	1.00	2.00	2.00	2.00
235	2.00	2.00	2.00	1.00	1.00	2.00	2.00	2.00
236	2.00	2.00	1.00	1.00	1.00	2.00	2.00	2.00
237	2.00	2.00	2.00	1.00	1.00	2.00	2.00	2.00
238	2.00	2.00	2.00	2.00	1.00	2.00	1.00	2.00
239	2.00	2.00	1.00	2.00	1.00	2.00	2.00	2.00
240	2.00	2.00	1.00	2.00	1.00	2.00	2.00	2.00
241	2.00	2.00	2.00	1.00	1.00	2.00	2.00	2.00
242	1.00	1.00	1.00	1.00	1.00	2.00	2.00	2.00
243	2.00	2.00	1.00	2.00	1.00	2.00	1.00	2.00
244	2.00	2.00	1.00	2.00	1.00	2.00	1.00	2.00
245	1.00	2.00	1.00	1.00	1.00	2.00	1.00	2.00
246	1.00	1.00	2.00	1.00	1.00	2.00	1.00	2.00
247	2.00	2.00	2.00	1.00	1.00	2.00	2.00	2.00
248	2.00	2.00	1.00	2.00	1.00	2.00	1.00	2.00
249	1.00	1.00	1.00	1.00	1.00	2.00	1.00	2.00
250	2.00	1.00	2.00	2.00	1.00	2.00	2.00	2.00
251	2.00	2.00	2.00	2.00	1.00	2.00	2.00	2.00
252	2.00	1.00	1.00	1.00	1.00	2.00	1.00	1.00

	C2	C3	C4	C5	C6	C7	filter_$
211	2.00	1.00	2.00	2.00	2.00	2.00	1
212	2.00	2.00	1.00	2.00	2.00	2.00	1
213	2.00	1.00	1.00	1.00	1.00	2.00	1
214	2.00	1.00	2.00	2.00	2.00	2.00	1
215	2.00	1.00	1.00	1.00	2.00	2.00	1
216	2.00	2.00	1.00	2.00	1.00	2.00	1
217	2.00	2.00	1.00	2.00	2.00	2.00	1
218	2.00	1.00	2.00	2.00	1.00	2.00	1
219	2.00	1.00	2.00	1.00	2.00	2.00	1
220	2.00	2.00	1.00	1.00	2.00	2.00	1
221	2.00	1.00	1.00	1.00	1.00	2.00	1
222	2.00	1.00	2.00	2.00	2.00	2.00	1
223	2.00	1.00	1.00	2.00	2.00	2.00	1
224	2.00	1.00	2.00	2.00	2.00	2.00	1
225	2.00	1.00	2.00	2.00	2.00	2.00	1
226	2.00	1.00	1.00	2.00	2.00	2.00	1
227	2.00	1.00	1.00	1.00	2.00	2.00	1
228	2.00	1.00	2.00	2.00	2.00	2.00	1
229	2.00	1.00	2.00	2.00	2.00	2.00	1
230	2.00	1.00	2.00	1.00	2.00	2.00	1
231	2.00	2.00	2.00	2.00	2.00	1.00	1
232	2.00	1.00	2.00	1.00	2.00	2.00	1
233	2.00	1.00	1.00	2.00	2.00	2.00	1
234	2.00	1.00	1.00	2.00	2.00	2.00	1
235	2.00	2.00	2.00	2.00	2.00	1.00	1
236	2.00	2.00	1.00	1.00	2.00	2.00	1
237	2.00	2.00	2.00	1.00	2.00	2.00	1
238	2.00	1.00	2.00	2.00	2.00	2.00	1
239	2.00	1.00	2.00	2.00	2.00	2.00	1
240	2.00	1.00	2.00	2.00	2.00	2.00	1
241	2.00	2.00	2.00	2.00	2.00	1.00	1
242	2.00	2.00	2.00	2.00	2.00	1.00	1
243	2.00	1.00	1.00	2.00	2.00	2.00	1
244	2.00	1.00	2.00	2.00	2.00	2.00	1
245	2.00	2.00	2.00	1.00	1.00	2.00	1
246	2.00	1.00	1.00	2.00	2.00	2.00	1
247	2.00	1.00	2.00	2.00	2.00	2.00	1
248	1.00	1.00	2.00	1.00	1.00	2.00	1
249	2.00	1.00	2.00	2.00	2.00	2.00	1
250	2.00	1.00	2.00	2.00	2.00	2.00	1
251	2.00	1.00	1.00	1.00	2.00	2.00	1
252	1.00	2.00	1.00	1.00	2.00	2.00	1

	SN	Group	Gender	Age	Work	Work2	Work3	Sector
253	332.00	1.00	1.00	21.00	4.00	1.00	1.00	1.00
254	333.00	1.00	1.00	22.00	4.00	1.00	1.00	1.00
255	335.00	1.00	1.00	22.00	5.00	1.00	1.00	1.00
256	336.00	1.00	1.00	19.00	2.00	1.00	1.00	1.00
257	338.00	1.00	2.00	22.00	5.00	1.00	1.00	1.00
258	339.00	1.00	2.00	22.00	5.00	1.00	1.00	1.00
259	341.00	1.00	2.00	22.00	4.00	1.00	1.00	1.00
260	342.00	1.00	2.00	23.00	5.00	1.00	1.00	1.00
261	343.00	1.00	2.00	21.00	5.00	1.00	1.00	1.00
262	344.00	1.00	2.00	23.00	6.00	1.00	1.00	1.00
263	345.00	1.00	2.00	22.00	5.00	1.00	1.00	1.00
264	346.00	1.00	2.00	22.00	5.00	1.00	1.00	1.00
265	347.00	1.00	1.00	30.00	8.00	2.00	2.00	1.00
266	348.00	1.00	2.00	25.00	7.00	2.00	2.00	1.00
267	350.00	1.00	2.00	22.00	5.00	1.00	1.00	1.00
268	351.00	1.00	2.00	20.00	3.00	1.00	1.00	1.00
269	352.00	1.00	1.00	20.00	3.00	1.00	1.00	1.00
270	353.00	1.00	2.00	30.00	8.00	2.00	2.00	2.00
271	354.00	1.00	2.00	27.00	8.00	2.00	2.00	2.00
272	357.00	1.00	1.00	23.00	4.00	1.00	1.00	1.00
273	358.00	1.00	2.00	40.00	10.00	3.00	2.00	1.00
274	359.00	1.00	2.00	29.00	3.00	1.00	1.00	1.00
275	360.00	1.00	2.00	24.00	6.00	1.00	1.00	1.00
276	361.00	1.00	2.00	28.00	8.00	2.00	2.00	1.00
277	363.00	1.00	1.00	22.00	3.00	1.00	1.00	1.00
278	364.00	1.00	2.00	23.00	5.00	1.00	1.00	1.00
279	365.00	1.00	1.00	26.00	7.00	2.00	2.00	1.00
280	366.00	1.00	2.00	25.00	7.00	2.00	2.00	2.00
281	368.00	1.00	1.00	26.00	8.00	2.00	2.00	2.00
282	370.00	1.00	1.00	24.00	6.00	1.00	1.00	1.00
283	371.00	1.00	2.00	23.00	6.00	1.00	1.00	1.00
284	373.00	1.00	2.00	19.00	2.00	1.00	1.00	1.00
285	374.00	1.00	2.00	23.00	6.00	1.00	1.00	1.00
286	375.00	1.00	1.00	25.00	8.00	2.00	2.00	1.00
287	376.00	1.00	2.00	22.00	4.00	1.00	1.00	1.00
288	377.00	1.00	2.00	22.00	4.00	1.00	1.00	1.00
289	382.00	1.00	2.00	20.00	2.00	1.00	1.00	1.00
290	383.00	1.00	2.00	22.00	5.00	1.00	1.00	1.00
291	386.00	1.00	1.00	26.00	7.00	2.00	2.00	1.00
292	387.00	1.00	2.00	24.00	7.00	2.00	2.00	1.00
293	388.00	1.00	1.00	23.00	6.00	1.00	1.00	1.00
294	390.00	1.00	1.00	23.00	6.00	1.00	1.00	1.00

	Region	Nationality	M1	M2	M3	M4	M5	M6
253	5.00	1.00	1.00	2.00	1.00	1.00	1.00	2.00
254	5.00	1.00	1.00	1.00	2.00	1.00	1.00	1.00
255	5.00	1.00	1.00	1.00	1.00	1.00	1.00	1.00
256	5.00	1.00	2.00	2.00	2.00	1.00	1.00	2.00
257	5.00	1.00	1.00	1.00	1.00	1.00	1.00	1.00
258	2.00	1.00	1.00	1.00	1.00	1.00	2.00	1.00
259	5.00	1.00	1.00	1.00	1.00	1.00	1.00	1.00
260	5.00	1.00	1.00	1.00	2.00	2.00	1.00	1.00
261	5.00	1.00	1.00	1.00	1.00	2.00	2.00	1.00
262	5.00	1.00	1.00	1.00	1.00	1.00	1.00	1.00
263	5.00	1.00	1.00	1.00	1.00	1.00	1.00	1.00
264	5.00	1.00	1.00	1.00	1.00	1.00	1.00	1.00
265	4.00	1.00	2.00	2.00	2.00	1.00	1.00	1.00
266	5.00	1.00	1.00	1.00	1.00	1.00	1.00	1.00
267	2.00	1.00	2.00	1.00	2.00	1.00	1.00	1.00
268	5.00	1.00	1.00	1.00	1.00	1.00	1.00	1.00
269	5.00	1.00	2.00	2.00	2.00	1.00	1.00	1.00
270	1.00	2.00	1.00	1.00	2.00	1.00	1.00	2.00
271	2.00	1.00	1.00	1.00	1.00	1.00	1.00	2.00
272	5.00	1.00	2.00	2.00	2.00	2.00	2.00	2.00
273	1.00	1.00	1.00	1.00	1.00	1.00	1.00	2.00
274	3.00	1.00	2.00	1.00	1.00	2.00	1.00	1.00
275	4.00	1.00	1.00	2.00	2.00	1.00	1.00	1.00
276	5.00	1.00	2.00	2.00	1.00	2.00	2.00	1.00
277	5.00	1.00	2.00	2.00	2.00	1.00	2.00	2.00
278	5.00	1.00	1.00	1.00	1.00	1.00	1.00	1.00
279	3.00	1.00	1.00	1.00	2.00	1.00	1.00	2.00
280	3.00	1.00	1.00	1.00	1.00	2.00	1.00	1.00
281	2.00	1.00	1.00	1.00	1.00	1.00	2.00	1.00
282	5.00	1.00	1.00	1.00	1.00	1.00	1.00	1.00
283	5.00	1.00	1.00	1.00	1.00	1.00	1.00	1.00
284	5.00	1.00	1.00	2.00	2.00	2.00	2.00	1.00
285	2.00	1.00	1.00	1.00	1.00	1.00	1.00	1.00
286	3.00	1.00	1.00	2.00	1.00	2.00	1.00	2.00
287	2.00	1.00	1.00	1.00	2.00	1.00	1.00	1.00
288	4.00	1.00	1.00	1.00	1.00	1.00	1.00	1.00
289	2.00	1.00	1.00	1.00	1.00	1.00	2.00	1.00
290	2.00	1.00	1.00	1.00	1.00	1.00	1.00	1.00
291	3.00	1.00	1.00	1.00	1.00	1.00	1.00	1.00
292	4.00	1.00	1.00	1.00	2.00	1.00	1.00	2.00
293	5.00	1.00	1.00	1.00	1.00	1.00	1.00	1.00
294	5.00	1.00	2.00	2.00	2.00	2.00	2.00	1.00

	M7	M8	M9	M10	M11	M12	M13	M14
253	1.00	1.00	1.00	1.00	1.00	1.00	2.00	1.00
254	1.00	1.00	1.00	1.00	2.00	2.00	1.00	1.00
255	1.00	1.00	2.00	1.00	1.00	2.00	1.00	1.00
256	1.00	2.00	1.00	1.00	1.00	2.00	2.00	1.00
257	2.00	1.00	1.00	1.00	2.00	2.00	1.00	1.00
258	1.00	1.00	1.00	1.00	2.00	1.00	2.00	1.00
259	1.00	1.00	1.00	1.00	2.00	1.00	1.00	1.00
260	2.00	1.00	1.00	1.00	2.00	1.00	2.00	1.00
261	2.00	1.00	2.00	1.00	2.00	1.00	1.00	2.00
262	2.00	2.00	2.00	2.00	2.00	1.00	1.00	1.00
263	1.00	1.00	1.00	1.00	1.00	1.00	1.00	1.00
264	2.00	1.00	1.00	2.00	2.00	1.00	1.00	1.00
265	2.00	1.00	2.00	1.00	1.00	2.00	2.00	1.00
266	1.00	1.00	1.00	1.00	1.00	2.00	1.00	1.00
267	2.00	1.00	1.00	2.00	1.00	1.00	2.00	1.00
268	1.00	1.00	1.00	1.00	1.00	1.00	1.00	1.00
269	1.00	1.00	1.00	1.00	1.00	1.00	1.00	1.00
270	2.00	1.00	2.00	1.00	1.00	1.00	1.00	1.00
271	2.00	1.00	2.00	2.00	1.00	1.00	2.00	1.00
272	2.00	1.00	2.00	2.00	2.00	2.00	2.00	2.00
273	1.00	1.00	1.00	1.00	1.00	1.00	1.00	2.00
274	1.00	1.00	1.00	1.00	2.00	1.00	2.00	1.00
275	1.00	1.00	1.00	2.00	2.00	1.00	2.00	1.00
276	2.00	1.00	2.00	2.00	2.00	2.00	2.00	2.00
277	1.00	1.00	2.00	1.00	2.00	2.00	1.00	1.00
278	1.00	1.00	1.00	1.00	2.00	2.00	1.00	1.00
279	1.00	1.00	1.00	1.00	2.00	1.00	1.00	1.00
280	2.00	1.00	1.00	2.00	2.00	1.00	1.00	1.00
281	1.00	1.00	1.00	1.00	2.00	1.00	2.00	1.00
282	1.00	1.00	1.00	1.00	2.00	1.00	1.00	1.00
283	1.00	1.00	1.00	1.00	1.00	1.00	1.00	1.00
284	2.00	1.00	1.00	2.00	1.00	2.00	1.00	1.00
285	1.00	1.00	1.00	1.00	1.00	1.00	1.00	1.00
286	1.00	1.00	2.00	1.00	1.00	2.00	1.00	1.00
287	2.00	2.00	2.00	2.00	2.00	1.00	2.00	1.00
288	1.00	1.00	1.00	1.00	1.00	1.00	1.00	1.00
289	2.00	1.00	2.00	2.00	2.00	2.00	2.00	1.00
290	1.00	1.00	1.00	1.00	1.00	1.00	1.00	1.00
291	1.00	1.00	1.00	1.00	1.00	1.00	1.00	1.00
292	2.00	1.00	1.00	2.00	2.00	2.00	2.00	2.00
293	1.00	1.00	1.00	1.00	2.00	1.00	1.00	1.00
294	2.00	2.00	2.00	1.00	2.00	1.00	2.00	1.00

	M15	B1	B2	B3	B4	B5	B6	B7
253	1.00	1.00	1.00	1.00	1.00	2.00	1.00	1.00
254	2.00	2.00	1.00	1.00	1.00	1.00	1.00	1.00
255	1.00	2.00	2.00	2.00	2.00	2.00	2.00	2.00
256	1.00	1.00	2.00	2.00	2.00	1.00	1.00	1.00
257	1.00	2.00	2.00	1.00	2.00	1.00	1.00	1.00
258	1.00	2.00	2.00	2.00	1.00	2.00	2.00	2.00
259	1.00	1.00	2.00	2.00	1.00	2.00	1.00	1.00
260	2.00	1.00	1.00	1.00	1.00	1.00	1.00	1.00
261	1.00	1.00	1.00	1.00	1.00	1.00	1.00	1.00
262	1.00	2.00	1.00	2.00	1.00	1.00	1.00	1.00
263	1.00	2.00	2.00	2.00	1.00	1.00	1.00	1.00
264	1.00	2.00	2.00	1.00	1.00	1.00	1.00	1.00
265	2.00	2.00	1.00	1.00	1.00	1.00	2.00	1.00
266	1.00	1.00	1.00	1.00	1.00	1.00	1.00	1.00
267	2.00	1.00	1.00	2.00	1.00	2.00	2.00	2.00
268	1.00	1.00	2.00	2.00	1.00	2.00	2.00	2.00
269	1.00	1.00	1.00	2.00	1.00	2.00	2.00	1.00
270	1.00	1.00	2.00	2.00	1.00	2.00	2.00	2.00
271	1.00	2.00	1.00	1.00	1.00	2.00	2.00	2.00
272	2.00	2.00	1.00	1.00	1.00	1.00	1.00	1.00
273	1.00	1.00	1.00	1.00	2.00	2.00	2.00	2.00
274	1.00	1.00	1.00	1.00	1.00	1.00	2.00	1.00
275	1.00	2.00	1.00	2.00	2.00	2.00	2.00	2.00
276	1.00	2.00	1.00	1.00	1.00	1.00	2.00	2.00
277	2.00	2.00	1.00	1.00	1.00	1.00	2.00	2.00
278	1.00	1.00	2.00	2.00	2.00	2.00	1.00	1.00
279	2.00	2.00	1.00	1.00	1.00	1.00	1.00	1.00
280	1.00	1.00	1.00	1.00	1.00	1.00	1.00	1.00
281	1.00	1.00	2.00	1.00	1.00	2.00	1.00	1.00
282	1.00	1.00	2.00	2.00	2.00	2.00	2.00	1.00
283	1.00	1.00	1.00	1.00	1.00	1.00	1.00	1.00
284	1.00	1.00	1.00	2.00	1.00	2.00	1.00	1.00
285	1.00	2.00	2.00	2.00	2.00	1.00	2.00	2.00
286	2.00	2.00	1.00	1.00	2.00	1.00	2.00	2.00
287	1.00	2.00	2.00	2.00	1.00	2.00	2.00	2.00
288	1.00	1.00	1.00	1.00	1.00	1.00	1.00	1.00
289	1.00	1.00	1.00	1.00	1.00	2.00	2.00	2.00
290	1.00	2.00	2.00	2.00	2.00	2.00	2.00	2.00
291	1.00	1.00	1.00	1.00	1.00	1.00	1.00	1.00
292	1.00	1.00	1.00	2.00	1.00	1.00	2.00	2.00
293	1.00	1.00	2.00	2.00	1.00	2.00	2.00	2.00
294	2.00	2.00	2.00	2.00	1.00	1.00	2.00	2.00

	B8	B9	B10	B11	B12	B13	B14	W1
253	1.00	1.00	1.00	1.00	1.00	1.00	1.00	2.00
254	1.00	1.00	1.00	1.00	2.00	2.00	2.00	1.00
255	2.00	2.00	2.00	2.00	2.00	2.00	2.00	1.00
256	2.00	2.00	1.00	2.00	1.00	2.00	2.00	2.00
257	1.00	1.00	1.00	1.00	2.00	2.00	2.00	1.00
258	2.00	1.00	1.00	2.00	2.00	2.00	2.00	2.00
259	2.00	1.00	1.00	2.00	1.00	2.00	2.00	1.00
260	2.00	1.00	1.00	1.00	2.00	1.00	2.00	2.00
261	1.00	1.00	1.00	1.00	1.00	1.00	2.00	1.00
262	1.00	2.00	1.00	1.00	1.00	1.00	2.00	1.00
263	2.00	1.00	1.00	1.00	1.00	1.00	1.00	1.00
264	2.00	2.00	2.00	1.00	2.00	2.00	2.00	1.00
265	2.00	2.00	1.00	1.00	1.00	2.00	1.00	2.00
266	1.00	1.00	1.00	1.00	1.00	1.00	1.00	1.00
267	2.00	2.00	1.00	1.00	2.00	2.00	2.00	1.00
268	2.00	1.00	1.00	1.00	2.00	1.00	1.00	1.00
269	1.00	2.00	2.00	1.00	2.00	2.00	1.00	1.00
270	1.00	1.00	2.00	2.00	2.00	1.00	2.00	1.00
271	2.00	2.00	1.00	1.00	2.00	2.00	1.00	2.00
272	2.00	1.00	2.00	2.00	2.00	2.00	2.00	2.00
273	2.00	2.00	1.00	2.00	2.00	2.00	2.00	2.00
274	1.00	2.00	1.00	1.00	1.00	2.00	1.00	1.00
275	2.00	2.00	2.00	2.00	2.00	2.00	2.00	1.00
276	2.00	1.00	2.00	2.00	2.00	2.00	2.00	2.00
277	1.00	1.00	1.00	1.00	2.00	2.00	2.00	2.00
278	1.00	1.00	1.00	1.00	1.00	1.00	2.00	1.00
279	1.00	1.00	1.00	1.00	1.00	2.00	1.00	1.00
280	2.00	1.00	1.00	1.00	1.00	2.00	1.00	2.00
281	2.00	1.00	1.00	1.00	2.00	2.00	1.00	1.00
282	2.00	2.00	2.00	1.00	2.00	2.00	2.00	1.00
283	1.00	1.00	1.00	1.00	1.00	1.00	1.00	1.00
284	2.00	1.00	2.00	1.00	2.00	1.00	1.00	2.00
285	1.00	2.00	2.00	2.00	2.00	2.00	1.00	1.00
286	2.00	2.00	1.00	1.00	2.00	2.00	1.00	1.00
287	2.00	2.00	1.00	1.00	2.00	2.00	2.00	1.00
288	1.00	1.00	1.00	1.00	1.00	1.00	1.00	1.00
289	2.00	2.00	1.00	2.00	1.00	2.00	1.00	1.00
290	2.00	2.00	2.00	2.00	2.00	2.00	2.00	1.00
291	1.00	1.00	1.00	1.00	1.00	1.00	1.00	1.00
292	2.00	1.00	1.00	2.00	2.00	2.00	1.00	2.00
293	2.00	2.00	2.00	1.00	2.00	2.00	2.00	1.00
294	2.00	2.00	2.00	2.00	2.00	2.00	2.00	2.00

	W2	W3	W4	W5	W6	E1	V1	V2
253	2.00	1.00	1.00	1.00	1.00	1.00	2.00	2.00
254	1.00	1.00	1.00	1.00	1.00	1.00	1.00	1.00
255	1.00	1.00	1.00	1.00	2.00	1.00	2.00	2.00
256	1.00	2.00	2.00	1.00	2.00	1.00	1.00	2.00
257	1.00	1.00	1.00	1.00	1.00	1.00	2.00	2.00
258	2.00	2.00	2.00	2.00	2.00	1.00	2.00	2.00
259	1.00	1.00	1.00	1.00	2.00	1.00	2.00	2.00
260	2.00	1.00	2.00	1.00	2.00	1.00	2.00	2.00
261	1.00	1.00	1.00	1.00	1.00	1.00	1.00	2.00
262	1.00	1.00	1.00	1.00	1.00	1.00	1.00	1.00
263	1.00	1.00	1.00	1.00	1.00	1.00	2.00	2.00
264	1.00	1.00	2.00	2.00	2.00	1.00	2.00	2.00
265	2.00	1.00	1.00	1.00	2.00	1.00	2.00	2.00
266	1.00	2.00	2.00	1.00	2.00	1.00	2.00	2.00
267	1.00	1.00	1.00	1.00	1.00	1.00	2.00	2.00
268	1.00	1.00	1.00	1.00	1.00	1.00	2.00	2.00
269	1.00	1.00	1.00	1.00	1.00	1.00	1.00	1.00
270	2.00	1.00	2.00	1.00	2.00	1.00	2.00	2.00
271	2.00	1.00	1.00	1.00	2.00	1.00	2.00	2.00
272	2.00	2.00	2.00	2.00	2.00	1.00	2.00	2.00
273	2.00	1.00	1.00	1.00	1.00	1.00	2.00	2.00
274	2.00	1.00	2.00	1.00	2.00	1.00	2.00	2.00
275	2.00	1.00	2.00	1.00	1.00	1.00	2.00	2.00
276	2.00	2.00	1.00	1.00	2.00	1.00	2.00	2.00
277	2.00	1.00	2.00	2.00	2.00	1.00	2.00	2.00
278	1.00	1.00	1.00	2.00	2.00	1.00	2.00	2.00
279	1.00	1.00	1.00	2.00	2.00	1.00	2.00	2.00
280	1.00	1.00	1.00	1.00	1.00	1.00	2.00	2.00
281	1.00	1.00	1.00	1.00	2.00	1.00	2.00	2.00
282	2.00	1.00	1.00	2.00	2.00	1.00	2.00	2.00
283	1.00	1.00	1.00	1.00	1.00	1.00	1.00	1.00
284	1.00	2.00	1.00	1.00	2.00	1.00	1.00	2.00
285	1.00	1.00	1.00	1.00	1.00	1.00	2.00	2.00
286	1.00	1.00	2.00	1.00	2.00	1.00	2.00	2.00
287	2.00	1.00	2.00	1.00	2.00	1.00	2.00	2.00
288	1.00	2.00	1.00	2.00	2.00	1.00	2.00	2.00
289	1.00	1.00	1.00	1.00	1.00	1.00	2.00	2.00
290	1.00	1.00	1.00	1.00	1.00	1.00	1.00	1.00
291	1.00	1.00	1.00	1.00	1.00	1.00	1.00	1.00
292	2.00	2.00	2.00	2.00	1.00	1.00	2.00	2.00
293	2.00	1.00	1.00	1.00	2.00	1.00	2.00	2.00
294	2.00	1.00	1.00	2.00	2.00	1.00	2.00	2.00

	V3	V4	V5	V6	E2	E3	E4	C1
253	1.00	1.00	2.00	2.00	1.00	2.00	2.00	2.00
254	1.00	1.00	1.00	1.00	1.00	2.00	1.00	2.00
255	2.00	2.00	2.00	1.00	1.00	2.00	2.00	2.00
256	1.00	1.00	2.00	2.00	1.00	2.00	2.00	1.00
257	1.00	1.00	2.00	2.00	1.00	2.00	2.00	2.00
258	2.00	1.00	2.00	1.00	1.00	2.00	1.00	2.00
259	2.00	2.00	1.00	2.00	1.00	2.00	2.00	2.00
260	2.00	2.00	2.00	1.00	1.00	2.00	2.00	2.00
261	1.00	1.00	1.00	1.00	1.00	2.00	2.00	2.00
262	1.00	1.00	1.00	1.00	1.00	1.00	1.00	1.00
263	1.00	1.00	2.00	2.00	1.00	2.00	2.00	2.00
264	2.00	2.00	2.00	1.00	1.00	2.00	1.00	2.00
265	1.00	2.00	1.00	2.00	1.00	2.00	2.00	2.00
266	1.00	2.00	2.00	2.00	1.00	2.00	2.00	2.00
267	2.00	1.00	1.00	2.00	1.00	2.00	2.00	1.00
268	2.00	2.00	2.00	1.00	1.00	2.00	2.00	2.00
269	1.00	1.00	1.00	1.00	1.00	2.00	2.00	2.00
270	1.00	2.00	1.00	2.00	1.00	2.00	2.00	2.00
271	1.00	1.00	1.00	2.00	1.00	2.00	2.00	2.00
272	2.00	2.00	2.00	2.00	1.00	2.00	1.00	2.00
273	1.00	1.00	1.00	2.00	1.00	2.00	2.00	1.00
274	2.00	2.00	2.00	2.00	1.00	2.00	1.00	2.00
275	1.00	2.00	1.00	1.00	1.00	2.00	1.00	2.00
276	2.00	2.00	1.00	2.00	1.00	2.00	1.00	2.00
277	2.00	2.00	1.00	2.00	1.00	2.00	1.00	2.00
278	2.00	2.00	2.00	1.00	1.00	2.00	1.00	2.00
279	2.00	2.00	2.00	2.00	1.00	2.00	1.00	2.00
280	1.00	2.00	2.00	2.00	1.00	2.00	2.00	2.00
281	1.00	1.00	1.00	2.00	1.00	2.00	1.00	2.00
282	1.00	1.00	2.00	2.00	1.00	2.00	2.00	2.00
283	1.00	1.00	1.00	1.00	1.00	2.00	1.00	2.00
284	1.00	1.00	1.00	2.00	1.00	2.00	1.00	1.00
285	1.00	1.00	1.00	2.00	1.00	2.00	2.00	2.00
286	2.00	1.00	2.00	2.00	1.00	2.00	1.00	2.00
287	1.00	2.00	2.00	1.00	1.00	2.00	1.00	2.00
288	1.00	2.00	2.00	2.00	1.00	2.00	2.00	2.00
289	2.00	1.00	2.00	2.00	1.00	2.00	1.00	2.00
290	2.00	2.00	2.00	2.00	1.00	2.00	2.00	2.00
291	1.00	1.00	1.00	1.00	1.00	2.00	1.00	1.00
292	2.00	2.00	2.00	1.00	1.00	2.00	1.00	2.00
293	1.00	2.00	1.00	2.00	1.00	2.00	1.00	2.00
294	1.00	2.00	2.00	2.00	1.00	2.00	2.00	2.00

	C2	C3	C4	C5	C6	C7	filter_$
253	2.00	2.00	1.00	1.00	1.00	2.00	1
254	2.00	1.00	1.00	2.00	2.00	2.00	1
255	2.00	1.00	2.00	2.00	2.00	2.00	1
256	1.00	2.00	1.00	2.00	2.00	2.00	1
257	2.00	2.00	2.00	1.00	1.00	2.00	1
258	2.00	1.00	2.00	1.00	1.00	2.00	1
259	2.00	1.00	2.00	2.00	2.00	2.00	1
260	2.00	1.00	2.00	2.00	2.00	2.00	1
261	2.00	2.00	2.00	2.00	2.00	2.00	1
262	1.00	1.00	1.00	1.00	1.00	2.00	1
263	2.00	1.00	2.00	2.00	2.00	2.00	1
264	2.00	1.00	2.00	1.00	2.00	2.00	1
265	2.00	1.00	1.00	2.00	2.00	2.00	1
266	2.00	1.00	1.00	2.00	2.00	2.00	1
267	2.00	1.00	2.00	1.00	2.00	2.00	1
268	2.00	1.00	2.00	1.00	2.00	2.00	1
269	2.00	1.00	2.00	1.00	1.00	2.00	1
270	2.00	1.00	2.00	2.00	2.00	2.00	1
271	2.00	1.00	2.00	2.00	2.00	2.00	1
272	2.00	2.00	2.00	2.00	2.00	2.00	1
273	2.00	1.00	2.00	2.00	2.00	2.00	1
274	2.00	1.00	1.00	2.00	2.00	2.00	1
275	2.00	1.00	1.00	2.00	2.00	2.00	1
276	2.00	1.00	2.00	1.00	2.00	2.00	1
277	2.00	2.00	1.00	2.00	2.00	2.00	1
278	2.00	1.00	2.00	2.00	2.00	2.00	1
279	2.00	1.00	2.00	2.00	2.00	2.00	1
280	2.00	2.00	2.00	2.00	2.00	2.00	1
281	2.00	1.00	2.00	1.00	2.00	2.00	1
282	2.00	1.00	2.00	1.00	1.00	2.00	1
283	2.00	1.00	2.00	2.00	2.00	2.00	1
284	1.00	1.00	1.00	2.00	1.00	2.00	1
285	1.00	1.00	1.00	2.00	2.00	2.00	1
286	2.00	1.00	1.00	2.00	2.00	2.00	1
287	2.00	1.00	2.00	2.00	2.00	2.00	1
288	2.00	1.00	1.00	2.00	2.00	2.00	1
289	2.00	1.00	1.00	1.00	2.00	2.00	1
290	2.00	2.00	1.00	2.00	2.00	2.00	1
291	1.00	1.00	1.00	1.00	1.00	2.00	1
292	2.00	1.00	2.00	2.00	2.00	2.00	1
293	2.00	1.00	2.00	1.00	2.00	2.00	1
294	2.00	1.00	2.00	2.00	2.00	2.00	1

	SN	Group	Gender	Age	Work	Work2	Work3	Sector
295	391.00	1.00	2.00	24.00	7.00	2.00	2.00	1.00
296	393.00	1.00	2.00	21.00	3.00	1.00	1.00	1.00
297	395.00	1.00	2.00	24.00	7.00	2.00	2.00	1.00
298	398.00	1.00	1.00	26.00	8.00	2.00	2.00	2.00
299	399.00	1.00	1.00	22.00	3.00	1.00	1.00	1.00
300	404.00	1.00	1.00	25.00	7.00	2.00	2.00	1.00
301	405.00	1.00	2.00	26.00	8.00	2.00	2.00	2.00
302	406.00	1.00	1.00	21.00	2.00	1.00	1.00	1.00
303	407.00	1.00	2.00	25.00	8.00	2.00	2.00	1.00
304	408.00	1.00	1.00	29.00	8.00	2.00	2.00	2.00
305	409.00	1.00	2.00	25.00	8.00	2.00	2.00	1.00
306	410.00	1.00	2.00	31.00	8.00	2.00	2.00	2.00
307	412.00	1.00	1.00	21.00	4.00	1.00	1.00	1.00
308	413.00	1.00	1.00	22.00	4.00	1.00	1.00	1.00
309	414.00	1.00	1.00	22.00	5.00	1.00	1.00	1.00
310	415.00	1.00	1.00	22.00	5.00	1.00	1.00	1.00
311	416.00	1.00	1.00	21.00	4.00	1.00	1.00	1.00
312	417.00	1.00	1.00	22.00	5.00	1.00	1.00	1.00
313	418.00	1.00	1.00	23.00	5.00	1.00	1.00	1.00
314	419.00	1.00	1.00	24.00	7.00	2.00	2.00	1.00
315	420.00	1.00	1.00	24.00	7.00	2.00	2.00	1.00
316	421.00	1.00	1.00	24.00	7.00	2.00	2.00	1.00
317	422.00	1.00	1.00	24.00	7.00	2.00	2.00	1.00
318	423.00	1.00	1.00	20.00	3.00	1.00	1.00	1.00
319	424.00	1.00	1.00	22.00	3.00	1.00	1.00	1.00
320	425.00	1.00	1.00	23.00	6.00	1.00	1.00	1.00
321	429.00	1.00	2.00	23.00	5.00	1.00	1.00	1.00
322	430.00	1.00	2.00	24.00	7.00	2.00	2.00	2.00
323	431.00	1.00	1.00	22.00	5.00	1.00	1.00	2.00
324	432.00	1.00	2.00	23.00	5.00	1.00	1.00	2.00
325	433.00	1.00	1.00	23.00	5.00	1.00	1.00	2.00
326	434.00	1.00	1.00	24.00	6.00	1.00	1.00	1.00
327	435.00	1.00	2.00	22.00	5.00	1.00	1.00	2.00
328	436.00	1.00	2.00	25.00	3.00	1.00	1.00	1.00
329	437.00	1.00	1.00	25.00	7.00	2.00	2.00	1.00
330	439.00	1.00	1.00	27.00	7.00	2.00	2.00	1.00
331	440.00	1.00	1.00	26.00	8.00	2.00	2.00	2.00
332	441.00	1.00	1.00	24.00	8.00	2.00	2.00	2.00
333	442.00	1.00	1.00	20.00	2.00	1.00	1.00	1.00
334	443.00	1.00	2.00	23.00	6.00	1.00	1.00	1.00
335	444.00	1.00	2.00	24.00	6.00	1.00	1.00	1.00
336	445.00	1.00	2.00	22.00	3.00	1.00	1.00	1.00

	Region	Nationality	M1	M2	M3	M4	M5	M6
295	2.00	1.00	1.00	1.00	1.00	2.00	1.00	2.00
296	2.00	1.00	1.00	1.00	1.00	1.00	2.00	1.00
297	2.00	1.00	1.00	1.00	2.00	1.00	1.00	1.00
298	2.00	1.00	2.00	1.00	2.00	1.00	1.00	2.00
299	1.00	1.00	1.00	1.00	1.00	1.00	2.00	1.00
300	2.00	1.00	1.00	1.00	1.00	1.00	1.00	1.00
301	1.00	1.00	2.00	2.00	2.00	1.00	1.00	1.00
302	2.00	1.00	1.00	1.00	1.00	1.00	1.00	1.00
303	2.00	1.00	1.00	1.00	1.00	1.00	1.00	2.00
304	2.00	1.00	1.00	1.00	1.00	1.00	1.00	1.00
305	2.00	1.00	1.00	1.00	2.00	1.00	1.00	1.00
306	2.00	1.00	1.00	1.00	1.00	1.00	2.00	2.00
307	2.00	1.00	2.00	2.00	2.00	1.00	1.00	1.00
308	2.00	1.00	1.00	1.00	2.00	1.00	2.00	1.00
309	5.00	1.00	1.00	1.00	2.00	1.00	1.00	1.00
310	2.00	1.00	1.00	1.00	2.00	1.00	1.00	1.00
311	2.00	1.00	1.00	2.00	2.00	1.00	1.00	1.00
312	2.00	1.00	1.00	1.00	1.00	1.00	2.00	1.00
313	2.00	1.00	1.00	1.00	1.00	1.00	1.00	1.00
314	2.00	1.00	1.00	1.00	2.00	2.00	1.00	2.00
315	2.00	1.00	1.00	1.00	2.00	1.00	1.00	1.00
316	2.00	1.00	1.00	2.00	2.00	1.00	1.00	2.00
317	2.00	1.00	1.00	1.00	1.00	1.00	1.00	1.00
318	2.00	1.00	1.00	1.00	2.00	1.00	1.00	1.00
319	2.00	1.00	1.00	1.00	2.00	1.00	1.00	1.00
320	2.00	1.00	2.00	1.00	2.00	2.00	1.00	1.00
321	2.00	1.00	1.00	1.00	2.00	1.00	1.00	1.00
322	2.00	1.00	1.00	1.00	1.00	2.00	2.00	2.00
323	2.00	2.00	1.00	1.00	2.00	1.00	2.00	1.00
324	2.00	2.00	1.00	1.00	1.00	1.00	1.00	1.00
325	2.00	2.00	2.00	2.00	2.00	1.00	1.00	1.00
326	3.00	1.00	2.00	2.00	2.00	1.00	1.00	1.00
327	2.00	2.00	1.00	1.00	1.00	1.00	1.00	1.00
328	2.00	1.00	1.00	1.00	2.00	1.00	1.00	2.00
329	2.00	1.00	2.00	1.00	2.00	1.00	1.00	1.00
330	3.00	1.00	2.00	2.00	2.00	2.00	2.00	2.00
331	1.00	1.00	2.00	2.00	2.00	2.00	1.00	1.00
332	1.00	1.00	1.00	1.00	1.00	1.00	1.00	1.00
333	3.00	1.00	1.00	1.00	2.00	1.00	1.00	1.00
334	2.00	1.00	2.00	1.00	2.00	1.00	1.00	1.00
335	2.00	1.00	1.00	1.00	2.00	1.00	1.00	1.00
336	5.00	1.00	1.00	1.00	2.00	1.00	2.00	2.00

	M7	M8	M9	M10	M11	M12	M13	M14
295	1.00	1.00	1.00	1.00	1.00	1.00	1.00	1.00
296	1.00	1.00	1.00	1.00	2.00	1.00	2.00	1.00
297	1.00	1.00	1.00	1.00	1.00	2.00	1.00	1.00
298	2.00	1.00	2.00	2.00	2.00	1.00	2.00	1.00
299	2.00	1.00	1.00	1.00	1.00	1.00	2.00	2.00
300	1.00	1.00	1.00	1.00	1.00	1.00	1.00	1.00
301	1.00	1.00	1.00	2.00	2.00	2.00	1.00	1.00
302	1.00	1.00	1.00	1.00	2.00	1.00	2.00	1.00
303	2.00	2.00	2.00	1.00	2.00	1.00	2.00	1.00
304	1.00	1.00	1.00	1.00	2.00	1.00	1.00	1.00
305	1.00	1.00	1.00	1.00	2.00	1.00	2.00	1.00
306	1.00	1.00	1.00	2.00	2.00	1.00	2.00	1.00
307	2.00	1.00	1.00	2.00	2.00	1.00	2.00	1.00
308	1.00	1.00	1.00	1.00	2.00	1.00	1.00	1.00
309	1.00	1.00	2.00	1.00	1.00	1.00	2.00	1.00
310	1.00	1.00	1.00	1.00	2.00	1.00	1.00	1.00
311	2.00	1.00	1.00	1.00	1.00	1.00	1.00	1.00
312	1.00	1.00	2.00	1.00	2.00	1.00	2.00	1.00
313	1.00	1.00	1.00	1.00	1.00	1.00	1.00	1.00
314	2.00	2.00	1.00	1.00	2.00	1.00	2.00	1.00
315	2.00	1.00	1.00	1.00	2.00	1.00	2.00	1.00
316	2.00	1.00	2.00	1.00	2.00	1.00	2.00	2.00
317	1.00	1.00	1.00	1.00	1.00	1.00	1.00	1.00
318	1.00	1.00	1.00	1.00	2.00	2.00	1.00	1.00
319	1.00	1.00	1.00	1.00	2.00	1.00	1.00	1.00
320	1.00	1.00	2.00	1.00	2.00	2.00	1.00	2.00
321	1.00	1.00	1.00	1.00	2.00	1.00	1.00	1.00
322	2.00	1.00	1.00	1.00	2.00	1.00	2.00	1.00
323	1.00	1.00	1.00	1.00	2.00	1.00	2.00	1.00
324	1.00	1.00	1.00	1.00	1.00	1.00	2.00	1.00
325	1.00	1.00	1.00	1.00	1.00	1.00	1.00	1.00
326	2.00	1.00	2.00	2.00	2.00	2.00	2.00	1.00
327	1.00	1.00	1.00	1.00	2.00	2.00	1.00	1.00
328	1.00	1.00	1.00	1.00	2.00	1.00	1.00	1.00
329	1.00	1.00	1.00	1.00	2.00	1.00	2.00	1.00
330	2.00	1.00	2.00	2.00	2.00	2.00	2.00	1.00
331	2.00	1.00	2.00	1.00	1.00	1.00	2.00	1.00
332	1.00	1.00	2.00	2.00	2.00	1.00	1.00	1.00
333	1.00	1.00	1.00	1.00	2.00	1.00	1.00	1.00
334	1.00	1.00	2.00	1.00	2.00	1.00	1.00	1.00
335	1.00	1.00	1.00	1.00	2.00	1.00	1.00	1.00
336	2.00	1.00	2.00	2.00	2.00	1.00	1.00	1.00

	M15	B1	B2	B3	B4	B5	B6	B7
295	1.00	2.00	2.00	2.00	1.00	2.00	1.00	1.00
296	1.00	1.00	1.00	1.00	1.00	1.00	1.00	1.00
297	1.00	2.00	1.00	2.00	1.00	2.00	2.00	1.00
298	1.00	1.00	1.00	1.00	1.00	2.00	2.00	2.00
299	1.00	1.00	2.00	2.00	2.00	2.00	2.00	2.00
300	1.00	1.00	1.00	1.00	1.00	1.00	1.00	1.00
301	1.00	2.00	2.00	2.00	2.00	2.00	2.00	2.00
302	1.00	2.00	2.00	2.00	2.00	2.00	1.00	1.00
303	1.00	2.00	1.00	2.00	1.00	2.00	1.00	1.00
304	1.00	1.00	1.00	2.00	2.00	1.00	2.00	1.00
305	1.00	2.00	1.00	1.00	1.00	2.00	2.00	2.00
306	2.00	2.00	1.00	1.00	1.00	2.00	2.00	2.00
307	1.00	2.00	2.00	1.00	1.00	2.00	2.00	2.00
308	1.00	2.00	2.00	2.00	1.00	2.00	2.00	2.00
309	1.00	2.00	2.00	2.00	2.00	2.00	2.00	2.00
310	1.00	2.00	1.00	2.00	1.00	2.00	2.00	2.00
311	1.00	1.00	2.00	1.00	1.00	1.00	2.00	2.00
312	1.00	2.00	2.00	2.00	1.00	1.00	2.00	2.00
313	2.00	1.00	2.00	2.00	1.00	1.00	1.00	1.00
314	1.00	2.00	1.00	2.00	1.00	2.00	2.00	2.00
315	1.00	2.00	1.00	1.00	1.00	2.00	2.00	2.00
316	1.00	1.00	1.00	1.00	1.00	2.00	2.00	2.00
317	1.00	2.00	1.00	1.00	2.00	2.00	2.00	2.00
318	1.00	1.00	2.00	2.00	1.00	2.00	1.00	1.00
319	1.00	1.00	2.00	2.00	1.00	1.00	2.00	2.00
320	2.00	1.00	1.00	1.00	1.00	1.00	1.00	1.00
321	1.00	1.00	1.00	1.00	1.00	1.00	1.00	1.00
322	2.00	1.00	1.00	1.00	1.00	1.00	2.00	2.00
323	1.00	1.00	1.00	2.00	1.00	2.00	2.00	2.00
324	2.00	2.00	2.00	2.00	1.00	2.00	1.00	1.00
325	1.00	1.00	2.00	1.00	1.00	1.00	2.00	2.00
326	2.00	2.00	1.00	2.00	2.00	2.00	2.00	2.00
327	1.00	2.00	1.00	1.00	1.00	2.00	2.00	2.00
328	1.00	1.00	1.00	2.00	1.00	2.00	2.00	2.00
329	1.00	2.00	1.00	1.00	2.00	2.00	2.00	2.00
330	2.00	2.00	2.00	2.00	2.00	2.00	2.00	2.00
331	1.00	1.00	2.00	2.00	1.00	1.00	1.00	1.00
332	1.00	1.00	1.00	1.00	1.00	2.00	2.00	2.00
333	1.00	2.00	2.00	2.00	1.00	2.00	2.00	2.00
334	1.00	2.00	1.00	2.00	2.00	2.00	2.00	2.00
335	1.00	2.00	2.00	2.00	1.00	1.00	2.00	2.00
336	1.00	2.00	1.00	2.00	2.00	2.00	2.00	2.00

	B8	B9	B10	B11	B12	B13	B14	W1
295	2.00	1.00	1.00	2.00	2.00	1.00	2.00	2.00
296	1.00	1.00	1.00	1.00	2.00	1.00	1.00	2.00
297	1.00	1.00	1.00	1.00	2.00	2.00	1.00	2.00
298	2.00	2.00	2.00	1.00	2.00	2.00	2.00	1.00
299	2.00	2.00	1.00	2.00	2.00	2.00	2.00	1.00
300	1.00	1.00	1.00	1.00	1.00	1.00	1.00	1.00
301	2.00	2.00	1.00	1.00	2.00	2.00	2.00	1.00
302	1.00	1.00	2.00	1.00	2.00	2.00	2.00	1.00
303	2.00	2.00	2.00	1.00	2.00	1.00	2.00	2.00
304	1.00	2.00	2.00	2.00	2.00	2.00	2.00	1.00
305	2.00	1.00	1.00	1.00	2.00	2.00	1.00	1.00
306	1.00	1.00	1.00	2.00	1.00	2.00	1.00	2.00
307	2.00	2.00	2.00	1.00	2.00	2.00	2.00	2.00
308	1.00	2.00	2.00	1.00	2.00	2.00	2.00	1.00
309	2.00	2.00	2.00	1.00	2.00	2.00	2.00	1.00
310	2.00	1.00	1.00	2.00	2.00	2.00	2.00	1.00
311	2.00	2.00	2.00	2.00	2.00	2.00	1.00	2.00
312	2.00	2.00	2.00	1.00	2.00	2.00	2.00	1.00
313	2.00	2.00	2.00	1.00	1.00	1.00	1.00	1.00
314	2.00	2.00	1.00	1.00	2.00	2.00	2.00	1.00
315	2.00	2.00	2.00	1.00	2.00	2.00	2.00	2.00
316	2.00	2.00	2.00	1.00	2.00	2.00	1.00	1.00
317	1.00	2.00	2.00	2.00	2.00	2.00	2.00	1.00
318	1.00	2.00	1.00	2.00	2.00	2.00	2.00	1.00
319	2.00	2.00	1.00	2.00	2.00	2.00	1.00	1.00
320	1.00	1.00	2.00	2.00	2.00	2.00	1.00	1.00
321	1.00	2.00	1.00	1.00	1.00	2.00	1.00	1.00
322	2.00	2.00	1.00	1.00	2.00	1.00	2.00	1.00
323	2.00	2.00	1.00	1.00	2.00	2.00	2.00	2.00
324	2.00	1.00	1.00	1.00	2.00	2.00	1.00	1.00
325	2.00	2.00	1.00	1.00	2.00	2.00	2.00	2.00
326	2.00	2.00	2.00	1.00	2.00	2.00	2.00	1.00
327	2.00	2.00	1.00	1.00	1.00	2.00	2.00	1.00
328	2.00	1.00	1.00	1.00	1.00	2.00	2.00	2.00
329	2.00	2.00	2.00	2.00	2.00	2.00	2.00	2.00
330	2.00	2.00	2.00	1.00	1.00	2.00	2.00	2.00
331	1.00	2.00	1.00	1.00	2.00	1.00	1.00	1.00
332	1.00	1.00	1.00	1.00	1.00	2.00	1.00	1.00
333	2.00	2.00	1.00	1.00	2.00	2.00	2.00	1.00
334	2.00	2.00	2.00	2.00	2.00	2.00	2.00	1.00
335	2.00	2.00	2.00	2.00	1.00	1.00	1.00	1.00
336	2.00	2.00	2.00	1.00	2.00	2.00	2.00	1.00

	W2	W3	W4	W5	W6	E1	V1	V2
295	2.00	2.00	2.00	1.00	2.00	1.00	1.00	2.00
296	2.00	1.00	1.00	1.00	1.00	1.00	2.00	2.00
297	2.00	1.00	2.00	1.00	1.00	1.00	2.00	2.00
298	2.00	2.00	1.00	2.00	2.00	1.00	1.00	2.00
299	1.00	1.00	2.00	1.00	1.00	1.00	2.00	2.00
300	1.00	1.00	1.00	1.00	1.00	1.00	1.00	1.00
301	1.00	1.00	1.00	1.00	1.00	1.00	2.00	2.00
302	1.00	1.00	1.00	1.00	2.00	1.00	2.00	2.00
303	2.00	1.00	1.00	1.00	2.00	1.00	2.00	2.00
304	1.00	1.00	1.00	1.00	2.00	1.00	2.00	2.00
305	1.00	1.00	1.00	1.00	2.00	1.00	2.00	2.00
306	2.00	2.00	2.00	2.00	2.00	1.00	1.00	1.00
307	2.00	2.00	2.00	1.00	1.00	1.00	2.00	2.00
308	1.00	2.00	1.00	1.00	2.00	1.00	2.00	2.00
309	2.00	1.00	1.00	1.00	1.00	1.00	2.00	2.00
310	1.00	1.00	1.00	1.00	2.00	1.00	2.00	2.00
311	2.00	2.00	2.00	2.00	2.00	1.00	2.00	2.00
312	1.00	2.00	1.00	1.00	2.00	1.00	2.00	2.00
313	1.00	1.00	1.00	2.00	2.00	1.00	2.00	2.00
314	2.00	1.00	2.00	2.00	2.00	1.00	1.00	2.00
315	1.00	2.00	1.00	1.00	1.00	1.00	1.00	2.00
316	1.00	2.00	1.00	1.00	2.00	1.00	2.00	1.00
317	2.00	1.00	2.00	2.00	2.00	1.00	2.00	2.00
318	1.00	1.00	1.00	1.00	1.00	1.00	2.00	2.00
319	1.00	1.00	1.00	1.00	2.00	1.00	2.00	2.00
320	2.00	1.00	2.00	1.00	2.00	1.00	2.00	1.00
321	1.00	1.00	1.00	1.00	1.00	1.00	2.00	2.00
322	1.00	2.00	2.00	1.00	2.00	1.00	2.00	2.00
323	2.00	1.00	1.00	1.00	1.00	1.00	2.00	2.00
324	1.00	1.00	1.00	1.00	2.00	1.00	2.00	2.00
325	2.00	1.00	1.00	1.00	2.00	1.00	2.00	2.00
326	2.00	1.00	1.00	1.00	1.00	1.00	2.00	2.00
327	2.00	2.00	1.00	1.00	2.00	1.00	2.00	2.00
328	2.00	1.00	1.00	1.00	1.00	1.00	2.00	2.00
329	2.00	1.00	1.00	1.00	2.00	1.00	2.00	2.00
330	1.00	1.00	1.00	1.00	1.00	1.00	2.00	2.00
331	1.00	1.00	2.00	1.00	2.00	1.00	2.00	2.00
332	1.00	2.00	1.00	1.00	2.00	1.00	2.00	2.00
333	1.00	1.00	2.00	2.00	2.00	1.00	2.00	2.00
334	2.00	1.00	1.00	1.00	2.00	1.00	2.00	2.00
335	1.00	1.00	1.00	1.00	2.00	1.00	1.00	1.00
336	1.00	1.00	1.00	2.00	2.00	1.00	2.00	2.00

	V3	V4	V5	V6	E2	E3	E4	C1
295	1.00	2.00	1.00	2.00	1.00	2.00	2.00	2.00
296	2.00	1.00	2.00	1.00	1.00	2.00	2.00	2.00
297	2.00	2.00	2.00	2.00	1.00	2.00	2.00	2.00
298	2.00	2.00	2.00	2.00	1.00	2.00	1.00	2.00
299	2.00	2.00	1.00	1.00	1.00	2.00	2.00	2.00
300	1.00	1.00	1.00	1.00	1.00	2.00	2.00	1.00
301	1.00	1.00	1.00	2.00	1.00	2.00	2.00	2.00
302	2.00	1.00	1.00	2.00	1.00	1.00	1.00	2.00
303	1.00	1.00	1.00	1.00	1.00	2.00	1.00	2.00
304	1.00	1.00	2.00	2.00	1.00	2.00	1.00	2.00
305	1.00	1.00	1.00	2.00	1.00	2.00	1.00	2.00
306	1.00	1.00	1.00	2.00	1.00	2.00	1.00	2.00
307	2.00	2.00	2.00	1.00	1.00	2.00	2.00	2.00
308	2.00	1.00	2.00	1.00	1.00	2.00	1.00	2.00
309	2.00	2.00	2.00	1.00	1.00	2.00	1.00	2.00
310	2.00	2.00	2.00	2.00	1.00	2.00	2.00	2.00
311	2.00	2.00	2.00	2.00	1.00	2.00	2.00	2.00
312	2.00	2.00	2.00	2.00	1.00	2.00	1.00	2.00
313	2.00	1.00	1.00	2.00	1.00	2.00	1.00	2.00
314	2.00	2.00	2.00	2.00	1.00	2.00	2.00	2.00
315	1.00	2.00	2.00	2.00	1.00	2.00	2.00	2.00
316	1.00	1.00	1.00	2.00	1.00	2.00	2.00	2.00
317	2.00	1.00	1.00	1.00	1.00	2.00	1.00	2.00
318	2.00	2.00	2.00	1.00	1.00	2.00	1.00	2.00
319	1.00	2.00	2.00	2.00	1.00	2.00	2.00	2.00
320	2.00	1.00	2.00	2.00	1.00	2.00	1.00	2.00
321	2.00	2.00	2.00	2.00	1.00	2.00	2.00	2.00
322	2.00	1.00	2.00	2.00	1.00	2.00	2.00	2.00
323	1.00	1.00	1.00	1.00	1.00	1.00	1.00	2.00
324	2.00	1.00	2.00	1.00	1.00	2.00	2.00	2.00
325	2.00	1.00	1.00	2.00	1.00	2.00	2.00	2.00
326	1.00	2.00	2.00	2.00	1.00	2.00	2.00	2.00
327	2.00	2.00	1.00	2.00	1.00	2.00	2.00	2.00
328	1.00	1.00	2.00	1.00	1.00	2.00	1.00	2.00
329	1.00	2.00	1.00	2.00	1.00	2.00	1.00	2.00
330	1.00	2.00	2.00	2.00	1.00	2.00	1.00	2.00
331	1.00	1.00	2.00	1.00	1.00	2.00	2.00	1.00
332	1.00	1.00	1.00	2.00	1.00	2.00	2.00	1.00
333	2.00	2.00	1.00	2.00	1.00	2.00	2.00	2.00
334	1.00	1.00	1.00	2.00	1.00	2.00	2.00	2.00
335	1.00	1.00	1.00	2.00	1.00	2.00	1.00	2.00
336	1.00	1.00	1.00	2.00	1.00	2.00	1.00	2.00

	C2	C3	C4	C5	C6	C7	filter_$
295	2.00	1.00	1.00	2.00	2.00	2.00	1
296	1.00	1.00	1.00	1.00	2.00	2.00	1
297	2.00	1.00	1.00	2.00	2.00	2.00	1
298	2.00	1.00	2.00	2.00	2.00	2.00	1
299	2.00	1.00	2.00	2.00	2.00	2.00	1
300	1.00	1.00	1.00	1.00	1.00	2.00	1
301	2.00	1.00	2.00	2.00	2.00	2.00	1
302	2.00	1.00	1.00	2.00	1.00	2.00	1
303	2.00	1.00	2.00	2.00	2.00	2.00	1
304	2.00	1.00	1.00	1.00	2.00	2.00	1
305	2.00	1.00	1.00	2.00	2.00	2.00	1
306	2.00	1.00	2.00	2.00	2.00	2.00	1
307	2.00	1.00	2.00	2.00	2.00	2.00	1
308	2.00	1.00	1.00	1.00	1.00	2.00	1
309	2.00	1.00	1.00	1.00	2.00	2.00	1
310	2.00	1.00	2.00	2.00	2.00	2.00	1
311	2.00	2.00	2.00	2.00	2.00	1.00	1
312	2.00	1.00	2.00	2.00	2.00	2.00	1
313	2.00	1.00	2.00	1.00	2.00	2.00	1
314	2.00	1.00	2.00	2.00	2.00	2.00	1
315	2.00	1.00	2.00	2.00	2.00	2.00	1
316	2.00	1.00	1.00	2.00	2.00	2.00	1
317	2.00	1.00	2.00	2.00	2.00	2.00	1
318	2.00	2.00	2.00	2.00	2.00	1.00	1
319	2.00	1.00	2.00	2.00	2.00	2.00	1
320	2.00	1.00	2.00	2.00	2.00	2.00	1
321	2.00	1.00	2.00	2.00	2.00	2.00	1
322	2.00	2.00	1.00	2.00	2.00	2.00	1
323	2.00	1.00	2.00	1.00	2.00	2.00	1
324	2.00	1.00	2.00	2.00	2.00	2.00	1
325	2.00	2.00	2.00	1.00	2.00	2.00	1
326	2.00	2.00	2.00	2.00	2.00	1.00	1
327	2.00	1.00	2.00	1.00	1.00	2.00	1
328	2.00	1.00	1.00	2.00	2.00	2.00	1
329	2.00	1.00	2.00	2.00	2.00	2.00	1
330	2.00	1.00	1.00	2.00	2.00	2.00	1
331	2.00	1.00	1.00	2.00	2.00	2.00	1
332	1.00	1.00	1.00	1.00	2.00	2.00	1
333	2.00	1.00	2.00	1.00	1.00	2.00	1
334	2.00	1.00	1.00	1.00	2.00	2.00	1
335	2.00	1.00	1.00	2.00	2.00	2.00	1
336	2.00	2.00	2.00	2.00	2.00	1.00	1

	SN	Group	Gender	Age	Work	Work2	Work3	Sector
337	446.00	1.00	2.00	23.00	6.00	1.00	1.00	2.00
338	447.00	1.00	2.00	24.00	7.00	2.00	2.00	1.00
339	448.00	1.00	2.00	21.00	4.00	1.00	1.00	1.00
340	449.00	1.00	1.00	25.00	8.00	2.00	2.00	2.00
341	450.00	1.00	1.00	28.00	8.00	2.00	2.00	1.00
342	451.00	1.00	2.00	24.00	7.00	2.00	2.00	1.00
343	452.00	1.00	1.00	31.00	10.00	3.00	2.00	1.00
344	453.00	1.00	1.00	23.00	5.00	1.00	1.00	1.00
345	454.00	1.00	2.00	28.00	8.00	2.00	2.00	2.00
346	455.00	1.00	2.00	25.00	8.00	2.00	2.00	2.00
347	456.00	1.00	1.00	34.00	10.00	3.00	2.00	1.00
348	457.00	1.00	1.00	22.00	5.00	1.00	1.00	1.00
349	458.00	1.00	1.00	28.00	8.00	2.00	2.00	1.00
350	459.00	1.00	1.00	34.00	10.00	3.00	2.00	1.00
351	460.00	1.00	2.00	25.00	8.00	2.00	2.00	2.00
352	461.00	1.00	1.00	27.00	8.00	2.00	2.00	2.00
353	462.00	1.00	1.00	22.00	5.00	1.00	1.00	1.00
354	465.00	1.00	1.00	27.00	8.00	2.00	2.00	2.00
355	466.00	1.00	1.00	26.00	8.00	2.00	2.00	2.00
356	467.00	1.00	2.00	26.00	8.00	2.00	2.00	2.00
357	468.00	1.00	1.00	27.00	8.00	2.00	2.00	2.00
358	469.00	1.00	1.00	28.00	8.00	2.00	2.00	2.00
359	470.00	1.00	1.00	27.00	8.00	2.00	2.00	1.00
360	471.00	1.00	1.00	22.00	5.00	1.00	1.00	1.00
361	472.00	1.00	1.00	27.00	8.00	2.00	2.00	2.00
362	473.00	1.00	1.00	31.00	9.00	3.00	2.00	1.00
363	476.00	1.00	1.00	21.00	3.00	1.00	1.00	1.00
364	477.00	1.00	1.00	26.00	8.00	2.00	2.00	1.00
365	479.00	1.00	1.00	26.00	8.00	2.00	2.00	2.00
366	481.00	1.00	2.00	26.00	8.00	2.00	2.00	1.00
367	482.00	1.00	1.00	27.00	8.00	2.00	2.00	2.00
368	483.00	1.00	1.00	24.00	6.00	1.00	1.00	1.00
369	484.00	1.00	1.00	28.00	9.00	3.00	2.00	1.00
370	486.00	1.00	1.00	32.00	9.00	3.00	2.00	1.00
371	487.00	1.00	1.00	38.00	8.00	2.00	2.00	2.00
372	488.00	1.00	1.00	24.00	4.00	1.00	1.00	1.00
373	491.00	1.00	1.00	30.00	8.00	2.00	2.00	2.00
374	492.00	1.00	1.00	30.00	9.00	3.00	2.00	1.00
375	493.00	1.00	1.00	23.00	5.00	1.00	1.00	1.00
376	494.00	1.00	1.00	28.00	8.00	2.00	2.00	2.00
377	495.00	1.00	1.00	28.00	8.00	2.00	2.00	1.00
378	496.00	1.00	1.00	38.00	10.00	3.00	2.00	1.00

	Region	Nationality	M1	M2	M3	M4	M5	M6
337	2.00	1.00	1.00	1.00	1.00	1.00	1.00	1.00
338	2.00	1.00	1.00	1.00	2.00	1.00	1.00	1.00
339	2.00	1.00	2.00	1.00	2.00	2.00	1.00	1.00
340	3.00	1.00	1.00	1.00	1.00	1.00	1.00	1.00
341	2.00	1.00	1.00	1.00	2.00	2.00	2.00	1.00
342	2.00	1.00	1.00	1.00	1.00	1.00	1.00	1.00
343	2.00	1.00	1.00	1.00	1.00	1.00	1.00	1.00
344	4.00	1.00	1.00	2.00	1.00	1.00	1.00	1.00
345	2.00	1.00	1.00	1.00	2.00	1.00	1.00	1.00
346	2.00	1.00	2.00	1.00	1.00	1.00	1.00	1.00
347	2.00	1.00	1.00	1.00	2.00	1.00	2.00	2.00
348	4.00	1.00	1.00	1.00	2.00	1.00	2.00	2.00
349	5.00	1.00	1.00	1.00	2.00	1.00	2.00	2.00
350	2.00	1.00	1.00	1.00	2.00	1.00	1.00	1.00
351	5.00	1.00	1.00	1.00	1.00	1.00	1.00	1.00
352	4.00	1.00	2.00	2.00	2.00	2.00	2.00	2.00
353	4.00	1.00	2.00	1.00	2.00	2.00	1.00	1.00
354	4.00	1.00	1.00	1.00	1.00	1.00	1.00	1.00
355	4.00	1.00	1.00	1.00	1.00	1.00	1.00	1.00
356	3.00	1.00	1.00	1.00	1.00	1.00	1.00	1.00
357	3.00	1.00	1.00	1.00	1.00	1.00	2.00	1.00
358	4.00	1.00	1.00	1.00	1.00	1.00	1.00	1.00
359	1.00	1.00	1.00	2.00	1.00	2.00	1.00	1.00
360	4.00	1.00	1.00	1.00	2.00	1.00	1.00	2.00
361	2.00	1.00	2.00	1.00	2.00	2.00	1.00	1.00
362	2.00	1.00	1.00	1.00	1.00	1.00	1.00	1.00
363	4.00	1.00	1.00	1.00	2.00	1.00	1.00	1.00
364	4.00	1.00	1.00	2.00	1.00	2.00	1.00	1.00
365	5.00	1.00	1.00	1.00	2.00	1.00	1.00	2.00
366	1.00	1.00	1.00	1.00	1.00	1.00	1.00	1.00
367	4.00	1.00	1.00	1.00	2.00	1.00	1.00	2.00
368	1.00	1.00	1.00	1.00	2.00	2.00	1.00	2.00
369	2.00	1.00	1.00	2.00	2.00	1.00	1.00	1.00
370	5.00	1.00	1.00	1.00	1.00	1.00	1.00	1.00
371	4.00	2.00	1.00	1.00	1.00	1.00	1.00	1.00
372	2.00	1.00	2.00	1.00	2.00	1.00	1.00	2.00
373	3.00	1.00	1.00	1.00	2.00	1.00	2.00	2.00
374	3.00	1.00	1.00	2.00	2.00	2.00	2.00	1.00
375	3.00	1.00	1.00	1.00	1.00	1.00	1.00	1.00
376	4.00	1.00	2.00	2.00	2.00	1.00	1.00	2.00
377	5.00	1.00	1.00	1.00	1.00	1.00	1.00	1.00
378	3.00	1.00	1.00	1.00	2.00	2.00	2.00	2.00

	M7	M8	M9	M10	M11	M12	M13	M14
337	1.00	1.00	1.00	1.00	1.00	1.00	1.00	1.00
338	2.00	1.00	1.00	1.00	2.00	1.00	2.00	1.00
339	2.00	1.00	2.00	2.00	2.00	1.00	2.00	1.00
340	2.00	1.00	2.00	1.00	2.00	1.00	1.00	1.00
341	1.00	2.00	1.00	1.00	2.00	2.00	2.00	1.00
342	1.00	1.00	1.00	1.00	2.00	1.00	2.00	1.00
343	1.00	1.00	1.00	1.00	2.00	1.00	2.00	2.00
344	2.00	2.00	1.00	1.00	1.00	1.00	1.00	1.00
345	1.00	2.00	2.00	1.00	1.00	1.00	2.00	1.00
346	1.00	1.00	2.00	2.00	2.00	1.00	2.00	1.00
347	1.00	1.00	1.00	1.00	2.00	1.00	1.00	2.00
348	1.00	1.00	1.00	1.00	2.00	1.00	1.00	1.00
349	2.00	1.00	2.00	1.00	1.00	2.00	2.00	2.00
350	1.00	1.00	1.00	1.00	2.00	1.00	2.00	1.00
351	1.00	1.00	1.00	1.00	1.00	2.00	2.00	1.00
352	2.00	2.00	2.00	1.00	2.00	1.00	1.00	1.00
353	1.00	1.00	2.00	1.00	1.00	1.00	1.00	1.00
354	1.00	1.00	1.00	1.00	2.00	1.00	1.00	1.00
355	1.00	1.00	1.00	1.00	1.00	1.00	1.00	1.00
356	2.00	1.00	1.00	1.00	1.00	1.00	2.00	1.00
357	1.00	1.00	1.00	1.00	2.00	1.00	1.00	1.00
358	1.00	1.00	1.00	1.00	1.00	1.00	1.00	1.00
359	1.00	2.00	1.00	2.00	1.00	1.00	1.00	2.00
360	1.00	2.00	1.00	1.00	2.00	1.00	2.00	1.00
361	1.00	1.00	1.00	1.00	1.00	1.00	2.00	1.00
362	2.00	1.00	1.00	2.00	2.00	1.00	1.00	1.00
363	1.00	1.00	1.00	1.00	2.00	1.00	1.00	1.00
364	1.00	2.00	2.00	2.00	1.00	1.00	1.00	2.00
365	2.00	1.00	1.00	1.00	1.00	1.00	1.00	1.00
366	1.00	1.00	1.00	1.00	1.00	1.00	1.00	1.00
367	2.00	2.00	2.00	1.00	1.00	1.00	1.00	1.00
368	2.00	1.00	2.00	1.00	2.00	1.00	1.00	1.00
369	1.00	2.00	1.00	1.00	2.00	2.00	2.00	2.00
370	1.00	1.00	1.00	1.00	1.00	1.00	1.00	1.00
371	1.00	1.00	1.00	1.00	2.00	2.00	2.00	2.00
372	1.00	1.00	1.00	1.00	2.00	1.00	2.00	2.00
373	2.00	1.00	1.00	1.00	2.00	1.00	2.00	2.00
374	2.00	2.00	1.00	1.00	2.00	1.00	2.00	2.00
375	1.00	1.00	1.00	1.00	1.00	1.00	1.00	2.00
376	1.00	1.00	1.00	1.00	1.00	1.00	2.00	1.00
377	1.00	1.00	1.00	1.00	1.00	1.00	1.00	1.00
378	1.00	1.00	1.00	2.00	2.00	1.00	1.00	1.00

	M15	B1	B2	B3	B4	B5	B6	B7
337	1.00	1.00	1.00	1.00	1.00	1.00	2.00	2.00
338	1.00	2.00	2.00	2.00	2.00	2.00	2.00	2.00
339	1.00	1.00	1.00	2.00	1.00	2.00	2.00	2.00
340	1.00	1.00	1.00	1.00	2.00	1.00	1.00	1.00
341	1.00	1.00	1.00	2.00	2.00	1.00	2.00	2.00
342	1.00	2.00	1.00	2.00	2.00	2.00	2.00	2.00
343	2.00	2.00	2.00	1.00	2.00	2.00	2.00	1.00
344	1.00	2.00	2.00	1.00	1.00	2.00	1.00	2.00
345	2.00	2.00	2.00	2.00	1.00	1.00	1.00	1.00
346	1.00	2.00	1.00	1.00	1.00	1.00	2.00	2.00
347	2.00	1.00	1.00	1.00	2.00	2.00	2.00	2.00
348	1.00	2.00	2.00	2.00	1.00	2.00	2.00	2.00
349	1.00	2.00	2.00	1.00	1.00	1.00	2.00	2.00
350	1.00	2.00	2.00	2.00	1.00	2.00	2.00	2.00
351	2.00	2.00	1.00	1.00	1.00	2.00	2.00	2.00
352	2.00	1.00	1.00	1.00	1.00	1.00	1.00	1.00
353	1.00	1.00	1.00	1.00	1.00	1.00	2.00	2.00
354	1.00	2.00	1.00	1.00	1.00	2.00	2.00	2.00
355	1.00	1.00	1.00	1.00	1.00	2.00	2.00	2.00
356	1.00	2.00	1.00	2.00	1.00	2.00	2.00	2.00
357	1.00	2.00	1.00	1.00	2.00	2.00	2.00	2.00
358	1.00	1.00	1.00	1.00	1.00	1.00	1.00	1.00
359	2.00	1.00	2.00	2.00	2.00	2.00	1.00	1.00
360	1.00	2.00	2.00	2.00	1.00	2.00	1.00	1.00
361	1.00	2.00	1.00	1.00	1.00	1.00	2.00	2.00
362	1.00	1.00	2.00	2.00	1.00	1.00	1.00	1.00
363	1.00	1.00	2.00	2.00	1.00	2.00	2.00	2.00
364	2.00	1.00	1.00	1.00	1.00	1.00	1.00	1.00
365	1.00	2.00	1.00	1.00	1.00	2.00	1.00	1.00
366	1.00	2.00	1.00	1.00	1.00	1.00	1.00	1.00
367	1.00	1.00	1.00	1.00	1.00	1.00	2.00	2.00
368	1.00	2.00	2.00	2.00	1.00	1.00	1.00	1.00
369	1.00	2.00	1.00	1.00	1.00	2.00	2.00	2.00
370	1.00	1.00	1.00	1.00	1.00	1.00	2.00	2.00
371	1.00	2.00	2.00	2.00	2.00	2.00	2.00	2.00
372	2.00	2.00	2.00	2.00	1.00	1.00	2.00	2.00
373	1.00	2.00	2.00	2.00	1.00	2.00	2.00	2.00
374	1.00	2.00	1.00	2.00	2.00	2.00	2.00	2.00
375	1.00	2.00	2.00	2.00	1.00	2.00	2.00	2.00
376	1.00	1.00	2.00	1.00	1.00	2.00	2.00	2.00
377	1.00	1.00	1.00	2.00	1.00	1.00	2.00	2.00
378	1.00	1.00	1.00	1.00	1.00	2.00	2.00	2.00

	B8	B9	B10	B11	B12	B13	B14	W1
337	1.00	2.00	1.00	1.00	1.00	2.00	1.00	1.00
338	2.00	2.00	2.00	1.00	2.00	2.00	2.00	1.00
339	2.00	1.00	2.00	1.00	1.00	2.00	2.00	1.00
340	1.00	2.00	2.00	2.00	2.00	2.00	2.00	1.00
341	2.00	2.00	2.00	1.00	2.00	2.00	2.00	1.00
342	2.00	2.00	2.00	2.00	2.00	2.00	2.00	2.00
343	1.00	2.00	1.00	1.00	2.00	2.00	2.00	2.00
344	1.00	2.00	1.00	2.00	2.00	1.00	2.00	1.00
345	1.00	1.00	2.00	2.00	2.00	2.00	2.00	2.00
346	2.00	2.00	1.00	1.00	2.00	2.00	2.00	2.00
347	2.00	2.00	1.00	1.00	2.00	2.00	1.00	1.00
348	2.00	2.00	1.00	1.00	2.00	2.00	2.00	2.00
349	2.00	2.00	1.00	1.00	2.00	2.00	2.00	2.00
350	2.00	2.00	2.00	1.00	2.00	2.00	2.00	2.00
351	2.00	2.00	2.00	2.00	2.00	2.00	2.00	2.00
352	1.00	1.00	1.00	1.00	1.00	2.00	1.00	2.00
353	2.00	2.00	1.00	1.00	2.00	2.00	2.00	1.00
354	2.00	2.00	2.00	2.00	2.00	2.00	2.00	1.00
355	1.00	1.00	1.00	1.00	2.00	2.00	1.00	1.00
356	2.00	2.00	1.00	1.00	2.00	2.00	2.00	2.00
357	2.00	2.00	2.00	2.00	2.00	2.00	2.00	2.00
358	1.00	1.00	1.00	1.00	1.00	1.00	1.00	2.00
359	2.00	1.00	1.00	1.00	1.00	1.00	1.00	1.00
360	1.00	2.00	1.00	1.00	2.00	2.00	2.00	2.00
361	2.00	2.00	2.00	2.00	2.00	2.00	1.00	1.00
362	2.00	1.00	1.00	1.00	1.00	1.00	1.00	1.00
363	1.00	2.00	2.00	2.00	2.00	2.00	2.00	1.00
364	2.00	2.00	2.00	2.00	1.00	1.00	1.00	2.00
365	1.00	2.00	1.00	1.00	1.00	1.00	1.00	1.00
366	1.00	2.00	2.00	2.00	1.00	2.00	2.00	2.00
367	2.00	2.00	1.00	1.00	1.00	2.00	1.00	2.00
368	1.00	2.00	1.00	1.00	1.00	2.00	1.00	1.00
369	2.00	2.00	2.00	1.00	2.00	2.00	2.00	1.00
370	2.00	2.00	2.00	2.00	1.00	2.00	2.00	1.00
371	1.00	2.00	2.00	1.00	2.00	2.00	2.00	1.00
372	2.00	1.00	2.00	1.00	2.00	2.00	1.00	1.00
373	2.00	1.00	1.00	1.00	2.00	2.00	2.00	2.00
374	2.00	2.00	2.00	1.00	1.00	2.00	2.00	2.00
375	2.00	1.00	1.00	2.00	2.00	2.00	2.00	1.00
376	2.00	1.00	1.00	1.00	2.00	2.00	2.00	2.00
377	1.00	2.00	1.00	1.00	1.00	2.00	1.00	2.00
378	2.00	2.00	1.00	1.00	2.00	2.00	1.00	1.00

	W2	W3	W4	W5	W6	E1	V1	V2
337	1.00	1.00	1.00	1.00	1.00	1.00	2.00	2.00
338	1.00	1.00	2.00	1.00	2.00	1.00	2.00	2.00
339	1.00	2.00	2.00	1.00	2.00	1.00	2.00	2.00
340	1.00	1.00	1.00	1.00	1.00	1.00	1.00	1.00
341	2.00	2.00	2.00	2.00	2.00	1.00	2.00	2.00
342	2.00	2.00	2.00	2.00	2.00	1.00	2.00	2.00
343	2.00	1.00	2.00	1.00	1.00	1.00	2.00	2.00
344	2.00	2.00	1.00	1.00	2.00	1.00	2.00	2.00
345	2.00	1.00	2.00	2.00	2.00	1.00	2.00	2.00
346	2.00	1.00	1.00	1.00	1.00	1.00	2.00	2.00
347	2.00	2.00	2.00	2.00	2.00	1.00	2.00	2.00
348	2.00	1.00	2.00	2.00	2.00	1.00	2.00	2.00
349	2.00	2.00	2.00	2.00	2.00	1.00	2.00	2.00
350	2.00	1.00	2.00	2.00	2.00	1.00	2.00	2.00
351	2.00	1.00	1.00	2.00	2.00	1.00	2.00	2.00
352	2.00	2.00	2.00	2.00	2.00	1.00	2.00	1.00
353	1.00	1.00	1.00	1.00	1.00	1.00	2.00	2.00
354	1.00	2.00	1.00	1.00	2.00	1.00	2.00	1.00
355	1.00	1.00	1.00	1.00	1.00	1.00	2.00	2.00
356	1.00	1.00	1.00	1.00	1.00	1.00	2.00	2.00
357	1.00	2.00	1.00	2.00	1.00	1.00	2.00	2.00
358	2.00	2.00	2.00	2.00	2.00	1.00	1.00	1.00
359	1.00	1.00	1.00	1.00	1.00	1.00	1.00	1.00
360	2.00	1.00	2.00	1.00	2.00	1.00	2.00	2.00
361	2.00	2.00	2.00	1.00	2.00	1.00	2.00	2.00
362	1.00	1.00	1.00	1.00	1.00	1.00	1.00	2.00
363	1.00	1.00	1.00	1.00	2.00	1.00	2.00	2.00
364	1.00	1.00	1.00	2.00	2.00	1.00	1.00	2.00
365	1.00	1.00	1.00	1.00	1.00	1.00	1.00	1.00
366	2.00	1.00	2.00	1.00	2.00	1.00	2.00	2.00
367	2.00	2.00	2.00	1.00	2.00	1.00	2.00	2.00
368	1.00	2.00	2.00	2.00	2.00	1.00	1.00	1.00
369	1.00	2.00	1.00	1.00	2.00	1.00	1.00	1.00
370	2.00	1.00	2.00	2.00	2.00	1.00	1.00	2.00
371	1.00	1.00	1.00	1.00	1.00	1.00	2.00	2.00
372	1.00	2.00	1.00	1.00	2.00	1.00	1.00	2.00
373	2.00	1.00	2.00	1.00	1.00	1.00	2.00	2.00
374	1.00	2.00	2.00	1.00	2.00	1.00	2.00	1.00
375	1.00	1.00	1.00	1.00	2.00	1.00	1.00	2.00
376	2.00	1.00	1.00	1.00	1.00	1.00	2.00	2.00
377	2.00	1.00	2.00	2.00	2.00	1.00	2.00	2.00
378	2.00	1.00	2.00	2.00	2.00	1.00	2.00	2.00

	V3	V4	V5	V6	E2	E3	E4	C1
337	1.00	2.00	1.00	1.00	1.00	2.00	2.00	1.00
338	2.00	2.00	1.00	1.00	1.00	2.00	1.00	2.00
339	2.00	2.00	2.00	1.00	1.00	2.00	2.00	2.00
340	1.00	2.00	1.00	1.00	1.00	2.00	1.00	1.00
341	1.00	2.00	1.00	2.00	1.00	2.00	2.00	2.00
342	2.00	2.00	2.00	2.00	1.00	2.00	2.00	2.00
343	1.00	2.00	2.00	2.00	1.00	2.00	2.00	2.00
344	2.00	2.00	1.00	1.00	1.00	2.00	1.00	2.00
345	1.00	2.00	2.00	2.00	1.00	2.00	2.00	2.00
346	1.00	1.00	1.00	1.00	1.00	2.00	1.00	2.00
347	1.00	2.00	2.00	2.00	1.00	2.00	2.00	2.00
348	1.00	2.00	2.00	2.00	1.00	2.00	2.00	2.00
349	2.00	2.00	2.00	2.00	1.00	2.00	2.00	2.00
350	1.00	2.00	2.00	2.00	1.00	1.00	2.00	2.00
351	1.00	1.00	2.00	2.00	1.00	2.00	2.00	2.00
352	2.00	2.00	1.00	2.00	1.00	2.00	1.00	2.00
353	2.00	2.00	2.00	2.00	1.00	2.00	1.00	2.00
354	2.00	1.00	1.00	1.00	1.00	2.00	2.00	2.00
355	1.00	1.00	2.00	1.00	1.00	1.00	1.00	2.00
356	2.00	2.00	1.00	2.00	1.00	2.00	2.00	2.00
357	2.00	2.00	2.00	1.00	1.00	2.00	1.00	2.00
358	1.00	1.00	1.00	1.00	1.00	2.00	2.00	1.00
359	2.00	2.00	1.00	1.00	1.00	2.00	1.00	2.00
360	1.00	2.00	2.00	2.00	1.00	2.00	2.00	2.00
361	2.00	1.00	2.00	2.00	1.00	2.00	1.00	2.00
362	1.00	1.00	1.00	1.00	1.00	2.00	1.00	2.00
363	2.00	2.00	2.00	2.00	1.00	2.00	2.00	2.00
364	1.00	2.00	1.00	2.00	1.00	2.00	2.00	2.00
365	1.00	1.00	1.00	1.00	1.00	2.00	1.00	1.00
366	1.00	1.00	2.00	2.00	1.00	2.00	1.00	2.00
367	1.00	2.00	2.00	2.00	1.00	2.00	2.00	2.00
368	2.00	1.00	2.00	2.00	1.00	2.00	1.00	2.00
369	1.00	1.00	1.00	2.00	1.00	1.00	2.00	2.00
370	1.00	2.00	2.00	2.00	1.00	2.00	2.00	2.00
371	1.00	2.00	1.00	2.00	1.00	1.00	2.00	2.00
372	2.00	2.00	1.00	2.00	1.00	2.00	1.00	2.00
373	1.00	1.00	1.00	2.00	1.00	2.00	1.00	2.00
374	1.00	2.00	1.00	2.00	1.00	2.00	2.00	2.00
375	1.00	1.00	1.00	2.00	1.00	2.00	1.00	2.00
376	1.00	2.00	1.00	1.00	1.00	2.00	1.00	2.00
377	1.00	2.00	2.00	2.00	1.00	2.00	2.00	2.00
378	1.00	1.00	2.00	2.00	1.00	2.00	1.00	2.00

	C2	C3	C4	C5	C6	C7	filter_$
337	1.00	1.00	1.00	1.00	1.00	2.00	1
338	2.00	1.00	2.00	2.00	2.00	2.00	1
339	2.00	1.00	2.00	2.00	2.00	2.00	1
340	1.00	1.00	2.00	1.00	1.00	2.00	1
341	2.00	1.00	1.00	2.00	2.00	2.00	1
342	2.00	1.00	1.00	2.00	2.00	2.00	1
343	2.00	1.00	2.00	2.00	2.00	2.00	1
344	2.00	1.00	1.00	1.00	2.00	2.00	1
345	2.00	1.00	1.00	2.00	2.00	2.00	1
346	2.00	1.00	1.00	1.00	2.00	2.00	1
347	2.00	1.00	2.00	2.00	2.00	2.00	1
348	2.00	1.00	1.00	2.00	2.00	2.00	1
349	2.00	1.00	1.00	1.00	2.00	2.00	1
350	1.00	1.00	1.00	1.00	2.00	2.00	1
351	2.00	1.00	2.00	1.00	2.00	2.00	1
352	2.00	1.00	1.00	2.00	1.00	2.00	1
353	2.00	2.00	2.00	2.00	2.00	1.00	1
354	2.00	1.00	1.00	2.00	2.00	2.00	1
355	2.00	1.00	2.00	2.00	2.00	2.00	1
356	2.00	1.00	2.00	2.00	2.00	2.00	1
357	2.00	1.00	2.00	2.00	2.00	2.00	1
358	1.00	1.00	1.00	1.00	1.00	2.00	1
359	2.00	1.00	2.00	2.00	2.00	2.00	1
360	2.00	1.00	2.00	2.00	2.00	2.00	1
361	2.00	1.00	2.00	2.00	2.00	2.00	1
362	1.00	1.00	1.00	1.00	1.00	2.00	1
363	2.00	1.00	2.00	1.00	2.00	2.00	1
364	2.00	1.00	2.00	2.00	2.00	2.00	1
365	1.00	2.00	2.00	1.00	1.00	2.00	1
366	2.00	1.00	2.00	2.00	2.00	2.00	1
367	2.00	1.00	1.00	2.00	2.00	2.00	1
368	2.00	1.00	1.00	1.00	1.00	2.00	1
369	2.00	1.00	2.00	2.00	2.00	2.00	1
370	2.00	1.00	2.00	2.00	2.00	2.00	1
371	2.00	1.00	2.00	2.00	2.00	2.00	1
372	2.00	1.00	1.00	2.00	2.00	2.00	1
373	2.00	1.00	2.00	1.00	2.00	2.00	1
374	2.00	1.00	2.00	2.00	2.00	2.00	1
375	2.00	1.00	1.00	1.00	2.00	2.00	1
376	2.00	1.00	2.00	2.00	2.00	2.00	1
377	2.00	1.00	2.00	1.00	2.00	2.00	1
378	2.00	2.00	2.00	2.00	2.00	1.00	1

	SN	Group	Gender	Age	Work	Work2	Work3	Sector
379	497.00	1.00	1.00	26.00	8.00	2.00	2.00	1.00
380	498.00	1.00	2.00	29.00	8.00	2.00	2.00	2.00
381	499.00	1.00	2.00	19.00	2.00	1.00	1.00	1.00
382	500.00	1.00	1.00	31.00	8.00	2.00	2.00	2.00
383	501.00	1.00	1.00	30.00	8.00	2.00	2.00	2.00
384	502.00	1.00	2.00	27.00	8.00	2.00	2.00	1.00
385	503.00	1.00	2.00	31.00	8.00	2.00	2.00	1.00
386	504.00	1.00	2.00	26.00	8.00	2.00	2.00	2.00
387	505.00	1.00	1.00	21.00	2.00	1.00	1.00	2.00
388	506.00	1.00	2.00	36.00	10.00	3.00	2.00	1.00
389	507.00	1.00	2.00	25.00	8.00	2.00	2.00	2.00
390	508.00	1.00	1.00	25.00	6.00	1.00	1.00	1.00
391	509.00	1.00	2.00	33.00	8.00	2.00	2.00	2.00
392	510.00	2.00	2.00	22.00	4.00	1.00	1.00	1.00
393	511.00	2.00	2.00	22.00	4.00	1.00	1.00	1.00
394	512.00	2.00	2.00	27.00	8.00	2.00	2.00	1.00
395	513.00	2.00	2.00	23.00	6.00	1.00	1.00	1.00
396	514.00	2.00	2.00	25.00	6.00	1.00	1.00	1.00
397	515.00	2.00	2.00	24.00	6.00	1.00	1.00	1.00
398	516.00	2.00	2.00	23.00	5.00	1.00	1.00	1.00
399	517.00	2.00	2.00	20.00	2.00	1.00	1.00	1.00
400	520.00	2.00	2.00	27.00	8.00	2.00	2.00	1.00
401	521.00	2.00	2.00	22.00	5.00	1.00	1.00	1.00
402	523.00	2.00	2.00	24.00	8.00	2.00	2.00	2.00
403	524.00	2.00	2.00	21.00	3.00	1.00	1.00	1.00
404	527.00	2.00	2.00	21.00	3.00	1.00	1.00	1.00
405	528.00	2.00	2.00	23.00	5.00	1.00	1.00	1.00
406	529.00	2.00	2.00	23.00	5.00	1.00	1.00	1.00
407	532.00	2.00	2.00	24.00	7.00	2.00	2.00	1.00
408	536.00	2.00	1.00	24.00	8.00	2.00	2.00	1.00
409	537.00	2.00	2.00	30.00	8.00	2.00	2.00	1.00
410	538.00	2.00	2.00	25.00	8.00	2.00	2.00	2.00
411	541.00	2.00	2.00	24.00	5.00	1.00	1.00	1.00
412	542.00	2.00	2.00	23.00	5.00	1.00	1.00	1.00
413	543.00	2.00	2.00	23.00	5.00	1.00	1.00	1.00
414	544.00	2.00	2.00	20.00	2.00	1.00	1.00	1.00
415	548.00	2.00	2.00	22.00	4.00	1.00	1.00	1.00
416	551.00	2.00	2.00	21.00	4.00	1.00	1.00	1.00
417	552.00	2.00	2.00	22.00	4.00	1.00	1.00	1.00
418	553.00	2.00	2.00	21.00	4.00	1.00	1.00	1.00
419	554.00	2.00	2.00	21.00	4.00	1.00	1.00	1.00
420	556.00	2.00	2.00	22.00	4.00	1.00	1.00	1.00

	Region	Nationality	M1	M2	M3	M4	M5	M6
379	2.00	1.00	1.00	1.00	1.00	1.00	2.00	1.00
380	1.00	1.00	1.00	1.00	1.00	1.00	2.00	2.00
381	4.00	1.00	1.00	1.00	1.00	1.00	1.00	1.00
382	1.00	2.00	1.00	2.00	2.00	1.00	1.00	2.00
383	3.00	1.00	1.00	1.00	1.00	1.00	1.00	1.00
384	1.00	1.00	2.00	2.00	2.00	2.00	2.00	1.00
385	1.00	1.00	1.00	1.00	2.00	1.00	1.00	1.00
386	2.00	1.00	1.00	2.00	1.00	2.00	1.00	1.00
387	1.00	1.00	1.00	1.00	1.00	1.00	1.00	1.00
388	1.00	1.00	2.00	1.00	2.00	2.00	1.00	1.00
389	4.00	1.00	1.00	1.00	1.00	1.00	1.00	1.00
390	1.00	1.00	1.00	1.00	1.00	1.00	1.00	1.00
391	5.00	2.00	1.00	2.00	2.00	1.00	1.00	2.00
392	1.00	1.00	1.00	1.00	1.00	1.00	1.00	1.00
393	1.00	1.00	2.00	1.00	2.00	1.00	1.00	1.00
394	4.00	1.00	1.00	1.00	1.00	1.00	2.00	2.00
395	2.00	1.00	1.00	2.00	1.00	1.00	2.00	1.00
396	1.00	1.00	1.00	1.00	1.00	1.00	1.00	1.00
397	1.00	1.00	2.00	2.00	2.00	2.00	1.00	1.00
398	3.00	1.00	1.00	2.00	2.00	1.00	1.00	1.00
399	1.00	1.00	1.00	1.00	2.00	1.00	1.00	1.00
400	3.00	1.00	1.00	1.00	2.00	1.00	1.00	2.00
401	1.00	1.00	1.00	1.00	1.00	1.00	2.00	1.00
402	1.00	1.00	1.00	1.00	1.00	1.00	1.00	1.00
403	3.00	1.00	1.00	1.00	1.00	1.00	2.00	1.00
404	3.00	1.00	1.00	1.00	1.00	1.00	1.00	1.00
405	1.00	1.00	1.00	1.00	2.00	1.00	2.00	1.00
406	1.00	1.00	1.00	1.00	1.00	1.00	1.00	2.00
407	5.00	1.00	1.00	1.00	1.00	1.00	1.00	2.00
408	4.00	1.00	1.00	2.00	2.00	1.00	2.00	1.00
409	2.00	1.00	1.00	1.00	2.00	1.00	2.00	2.00
410	1.00	1.00	1.00	1.00	1.00	1.00	2.00	1.00
411	2.00	1.00	1.00	1.00	2.00	1.00	1.00	1.00
412	2.00	1.00	1.00	1.00	2.00	1.00	1.00	2.00
413	2.00	1.00	1.00	1.00	2.00	1.00	1.00	2.00
414	1.00	1.00	1.00	1.00	1.00	1.00	1.00	1.00
415	2.00	1.00	2.00	1.00	2.00	1.00	1.00	2.00
416	1.00	1.00	1.00	1.00	2.00	1.00	1.00	1.00
417	1.00	1.00	1.00	1.00	1.00	1.00	1.00	1.00
418	1.00	1.00	1.00	1.00	1.00	1.00	1.00	1.00
419	4.00	1.00	1.00	2.00	2.00	2.00	1.00	2.00
420	1.00	1.00	1.00	1.00	2.00	2.00	2.00	1.00

	M7	M8	M9	M10	M11	M12	M13	M14
379	1.00	1.00	1.00	1.00	1.00	1.00	1.00	1.00
380	1.00	2.00	2.00	1.00	2.00	2.00	2.00	1.00
381	1.00	1.00	1.00	1.00	1.00	1.00	1.00	1.00
382	2.00	1.00	2.00	1.00	2.00	2.00	2.00	2.00
383	1.00	1.00	1.00	1.00	2.00	1.00	2.00	1.00
384	2.00	1.00	2.00	1.00	2.00	1.00	2.00	1.00
385	1.00	1.00	2.00	1.00	2.00	1.00	2.00	1.00
386	1.00	1.00	2.00	2.00	2.00	1.00	2.00	1.00
387	1.00	1.00	1.00	1.00	1.00	1.00	1.00	1.00
388	1.00	1.00	1.00	1.00	2.00	2.00	2.00	2.00
389	1.00	1.00	1.00	1.00	1.00	1.00	1.00	1.00
390	1.00	1.00	1.00	1.00	1.00	1.00	1.00	1.00
391	2.00	1.00	2.00	2.00	2.00	2.00	2.00	2.00
392	1.00	1.00	1.00	1.00	1.00	1.00	1.00	1.00
393	2.00	1.00	2.00	1.00	2.00	2.00	2.00	1.00
394	2.00	2.00	2.00	1.00	1.00	1.00	2.00	1.00
395	2.00	1.00	2.00	1.00	1.00	2.00	1.00	2.00
396	2.00	2.00	2.00	2.00	2.00	2.00	1.00	1.00
397	1.00	1.00	1.00	1.00	1.00	1.00	1.00	1.00
398	2.00	1.00	1.00	1.00	2.00	1.00	1.00	1.00
399	2.00	1.00	1.00	1.00	2.00	1.00	2.00	1.00
400	1.00	1.00	2.00	1.00	2.00	1.00	1.00	1.00
401	1.00	1.00	1.00	1.00	2.00	2.00	1.00	1.00
402	1.00	1.00	1.00	1.00	2.00	1.00	2.00	1.00
403	2.00	1.00	1.00	1.00	1.00	1.00	1.00	1.00
404	1.00	1.00	1.00	1.00	1.00	1.00	1.00	1.00
405	2.00	1.00	2.00	1.00	2.00	1.00	2.00	1.00
406	2.00	1.00	1.00	1.00	1.00	1.00	2.00	1.00
407	1.00	1.00	2.00	1.00	1.00	2.00	1.00	1.00
408	1.00	2.00	2.00	1.00	2.00	1.00	2.00	1.00
409	1.00	2.00	2.00	1.00	2.00	1.00	2.00	2.00
410	1.00	1.00	1.00	1.00	2.00	1.00	1.00	1.00
411	1.00	1.00	1.00	1.00	2.00	1.00	2.00	1.00
412	1.00	1.00	1.00	1.00	2.00	2.00	2.00	1.00
413	1.00	1.00	1.00	1.00	2.00	1.00	2.00	1.00
414	1.00	1.00	1.00	1.00	1.00	1.00	1.00	1.00
415	2.00	1.00	1.00	1.00	2.00	1.00	2.00	2.00
416	2.00	1.00	1.00	1.00	2.00	1.00	2.00	1.00
417	1.00	1.00	1.00	1.00	1.00	2.00	1.00	1.00
418	1.00	1.00	1.00	1.00	1.00	1.00	1.00	1.00
419	2.00	1.00	2.00	2.00	1.00	2.00	2.00	2.00
420	1.00	1.00	2.00	1.00	1.00	1.00	1.00	1.00

	M15	B1	B2	B3	B4	B5	B6	B7
379	1.00	1.00	1.00	2.00	1.00	2.00	1.00	1.00
380	1.00	2.00	1.00	1.00	2.00	2.00	2.00	2.00
381	1.00	1.00	1.00	1.00	1.00	1.00	1.00	1.00
382	2.00	2.00	2.00	2.00	2.00	2.00	2.00	2.00
383	1.00	1.00	1.00	1.00	1.00	2.00	1.00	1.00
384	2.00	2.00	1.00	2.00	2.00	1.00	2.00	2.00
385	2.00	2.00	1.00	1.00	1.00	2.00	1.00	1.00
386	2.00	1.00	1.00	2.00	1.00	1.00	2.00	1.00
387	1.00	1.00	1.00	1.00	1.00	1.00	1.00	1.00
388	2.00	2.00	2.00	2.00	1.00	1.00	1.00	1.00
389	1.00	2.00	1.00	1.00	1.00	2.00	2.00	1.00
390	1.00	1.00	1.00	1.00	1.00	1.00	1.00	1.00
391	2.00	2.00	2.00	1.00	1.00	1.00	2.00	2.00
392	1.00	2.00	2.00	2.00	2.00	2.00	2.00	2.00
393	2.00	1.00	1.00	2.00	1.00	1.00	2.00	2.00
394	1.00	1.00	1.00	1.00	1.00	2.00	2.00	2.00
395	1.00	1.00	2.00	1.00	2.00	1.00	2.00	1.00
396	1.00	2.00	2.00	2.00	1.00	2.00	2.00	2.00
397	1.00	2.00	1.00	2.00	1.00	1.00	1.00	1.00
398	1.00	1.00	1.00	1.00	1.00	2.00	1.00	1.00
399	2.00	1.00	2.00	2.00	1.00	1.00	2.00	2.00
400	1.00	1.00	1.00	2.00	1.00	1.00	1.00	1.00
401	2.00	1.00	1.00	1.00	1.00	2.00	2.00	2.00
402	1.00	2.00	2.00	2.00	1.00	2.00	2.00	2.00
403	1.00	1.00	1.00	1.00	2.00	2.00	2.00	2.00
404	1.00	1.00	1.00	1.00	1.00	1.00	1.00	1.00
405	1.00	2.00	2.00	2.00	2.00	2.00	2.00	2.00
406	1.00	2.00	1.00	1.00	1.00	1.00	1.00	1.00
407	1.00	1.00	2.00	2.00	1.00	2.00	1.00	1.00
408	2.00	2.00	1.00	1.00	2.00	1.00	2.00	2.00
409	2.00	1.00	1.00	2.00	2.00	2.00	2.00	2.00
410	1.00	1.00	1.00	1.00	1.00	2.00	1.00	1.00
411	1.00	2.00	1.00	2.00	2.00	2.00	2.00	2.00
412	1.00	2.00	2.00	2.00	2.00	2.00	2.00	2.00
413	1.00	2.00	2.00	2.00	1.00	1.00	2.00	2.00
414	1.00	2.00	2.00	1.00	2.00	1.00	2.00	2.00
415	1.00	1.00	1.00	2.00	1.00	2.00	2.00	2.00
416	1.00	1.00	2.00	2.00	1.00	2.00	1.00	1.00
417	2.00	1.00	2.00	1.00	1.00	1.00	1.00	1.00
418	1.00	1.00	1.00	1.00	1.00	1.00	1.00	1.00
419	2.00	2.00	1.00	1.00	2.00	2.00	2.00	2.00
420	1.00	2.00	1.00	2.00	2.00	1.00	1.00	2.00

	B8	B9	B10	B11	B12	B13	B14	W1
379	2.00	1.00	2.00	1.00	1.00	2.00	1.00	1.00
380	2.00	2.00	2.00	2.00	2.00	2.00	2.00	2.00
381	1.00	1.00	1.00	1.00	1.00	1.00	1.00	1.00
382	2.00	2.00	2.00	1.00	2.00	2.00	2.00	2.00
383	1.00	1.00	1.00	1.00	1.00	1.00	1.00	1.00
384	2.00	2.00	2.00	2.00	2.00	2.00	2.00	2.00
385	1.00	2.00	2.00	1.00	2.00	2.00	1.00	2.00
386	1.00	1.00	1.00	1.00	1.00	2.00	1.00	2.00
387	1.00	1.00	1.00	1.00	1.00	1.00	1.00	1.00
388	1.00	1.00	2.00	1.00	1.00	2.00	1.00	2.00
389	2.00	1.00	1.00	1.00	2.00	2.00	2.00	1.00
390	1.00	1.00	1.00	1.00	1.00	1.00	1.00	1.00
391	2.00	1.00	2.00	2.00	2.00	2.00	1.00	2.00
392	2.00	2.00	2.00	2.00	2.00	2.00	2.00	1.00
393	1.00	1.00	1.00	1.00	1.00	1.00	1.00	2.00
394	1.00	2.00	2.00	2.00	2.00	2.00	2.00	1.00
395	1.00	2.00	1.00	2.00	1.00	2.00	1.00	1.00
396	2.00	2.00	2.00	2.00	2.00	2.00	2.00	2.00
397	2.00	1.00	1.00	1.00	2.00	2.00	1.00	1.00
398	2.00	2.00	2.00	1.00	2.00	1.00	2.00	1.00
399	2.00	2.00	1.00	1.00	2.00	2.00	1.00	2.00
400	1.00	1.00	2.00	1.00	1.00	2.00	1.00	2.00
401	2.00	2.00	1.00	1.00	2.00	2.00	2.00	2.00
402	2.00	1.00	2.00	2.00	2.00	2.00	2.00	2.00
403	2.00	2.00	1.00	1.00	2.00	2.00	2.00	1.00
404	1.00	1.00	1.00	1.00	1.00	1.00	1.00	1.00
405	2.00	1.00	1.00	1.00	2.00	1.00	2.00	1.00
406	1.00	2.00	1.00	1.00	1.00	2.00	1.00	2.00
407	1.00	1.00	1.00	2.00	1.00	1.00	1.00	1.00
408	2.00	2.00	2.00	2.00	2.00	2.00	1.00	2.00
409	2.00	2.00	2.00	1.00	1.00	2.00	2.00	2.00
410	1.00	2.00	1.00	2.00	2.00	2.00	2.00	2.00
411	2.00	2.00	2.00	2.00	2.00	2.00	2.00	2.00
412	2.00	2.00	2.00	2.00	2.00	2.00	2.00	1.00
413	2.00	1.00	1.00	1.00	2.00	2.00	2.00	2.00
414	2.00	2.00	2.00	1.00	2.00	2.00	2.00	1.00
415	2.00	2.00	2.00	2.00	2.00	2.00	2.00	1.00
416	2.00	1.00	1.00	1.00	1.00	1.00	1.00	2.00
417	1.00	1.00	1.00	1.00	1.00	1.00	1.00	2.00
418	1.00	1.00	1.00	1.00	1.00	1.00	1.00	1.00
419	2.00	2.00	2.00	1.00	1.00	1.00	1.00	1.00
420	1.00	2.00	1.00	1.00	2.00	1.00	1.00	1.00

	W2	W3	W4	W5	W6	E1	V1	V2
379	1.00	1.00	1.00	1.00	1.00	1.00	1.00	1.00
380	2.00	2.00	2.00	2.00	2.00	1.00	2.00	2.00
381	1.00	1.00	2.00	1.00	2.00	1.00	2.00	2.00
382	2.00	1.00	2.00	2.00	2.00	1.00	2.00	2.00
383	1.00	2.00	2.00	2.00	2.00	1.00	2.00	2.00
384	2.00	2.00	2.00	2.00	2.00	1.00	2.00	2.00
385	2.00	1.00	1.00	1.00	2.00	1.00	2.00	2.00
386	2.00	1.00	1.00	1.00	2.00	1.00	2.00	2.00
387	1.00	1.00	1.00	1.00	1.00	1.00	1.00	1.00
388	2.00	1.00	2.00	2.00	2.00	1.00	2.00	2.00
389	1.00	2.00	1.00	2.00	2.00	1.00	2.00	2.00
390	1.00	1.00	1.00	1.00	1.00	1.00	2.00	1.00
391	2.00	1.00	2.00	1.00	2.00	1.00	2.00	2.00
392	1.00	1.00	1.00	1.00	1.00	1.00	2.00	2.00
393	2.00	1.00	2.00	1.00	2.00	1.00	2.00	2.00
394	1.00	1.00	1.00	1.00	1.00	1.00	2.00	2.00
395	1.00	1.00	2.00	1.00	2.00	1.00	2.00	2.00
396	2.00	1.00	1.00	2.00	2.00	1.00	2.00	2.00
397	2.00	2.00	1.00	2.00	2.00	1.00	2.00	1.00
398	2.00	2.00	1.00	1.00	2.00	1.00	2.00	2.00
399	2.00	1.00	2.00	1.00	1.00	1.00	2.00	2.00
400	2.00	1.00	2.00	2.00	2.00	1.00	2.00	2.00
401	2.00	1.00	2.00	1.00	2.00	1.00	2.00	2.00
402	2.00	1.00	2.00	2.00	2.00	1.00	2.00	2.00
403	1.00	1.00	1.00	1.00	1.00	1.00	2.00	2.00
404	1.00	1.00	1.00	1.00	1.00	1.00	1.00	1.00
405	2.00	1.00	1.00	2.00	2.00	1.00	2.00	2.00
406	2.00	1.00	1.00	1.00	2.00	1.00	2.00	2.00
407	1.00	2.00	1.00	1.00	1.00	1.00	2.00	2.00
408	2.00	1.00	1.00	1.00	2.00	1.00	2.00	2.00
409	2.00	1.00	2.00	2.00	2.00	1.00	2.00	2.00
410	2.00	1.00	2.00	2.00	1.00	1.00	2.00	2.00
411	2.00	1.00	2.00	1.00	1.00	1.00	2.00	2.00
412	1.00	2.00	1.00	1.00	2.00	1.00	2.00	2.00
413	2.00	1.00	2.00	1.00	2.00	1.00	2.00	2.00
414	1.00	1.00	2.00	2.00	1.00	1.00	2.00	1.00
415	2.00	1.00	2.00	1.00	2.00	1.00	2.00	2.00
416	2.00	1.00	1.00	1.00	2.00	1.00	2.00	1.00
417	1.00	2.00	2.00	1.00	2.00	1.00	1.00	1.00
418	1.00	1.00	1.00	2.00	2.00	1.00	2.00	2.00
419	2.00	1.00	1.00	1.00	1.00	1.00	2.00	1.00
420	2.00	1.00	2.00	1.00	2.00	1.00	2.00	1.00

	V3	V4	V5	V6	E2	E3	E4	C1
379	1.00	2.00	2.00	1.00	1.00	2.00	1.00	2.00
380	1.00	2.00	2.00	2.00	1.00	2.00	1.00	2.00
381	2.00	2.00	2.00	2.00	1.00	2.00	2.00	2.00
382	2.00	1.00	2.00	2.00	1.00	2.00	2.00	2.00
383	1.00	2.00	1.00	1.00	1.00	2.00	1.00	2.00
384	1.00	2.00	2.00	2.00	1.00	2.00	2.00	2.00
385	1.00	2.00	2.00	2.00	1.00	2.00	2.00	2.00
386	1.00	2.00	1.00	1.00	1.00	2.00	1.00	2.00
387	1.00	1.00	1.00	1.00	1.00	1.00	1.00	1.00
388	1.00	2.00	2.00	1.00	1.00	1.00	1.00	2.00
389	2.00	2.00	2.00	2.00	1.00	2.00	1.00	2.00
390	2.00	1.00	2.00	1.00	1.00	2.00	1.00	2.00
391	1.00	2.00	1.00	2.00	1.00	1.00	2.00	2.00
392	2.00	2.00	1.00	2.00	1.00	2.00	2.00	2.00
393	2.00	2.00	2.00	2.00	1.00	2.00	2.00	2.00
394	1.00	1.00	1.00	1.00	1.00	2.00	1.00	2.00
395	2.00	2.00	2.00	1.00	1.00	2.00	2.00	2.00
396	1.00	2.00	1.00	2.00	1.00	2.00	2.00	2.00
397	1.00	1.00	2.00	1.00	1.00	2.00	2.00	1.00
398	1.00	2.00	2.00	2.00	1.00	2.00	2.00	2.00
399	2.00	2.00	1.00	1.00	1.00	2.00	2.00	2.00
400	2.00	2.00	1.00	2.00	1.00	2.00	2.00	2.00
401	2.00	2.00	2.00	1.00	1.00	2.00	2.00	2.00
402	1.00	2.00	2.00	2.00	1.00	2.00	2.00	2.00
403	2.00	1.00	2.00	2.00	1.00	2.00	1.00	2.00
404	1.00	1.00	1.00	1.00	1.00	1.00	1.00	1.00
405	2.00	2.00	2.00	1.00	1.00	2.00	2.00	2.00
406	1.00	1.00	1.00	1.00	1.00	2.00	2.00	2.00
407	1.00	2.00	1.00	2.00	1.00	2.00	1.00	2.00
408	1.00	1.00	2.00	2.00	1.00	2.00	1.00	2.00
409	1.00	1.00	2.00	2.00	1.00	2.00	1.00	2.00
410	2.00	1.00	2.00	2.00	1.00	2.00	1.00	2.00
411	1.00	2.00	1.00	2.00	1.00	2.00	1.00	2.00
412	1.00	2.00	1.00	1.00	1.00	2.00	2.00	2.00
413	2.00	2.00	1.00	2.00	1.00	2.00	2.00	2.00
414	1.00	2.00	1.00	1.00	1.00	2.00	2.00	2.00
415	1.00	2.00	1.00	2.00	1.00	2.00	2.00	2.00
416	2.00	1.00	2.00	2.00	1.00	2.00	2.00	2.00
417	2.00	2.00	1.00	2.00	1.00	2.00	1.00	2.00
418	2.00	1.00	2.00	2.00	1.00	2.00	1.00	2.00
419	1.00	2.00	1.00	1.00	1.00	2.00	2.00	1.00
420	1.00	2.00	1.00	2.00	1.00	2.00	1.00	1.00

	C2	C3	C4	C5	C6	C7	filter_$
379	2.00	1.00	1.00	1.00	1.00	2.00	1
380	2.00	1.00	2.00	2.00	2.00	2.00	1
381	2.00	2.00	1.00	1.00	2.00	2.00	1
382	2.00	1.00	1.00	2.00	2.00	2.00	1
383	2.00	1.00	1.00	2.00	2.00	2.00	1
384	2.00	1.00	2.00	2.00	2.00	2.00	1
385	2.00	1.00	2.00	2.00	2.00	2.00	1
386	2.00	1.00	1.00	2.00	2.00	2.00	1
387	1.00	1.00	1.00	1.00	1.00	2.00	1
388	1.00	1.00	1.00	2.00	2.00	2.00	1
389	2.00	1.00	2.00	2.00	2.00	2.00	1
390	2.00	2.00	2.00	2.00	2.00	1.00	1
391	2.00	1.00	2.00	2.00	2.00	2.00	1
392	2.00	1.00	1.00	2.00	2.00	2.00	1
393	2.00	2.00	2.00	2.00	2.00	1.00	1
394	2.00	1.00	2.00	1.00	2.00	2.00	1
395	2.00	2.00	2.00	1.00	2.00	1.00	1
396	2.00	1.00	2.00	2.00	2.00	1.00	1
397	1.00	1.00	1.00	1.00	1.00	1.00	1
398	2.00	1.00	2.00	2.00	2.00	2.00	1
399	2.00	1.00	2.00	2.00	2.00	2.00	1
400	2.00	1.00	1.00	1.00	2.00	2.00	1
401	2.00	2.00	2.00	2.00	2.00	1.00	1
402	2.00	1.00	2.00	2.00	2.00	2.00	1
403	2.00	1.00	2.00	2.00	2.00	2.00	1
404	1.00	1.00	1.00	1.00	1.00	1.00	1
405	1.00	1.00	2.00	2.00	2.00	2.00	1
406	2.00	1.00	2.00	2.00	2.00	1.00	1
407	1.00	1.00	2.00	1.00	1.00	2.00	1
408	2.00	1.00	2.00	2.00	2.00	2.00	1
409	2.00	2.00	2.00	2.00	2.00	1.00	1
410	2.00	1.00	2.00	2.00	2.00	2.00	1
411	2.00	1.00	1.00	2.00	2.00	2.00	1
412	2.00	1.00	2.00	2.00	2.00	2.00	1
413	2.00	1.00	2.00	2.00	2.00	1.00	1
414	2.00	1.00	2.00	1.00	2.00	2.00	1
415	2.00	1.00	2.00	2.00	2.00	1.00	1
416	2.00	1.00	1.00	2.00	2.00	2.00	1
417	2.00	2.00	2.00	2.00	2.00	2.00	1
418	2.00	2.00	1.00	2.00	2.00	2.00	1
419	1.00	2.00	1.00	1.00	1.00	1.00	1
420	2.00	1.00	2.00	1.00	1.00	2.00	1

	SN	Group	Gender	Age	Work	Work2	Work3	Sector
421	557.00	2.00	2.00	21.00	4.00	1.00	1.00	1.00
422	558.00	2.00	2.00	21.00	3.00	1.00	1.00	1.00
423	559.00	2.00	2.00	23.00	5.00	1.00	1.00	1.00
424	561.00	2.00	2.00	20.00	3.00	1.00	1.00	1.00
425	562.00	2.00	2.00	21.00	4.00	1.00	1.00	1.00
426	563.00	2.00	2.00	20.00	3.00	1.00	1.00	1.00
427	564.00	2.00	2.00	22.00	4.00	1.00	1.00	1.00
428	565.00	2.00	2.00	21.00	4.00	1.00	1.00	1.00
429	566.00	2.00	2.00	22.00	4.00	1.00	1.00	1.00
430	568.00	2.00	1.00	20.00	2.00	1.00	1.00	1.00
431	570.00	2.00	2.00	21.00	4.00	1.00	1.00	1.00
432	572.00	2.00	2.00	21.00	4.00	1.00	1.00	1.00
433	573.00	2.00	2.00	23.00	3.00	1.00	1.00	1.00
434	575.00	2.00	2.00	22.00	4.00	1.00	1.00	1.00
435	576.00	2.00	2.00	23.00	3.00	1.00	1.00	1.00
436	577.00	2.00	2.00	26.00	8.00	2.00	2.00	1.00
437	579.00	2.00	2.00	22.00	4.00	1.00	1.00	1.00
438	580.00	2.00	1.00	22.00	4.00	1.00	1.00	1.00
439	581.00	2.00	2.00	24.00	5.00	1.00	1.00	1.00
440	582.00	2.00	2.00	21.00	4.00	1.00	1.00	1.00
441	583.00	2.00	2.00	21.00	4.00	1.00	1.00	1.00
442	584.00	2.00	2.00	26.00	8.00	2.00	2.00	1.00
443	587.00	2.00	1.00	27.00	8.00	2.00	2.00	2.00
444	589.00	2.00	2.00	25.00	8.00	2.00	2.00	2.00
445	590.00	2.00	2.00	50.00	8.00	2.00	2.00	2.00
446	591.00	2.00	2.00	22.00	4.00	1.00	1.00	1.00
447	592.00	2.00	1.00	30.00	7.00	2.00	2.00	2.00
448	593.00	2.00	2.00	30.00	8.00	2.00	2.00	2.00
449	594.00	2.00	2.00	26.00	8.00	2.00	2.00	1.00
450	596.00	2.00	2.00	22.00	4.00	1.00	1.00	1.00
451	597.00	2.00	1.00	26.00	8.00	2.00	2.00	2.00
452	598.00	2.00	2.00	27.00	8.00	2.00	2.00	1.00
453	599.00	2.00	2.00	23.00	6.00	1.00	1.00	1.00
454	600.00	2.00	2.00	26.00	8.00	2.00	2.00	1.00
455	601.00	2.00	2.00	26.00	8.00	2.00	2.00	2.00
456	604.00	2.00	2.00	21.00	3.00	1.00	1.00	1.00
457	606.00	2.00	2.00	21.00	3.00	1.00	1.00	1.00
458	607.00	2.00	2.00	21.00	3.00	1.00	1.00	1.00
459	608.00	2.00	2.00	25.00	6.00	1.00	1.00	1.00
460	609.00	2.00	2.00	21.00	3.00	1.00	1.00	1.00
461	611.00	2.00	2.00	25.00	8.00	2.00	2.00	1.00
462	612.00	2.00	1.00	31.00	8.00	2.00	2.00	2.00

	Region	Nationality	M1	M2	M3	M4	M5	M6
421	1.00	1.00	1.00	1.00	2.00	1.00	2.00	1.00
422	1.00	1.00	1.00	1.00	2.00	1.00	1.00	1.00
423	5.00	1.00	1.00	1.00	1.00	1.00	1.00	1.00
424	1.00	1.00	1.00	1.00	2.00	1.00	1.00	1.00
425	1.00	1.00	2.00	1.00	1.00	1.00	1.00	1.00
426	1.00	1.00	1.00	1.00	1.00	1.00	2.00	1.00
427	5.00	1.00	1.00	1.00	2.00	1.00	1.00	1.00
428	1.00	1.00	1.00	2.00	2.00	1.00	1.00	1.00
429	1.00	1.00	1.00	1.00	2.00	1.00	1.00	1.00
430	2.00	1.00	1.00	1.00	2.00	1.00	1.00	1.00
431	1.00	2.00	1.00	1.00	1.00	1.00	1.00	2.00
432	4.00	1.00	1.00	1.00	1.00	1.00	1.00	2.00
433	2.00	1.00	1.00	1.00	2.00	1.00	1.00	1.00
434	5.00	1.00	1.00	1.00	1.00	1.00	1.00	1.00
435	2.00	1.00	1.00	1.00	2.00	1.00	1.00	1.00
436	2.00	1.00	1.00	2.00	2.00	2.00	2.00	1.00
437	2.00	1.00	2.00	2.00	1.00	2.00	2.00	2.00
438	3.00	1.00	1.00	1.00	1.00	1.00	2.00	1.00
439	3.00	1.00	1.00	1.00	2.00	1.00	1.00	1.00
440	1.00	1.00	1.00	1.00	2.00	1.00	2.00	1.00
441	1.00	1.00	1.00	2.00	1.00	1.00	2.00	1.00
442	2.00	1.00	1.00	1.00	1.00	1.00	1.00	1.00
443	1.00	1.00	1.00	1.00	2.00	2.00	1.00	1.00
444	2.00	1.00	2.00	2.00	2.00	2.00	2.00	1.00
445	1.00	1.00	1.00	1.00	1.00	1.00	2.00	1.00
446	1.00	1.00	1.00	1.00	1.00	1.00	1.00	1.00
447	4.00	2.00	1.00	1.00	2.00	1.00	1.00	1.00
448	4.00	1.00	1.00	1.00	1.00	1.00	1.00	1.00
449	4.00	1.00	1.00	1.00	2.00	1.00	1.00	1.00
450	1.00	1.00	1.00	1.00	2.00	1.00	1.00	1.00
451	1.00	1.00	1.00	1.00	1.00	1.00	1.00	1.00
452	4.00	1.00	2.00	1.00	2.00	1.00	1.00	1.00
453	1.00	1.00	1.00	1.00	1.00	1.00	1.00	1.00
454	4.00	1.00	1.00	1.00	1.00	2.00	2.00	1.00
455	4.00	1.00	2.00	2.00	1.00	2.00	1.00	2.00
456	4.00	1.00	1.00	1.00	1.00	1.00	1.00	1.00
457	1.00	1.00	2.00	1.00	1.00	1.00	2.00	1.00
458	1.00	1.00	1.00	1.00	1.00	2.00	1.00	1.00
459	1.00	1.00	1.00	1.00	2.00	1.00	1.00	1.00
460	1.00	1.00	1.00	1.00	2.00	1.00	1.00	1.00
461	2.00	1.00	1.00	1.00	2.00	1.00	2.00	2.00
462	1.00	1.00	1.00	1.00	1.00	1.00	1.00	1.00

	M7	M8	M9	M10	M11	M12	M13	M14
421	1.00	1.00	1.00	1.00	1.00	2.00	1.00	1.00
422	2.00	1.00	1.00	1.00	1.00	1.00	1.00	1.00
423	1.00	1.00	2.00	1.00	2.00	1.00	2.00	1.00
424	1.00	1.00	2.00	1.00	1.00	1.00	1.00	1.00
425	1.00	1.00	1.00	1.00	1.00	2.00	2.00	1.00
426	2.00	1.00	1.00	1.00	1.00	1.00	1.00	1.00
427	2.00	1.00	1.00	1.00	1.00	2.00	2.00	1.00
428	1.00	1.00	1.00	2.00	2.00	2.00	2.00	1.00
429	1.00	1.00	1.00	2.00	2.00	1.00	1.00	1.00
430	1.00	1.00	1.00	1.00	2.00	1.00	2.00	1.00
431	1.00	1.00	1.00	1.00	1.00	1.00	1.00	1.00
432	2.00	1.00	1.00	1.00	2.00	1.00	2.00	1.00
433	2.00	1.00	1.00	1.00	2.00	1.00	1.00	1.00
434	1.00	1.00	1.00	1.00	1.00	1.00	1.00	1.00
435	1.00	1.00	2.00	1.00	2.00	1.00	2.00	1.00
436	1.00	1.00	1.00	1.00	1.00	1.00	1.00	1.00
437	2.00	2.00	1.00	2.00	2.00	2.00	2.00	2.00
438	1.00	1.00	2.00	2.00	2.00	1.00	1.00	1.00
439	1.00	1.00	1.00	1.00	1.00	2.00	1.00	1.00
440	1.00	1.00	1.00	1.00	2.00	1.00	2.00	1.00
441	2.00	1.00	2.00	2.00	2.00	2.00	1.00	1.00
442	1.00	1.00	1.00	1.00	1.00	1.00	2.00	1.00
443	2.00	2.00	2.00	1.00	2.00	1.00	2.00	1.00
444	2.00	2.00	2.00	2.00	2.00	1.00	2.00	1.00
445	1.00	1.00	2.00	1.00	1.00	1.00	1.00	1.00
446	1.00	1.00	2.00	1.00	1.00	2.00	1.00	1.00
447	1.00	1.00	1.00	1.00	2.00	1.00	1.00	1.00
448	1.00	1.00	1.00	1.00	1.00	1.00	1.00	1.00
449	1.00	1.00	1.00	1.00	2.00	1.00	2.00	1.00
450	1.00	1.00	2.00	2.00	1.00	1.00	2.00	1.00
451	1.00	2.00	1.00	1.00	2.00	1.00	2.00	2.00
452	1.00	1.00	2.00	2.00	2.00	2.00	2.00	1.00
453	2.00	1.00	1.00	1.00	1.00	1.00	1.00	1.00
454	2.00	1.00	1.00	1.00	1.00	2.00	2.00	2.00
455	2.00	2.00	2.00	2.00	2.00	1.00	2.00	1.00
456	1.00	1.00	1.00	1.00	1.00	1.00	2.00	1.00
457	1.00	1.00	2.00	2.00	1.00	2.00	1.00	1.00
458	2.00	1.00	1.00	1.00	2.00	1.00	1.00	1.00
459	2.00	1.00	1.00	2.00	2.00	1.00	2.00	1.00
460	1.00	1.00	2.00	1.00	2.00	1.00	2.00	1.00
461	1.00	1.00	1.00	1.00	2.00	1.00	2.00	2.00
462	1.00	1.00	1.00	1.00	1.00	1.00	1.00	1.00

	M15	B1	B2	B3	B4	B5	B6	B7
421	1.00	1.00	2.00	1.00	2.00	2.00	2.00	1.00
422	2.00	2.00	1.00	2.00	1.00	2.00	2.00	2.00
423	1.00	2.00	2.00	2.00	1.00	2.00	1.00	2.00
424	1.00	1.00	1.00	1.00	2.00	1.00	2.00	1.00
425	1.00	1.00	1.00	2.00	2.00	1.00	1.00	1.00
426	1.00	1.00	1.00	1.00	1.00	2.00	2.00	2.00
427	2.00	2.00	1.00	1.00	1.00	1.00	1.00	1.00
428	1.00	2.00	2.00	2.00	2.00	2.00	1.00	1.00
429	1.00	1.00	1.00	1.00	1.00	1.00	1.00	1.00
430	1.00	1.00	2.00	2.00	2.00	2.00	2.00	2.00
431	1.00	1.00	2.00	2.00	1.00	1.00	2.00	2.00
432	1.00	1.00	1.00	1.00	1.00	2.00	2.00	2.00
433	1.00	1.00	1.00	2.00	1.00	2.00	1.00	1.00
434	1.00	1.00	2.00	1.00	1.00	2.00	1.00	2.00
435	1.00	1.00	2.00	2.00	2.00	1.00	2.00	2.00
436	1.00	1.00	2.00	1.00	1.00	2.00	2.00	2.00
437	2.00	2.00	2.00	1.00	1.00	2.00	1.00	1.00
438	1.00	1.00	2.00	1.00	1.00	2.00	1.00	1.00
439	1.00	1.00	2.00	2.00	1.00	1.00	1.00	1.00
440	1.00	1.00	2.00	2.00	1.00	2.00	2.00	2.00
441	1.00	1.00	1.00	2.00	1.00	2.00	1.00	2.00
442	1.00	2.00	1.00	1.00	1.00	2.00	2.00	2.00
443	2.00	2.00	2.00	2.00	1.00	2.00	2.00	2.00
444	2.00	1.00	1.00	1.00	1.00	1.00	2.00	2.00
445	1.00	2.00	1.00	1.00	1.00	1.00	2.00	1.00
446	1.00	1.00	1.00	1.00	1.00	2.00	1.00	1.00
447	1.00	1.00	2.00	2.00	1.00	2.00	1.00	1.00
448	1.00	1.00	1.00	2.00	1.00	1.00	1.00	1.00
449	1.00	2.00	1.00	1.00	1.00	1.00	2.00	2.00
450	2.00	2.00	1.00	1.00	1.00	1.00	2.00	2.00
451	1.00	2.00	2.00	1.00	1.00	2.00	2.00	2.00
452	1.00	2.00	2.00	2.00	2.00	2.00	2.00	2.00
453	1.00	1.00	1.00	1.00	1.00	1.00	1.00	1.00
454	1.00	1.00	1.00	1.00	1.00	2.00	2.00	2.00
455	2.00	1.00	1.00	1.00	1.00	1.00	1.00	1.00
456	1.00	1.00	1.00	2.00	1.00	2.00	2.00	2.00
457	1.00	1.00	2.00	2.00	1.00	1.00	1.00	1.00
458	1.00	1.00	2.00	1.00	1.00	2.00	1.00	1.00
459	2.00	1.00	1.00	1.00	1.00	2.00	2.00	2.00
460	1.00	1.00	2.00	2.00	1.00	2.00	1.00	1.00
461	1.00	2.00	1.00	2.00	1.00	2.00	2.00	2.00
462	1.00	2.00	2.00	2.00	1.00	1.00	2.00	1.00

	B8	B9	B10	B11	B12	B13	B14	W1
421	2.00	2.00	2.00	1.00	2.00	2.00	2.00	1.00
422	2.00	1.00	1.00	1.00	1.00	2.00	1.00	1.00
423	2.00	2.00	1.00	1.00	2.00	2.00	2.00	1.00
424	1.00	1.00	1.00	1.00	1.00	1.00	1.00	1.00
425	1.00	1.00	1.00	1.00	2.00	2.00	1.00	1.00
426	2.00	1.00	1.00	1.00	1.00	1.00	1.00	1.00
427	1.00	1.00	1.00	1.00	1.00	2.00	1.00	1.00
428	2.00	1.00	2.00	1.00	2.00	1.00	2.00	1.00
429	1.00	2.00	1.00	1.00	2.00	1.00	1.00	1.00
430	2.00	2.00	2.00	2.00	2.00	2.00	1.00	1.00
431	1.00	1.00	1.00	1.00	1.00	1.00	2.00	1.00
432	2.00	2.00	2.00	2.00	2.00	1.00	1.00	1.00
433	2.00	1.00	1.00	1.00	2.00	1.00	2.00	1.00
434	1.00	1.00	2.00	1.00	2.00	1.00	2.00	1.00
435	2.00	1.00	2.00	2.00	2.00	1.00	2.00	2.00
436	1.00	1.00	1.00	1.00	1.00	1.00	1.00	1.00
437	1.00	1.00	1.00	2.00	2.00	1.00	1.00	1.00
438	2.00	2.00	1.00	1.00	2.00	2.00	2.00	2.00
439	2.00	2.00	1.00	1.00	1.00	2.00	2.00	2.00
440	1.00	2.00	1.00	1.00	2.00	2.00	2.00	2.00
441	1.00	2.00	2.00	1.00	2.00	1.00	1.00	1.00
442	1.00	2.00	2.00	2.00	1.00	1.00	1.00	2.00
443	2.00	2.00	2.00	2.00	2.00	2.00	2.00	2.00
444	2.00	1.00	1.00	2.00	2.00	2.00	1.00	2.00
445	1.00	1.00	1.00	1.00	1.00	2.00	1.00	1.00
446	2.00	2.00	2.00	1.00	1.00	1.00	2.00	1.00
447	1.00	1.00	1.00	1.00	2.00	2.00	1.00	1.00
448	2.00	1.00	1.00	1.00	1.00	2.00	1.00	1.00
449	2.00	2.00	1.00	1.00	1.00	2.00	2.00	2.00
450	1.00	1.00	1.00	1.00	1.00	1.00	1.00	1.00
451	2.00	2.00	2.00	2.00	2.00	2.00	2.00	1.00
452	2.00	2.00	2.00	1.00	2.00	2.00	2.00	2.00
453	1.00	2.00	1.00	1.00	1.00	2.00	1.00	2.00
454	2.00	2.00	2.00	2.00	1.00	1.00	2.00	1.00
455	1.00	2.00	2.00	2.00	1.00	2.00	1.00	2.00
456	2.00	1.00	2.00	1.00	2.00	1.00	1.00	2.00
457	1.00	1.00	2.00	2.00	2.00	2.00	2.00	1.00
458	2.00	2.00	1.00	1.00	1.00	1.00	2.00	1.00
459	2.00	1.00	1.00	1.00	1.00	2.00	1.00	1.00
460	2.00	2.00	1.00	1.00	2.00	2.00	2.00	1.00
461	2.00	2.00	1.00	1.00	2.00	2.00	1.00	1.00
462	1.00	1.00	1.00	1.00	2.00	2.00	2.00	1.00

	W2	W3	W4	W5	W6	E1	V1	V2
421	2.00	1.00	1.00	1.00	2.00	1.00	2.00	2.00
422	1.00	2.00	2.00	1.00	2.00	1.00	2.00	2.00
423	2.00	2.00	2.00	1.00	2.00	1.00	2.00	2.00
424	1.00	1.00	2.00	2.00	2.00	1.00	2.00	2.00
425	1.00	1.00	1.00	1.00	1.00	1.00	2.00	2.00
426	1.00	1.00	1.00	1.00	1.00	1.00	2.00	2.00
427	1.00	1.00	2.00	2.00	2.00	1.00	2.00	2.00
428	1.00	2.00	1.00	1.00	1.00	1.00	2.00	2.00
429	1.00	1.00	1.00	1.00	2.00	1.00	2.00	2.00
430	1.00	1.00	1.00	1.00	1.00	1.00	2.00	2.00
431	1.00	2.00	2.00	1.00	1.00	1.00	2.00	2.00
432	1.00	1.00	1.00	1.00	1.00	1.00	2.00	2.00
433	2.00	1.00	1.00	1.00	1.00	1.00	2.00	2.00
434	1.00	1.00	1.00	1.00	1.00	1.00	1.00	1.00
435	2.00	1.00	1.00	1.00	1.00	1.00	2.00	2.00
436	1.00	2.00	2.00	2.00	1.00	1.00	1.00	2.00
437	1.00	2.00	1.00	1.00	2.00	1.00	2.00	2.00
438	1.00	1.00	2.00	1.00	2.00	1.00	2.00	2.00
439	2.00	1.00	1.00	1.00	1.00	1.00	2.00	2.00
440	2.00	1.00	1.00	1.00	1.00	1.00	2.00	2.00
441	2.00	1.00	2.00	1.00	2.00	1.00	2.00	2.00
442	2.00	1.00	1.00	1.00	2.00	1.00	2.00	2.00
443	2.00	1.00	2.00	2.00	2.00	1.00	2.00	2.00
444	2.00	2.00	2.00	2.00	2.00	1.00	2.00	2.00
445	1.00	1.00	2.00	2.00	2.00	1.00	2.00	2.00
446	2.00	1.00	2.00	1.00	2.00	1.00	1.00	1.00
447	1.00	1.00	1.00	1.00	1.00	1.00	2.00	2.00
448	1.00	2.00	2.00	1.00	2.00	1.00	1.00	1.00
449	2.00	1.00	2.00	2.00	1.00	1.00	2.00	2.00
450	1.00	1.00	2.00	1.00	2.00	1.00	2.00	2.00
451	1.00	1.00	1.00	1.00	2.00	1.00	2.00	1.00
452	2.00	1.00	1.00	1.00	1.00	1.00	1.00	2.00
453	2.00	1.00	2.00	1.00	1.00	1.00	2.00	2.00
454	1.00	1.00	2.00	1.00	2.00	1.00	2.00	2.00
455	2.00	1.00	1.00	1.00	1.00	1.00	2.00	2.00
456	2.00	1.00	2.00	1.00	2.00	1.00	2.00	2.00
457	1.00	2.00	2.00	1.00	1.00	1.00	2.00	2.00
458	1.00	1.00	2.00	1.00	1.00	1.00	2.00	2.00
459	1.00	2.00	2.00	2.00	2.00	1.00	2.00	2.00
460	1.00	1.00	2.00	1.00	2.00	1.00	2.00	2.00
461	1.00	2.00	2.00	2.00	2.00	1.00	2.00	2.00
462	1.00	1.00	1.00	1.00	1.00	1.00	1.00	2.00

	V3	V4	V5	V6	E2	E3	E4	C1
421	2.00	1.00	1.00	2.00	1.00	2.00	1.00	2.00
422	2.00	2.00	2.00	2.00	1.00	2.00	1.00	2.00
423	2.00	2.00	2.00	2.00	1.00	2.00	1.00	2.00
424	2.00	2.00	2.00	1.00	1.00	2.00	2.00	2.00
425	2.00	2.00	2.00	2.00	1.00	2.00	2.00	2.00
426	2.00	2.00	2.00	2.00	1.00	2.00	2.00	2.00
427	2.00	2.00	1.00	1.00	1.00	2.00	1.00	2.00
428	2.00	1.00	1.00	2.00	1.00	2.00	2.00	2.00
429	1.00	2.00	2.00	2.00	1.00	2.00	2.00	2.00
430	2.00	2.00	1.00	1.00	1.00	2.00	2.00	2.00
431	1.00	1.00	2.00	1.00	1.00	2.00	1.00	2.00
432	2.00	2.00	2.00	2.00	1.00	2.00	1.00	2.00
433	2.00	2.00	1.00	1.00	1.00	2.00	2.00	2.00
434	1.00	1.00	1.00	1.00	1.00	2.00	1.00	2.00
435	2.00	2.00	2.00	1.00	1.00	2.00	2.00	2.00
436	2.00	2.00	1.00	1.00	1.00	2.00	1.00	2.00
437	2.00	1.00	1.00	2.00	1.00	2.00	1.00	2.00
438	2.00	1.00	1.00	1.00	1.00	2.00	1.00	2.00
439	1.00	2.00	2.00	1.00	1.00	2.00	2.00	2.00
440	2.00	1.00	2.00	1.00	1.00	2.00	1.00	2.00
441	2.00	2.00	1.00	2.00	1.00	2.00	1.00	2.00
442	1.00	1.00	1.00	1.00	1.00	2.00	1.00	2.00
443	1.00	2.00	2.00	2.00	1.00	2.00	2.00	2.00
444	2.00	2.00	2.00	2.00	1.00	2.00	1.00	2.00
445	2.00	1.00	1.00	2.00	1.00	2.00	1.00	2.00
446	1.00	2.00	1.00	2.00	1.00	2.00	2.00	2.00
447	1.00	2.00	1.00	1.00	1.00	2.00	2.00	2.00
448	2.00	1.00	1.00	2.00	1.00	2.00	1.00	2.00
449	1.00	2.00	2.00	2.00	1.00	2.00	1.00	2.00
450	2.00	1.00	2.00	2.00	1.00	2.00	2.00	2.00
451	1.00	1.00	2.00	2.00	1.00	2.00	2.00	2.00
452	1.00	1.00	2.00	2.00	1.00	2.00	2.00	2.00
453	2.00	2.00	1.00	1.00	1.00	2.00	1.00	2.00
454	1.00	1.00	1.00	1.00	1.00	1.00	1.00	2.00
455	1.00	1.00	1.00	2.00	1.00	2.00	1.00	2.00
456	1.00	1.00	1.00	2.00	1.00	2.00	2.00	2.00
457	2.00	2.00	2.00	2.00	1.00	2.00	2.00	2.00
458	2.00	1.00	2.00	1.00	1.00	2.00	2.00	2.00
459	2.00	2.00	2.00	1.00	1.00	2.00	2.00	2.00
460	2.00	2.00	2.00	1.00	1.00	2.00	2.00	2.00
461	2.00	1.00	2.00	2.00	1.00	2.00	1.00	2.00
462	2.00	2.00	1.00	1.00	1.00	2.00	1.00	2.00

	C2	C3	C4	C5	C6	C7	filter_$
421	2.00	2.00	2.00	1.00	1.00	1.00	1
422	2.00	1.00	2.00	2.00	2.00	1.00	1
423	2.00	1.00	1.00	2.00	2.00	1.00	1
424	2.00	1.00	2.00	1.00	2.00	1.00	1
425	2.00	2.00	1.00	1.00	1.00	1.00	1
426	2.00	1.00	2.00	2.00	2.00	2.00	1
427	2.00	2.00	1.00	2.00	2.00	2.00	1
428	2.00	1.00	1.00	2.00	2.00	2.00	1
429	2.00	1.00	2.00	2.00	2.00	2.00	1
430	2.00	1.00	2.00	2.00	2.00	1.00	1
431	2.00	1.00	1.00	1.00	2.00	2.00	1
432	2.00	1.00	1.00	2.00	2.00	2.00	1
433	2.00	1.00	2.00	2.00	2.00	1.00	1
434	2.00	2.00	2.00	2.00	2.00	2.00	1
435	2.00	1.00	2.00	2.00	2.00	2.00	1
436	2.00	1.00	1.00	2.00	2.00	2.00	1
437	2.00	1.00	1.00	2.00	1.00	2.00	1
438	2.00	1.00	1.00	1.00	2.00	2.00	1
439	2.00	1.00	1.00	2.00	2.00	2.00	1
440	2.00	1.00	2.00	2.00	2.00	2.00	1
441	2.00	1.00	1.00	2.00	2.00	2.00	1
442	2.00	1.00	1.00	1.00	2.00	2.00	1
443	2.00	1.00	1.00	2.00	2.00	2.00	1
444	2.00	1.00	2.00	1.00	2.00	2.00	1
445	2.00	1.00	1.00	2.00	2.00	2.00	1
446	2.00	2.00	1.00	1.00	1.00	1.00	1
447	2.00	1.00	1.00	1.00	1.00	2.00	1
448	2.00	1.00	2.00	2.00	1.00	2.00	1
449	2.00	1.00	2.00	1.00	2.00	2.00	1
450	2.00	1.00	2.00	2.00	2.00	2.00	1
451	2.00	1.00	2.00	2.00	2.00	2.00	1
452	2.00	1.00	2.00	1.00	1.00	2.00	1
453	2.00	1.00	1.00	1.00	2.00	1.00	1
454	2.00	1.00	1.00	1.00	2.00	2.00	1
455	2.00	1.00	1.00	2.00	2.00	2.00	1
456	2.00	1.00	2.00	1.00	2.00	1.00	1
457	2.00	2.00	1.00	1.00	2.00	2.00	1
458	2.00	1.00	1.00	1.00	1.00	1.00	1
459	2.00	1.00	2.00	2.00	2.00	2.00	1
460	2.00	1.00	2.00	2.00	2.00	2.00	1
461	2.00	1.00	2.00	2.00	2.00	2.00	1
462	2.00	1.00	2.00	2.00	2.00	1.00	1

	SN	Group	Gender	Age	Work	Work2	Work3	Sector
463	614.00	2.00	1.00	26.00	7.00	2.00	2.00	2.00
464	615.00	2.00	1.00	27.00	7.00	2.00	2.00	2.00
465	616.00	2.00	2.00	24.00	7.00	2.00	2.00	1.00
466	617.00	2.00	2.00	27.00	8.00	2.00	2.00	1.00
467	620.00	2.00	2.00	21.00	4.00	1.00	1.00	1.00
468	622.00	2.00	1.00	30.00	7.00	2.00	2.00	1.00
469	623.00	2.00	2.00	20.00	3.00	1.00	1.00	2.00
470	626.00	2.00	1.00	25.00	7.00	2.00	2.00	1.00
471	627.00	2.00	1.00	28.00	8.00	2.00	2.00	1.00
472	628.00	2.00	1.00	21.00	4.00	1.00	1.00	1.00
473	630.00	2.00	1.00	21.00	4.00	1.00	1.00	1.00
474	631.00	2.00	1.00	27.00	8.00	2.00	2.00	1.00
475	632.00	2.00	2.00	28.00	8.00	2.00	2.00	2.00
476	633.00	2.00	2.00	26.00	8.00	2.00	2.00	2.00
477	634.00	2.00	2.00	27.00	8.00	2.00	2.00	2.00
478	635.00	2.00	2.00	26.00	7.00	2.00	2.00	2.00
479	636.00	2.00	2.00	24.00	7.00	2.00	2.00	1.00
480	637.00	2.00	2.00	26.00	8.00	2.00	2.00	1.00
481	644.00	2.00	2.00	26.00	8.00	2.00	2.00	2.00
482	646.00	2.00	1.00	22.00	4.00	1.00	1.00	1.00
483	647.00	2.00	1.00	22.00	4.00	1.00	1.00	1.00
484	648.00	2.00	1.00	22.00	4.00	1.00	1.00	1.00
485	649.00	2.00	1.00	22.00	4.00	1.00	1.00	1.00
486	650.00	2.00	1.00	26.00	8.00	2.00	2.00	2.00
487	651.00	2.00	1.00	21.00	4.00	1.00	1.00	1.00
488	652.00	2.00	2.00	26.00	7.00	2.00	2.00	2.00
489	653.00	2.00	1.00	23.00	5.00	1.00	1.00	1.00
490	654.00	2.00	2.00	19.00	2.00	1.00	1.00	1.00
491	655.00	2.00	1.00	23.00	6.00	1.00	1.00	1.00
492	8.00	1.00	1.00	24.00	6.00	1.00	1.00	1.00
493	9.00	1.00	1.00	19.00	2.00	1.00	1.00	1.00
494	15.00	1.00	1.00	35.00	10.00	3.00	2.00	1.00
495	19.00	1.00	1.00	21.00	4.00	1.00	1.00	1.00
496	23.00	1.00	1.00	21.00	4.00	1.00	1.00	1.00
497	24.00	1.00	2.00	20.00	2.00	1.00	1.00	1.00
498	30.00	1.00	2.00	23.00	6.00	1.00	1.00	1.00
499	35.00	1.00	1.00	23.00	6.00	1.00	1.00	1.00
500	36.00	1.00	2.00	23.00	5.00	1.00	1.00	1.00
501	44.00	1.00	1.00	23.00	5.00	1.00	1.00	1.00
502	45.00	1.00	1.00	35.00	10.00	3.00	2.00	1.00
503	50.00	1.00	1.00	36.00	10.00	3.00	2.00	1.00
504	58.00	1.00	1.00	20.00	2.00	1.00	1.00	1.00

	Region	Nationality	M1	M2	M3	M4	M5	M6
463	1.00	1.00	1.00	1.00	1.00	1.00	1.00	1.00
464	1.00	1.00	1.00	1.00	2.00	1.00	1.00	1.00
465	3.00	1.00	1.00	1.00	2.00	1.00	1.00	1.00
466	1.00	1.00	1.00	2.00	1.00	1.00	2.00	2.00
467	1.00	1.00	1.00	1.00	2.00	1.00	2.00	1.00
468	1.00	1.00	1.00	2.00	1.00	1.00	1.00	1.00
469	1.00	1.00	1.00	1.00	2.00	1.00	1.00	1.00
470	2.00	1.00	1.00	1.00	2.00	1.00	2.00	1.00
471	5.00	1.00	1.00	2.00	2.00	2.00	2.00	2.00
472	5.00	1.00	1.00	1.00	2.00	1.00	1.00	1.00
473	5.00	1.00	1.00	1.00	1.00	1.00	1.00	1.00
474	2.00	1.00	1.00	1.00	2.00	1.00	1.00	1.00
475	4.00	1.00	1.00	1.00	1.00	1.00	1.00	1.00
476	4.00	1.00	1.00	2.00	1.00	1.00	1.00	1.00
477	2.00	1.00	1.00	1.00	1.00	1.00	1.00	1.00
478	1.00	1.00	1.00	1.00	1.00	1.00	2.00	1.00
479	2.00	1.00	1.00	1.00	1.00	1.00	1.00	1.00
480	3.00	1.00	1.00	1.00	1.00	1.00	2.00	2.00
481	5.00	1.00	1.00	1.00	2.00	1.00	1.00	2.00
482	2.00	1.00	2.00	1.00	2.00	1.00	1.00	1.00
483	2.00	1.00	1.00	1.00	2.00	1.00	1.00	1.00
484	2.00	1.00	2.00	2.00	1.00	1.00	1.00	1.00
485	2.00	1.00	1.00	2.00	1.00	1.00	2.00	1.00
486	1.00	1.00	2.00	2.00	2.00	2.00	1.00	1.00
487	3.00	1.00	1.00	1.00	1.00	1.00	1.00	1.00
488	1.00	1.00	1.00	1.00	1.00	2.00	1.00	1.00
489	4.00	1.00	1.00	1.00	2.00	1.00	2.00	1.00
490	4.00	1.00	1.00	1.00	2.00	1.00	1.00	1.00
491	1.00	1.00	1.00	1.00	2.00	1.00	1.00	1.00
492	1.00	1.00	1.00	1.00	1.00	1.00	1.00	2.00
493	1.00	1.00	1.00	1.00	1.00	1.00	1.00	1.00
494	1.00	1.00	2.00	1.00	1.00	2.00	1.00	1.00
495	1.00	1.00	1.00	1.00	1.00	1.00	2.00	1.00
496	2.00	1.00	1.00	1.00	2.00	1.00	1.00	1.00
497	2.00	1.00	1.00	1.00	1.00	1.00	1.00	2.00
498	1.00	1.00	1.00	1.00	2.00	1.00	1.00	1.00
499	1.00	1.00	2.00	1.00	1.00	2.00	1.00	2.00
500	1.00	1.00	2.00	1.00	1.00	1.00	1.00	2.00
501	1.00	1.00	1.00	1.00	2.00	1.00	1.00	1.00
502	1.00	1.00	1.00	1.00	1.00	1.00	1.00	1.00
503	1.00	1.00	1.00	1.00	2.00	1.00	1.00	2.00
504	2.00	1.00	1.00	1.00	2.00	1.00	1.00	1.00

	M7	M8	M9	M10	M11	M12	M13	M14
463	2.00	2.00	2.00	1.00	2.00	1.00	2.00	1.00
464	1.00	1.00	1.00	2.00	1.00	1.00	2.00	1.00
465	2.00	1.00	1.00	1.00	1.00	2.00	1.00	1.00
466	2.00	2.00	1.00	2.00	2.00	1.00	2.00	1.00
467	2.00	1.00	2.00	1.00	1.00	1.00	2.00	1.00
468	1.00	2.00	1.00	1.00	1.00	1.00	1.00	2.00
469	1.00	1.00	1.00	1.00	1.00	1.00	1.00	1.00
470	2.00	1.00	1.00	1.00	2.00	1.00	2.00	1.00
471	2.00	2.00	1.00	1.00	2.00	1.00	1.00	2.00
472	1.00	2.00	1.00	1.00	1.00	2.00	1.00	1.00
473	1.00	1.00	1.00	1.00	2.00	1.00	1.00	1.00
474	1.00	1.00	1.00	1.00	1.00	2.00	2.00	1.00
475	1.00	1.00	1.00	1.00	2.00	1.00	1.00	1.00
476	2.00	2.00	1.00	1.00	2.00	1.00	1.00	1.00
477	1.00	1.00	1.00	1.00	1.00	1.00	1.00	1.00
478	1.00	1.00	1.00	1.00	2.00	2.00	2.00	2.00
479	1.00	1.00	1.00	1.00	1.00	1.00	1.00	1.00
480	2.00	1.00	1.00	2.00	1.00	1.00	2.00	1.00
481	1.00	1.00	1.00	1.00	2.00	1.00	2.00	2.00
482	2.00	1.00	1.00	1.00	1.00	1.00	2.00	1.00
483	1.00	1.00	1.00	1.00	2.00	1.00	2.00	1.00
484	1.00	2.00	2.00	1.00	1.00	2.00	2.00	1.00
485	1.00	1.00	2.00	1.00	2.00	1.00	1.00	2.00
486	2.00	1.00	2.00	1.00	2.00	2.00	1.00	2.00
487	1.00	1.00	1.00	1.00	1.00	1.00	1.00	1.00
488	1.00	1.00	1.00	1.00	2.00	1.00	2.00	1.00
489	2.00	1.00	1.00	1.00	2.00	1.00	2.00	2.00
490	1.00	2.00	1.00	2.00	2.00	1.00	1.00	1.00
491	1.00	1.00	1.00	1.00	1.00	1.00	1.00	1.00
492	1.00	2.00	2.00	1.00	1.00	2.00	2.00	1.00
493	1.00	1.00	1.00	1.00	2.00	1.00	1.00	1.00
494	1.00	1.00	1.00	1.00	2.00	2.00	2.00	2.00
495	1.00	1.00	1.00	1.00	2.00	1.00	1.00	1.00
496	1.00	1.00	1.00	1.00	1.00	1.00	1.00	1.00
497	1.00	1.00	1.00	1.00	1.00	1.00	1.00	1.00
498	1.00	1.00	2.00	2.00	1.00	1.00	1.00	1.00
499	2.00	1.00	2.00	1.00	1.00	1.00	1.00	1.00
500	1.00	1.00	1.00	1.00	2.00	1.00	1.00	1.00
501	1.00	1.00	1.00	1.00	1.00	1.00	1.00	1.00
502	1.00	1.00	2.00	1.00	1.00	2.00	2.00	2.00
503	1.00	1.00	1.00	1.00	1.00	1.00	1.00	2.00
504	1.00	1.00	1.00	1.00	2.00	1.00	2.00	1.00

	M15	B1	B2	B3	B4	B5	B6	B7
463	1.00	1.00	1.00	1.00	1.00	2.00	1.00	1.00
464	1.00	1.00	1.00	2.00	1.00	2.00	2.00	2.00
465	1.00	1.00	1.00	1.00	1.00	1.00	1.00	1.00
466	1.00	2.00	1.00	1.00	1.00	2.00	2.00	2.00
467	2.00	2.00	2.00	2.00	2.00	2.00	2.00	2.00
468	1.00	1.00	1.00	2.00	1.00	1.00	2.00	1.00
469	1.00	1.00	2.00	1.00	1.00	1.00	2.00	2.00
470	1.00	2.00	1.00	2.00	1.00	2.00	2.00	2.00
471	2.00	2.00	2.00	1.00	1.00	1.00	2.00	2.00
472	1.00	1.00	1.00	2.00	1.00	1.00	2.00	1.00
473	1.00	1.00	1.00	1.00	1.00	1.00	1.00	1.00
474	1.00	2.00	1.00	1.00	1.00	1.00	2.00	2.00
475	1.00	2.00	1.00	1.00	2.00	2.00	2.00	2.00
476	1.00	2.00	1.00	1.00	1.00	1.00	1.00	1.00
477	1.00	1.00	1.00	1.00	1.00	1.00	1.00	1.00
478	1.00	2.00	1.00	2.00	2.00	2.00	2.00	2.00
479	1.00	1.00	1.00	1.00	1.00	1.00	1.00	1.00
480	1.00	1.00	2.00	2.00	1.00	2.00	1.00	1.00
481	1.00	1.00	2.00	2.00	1.00	2.00	2.00	1.00
482	1.00	1.00	1.00	2.00	1.00	1.00	2.00	2.00
483	1.00	2.00	1.00	2.00	1.00	2.00	2.00	2.00
484	1.00	1.00	1.00	1.00	2.00	2.00	1.00	1.00
485	1.00	2.00	1.00	1.00	1.00	2.00	1.00	1.00
486	2.00	1.00	2.00	2.00	1.00	1.00	1.00	1.00
487	1.00	1.00	1.00	1.00	1.00	1.00	1.00	1.00
488	1.00	2.00	1.00	2.00	1.00	2.00	2.00	2.00
489	1.00	2.00	2.00	2.00	2.00	1.00	2.00	2.00
490	1.00	2.00	2.00	1.00	2.00	1.00	1.00	1.00
491	1.00	2.00	1.00	1.00	1.00	2.00	2.00	2.00
492	2.00	1.00	1.00	1.00	1.00	1.00	1.00	1.00
493	1.00	1.00	1.00	2.00	1.00	1.00	1.00	1.00
494	2.00	1.00	2.00	2.00	1.00	2.00	2.00	2.00
495	1.00	2.00	2.00	2.00	2.00	2.00	2.00	2.00
496	2.00	2.00	2.00	2.00	1.00	2.00	2.00	2.00
497	1.00	1.00	2.00	2.00	1.00	2.00	1.00	1.00
498	1.00	1.00	1.00	2.00	1.00	1.00	1.00	1.00
499	2.00	1.00	1.00	2.00	1.00	1.00	1.00	1.00
500	1.00	2.00	2.00	1.00	1.00	1.00	2.00	2.00
501	2.00	2.00	2.00	2.00	1.00	1.00	1.00	1.00
502	2.00	1.00	1.00	2.00	1.00	2.00	2.00	2.00
503	1.00	1.00	1.00	1.00	1.00	2.00	2.00	2.00
504	1.00	2.00	2.00	2.00	1.00	1.00	2.00	2.00

	B8	B9	B10	B11	B12	B13	B14	W1
463	1.00	1.00	1.00	1.00	1.00	1.00	1.00	2.00
464	2.00	2.00	1.00	1.00	2.00	2.00	1.00	2.00
465	2.00	1.00	1.00	1.00	2.00	1.00	1.00	1.00
466	2.00	1.00	2.00	2.00	1.00	2.00	2.00	2.00
467	2.00	2.00	2.00	1.00	2.00	2.00	1.00	2.00
468	2.00	1.00	1.00	1.00	1.00	2.00	1.00	1.00
469	2.00	2.00	1.00	1.00	2.00	2.00	1.00	1.00
470	2.00	2.00	2.00	1.00	1.00	2.00	1.00	1.00
471	2.00	2.00	2.00	2.00	2.00	2.00	2.00	1.00
472	1.00	1.00	2.00	1.00	1.00	1.00	1.00	1.00
473	1.00	1.00	1.00	2.00	1.00	2.00	1.00	1.00
474	1.00	1.00	1.00	1.00	2.00	1.00	1.00	1.00
475	1.00	2.00	1.00	2.00	2.00	2.00	1.00	2.00
476	1.00	1.00	2.00	2.00	2.00	2.00	1.00	2.00
477	1.00	1.00	1.00	1.00	1.00	2.00	2.00	1.00
478	2.00	2.00	2.00	1.00	2.00	2.00	2.00	1.00
479	1.00	1.00	1.00	1.00	1.00	1.00	2.00	1.00
480	2.00	2.00	1.00	1.00	2.00	2.00	1.00	2.00
481	1.00	2.00	1.00	1.00	2.00	1.00	1.00	1.00
482	2.00	2.00	1.00	1.00	2.00	2.00	2.00	2.00
483	2.00	2.00	1.00	1.00	2.00	2.00	1.00	1.00
484	2.00	2.00	2.00	2.00	2.00	2.00	2.00	2.00
485	2.00	1.00	2.00	1.00	1.00	1.00	1.00	1.00
486	1.00	1.00	1.00	1.00	1.00	1.00	1.00	1.00
487	1.00	1.00	1.00	1.00	1.00	2.00	2.00	1.00
488	2.00	1.00	2.00	1.00	2.00	2.00	2.00	2.00
489	2.00	2.00	2.00	1.00	2.00	2.00	2.00	1.00
490	2.00	2.00	1.00	2.00	1.00	1.00	1.00	1.00
491	2.00	2.00	2.00	1.00	2.00	2.00	1.00	1.00
492	1.00	1.00	1.00	1.00	1.00	1.00	1.00	1.00
493	1.00	1.00	1.00	1.00	1.00	1.00	1.00	1.00
494	1.00	2.00	2.00	2.00	2.00	2.00	1.00	2.00
495	2.00	2.00	2.00	2.00	2.00	2.00	2.00	1.00
496	2.00	2.00	1.00	2.00	2.00	2.00	2.00	2.00
497	2.00	1.00	2.00	2.00	2.00	1.00	1.00	2.00
498	2.00	1.00	2.00	1.00	2.00	1.00	2.00	1.00
499	1.00	1.00	1.00	1.00	2.00	2.00	2.00	1.00
500	2.00	2.00	1.00	1.00	2.00	2.00	2.00	1.00
501	2.00	2.00	1.00	1.00	2.00	1.00	2.00	1.00
502	2.00	2.00	1.00	1.00	2.00	2.00	2.00	2.00
503	1.00	2.00	1.00	1.00	2.00	2.00	2.00	2.00
504	2.00	2.00	2.00	1.00	2.00	1.00	2.00	1.00

	W2	W3	W4	W5	W6	E1	V1	V2
463	2.00	1.00	1.00	2.00	2.00	1.00	1.00	1.00
464	2.00	2.00	2.00	1.00	2.00	1.00	2.00	2.00
465	2.00	1.00	1.00	1.00	2.00	1.00	2.00	2.00
466	1.00	1.00	2.00	1.00	1.00	1.00	1.00	1.00
467	2.00	1.00	1.00	1.00	2.00	1.00	2.00	2.00
468	1.00	1.00	2.00	1.00	1.00	1.00	1.00	2.00
469	1.00	1.00	1.00	1.00	2.00	1.00	2.00	2.00
470	2.00	1.00	1.00	2.00	2.00	1.00	2.00	2.00
471	1.00	2.00	2.00	2.00	2.00	1.00	1.00	1.00
472	1.00	1.00	1.00	1.00	1.00	1.00	1.00	2.00
473	1.00	2.00	2.00	2.00	2.00	1.00	2.00	2.00
474	1.00	2.00	1.00	2.00	2.00	1.00	2.00	2.00
475	2.00	1.00	1.00	2.00	2.00	1.00	2.00	2.00
476	2.00	1.00	1.00	1.00	1.00	1.00	2.00	2.00
477	1.00	1.00	1.00	1.00	1.00	1.00	2.00	2.00
478	1.00	2.00	2.00	1.00	2.00	1.00	1.00	1.00
479	1.00	1.00	1.00	1.00	1.00	1.00	1.00	1.00
480	2.00	1.00	2.00	2.00	1.00	1.00	2.00	2.00
481	1.00	1.00	1.00	2.00	2.00	1.00	2.00	2.00
482	2.00	1.00	2.00	2.00	2.00	1.00	2.00	2.00
483	2.00	1.00	2.00	1.00	2.00	1.00	2.00	2.00
484	1.00	2.00	2.00	2.00	1.00	1.00	2.00	1.00
485	2.00	1.00	1.00	1.00	2.00	1.00	1.00	1.00
486	2.00	1.00	1.00	1.00	1.00	1.00	1.00	2.00
487	1.00	1.00	2.00	2.00	2.00	1.00	1.00	1.00
488	2.00	1.00	2.00	1.00	2.00	1.00	2.00	2.00
489	2.00	1.00	1.00	1.00	2.00	1.00	1.00	1.00
490	1.00	1.00	1.00	2.00	2.00	1.00	1.00	1.00
491	1.00	1.00	1.00	1.00	1.00	1.00	2.00	2.00
492	1.00	2.00	1.00	2.00	2.00	2.00	.	.
493	1.00	1.00	1.00	1.00	2.00	2.00	.	.
494	2.00	1.00	1.00	2.00	1.00	2.00	.	.
495	1.00	1.00	1.00	1.00	1.00	2.00	.	.
496	2.00	1.00	1.00	1.00	1.00	2.00	.	.
497	2.00	1.00	1.00	1.00	1.00	2.00	.	.
498	2.00	1.00	2.00	1.00	1.00	2.00	.	.
499	1.00	2.00	1.00	1.00	2.00	2.00	.	.
500	2.00	1.00	2.00	1.00	2.00	2.00	.	.
501	1.00	1.00	1.00	1.00	2.00	2.00	.	.
502	2.00	2.00	2.00	2.00	2.00	2.00	.	.
503	2.00	1.00	1.00	1.00	1.00	2.00	.	.
504	1.00	1.00	1.00	1.00	2.00	2.00	.	.

	V3	V4	V5	V6	E2	E3	E4	C1
463	1.00	1.00	1.00	1.00	1.00	2.00	2.00	2.00
464	2.00	2.00	1.00	2.00	1.00	2.00	1.00	2.00
465	1.00	2.00	2.00	2.00	1.00	2.00	2.00	2.00
466	2.00	1.00	2.00	2.00	1.00	2.00	2.00	1.00
467	2.00	2.00	1.00	2.00	1.00	2.00	2.00	1.00
468	1.00	1.00	1.00	1.00	1.00	2.00	1.00	1.00
469	2.00	2.00	2.00	1.00	1.00	2.00	2.00	2.00
470	1.00	1.00	2.00	1.00	1.00	2.00	1.00	2.00
471	2.00	2.00	2.00	2.00	1.00	1.00	1.00	2.00
472	1.00	1.00	1.00	1.00	1.00	2.00	2.00	2.00
473	2.00	2.00	2.00	1.00	1.00	2.00	1.00	2.00
474	2.00	2.00	2.00	2.00	1.00	2.00	1.00	2.00
475	2.00	2.00	2.00	1.00	1.00	2.00	2.00	2.00
476	1.00	1.00	1.00	1.00	1.00	2.00	1.00	2.00
477	2.00	2.00	1.00	1.00	1.00	2.00	1.00	2.00
478	2.00	2.00	1.00	2.00	1.00	2.00	1.00	2.00
479	1.00	1.00	1.00	1.00	1.00	2.00	1.00	1.00
480	1.00	2.00	2.00	1.00	1.00	2.00	1.00	2.00
481	1.00	2.00	1.00	2.00	1.00	2.00	2.00	2.00
482	2.00	2.00	2.00	2.00	1.00	2.00	1.00	2.00
483	2.00	2.00	2.00	1.00	1.00	2.00	2.00	2.00
484	2.00	2.00	2.00	2.00	1.00	2.00	2.00	2.00
485	2.00	1.00	2.00	1.00	1.00	2.00	2.00	2.00
486	1.00	1.00	1.00	1.00	1.00	2.00	1.00	2.00
487	1.00	1.00	1.00	1.00	1.00	1.00	1.00	1.00
488	2.00	2.00	2.00	2.00	1.00	2.00	1.00	2.00
489	1.00	2.00	2.00	2.00	1.00	2.00	1.00	2.00
490	2.00	2.00	1.00	1.00	1.00	2.00	2.00	2.00
491	2.00	1.00	1.00	1.00	1.00	2.00	2.00	2.00
492	.	.	.	.	.	.	.	.
493	.	.	.	.	.	.	.	.
494	.	.	.	.	.	.	.	.
495	.	.	.	.	.	.	.	.
496	.	.	.	.	.	.	.	.
497	.	.	.	.	.	.	.	.
498	.	.	.	.	.	.	.	.
499	.	.	.	.	.	.	.	.
500	.	.	.	.	.	.	.	.
501	.	.	.	.	.	.	.	.
502	.	.	.	.	.	.	.	.
503	.	.	.	.	.	.	.	.
504	.	.	.	.	.	.	.	.

	C2	C3	C4	C5	C6	C7	filter_$
463	2.00	1.00	1.00	1.00	1.00	1.00	1
464	2.00	1.00	1.00	2.00	2.00	2.00	1
465	2.00	1.00	2.00	2.00	2.00	2.00	1
466	1.00	2.00	2.00	2.00	1.00	1.00	1
467	2.00	1.00	2.00	1.00	1.00	1.00	1
468	2.00	1.00	1.00	1.00	1.00	1.00	1
469	2.00	1.00	2.00	2.00	2.00	1.00	1
470	2.00	1.00	1.00	1.00	2.00	2.00	1
471	2.00	1.00	1.00	2.00	2.00	2.00	1
472	2.00	2.00	2.00	2.00	2.00	2.00	1
473	2.00	1.00	1.00	1.00	2.00	2.00	1
474	2.00	1.00	1.00	1.00	2.00	2.00	1
475	1.00	1.00	1.00	1.00	2.00	2.00	1
476	2.00	1.00	2.00	1.00	2.00	1.00	1
477	2.00	1.00	1.00	1.00	1.00	1.00	1
478	2.00	1.00	2.00	1.00	2.00	2.00	1
479	1.00	1.00	1.00	1.00	1.00	1.00	1
480	2.00	1.00	1.00	2.00	2.00	2.00	1
481	2.00	1.00	1.00	2.00	2.00	1.00	1
482	2.00	1.00	1.00	1.00	2.00	2.00	1
483	2.00	1.00	2.00	2.00	2.00	2.00	1
484	2.00	2.00	2.00	2.00	2.00	2.00	1
485	2.00	1.00	1.00	1.00	1.00	1.00	1
486	2.00	1.00	2.00	1.00	2.00	1.00	1
487	1.00	1.00	1.00	1.00	1.00	1.00	1
488	2.00	1.00	2.00	2.00	2.00	2.00	1
489	2.00	1.00	2.00	1.00	2.00	2.00	1
490	2.00	1.00	1.00	2.00	2.00	2.00	1
491	2.00	1.00	1.00	1.00	1.00	2.00	1
492	.	.	.	.	.	.	0
493	.	.	.	.	.	.	0
494	.	.	.	.	.	.	0
495	.	.	.	.	.	.	0
496	.	.	.	.	.	.	0
497	.	.	.	.	.	.	0
498	.	.	.	.	.	.	0
499	.	.	.	.	.	.	0
500	.	.	.	.	.	.	0
501	.	.	.	.	.	.	0
502	.	.	.	.	.	.	0
503	.	.	.	.	.	.	0
504	.	.	.	.	.	.	0

	SN	Group	Gender	Age	Work	Work2	Work3	Sector
505	59.00	1.00	2.00	20.00	3.00	1.00	1.00	1.00
506	60.00	1.00	1.00	20.00	2.00	1.00	1.00	1.00
507	61.00	1.00	1.00	23.00	2.00	1.00	1.00	1.00
508	62.00	1.00	1.00	23.00	5.00	1.00	1.00	1.00
509	63.00	1.00	2.00	21.00	3.00	1.00	1.00	1.00
510	65.00	1.00	2.00	19.00	2.00	1.00	1.00	2.00
511	68.00	1.00	2.00	20.00	7.00	2.00	2.00	1.00
512	70.00	1.00	2.00	20.00	3.00	1.00	1.00	1.00
513	71.00	1.00	2.00	20.00	3.00	1.00	1.00	1.00
514	74.00	1.00	1.00	21.00	3.00	1.00	1.00	1.00
515	76.00	1.00	1.00	21.00	3.00	1.00	1.00	1.00
516	81.00	1.00	1.00	21.00	3.00	1.00	1.00	1.00
517	83.00	1.00	1.00	21.00	3.00	1.00	1.00	1.00
518	85.00	1.00	1.00	19.00	2.00	1.00	1.00	1.00
519	86.00	1.00	1.00	20.00	2.00	1.00	1.00	1.00
520	87.00	1.00	1.00	20.00	2.00	1.00	1.00	1.00
521	89.00	1.00	1.00	20.00	3.00	1.00	1.00	1.00
522	92.00	1.00	1.00	19.00	2.00	1.00	1.00	1.00
523	93.00	1.00	1.00	19.00	2.00	1.00	1.00	1.00
524	94.00	1.00	1.00	20.00	3.00	1.00	1.00	1.00
525	97.00	1.00	1.00	23.00	5.00	1.00	1.00	1.00
526	101.00	1.00	1.00	21.00	3.00	1.00	1.00	1.00
527	102.00	1.00	1.00	22.00	5.00	1.00	1.00	1.00
528	103.00	1.00	1.00	20.00	3.00	1.00	1.00	1.00
529	107.00	1.00	1.00	21.00	4.00	1.00	1.00	1.00
530	109.00	1.00	1.00	25.00	6.00	1.00	1.00	1.00
531	112.00	1.00	1.00	21.00	3.00	1.00	1.00	1.00
532	113.00	1.00	1.00	22.00	5.00	1.00	1.00	1.00
533	114.00	1.00	2.00	37.00	10.00	3.00	2.00	1.00
534	119.00	1.00	1.00	27.00	8.00	2.00	2.00	2.00
535	123.00	1.00	2.00	20.00	2.00	1.00	1.00	1.00
536	132.00	1.00	2.00	22.00	5.00	1.00	1.00	1.00
537	135.00	1.00	2.00	21.00	6.00	1.00	1.00	1.00
538	140.00	1.00	2.00	28.00	8.00	2.00	2.00	1.00
539	143.00	1.00	1.00	20.00	2.00	1.00	1.00	1.00
540	145.00	1.00	1.00	22.00	3.00	1.00	1.00	1.00
541	152.00	1.00	1.00	24.00	6.00	1.00	1.00	1.00
542	156.00	1.00	1.00	23.00	5.00	1.00	1.00	2.00
543	162.00	1.00	1.00	23.00	6.00	1.00	1.00	1.00
544	178.00	1.00	1.00	21.00	4.00	1.00	1.00	1.00
545	194.00	1.00	1.00	21.00	3.00	1.00	1.00	1.00
546	199.00	1.00	2.00	19.00	2.00	1.00	1.00	1.00

	Region	Nationality	M1	M2	M3	M4	M5	M6
505	1.00	1.00	1.00	1.00	2.00	1.00	1.00	1.00
506	2.00	1.00	1.00	1.00	1.00	1.00	1.00	1.00
507	3.00	1.00	2.00	1.00	1.00	2.00	1.00	2.00
508	1.00	1.00	1.00	1.00	2.00	1.00	1.00	1.00
509	1.00	1.00	1.00	1.00	1.00	1.00	1.00	1.00
510	1.00	2.00	1.00	1.00	2.00	1.00	1.00	1.00
511	4.00	1.00	1.00	2.00	1.00	2.00	1.00	1.00
512	1.00	1.00	1.00	1.00	2.00	1.00	1.00	1.00
513	1.00	1.00	1.00	1.00	2.00	1.00	1.00	1.00
514	2.00	1.00	2.00	1.00	1.00	2.00	1.00	1.00
515	1.00	1.00	1.00	1.00	1.00	1.00	1.00	2.00
516	1.00	1.00	1.00	1.00	1.00	1.00	1.00	1.00
517	1.00	1.00	1.00	1.00	1.00	1.00	1.00	1.00
518	4.00	1.00	1.00	1.00	1.00	1.00	1.00	1.00
519	1.00	1.00	1.00	1.00	1.00	1.00	1.00	1.00
520	2.00	1.00	1.00	1.00	1.00	2.00	1.00	1.00
521	4.00	1.00	1.00	1.00	2.00	1.00	1.00	1.00
522	1.00	1.00	1.00	1.00	1.00	1.00	1.00	1.00
523	1.00	1.00	1.00	2.00	1.00	1.00	1.00	1.00
524	1.00	1.00	2.00	1.00	2.00	1.00	1.00	1.00
525	1.00	1.00	2.00	1.00	2.00	1.00	1.00	1.00
526	1.00	1.00	1.00	1.00	1.00	1.00	2.00	1.00
527	1.00	1.00	1.00	1.00	2.00	1.00	1.00	1.00
528	1.00	1.00	1.00	1.00	2.00	1.00	1.00	1.00
529	1.00	1.00	1.00	1.00	1.00	2.00	2.00	2.00
530	1.00	1.00	2.00	1.00	2.00	2.00	1.00	2.00
531	4.00	1.00	1.00	2.00	1.00	1.00	1.00	2.00
532	2.00	1.00	1.00	2.00	2.00	1.00	1.00	1.00
533	2.00	1.00	1.00	1.00	2.00	1.00	1.00	2.00
534	5.00	1.00	1.00	2.00	1.00	1.00	1.00	2.00
535	4.00	1.00	1.00	1.00	2.00	1.00	2.00	2.00
536	3.00	1.00	1.00	1.00	2.00	1.00	2.00	2.00
537	4.00	1.00	1.00	2.00	1.00	1.00	2.00	1.00
538	1.00	1.00	1.00	2.00	1.00	2.00	1.00	2.00
539	3.00	1.00	1.00	1.00	1.00	1.00	1.00	1.00
540	3.00	1.00	1.00	1.00	2.00	1.00	1.00	1.00
541	4.00	1.00	1.00	1.00	1.00	1.00	1.00	1.00
542	2.00	2.00	1.00	1.00	2.00	1.00	1.00	1.00
543	3.00	1.00	2.00	1.00	1.00	2.00	1.00	2.00
544	4.00	1.00	1.00	1.00	2.00	1.00	1.00	2.00
545	1.00	1.00	1.00	1.00	2.00	1.00	2.00	1.00
546	3.00	1.00	1.00	1.00	2.00	1.00	1.00	2.00

	M7	M8	M9	M10	M11	M12	M13	M14
505	1.00	1.00	1.00	1.00	1.00	1.00	1.00	1.00
506	1.00	1.00	1.00	1.00	2.00	1.00	1.00	1.00
507	2.00	1.00	2.00	2.00	1.00	2.00	1.00	2.00
508	2.00	1.00	1.00	1.00	1.00	1.00	1.00	1.00
509	1.00	1.00	1.00	1.00	1.00	1.00	1.00	1.00
510	1.00	1.00	2.00	1.00	1.00	1.00	1.00	1.00
511	1.00	1.00	2.00	1.00	2.00	1.00	1.00	1.00
512	2.00	1.00	2.00	1.00	1.00	2.00	1.00	1.00
513	1.00	1.00	1.00	1.00	2.00	1.00	1.00	1.00
514	2.00	1.00	2.00	1.00	2.00	1.00	1.00	1.00
515	1.00	1.00	1.00	1.00	1.00	2.00	1.00	1.00
516	1.00	1.00	1.00	1.00	1.00	2.00	1.00	1.00
517	2.00	2.00	1.00	1.00	1.00	2.00	1.00	1.00
518	1.00	2.00	1.00	1.00	1.00	2.00	1.00	1.00
519	1.00	1.00	2.00	1.00	1.00	1.00	1.00	1.00
520	2.00	1.00	1.00	1.00	1.00	2.00	2.00	1.00
521	1.00	1.00	2.00	1.00	1.00	1.00	1.00	1.00
522	1.00	1.00	1.00	1.00	1.00	1.00	1.00	1.00
523	1.00	1.00	2.00	1.00	1.00	2.00	1.00	1.00
524	2.00	1.00	1.00	2.00	2.00	1.00	1.00	1.00
525	2.00	1.00	2.00	2.00	1.00	1.00	1.00	1.00
526	1.00	1.00	1.00	1.00	1.00	2.00	1.00	1.00
527	1.00	1.00	1.00	1.00	2.00	1.00	2.00	1.00
528	1.00	1.00	2.00	1.00	1.00	1.00	1.00	1.00
529	1.00	1.00	2.00	2.00	2.00	1.00	1.00	1.00
530	1.00	2.00	1.00	1.00	1.00	1.00	1.00	2.00
531	2.00	1.00	2.00	2.00	1.00	1.00	2.00	1.00
532	1.00	1.00	1.00	1.00	2.00	1.00	1.00	1.00
533	1.00	2.00	2.00	1.00	2.00	2.00	2.00	2.00
534	2.00	2.00	2.00	2.00	2.00	2.00	2.00	2.00
535	2.00	1.00	1.00	2.00	2.00	1.00	2.00	1.00
536	2.00	1.00	1.00	1.00	1.00	2.00	2.00	1.00
537	2.00	2.00	1.00	1.00	1.00	1.00	2.00	1.00
538	1.00	1.00	1.00	1.00	1.00	1.00	2.00	1.00
539	1.00	1.00	1.00	1.00	2.00	1.00	1.00	1.00
540	2.00	1.00	1.00	1.00	2.00	1.00	2.00	1.00
541	2.00	1.00	1.00	1.00	2.00	2.00	2.00	2.00
542	2.00	1.00	1.00	2.00	2.00	2.00	2.00	2.00
543	1.00	2.00	1.00	2.00	1.00	2.00	1.00	2.00
544	1.00	1.00	2.00	1.00	1.00	1.00	1.00	1.00
545	2.00	1.00	1.00	1.00	1.00	2.00	1.00	1.00
546	2.00	1.00	1.00	1.00	1.00	1.00	1.00	1.00

	M15	B1	B2	B3	B4	B5	B6	B7
505	1.00	1.00	2.00	2.00	2.00	2.00	1.00	1.00
506	1.00	1.00	2.00	2.00	1.00	2.00	2.00	2.00
507	1.00	2.00	1.00	1.00	2.00	1.00	2.00	1.00
508	1.00	1.00	2.00	1.00	1.00	1.00	1.00	1.00
509	1.00	2.00	2.00	2.00	2.00	2.00	2.00	2.00
510	1.00	1.00	2.00	2.00	1.00	1.00	1.00	1.00
511	2.00	1.00	1.00	1.00	2.00	2.00	2.00	2.00
512	1.00	1.00	2.00	1.00	1.00	1.00	1.00	1.00
513	1.00	1.00	1.00	2.00	2.00	2.00	2.00	2.00
514	2.00	1.00	2.00	1.00	2.00	2.00	1.00	1.00
515	1.00	1.00	2.00	1.00	1.00	1.00	1.00	1.00
516	1.00	1.00	2.00	2.00	1.00	2.00	2.00	2.00
517	1.00	2.00	1.00	2.00	1.00	1.00	1.00	1.00
518	2.00	1.00	1.00	2.00	1.00	2.00	2.00	2.00
519	1.00	1.00	2.00	2.00	1.00	1.00	2.00	2.00
520	1.00	1.00	2.00	2.00	1.00	2.00	1.00	2.00
521	1.00	1.00	2.00	2.00	1.00	1.00	1.00	1.00
522	1.00	1.00	2.00	2.00	1.00	2.00	2.00	2.00
523	1.00	1.00	1.00	2.00	1.00	1.00	2.00	2.00
524	1.00	1.00	2.00	2.00	1.00	2.00	2.00	2.00
525	1.00	1.00	1.00	2.00	1.00	1.00	1.00	1.00
526	2.00	1.00	1.00	1.00	1.00	2.00	2.00	2.00
527	1.00	2.00	2.00	1.00	1.00	2.00	1.00	1.00
528	1.00	1.00	2.00	2.00	1.00	2.00	2.00	1.00
529	1.00	1.00	1.00	2.00	1.00	2.00	2.00	1.00
530	1.00	1.00	2.00	1.00	1.00	1.00	2.00	1.00
531	1.00	1.00	1.00	2.00	1.00	1.00	2.00	1.00
532	1.00	2.00	1.00	2.00	1.00	2.00	2.00	2.00
533	2.00	2.00	2.00	2.00	1.00	2.00	2.00	2.00
534	2.00	2.00	1.00	1.00	2.00	2.00	2.00	2.00
535	2.00	2.00	1.00	2.00	2.00	1.00	1.00	1.00
536	1.00	1.00	1.00	2.00	1.00	2.00	2.00	2.00
537	2.00	1.00	2.00	1.00	2.00	1.00	2.00	1.00
538	1.00	1.00	1.00	2.00	1.00	1.00	2.00	1.00
539	1.00	1.00	2.00	2.00	1.00	2.00	1.00	1.00
540	1.00	2.00	2.00	2.00	1.00	2.00	2.00	2.00
541	1.00	1.00	2.00	1.00	1.00	1.00	2.00	2.00
542	1.00	1.00	1.00	1.00	1.00	2.00	2.00	2.00
543	1.00	1.00	2.00	1.00	2.00	1.00	2.00	1.00
544	1.00	1.00	2.00	2.00	2.00	2.00	1.00	1.00
545	1.00	2.00	1.00	1.00	1.00	1.00	2.00	2.00
546	1.00	1.00	2.00	2.00	1.00	1.00	1.00	2.00

	B8	B9	B10	B11	B12	B13	B14	W1
505	1.00	1.00	1.00	2.00	1.00	1.00	1.00	1.00
506	1.00	1.00	1.00	1.00	2.00	2.00	2.00	1.00
507	2.00	1.00	2.00	1.00	2.00	1.00	2.00	1.00
508	1.00	1.00	2.00	1.00	2.00	2.00	2.00	2.00
509	2.00	2.00	2.00	2.00	2.00	2.00	2.00	1.00
510	1.00	2.00	2.00	2.00	1.00	1.00	1.00	2.00
511	1.00	1.00	1.00	2.00	1.00	1.00	2.00	1.00
512	1.00	1.00	1.00	1.00	1.00	1.00	1.00	1.00
513	2.00	2.00	2.00	1.00	2.00	2.00	2.00	1.00
514	2.00	2.00	1.00	2.00	1.00	2.00	1.00	1.00
515	2.00	1.00	1.00	2.00	1.00	1.00	1.00	1.00
516	1.00	1.00	2.00	2.00	2.00	2.00	2.00	1.00
517	2.00	1.00	2.00	1.00	1.00	2.00	1.00	1.00
518	2.00	2.00	2.00	1.00	2.00	2.00	2.00	2.00
519	2.00	1.00	1.00	1.00	2.00	2.00	2.00	1.00
520	2.00	1.00	1.00	2.00	2.00	1.00	2.00	2.00
521	1.00	1.00	1.00	1.00	2.00	2.00	1.00	1.00
522	2.00	2.00	2.00	1.00	2.00	2.00	2.00	1.00
523	2.00	2.00	1.00	1.00	1.00	2.00	1.00	1.00
524	2.00	2.00	2.00	1.00	2.00	2.00	2.00	1.00
525	2.00	2.00	2.00	1.00	2.00	2.00	1.00	2.00
526	1.00	1.00	1.00	1.00	2.00	2.00	2.00	1.00
527	2.00	2.00	1.00	1.00	2.00	2.00	1.00	2.00
528	2.00	2.00	2.00	1.00	2.00	2.00	2.00	2.00
529	2.00	1.00	2.00	1.00	2.00	2.00	2.00	2.00
530	1.00	1.00	1.00	1.00	1.00	1.00	1.00	1.00
531	2.00	2.00	2.00	2.00	1.00	2.00	1.00	2.00
532	2.00	2.00	2.00	2.00	2.00	2.00	2.00	1.00
533	2.00	1.00	2.00	1.00	2.00	2.00	2.00	2.00
534	2.00	2.00	2.00	2.00	2.00	2.00	2.00	2.00
535	2.00	2.00	2.00	1.00	1.00	2.00	1.00	2.00
536	1.00	1.00	1.00	2.00	2.00	2.00	2.00	2.00
537	2.00	1.00	2.00	2.00	1.00	1.00	2.00	1.00
538	1.00	1.00	1.00	2.00	2.00	1.00	1.00	2.00
539	1.00	2.00	1.00	1.00	2.00	2.00	2.00	2.00
540	2.00	2.00	1.00	1.00	2.00	2.00	2.00	1.00
541	2.00	2.00	2.00	2.00	2.00	2.00	1.00	2.00
542	2.00	2.00	2.00	2.00	2.00	2.00	2.00	1.00
543	2.00	1.00	2.00	1.00	2.00	1.00	2.00	2.00
544	1.00	1.00	1.00	1.00	2.00	2.00	2.00	1.00
545	2.00	2.00	1.00	1.00	2.00	2.00	1.00	1.00
546	2.00	1.00	1.00	1.00	1.00	1.00	1.00	2.00

	W2	W3	W4	W5	W6	E1	V1	V2
505	2.00	2.00	1.00	1.00	1.00	2.00	.	.
506	1.00	1.00	1.00	1.00	2.00	2.00	.	.
507	2.00	1.00	2.00	1.00	2.00	2.00	.	.
508	2.00	1.00	1.00	1.00	2.00	2.00	.	.
509	1.00	1.00	1.00	1.00	1.00	2.00	.	.
510	2.00	1.00	1.00	1.00	2.00	2.00	.	.
511	1.00	1.00	1.00	2.00	2.00	2.00	.	.
512	1.00	1.00	1.00	1.00	2.00	2.00	.	.
513	1.00	1.00	1.00	1.00	2.00	2.00	.	.
514	1.00	2.00	1.00	2.00	1.00	2.00	.	.
515	1.00	1.00	1.00	1.00	1.00	2.00	.	.
516	1.00	1.00	1.00	1.00	1.00	2.00	.	.
517	2.00	1.00	1.00	2.00	1.00	2.00	.	.
518	2.00	1.00	1.00	2.00	2.00	2.00	.	.
519	1.00	1.00	1.00	1.00	2.00	2.00	.	.
520	1.00	2.00	2.00	1.00	2.00	2.00	.	.
521	1.00	1.00	1.00	2.00	2.00	2.00	.	.
522	1.00	2.00	1.00	1.00	1.00	2.00	.	.
523	1.00	2.00	1.00	1.00	2.00	2.00	.	.
524	1.00	1.00	1.00	1.00	2.00	2.00	.	.
525	2.00	1.00	1.00	1.00	2.00	2.00	.	.
526	2.00	2.00	1.00	1.00	2.00	2.00	.	.
527	2.00	1.00	1.00	1.00	1.00	2.00	.	.
528	2.00	2.00	1.00	1.00	1.00	2.00	.	.
529	1.00	2.00	2.00	2.00	2.00	2.00	.	.
530	2.00	1.00	1.00	1.00	1.00	2.00	.	.
531	1.00	2.00	2.00	1.00	2.00	2.00	.	.
532	1.00	1.00	1.00	1.00	2.00	2.00	.	.
533	2.00	1.00	2.00	2.00	2.00	2.00	.	.
534	2.00	2.00	2.00	2.00	2.00	2.00	.	.
535	1.00	2.00	1.00	1.00	1.00	2.00	.	.
536	1.00	1.00	2.00	1.00	2.00	2.00	.	.
537	1.00	1.00	2.00	2.00	1.00	2.00	.	.
538	2.00	2.00	1.00	2.00	2.00	2.00	.	.
539	2.00	2.00	2.00	1.00	1.00	2.00	.	.
540	1.00	1.00	2.00	1.00	1.00	2.00	.	.
541	2.00	2.00	2.00	2.00	2.00	2.00	.	.
542	1.00	1.00	1.00	1.00	1.00	2.00	.	.
543	1.00	2.00	1.00	2.00	1.00	2.00	.	.
544	1.00	2.00	2.00	1.00	2.00	2.00	.	.
545	1.00	2.00	1.00	1.00	2.00	2.00	.	.
546	2.00	1.00	1.00	1.00	1.00	2.00	.	.

	V3	V4	V5	V6	E2	E3	E4	C1
505	.	.	.	.	.	.	.	.
506	.	.	.	.	.	.	.	.
507	.	.	.	.	.	.	.	.
508	.	.	.	.	.	.	.	.
509	.	.	.	.	.	.	.	.
510	.	.	.	.	.	.	.	.
511	.	.	.	.	.	.	.	.
512	.	.	.	.	.	.	.	.
513	.	.	.	.	.	.	.	.
514	.	.	.	.	.	.	.	.
515	.	.	.	.	.	.	.	.
516	.	.	.	.	.	.	.	.
517	.	.	.	.	.	.	.	.
518	.	.	.	.	.	.	.	.
519	.	.	.	.	.	.	.	.
520	.	.	.	.	.	.	.	.
521	.	.	.	.	.	.	.	.
522	.	.	.	.	.	.	.	.
523	.	.	.	.	.	.	.	.
524	.	.	.	.	.	.	.	.
525	.	.	.	.	.	.	.	.
526	.	.	.	.	.	.	.	.
527	.	.	.	.	.	.	.	.
528	.	.	.	.	.	.	.	.
529	.	.	.	.	.	.	.	.
530	.	.	.	.	.	.	.	.
531	.	.	.	.	.	.	.	.
532	.	.	.	.	.	.	.	.
533	.	.	.	.	.	.	.	.
534	.	.	.	.	.	.	.	.
535	.	.	.	.	.	.	.	.
536	.	.	.	.	.	.	.	.
537	.	.	.	.	.	.	.	.
538	.	.	.	.	.	.	.	.
539	.	.	.	.	.	.	.	.
540	.	.	.	.	.	.	.	.
541	.	.	.	.	.	.	.	.
542	.	.	.	.	.	.	.	.
543	.	.	.	.	.	.	.	.
544	.	.	.	.	.	.	.	.
545	.	.	.	.	.	.	.	.
546	.	.	.	.	.	.	.	.

	C2	C3	C4	C5	C6	C7	filter_$
505	.	.	.	.	.	.	0
506	.	.	.	.	.	.	0
507	.	.	.	.	.	.	0
508	.	.	.	.	.	.	0
509	.	.	.	.	.	.	0
510	.	.	.	.	.	.	0
511	.	.	.	.	.	.	0
512	.	.	.	.	.	.	0
513	.	.	.	.	.	.	0
514	.	.	.	.	.	.	0
515	.	.	.	.	.	.	0
516	.	.	.	.	.	.	0
517	.	.	.	.	.	.	0
518	.	.	.	.	.	.	0
519	.	.	.	.	.	.	0
520	.	.	.	.	.	.	0
521	.	.	.	.	.	.	0
522	.	.	.	.	.	.	0
523	.	.	.	.	.	.	0
524	.	.	.	.	.	.	0
525	.	.	.	.	.	.	0
526	.	.	.	.	.	.	0
527	.	.	.	.	.	.	0
528	.	.	.	.	.	.	0
529	.	.	.	.	.	.	0
530	.	.	.	.	.	.	0
531	.	.	.	.	.	.	0
532	.	.	.	.	.	.	0
533	.	.	.	.	.	.	0
534	.	.	.	.	.	.	0
535	.	.	.	.	.	.	0
536	.	.	.	.	.	.	0
537	.	.	.	.	.	.	0
538	.	.	.	.	.	.	0
539	.	.	.	.	.	.	0
540	.	.	.	.	.	.	0
541	.	.	.	.	.	.	0
542	.	.	.	.	.	.	0
543	.	.	.	.	.	.	0
544	.	.	.	.	.	.	0
545	.	.	.	.	.	.	0
546	.	.	.	.	.	.	0

	SN	Group	Gender	Age	Work	Work2	Work3	Sector
547	201.00	1.00	2.00	24.00	7.00	2.00	2.00	1.00
548	213.00	1.00	2.00	26.00	6.00	1.00	1.00	1.00
549	218.00	1.00	1.00	20.00	3.00	1.00	1.00	1.00
550	224.00	1.00	2.00	20.00	2.00	1.00	1.00	1.00
551	225.00	1.00	1.00	21.00	4.00	1.00	1.00	1.00
552	226.00	1.00	1.00	20.00	3.00	1.00	1.00	1.00
553	234.00	1.00	2.00	23.00	6.00	1.00	1.00	1.00
554	242.00	1.00	2.00	21.00	3.00	1.00	1.00	1.00
555	247.00	1.00	1.00	22.00	2.00	1.00	1.00	1.00
556	250.00	1.00	1.00	22.00	2.00	1.00	1.00	1.00
557	252.00	1.00	1.00	22.00	4.00	1.00	1.00	1.00
558	253.00	1.00	2.00	19.00	3.00	1.00	1.00	1.00
559	256.00	1.00	1.00	21.00	3.00	1.00	1.00	1.00
560	262.00	1.00	2.00	20.00	2.00	1.00	1.00	1.00
561	263.00	1.00	1.00	22.00	3.00	1.00	1.00	1.00
562	265.00	1.00	1.00	20.00	3.00	1.00	1.00	1.00
563	266.00	1.00	1.00	21.00	4.00	1.00	1.00	1.00
564	292.00	1.00	1.00	20.00	3.00	1.00	1.00	1.00
565	294.00	1.00	2.00	23.00	5.00	1.00	1.00	2.00
566	295.00	1.00	2.00	22.00	5.00	1.00	1.00	2.00
567	300.00	1.00	2.00	21.00	4.00	1.00	1.00	2.00
568	303.00	1.00	2.00	25.00	8.00	2.00	2.00	1.00
569	307.00	1.00	1.00	22.00	3.00	1.00	1.00	1.00
570	327.00	1.00	2.00	20.00	3.00	1.00	1.00	1.00
571	328.00	1.00	2.00	22.00	3.00	1.00	1.00	1.00
572	329.00	1.00	1.00	21.00	3.00	1.00	1.00	1.00
573	331.00	1.00	1.00	21.00	4.00	1.00	1.00	1.00
574	334.00	1.00	1.00	19.00	2.00	1.00	1.00	1.00
575	337.00	1.00	1.00	20.00	3.00	1.00	1.00	1.00
576	340.00	1.00	1.00	24.00	6.00	1.00	1.00	1.00
577	349.00	1.00	2.00	21.00	4.00	1.00	1.00	1.00
578	355.00	1.00	1.00	22.00	4.00	1.00	1.00	1.00
579	356.00	1.00	2.00	20.00	3.00	1.00	1.00	1.00
580	362.00	1.00	2.00	22.00	5.00	1.00	1.00	1.00
581	367.00	1.00	2.00	18.00	2.00	1.00	1.00	1.00
582	369.00	1.00	2.00	20.00	2.00	1.00	1.00	1.00
583	372.00	1.00	2.00	20.00	2.00	1.00	1.00	1.00
584	378.00	1.00	2.00	20.00	2.00	1.00	1.00	1.00
585	379.00	1.00	2.00	19.00	2.00	1.00	1.00	1.00
586	380.00	1.00	2.00	20.00	2.00	1.00	1.00	1.00
587	381.00	1.00	2.00	24.00	5.00	1.00	1.00	1.00
588	384.00	1.00	2.00	22.00	4.00	1.00	1.00	1.00

	Region	Nationality	M1	M2	M3	M4	M5	M6
547	1.00	1.00	1.00	1.00	1.00	1.00	2.00	2.00
548	1.00	1.00	1.00	1.00	2.00	1.00	2.00	1.00
549	2.00	1.00	1.00	2.00	2.00	1.00	2.00	1.00
550	2.00	1.00	1.00	1.00	1.00	1.00	1.00	2.00
551	2.00	1.00	1.00	2.00	2.00	1.00	1.00	1.00
552	2.00	1.00	2.00	2.00	2.00	2.00	2.00	1.00
553	4.00	1.00	1.00	1.00	2.00	1.00	1.00	1.00
554	2.00	1.00	1.00	1.00	2.00	1.00	1.00	1.00
555	2.00	1.00	1.00	1.00	2.00	1.00	1.00	1.00
556	2.00	1.00	2.00	1.00	1.00	1.00	1.00	2.00
557	2.00	1.00	1.00	1.00	2.00	2.00	1.00	1.00
558	1.00	1.00	1.00	2.00	2.00	1.00	1.00	1.00
559	1.00	1.00	1.00	1.00	1.00	1.00	1.00	1.00
560	5.00	1.00	1.00	1.00	1.00	1.00	1.00	1.00
561	1.00	1.00	1.00	1.00	1.00	1.00	1.00	1.00
562	2.00	1.00	1.00	1.00	1.00	1.00	1.00	1.00
563	1.00	1.00	1.00	2.00	1.00	1.00	1.00	1.00
564	2.00	1.00	2.00	2.00	2.00	2.00	2.00	2.00
565	3.00	1.00	1.00	1.00	1.00	1.00	1.00	2.00
566	1.00	2.00	2.00	1.00	2.00	2.00	1.00	2.00
567	1.00	1.00	1.00	2.00	2.00	1.00	1.00	1.00
568	1.00	1.00	2.00	2.00	2.00	2.00	2.00	2.00
569	3.00	1.00	2.00	2.00	2.00	2.00	2.00	2.00
570	5.00	1.00	1.00	1.00	1.00	1.00	1.00	1.00
571	5.00	1.00	1.00	1.00	1.00	1.00	1.00	1.00
572	5.00	1.00	1.00	1.00	2.00	1.00	2.00	2.00
573	5.00	1.00	2.00	2.00	2.00	1.00	2.00	1.00
574	5.00	1.00	1.00	1.00	1.00	1.00	1.00	1.00
575	3.00	1.00	1.00	1.00	2.00	1.00	1.00	1.00
576	3.00	1.00	2.00	2.00	2.00	2.00	2.00	2.00
577	2.00	1.00	1.00	1.00	1.00	1.00	1.00	2.00
578	5.00	1.00	1.00	1.00	2.00	1.00	1.00	1.00
579	4.00	1.00	1.00	1.00	2.00	1.00	2.00	1.00
580	5.00	1.00	2.00	1.00	2.00	1.00	1.00	1.00
581	5.00	1.00	1.00	1.00	1.00	2.00	2.00	2.00
582	5.00	1.00	1.00	1.00	2.00	1.00	1.00	1.00
583	5.00	1.00	1.00	1.00	1.00	1.00	1.00	1.00
584	1.00	1.00	1.00	1.00	2.00	1.00	1.00	1.00
585	1.00	1.00	1.00	1.00	2.00	1.00	1.00	1.00
586	1.00	1.00	1.00	2.00	1.00	2.00	1.00	1.00
587	2.00	1.00	1.00	1.00	1.00	1.00	1.00	1.00
588	2.00	1.00	2.00	1.00	2.00	2.00	2.00	2.00

	M7	M8	M9	M10	M11	M12	M13	M14
547	2.00	1.00	2.00	1.00	2.00	2.00	2.00	1.00
548	1.00	1.00	1.00	1.00	2.00	1.00	1.00	1.00
549	2.00	2.00	2.00	1.00	2.00	1.00	2.00	1.00
550	1.00	1.00	2.00	1.00	1.00	1.00	1.00	1.00
551	2.00	1.00	1.00	1.00	2.00	1.00	1.00	2.00
552	2.00	1.00	2.00	1.00	1.00	2.00	2.00	1.00
553	1.00	1.00	1.00	1.00	2.00	1.00	2.00	1.00
554	1.00	1.00	1.00	1.00	2.00	1.00	1.00	1.00
555	1.00	2.00	1.00	1.00	1.00	2.00	1.00	2.00
556	2.00	1.00	1.00	2.00	1.00	1.00	1.00	1.00
557	1.00	1.00	2.00	1.00	2.00	2.00	2.00	2.00
558	1.00	1.00	1.00	1.00	2.00	2.00	2.00	2.00
559	1.00	1.00	1.00	1.00	1.00	1.00	1.00	1.00
560	1.00	1.00	1.00	1.00	2.00	1.00	1.00	1.00
561	2.00	1.00	2.00	1.00	1.00	1.00	1.00	1.00
562	2.00	1.00	1.00	1.00	2.00	2.00	1.00	2.00
563	1.00	1.00	1.00	1.00	2.00	2.00	2.00	2.00
564	2.00	1.00	2.00	1.00	1.00	2.00	1.00	2.00
565	1.00	1.00	1.00	1.00	1.00	1.00	1.00	1.00
566	1.00	2.00	2.00	1.00	1.00	1.00	2.00	2.00
567	1.00	1.00	2.00	1.00	1.00	1.00	2.00	1.00
568	2.00	2.00	2.00	2.00	1.00	2.00	2.00	1.00
569	2.00	2.00	2.00	2.00	2.00	2.00	2.00	2.00
570	1.00	1.00	1.00	1.00	1.00	1.00	1.00	1.00
571	1.00	1.00	1.00	1.00	1.00	1.00	1.00	1.00
572	1.00	1.00	1.00	1.00	2.00	1.00	2.00	1.00
573	2.00	1.00	1.00	1.00	2.00	1.00	2.00	1.00
574	1.00	1.00	1.00	1.00	2.00	2.00	1.00	1.00
575	1.00	1.00	1.00	1.00	2.00	1.00	1.00	1.00
576	2.00	1.00	2.00	2.00	2.00	1.00	1.00	1.00
577	2.00	1.00	2.00	2.00	1.00	2.00	1.00	1.00
578	2.00	1.00	2.00	1.00	2.00	1.00	2.00	1.00
579	2.00	1.00	2.00	2.00	1.00	1.00	2.00	1.00
580	2.00	1.00	2.00	1.00	2.00	1.00	2.00	1.00
581	1.00	1.00	1.00	2.00	1.00	2.00	1.00	1.00
582	1.00	1.00	1.00	1.00	2.00	1.00	2.00	1.00
583	1.00	1.00	1.00	1.00	1.00	1.00	2.00	1.00
584	1.00	1.00	1.00	1.00	2.00	1.00	1.00	1.00
585	1.00	1.00	1.00	2.00	1.00	1.00	1.00	1.00
586	2.00	2.00	2.00	1.00	1.00	2.00	2.00	2.00
587	1.00	1.00	1.00	1.00	1.00	1.00	1.00	1.00
588	2.00	2.00	2.00	2.00	2.00	2.00	2.00	2.00

	M15	B1	B2	B3	B4	B5	B6	B7
547	1.00	2.00	1.00	2.00	1.00	2.00	1.00	1.00
548	2.00	2.00	1.00	2.00	1.00	2.00	2.00	2.00
549	2.00	1.00	1.00	1.00	1.00	2.00	2.00	2.00
550	1.00	1.00	2.00	2.00	1.00	2.00	1.00	1.00
551	1.00	1.00	1.00	1.00	1.00	1.00	2.00	2.00
552	2.00	1.00	1.00	1.00	1.00	1.00	2.00	2.00
553	2.00	1.00	1.00	2.00	1.00	1.00	2.00	2.00
554	1.00	1.00	2.00	2.00	1.00	2.00	2.00	1.00
555	1.00	1.00	2.00	1.00	2.00	1.00	2.00	1.00
556	1.00	1.00	1.00	1.00	1.00	1.00	2.00	2.00
557	2.00	2.00	2.00	2.00	1.00	1.00	1.00	1.00
558	1.00	2.00	2.00	2.00	1.00	2.00	2.00	2.00
559	1.00	1.00	1.00	1.00	1.00	1.00	1.00	1.00
560	1.00	1.00	1.00	1.00	1.00	1.00	1.00	1.00
561	1.00	1.00	2.00	2.00	1.00	2.00	1.00	1.00
562	2.00	1.00	2.00	2.00	1.00	1.00	2.00	1.00
563	1.00	1.00	2.00	1.00	1.00	2.00	2.00	2.00
564	2.00	2.00	1.00	1.00	2.00	2.00	2.00	2.00
565	1.00	1.00	1.00	2.00	2.00	2.00	2.00	2.00
566	1.00	1.00	1.00	1.00	1.00	1.00	2.00	1.00
567	2.00	1.00	1.00	2.00	1.00	1.00	1.00	1.00
568	2.00	2.00	1.00	1.00	2.00	2.00	2.00	2.00
569	2.00	2.00	1.00	1.00	1.00	1.00	2.00	2.00
570	1.00	1.00	1.00	1.00	1.00	1.00	1.00	1.00
571	1.00	2.00	2.00	2.00	1.00	2.00	1.00	1.00
572	1.00	1.00	2.00	2.00	1.00	1.00	1.00	1.00
573	1.00	1.00	1.00	2.00	2.00	1.00	1.00	2.00
574	1.00	2.00	2.00	2.00	2.00	2.00	2.00	2.00
575	1.00	1.00	2.00	2.00	1.00	2.00	2.00	2.00
576	1.00	1.00	2.00	2.00	1.00	1.00	2.00	2.00
577	1.00	2.00	1.00	2.00	1.00	1.00	2.00	2.00
578	1.00	1.00	2.00	1.00	2.00	2.00	2.00	1.00
579	1.00	1.00	1.00	2.00	1.00	2.00	2.00	2.00
580	2.00	1.00	1.00	2.00	1.00	2.00	2.00	2.00
581	2.00	2.00	1.00	2.00	1.00	1.00	1.00	1.00
582	1.00	1.00	2.00	1.00	1.00	2.00	1.00	1.00
583	2.00	1.00	1.00	2.00	1.00	2.00	2.00	1.00
584	1.00	1.00	2.00	2.00	2.00	1.00	1.00	1.00
585	1.00	1.00	2.00	2.00	1.00	1.00	1.00	1.00
586	1.00	2.00	2.00	1.00	2.00	1.00	1.00	1.00
587	1.00	1.00	1.00	1.00	1.00	1.00	1.00	1.00
588	2.00	1.00	1.00	2.00	1.00	1.00	2.00	2.00

	B8	B9	B10	B11	B12	B13	B14	W1
547	2.00	2.00	2.00	1.00	2.00	2.00	2.00	2.00
548	1.00	2.00	2.00	1.00	2.00	2.00	1.00	1.00
549	2.00	2.00	1.00	1.00	2.00	2.00	2.00	1.00
550	2.00	2.00	2.00	1.00	1.00	2.00	1.00	2.00
551	2.00	2.00	2.00	1.00	2.00	2.00	2.00	1.00
552	2.00	2.00	2.00	2.00	2.00	2.00	2.00	2.00
553	2.00	1.00	1.00	1.00	2.00	2.00	1.00	1.00
554	2.00	2.00	1.00	1.00	2.00	2.00	2.00	1.00
555	2.00	1.00	2.00	1.00	2.00	1.00	2.00	2.00
556	2.00	2.00	1.00	1.00	1.00	1.00	2.00	1.00
557	2.00	2.00	2.00	2.00	2.00	2.00	1.00	1.00
558	2.00	1.00	1.00	1.00	2.00	1.00	1.00	2.00
559	1.00	1.00	1.00	1.00	1.00	1.00	1.00	1.00
560	1.00	1.00	1.00	1.00	1.00	1.00	1.00	1.00
561	2.00	2.00	1.00	1.00	2.00	2.00	2.00	1.00
562	1.00	1.00	1.00	2.00	2.00	1.00	1.00	2.00
563	2.00	2.00	2.00	1.00	1.00	2.00	1.00	2.00
564	1.00	2.00	2.00	2.00	2.00	2.00	2.00	1.00
565	2.00	2.00	2.00	2.00	2.00	2.00	2.00	1.00
566	1.00	1.00	2.00	1.00	2.00	2.00	2.00	2.00
567	1.00	1.00	1.00	1.00	1.00	1.00	1.00	2.00
568	2.00	2.00	2.00	2.00	2.00	2.00	2.00	2.00
569	2.00	1.00	1.00	1.00	1.00	2.00	1.00	2.00
570	1.00	1.00	1.00	1.00	1.00	1.00	1.00	1.00
571	1.00	1.00	1.00	1.00	2.00	1.00	1.00	1.00
572	1.00	2.00	1.00	1.00	2.00	2.00	2.00	1.00
573	2.00	2.00	1.00	1.00	2.00	1.00	2.00	2.00
574	2.00	2.00	2.00	2.00	2.00	2.00	2.00	1.00
575	2.00	2.00	1.00	2.00	2.00	2.00	1.00	1.00
576	2.00	1.00	2.00	1.00	2.00	2.00	1.00	2.00
577	1.00	2.00	1.00	1.00	1.00	2.00	1.00	1.00
578	1.00	2.00	1.00	1.00	2.00	2.00	2.00	1.00
579	2.00	2.00	2.00	1.00	2.00	2.00	2.00	2.00
580	1.00	1.00	2.00	1.00	2.00	1.00	1.00	1.00
581	2.00	1.00	2.00	1.00	1.00	1.00	1.00	1.00
582	2.00	2.00	2.00	1.00	2.00	2.00	2.00	1.00
583	2.00	2.00	1.00	1.00	2.00	2.00	1.00	1.00
584	1.00	1.00	1.00	1.00	2.00	2.00	1.00	1.00
585	1.00	1.00	1.00	1.00	1.00	2.00	1.00	1.00
586	2.00	1.00	2.00	2.00	1.00	1.00	2.00	1.00
587	1.00	1.00	1.00	1.00	2.00	2.00	1.00	2.00
588	2.00	2.00	2.00	1.00	2.00	2.00	2.00	2.00

	W2	W3	W4	W5	W6	E1	V1	V2
547	2.00	1.00	1.00	2.00	2.00	2.00	.	.
548	1.00	1.00	2.00	1.00	2.00	2.00	.	.
549	2.00	1.00	2.00	1.00	2.00	2.00	.	.
550	2.00	1.00	1.00	1.00	2.00	2.00	.	.
551	2.00	1.00	1.00	1.00	2.00	2.00	.	.
552	2.00	1.00	2.00	1.00	2.00	2.00	.	.
553	1.00	1.00	1.00	1.00	2.00	2.00	.	.
554	1.00	1.00	2.00	1.00	2.00	2.00	.	.
555	1.00	2.00	1.00	2.00	1.00	2.00	.	.
556	1.00	1.00	1.00	1.00	2.00	2.00	.	.
557	1.00	1.00	1.00	1.00	1.00	2.00	.	.
558	2.00	2.00	2.00	1.00	1.00	2.00	.	.
559	1.00	1.00	1.00	1.00	1.00	2.00	.	.
560	1.00	1.00	1.00	1.00	1.00	2.00	.	.
561	1.00	1.00	2.00	1.00	2.00	2.00	.	.
562	2.00	1.00	1.00	1.00	1.00	2.00	.	.
563	1.00	1.00	1.00	1.00	2.00	2.00	.	.
564	2.00	1.00	2.00	1.00	2.00	2.00	.	.
565	1.00	1.00	1.00	1.00	1.00	2.00	.	.
566	2.00	1.00	2.00	1.00	1.00	2.00	.	.
567	2.00	2.00	2.00	1.00	1.00	2.00	.	.
568	2.00	2.00	2.00	2.00	2.00	2.00	.	.
569	2.00	2.00	2.00	2.00	2.00	2.00	.	.
570	1.00	1.00	1.00	1.00	1.00	2.00	.	.
571	1.00	2.00	2.00	1.00	2.00	2.00	.	.
572	1.00	2.00	1.00	1.00	2.00	2.00	.	.
573	1.00	2.00	1.00	1.00	1.00	2.00	.	.
574	1.00	1.00	1.00	1.00	1.00	2.00	.	.
575	1.00	1.00	1.00	1.00	2.00	2.00	.	.
576	2.00	1.00	1.00	2.00	1.00	2.00	.	.
577	1.00	2.00	2.00	1.00	2.00	2.00	.	.
578	1.00	1.00	2.00	1.00	1.00	2.00	.	.
579	2.00	1.00	2.00	1.00	2.00	2.00	.	.
580	2.00	1.00	1.00	2.00	2.00	2.00	.	.
581	2.00	1.00	2.00	1.00	1.00	2.00	.	.
582	1.00	2.00	1.00	1.00	2.00	2.00	.	.
583	1.00	1.00	2.00	2.00	2.00	2.00	.	.
584	1.00	1.00	1.00	1.00	1.00	2.00	.	.
585	1.00	1.00	1.00	1.00	1.00	2.00	.	.
586	2.00	2.00	2.00	1.00	1.00	2.00	.	.
587	2.00	1.00	2.00	2.00	1.00	2.00	.	.
588	2.00	1.00	2.00	1.00	1.00	2.00	.	.

	V3	V4	V5	V6	E2	E3	E4	C1
547	.	.	.	.	.	.	.	.
548	.	.	.	.	.	.	.	.
549	.	.	.	.	.	.	.	.
550	.	.	.	.	.	.	.	.
551	.	.	.	.	.	.	.	.
552	.	.	.	.	.	.	.	.
553	.	.	.	.	.	.	.	.
554	.	.	.	.	.	.	.	.
555	.	.	.	.	.	.	.	.
556	.	.	.	.	.	.	.	.
557	.	.	.	.	.	.	.	.
558	.	.	.	.	.	.	.	.
559	.	.	.	.	.	.	.	.
560	.	.	.	.	.	.	.	.
561	.	.	.	.	.	.	.	.
562	.	.	.	.	.	.	.	.
563	.	.	.	.	.	.	.	.
564	.	.	.	.	.	.	.	.
565	.	.	.	.	.	.	.	.
566	.	.	.	.	.	.	.	.
567	.	.	.	.	.	.	.	.
568	.	.	.	.	.	.	.	.
569	.	.	.	.	.	.	.	.
570	.	.	.	.	.	.	.	.
571	.	.	.	.	.	.	.	.
572	.	.	.	.	.	.	.	.
573	.	.	.	.	.	.	.	.
574	.	.	.	.	.	.	.	.
575	.	.	.	.	.	.	.	.
576	.	.	.	.	.	.	.	.
577	.	.	.	.	.	.	.	.
578	.	.	.	.	.	.	.	.
579	.	.	.	.	.	.	.	.
580	.	.	.	.	.	.	.	.
581	.	.	.	.	.	.	.	.
582	.	.	.	.	.	.	.	.
583	.	.	.	.	.	.	.	.
584	.	.	.	.	.	.	.	.
585	.	.	.	.	.	.	.	.
586	.	.	.	.	.	.	.	.
587	.	.	.	.	.	.	.	.
588	.	.	.	.	.	.	.	.

	C2	C3	C4	C5	C6	C7	filter_$
547	.	.	.	.	.	.	0
548	.	.	.	.	.	.	0
549	.	.	.	.	.	.	0
550	.	.	.	.	.	.	0
551	.	.	.	.	.	.	0
552	.	.	.	.	.	.	0
553	.	.	.	.	.	.	0
554	.	.	.	.	.	.	0
555	.	.	.	.	.	.	0
556	.	.	.	.	.	.	0
557	.	.	.	.	.	.	0
558	.	.	.	.	.	.	0
559	.	.	.	.	.	.	0
560	.	.	.	.	.	.	0
561	.	.	.	.	.	.	0
562	.	.	.	.	.	.	0
563	.	.	.	.	.	.	0
564	.	.	.	.	.	.	0
565	.	.	.	.	.	.	0
566	.	.	.	.	.	.	0
567	.	.	.	.	.	.	0
568	.	.	.	.	.	.	0
569	.	.	.	.	.	.	0
570	.	.	.	.	.	.	0
571	.	.	.	.	.	.	0
572	.	.	.	.	.	.	0
573	.	.	.	.	.	.	0
574	.	.	.	.	.	.	0
575	.	.	.	.	.	.	0
576	.	.	.	.	.	.	0
577	.	.	.	.	.	.	0
578	.	.	.	.	.	.	0
579	.	.	.	.	.	.	0
580	.	.	.	.	.	.	0
581	.	.	.	.	.	.	0
582	.	.	.	.	.	.	0
583	.	.	.	.	.	.	0
584	.	.	.	.	.	.	0
585	.	.	.	.	.	.	0
586	.	.	.	.	.	.	0
587	.	.	.	.	.	.	0
588	.	.	.	.	.	.	0

	SN	Group	Gender	Age	Work	Work2	Work3	Sector
589	385.00	1.00	2.00	20.00	2.00	1.00	1.00	1.00
590	389.00	1.00	1.00	24.00	6.00	1.00	1.00	1.00
591	392.00	1.00	2.00	19.00	2.00	1.00	1.00	1.00
592	394.00	1.00	2.00	18.00	2.00	1.00	1.00	1.00
593	396.00	1.00	2.00	18.00	2.00	1.00	1.00	1.00
594	397.00	1.00	2.00	19.00	2.00	1.00	1.00	1.00
595	400.00	1.00	2.00	19.00	2.00	1.00	1.00	1.00
596	401.00	1.00	2.00	18.00	2.00	1.00	1.00	1.00
597	402.00	1.00	2.00	18.00	2.00	1.00	1.00	1.00
598	403.00	1.00	2.00	19.00	2.00	1.00	1.00	1.00
599	411.00	1.00	1.00	21.00	4.00	1.00	1.00	1.00
600	426.00	1.00	1.00	26.00	5.00	1.00	1.00	1.00
601	427.00	1.00	1.00	21.00	3.00	1.00	1.00	1.00
602	428.00	1.00	2.00	22.00	5.00	1.00	1.00	1.00
603	438.00	1.00	2.00	22.00	4.00	1.00	1.00	1.00
604	463.00	1.00	1.00	19.00	2.00	1.00	1.00	1.00
605	464.00	1.00	1.00	22.00	3.00	1.00	1.00	1.00
606	474.00	1.00	1.00	21.00	3.00	1.00	1.00	1.00
607	475.00	1.00	1.00	20.00	2.00	1.00	1.00	1.00
608	478.00	1.00	1.00	28.00	8.00	2.00	2.00	2.00
609	485.00	1.00	2.00	24.00	6.00	1.00	1.00	2.00
610	489.00	1.00	2.00	23.00	6.00	1.00	1.00	1.00
611	518.00	2.00	2.00	23.00	6.00	1.00	1.00	1.00
612	519.00	2.00	2.00	20.00	2.00	1.00	1.00	1.00
613	522.00	2.00	2.00	19.00	2.00	1.00	1.00	1.00
614	525.00	2.00	2.00	19.00	2.00	1.00	1.00	1.00
615	526.00	2.00	2.00	21.00	3.00	1.00	1.00	1.00
616	530.00	2.00	2.00	20.00	3.00	1.00	1.00	1.00
617	531.00	2.00	2.00	21.00	3.00	1.00	1.00	1.00
618	533.00	2.00	2.00	19.00	2.00	1.00	1.00	1.00
619	534.00	2.00	2.00	20.00	2.00	1.00	1.00	1.00
620	535.00	2.00	1.00	19.00	2.00	1.00	1.00	1.00
621	539.00	2.00	2.00	26.00	8.00	2.00	2.00	2.00
622	540.00	2.00	2.00	25.00	5.00	1.00	1.00	1.00
623	545.00	2.00	2.00	19.00	2.00	1.00	1.00	1.00
624	546.00	2.00	2.00	20.00	2.00	1.00	1.00	1.00
625	547.00	2.00	2.00	21.00	2.00	1.00	1.00	1.00
626	549.00	2.00	2.00	22.00	4.00	1.00	1.00	1.00
627	550.00	2.00	2.00	21.00	2.00	1.00	1.00	1.00
628	555.00	2.00	2.00	21.00	3.00	1.00	1.00	1.00
629	560.00	2.00	2.00	21.00	4.00	1.00	1.00	1.00
630	567.00	2.00	1.00	21.00	2.00	1.00	1.00	1.00

	Region	Nationality	M1	M2	M3	M4	M5	M6
589	2.00	1.00	1.00	1.00	1.00	1.00	1.00	1.00
590	5.00	1.00	1.00	1.00	1.00	1.00	1.00	1.00
591	3.00	1.00	1.00	1.00	1.00	1.00	1.00	1.00
592	1.00	1.00	1.00	1.00	1.00	1.00	1.00	1.00
593	1.00	1.00	1.00	1.00	1.00	1.00	1.00	1.00
594	1.00	1.00	1.00	1.00	2.00	1.00	1.00	2.00
595	1.00	1.00	1.00	1.00	1.00	1.00	1.00	1.00
596	1.00	1.00	1.00	1.00	1.00	1.00	1.00	1.00
597	1.00	1.00	1.00	1.00	1.00	1.00	1.00	1.00
598	1.00	1.00	1.00	1.00	2.00	1.00	2.00	2.00
599	4.00	1.00	2.00	2.00	2.00	1.00	1.00	2.00
600	2.00	1.00	1.00	2.00	2.00	1.00	1.00	2.00
601	2.00	1.00	2.00	2.00	1.00	1.00	2.00	1.00
602	2.00	1.00	1.00	1.00	2.00	1.00	1.00	2.00
603	1.00	1.00	2.00	1.00	2.00	1.00	1.00	1.00
604	4.00	1.00	1.00	1.00	1.00	1.00	2.00	1.00
605	1.00	1.00	1.00	1.00	1.00	1.00	1.00	1.00
606	4.00	1.00	2.00	2.00	1.00	1.00	1.00	2.00
607	4.00	1.00	1.00	1.00	2.00	1.00	1.00	1.00
608	3.00	1.00	1.00	1.00	1.00	2.00	1.00	1.00
609	2.00	1.00	1.00	1.00	1.00	1.00	1.00	1.00
610	1.00	1.00	1.00	2.00	1.00	1.00	2.00	1.00
611	1.00	1.00	1.00	2.00	2.00	1.00	1.00	1.00
612	1.00	1.00	1.00	1.00	1.00	1.00	1.00	1.00
613	1.00	1.00	2.00	1.00	2.00	2.00	1.00	1.00
614	2.00	1.00	1.00	1.00	1.00	1.00	1.00	2.00
615	3.00	1.00	1.00	1.00	1.00	1.00	2.00	1.00
616	1.00	1.00	1.00	1.00	2.00	1.00	1.00	1.00
617	1.00	1.00	1.00	1.00	1.00	2.00	2.00	2.00
618	1.00	1.00	1.00	1.00	1.00	1.00	1.00	1.00
619	1.00	1.00	1.00	1.00	2.00	1.00	1.00	2.00
620	1.00	1.00	1.00	1.00	1.00	1.00	1.00	1.00
621	5.00	1.00	2.00	2.00	2.00	2.00	2.00	2.00
622	2.00	1.00	1.00	2.00	1.00	2.00	2.00	1.00
623	1.00	1.00	1.00	1.00	2.00	1.00	1.00	1.00
624	1.00	1.00	1.00	1.00	2.00	1.00	1.00	1.00
625	5.00	1.00	1.00	2.00	1.00	1.00	1.00	1.00
626	1.00	1.00	1.00	1.00	1.00	2.00	1.00	1.00
627	5.00	1.00	1.00	2.00	1.00	2.00	1.00	1.00
628	5.00	1.00	1.00	2.00	1.00	1.00	1.00	1.00
629	1.00	1.00	1.00	1.00	1.00	1.00	2.00	2.00
630	2.00	1.00	1.00	1.00	2.00	1.00	2.00	2.00

	M7	M8	M9	M10	M11	M12	M13	M14
589	1.00	1.00	2.00	1.00	1.00	1.00	1.00	1.00
590	2.00	1.00	1.00	1.00	2.00	1.00	2.00	1.00
591	1.00	1.00	1.00	1.00	1.00	1.00	1.00	1.00
592	1.00	1.00	1.00	1.00	1.00	1.00	1.00	1.00
593	1.00	1.00	1.00	1.00	1.00	2.00	2.00	1.00
594	2.00	1.00	1.00	1.00	1.00	1.00	1.00	1.00
595	2.00	1.00	1.00	1.00	2.00	2.00	1.00	1.00
596	1.00	1.00	1.00	1.00	1.00	1.00	1.00	1.00
597	1.00	1.00	2.00	1.00	1.00	1.00	1.00	1.00
598	2.00	1.00	2.00	1.00	2.00	1.00	1.00	1.00
599	2.00	2.00	2.00	2.00	1.00	1.00	1.00	1.00
600	1.00	1.00	1.00	1.00	2.00	1.00	1.00	2.00
601	1.00	1.00	2.00	1.00	2.00	2.00	2.00	1.00
602	1.00	1.00	2.00	1.00	1.00	1.00	2.00	1.00
603	1.00	1.00	2.00	2.00	1.00	2.00	1.00	1.00
604	1.00	1.00	1.00	1.00	2.00	1.00	1.00	1.00
605	1.00	1.00	1.00	1.00	1.00	1.00	1.00	1.00
606	2.00	1.00	1.00	1.00	1.00	1.00	1.00	2.00
607	1.00	1.00	1.00	1.00	2.00	1.00	1.00	1.00
608	2.00	1.00	2.00	1.00	2.00	1.00	2.00	1.00
609	1.00	1.00	1.00	1.00	2.00	1.00	1.00	1.00
610	1.00	1.00	1.00	1.00	1.00	2.00	1.00	1.00
611	2.00	2.00	2.00	1.00	2.00	1.00	1.00	1.00
612	1.00	1.00	1.00	1.00	1.00	1.00	1.00	1.00
613	2.00	1.00	1.00	1.00	1.00	1.00	1.00	1.00
614	2.00	1.00	1.00	1.00	2.00	2.00	2.00	2.00
615	1.00	1.00	1.00	1.00	2.00	1.00	1.00	1.00
616	1.00	1.00	1.00	1.00	1.00	2.00	2.00	1.00
617	1.00	1.00	1.00	2.00	1.00	2.00	1.00	1.00
618	1.00	1.00	1.00	1.00	1.00	1.00	1.00	1.00
619	1.00	1.00	1.00	1.00	1.00	1.00	1.00	1.00
620	1.00	1.00	2.00	1.00	2.00	1.00	1.00	1.00
621	2.00	2.00	2.00	1.00	2.00	2.00	1.00	1.00
622	1.00	1.00	1.00	1.00	2.00	2.00	2.00	1.00
623	1.00	1.00	1.00	1.00	1.00	1.00	1.00	1.00
624	1.00	1.00	1.00	1.00	1.00	1.00	1.00	1.00
625	1.00	1.00	1.00	1.00	2.00	1.00	1.00	1.00
626	2.00	1.00	2.00	1.00	1.00	2.00	1.00	1.00
627	1.00	1.00	1.00	1.00	1.00	2.00	1.00	1.00
628	2.00	2.00	1.00	1.00	1.00	2.00	1.00	1.00
629	1.00	1.00	2.00	1.00	1.00	1.00	1.00	1.00
630	2.00	2.00	2.00	2.00	2.00	1.00	2.00	2.00

	M15	B1	B2	B3	B4	B5	B6	B7
589	1.00	2.00	2.00	2.00	1.00	2.00	2.00	2.00
590	1.00	1.00	1.00	2.00	2.00	2.00	1.00	1.00
591	1.00	1.00	1.00	1.00	1.00	1.00	1.00	1.00
592	1.00	2.00	2.00	2.00	2.00	2.00	2.00	2.00
593	2.00	2.00	1.00	1.00	1.00	1.00	2.00	2.00
594	1.00	2.00	2.00	2.00	1.00	2.00	2.00	1.00
595	1.00	1.00	2.00	2.00	1.00	1.00	2.00	2.00
596	1.00	1.00	2.00	2.00	1.00	2.00	2.00	2.00
597	2.00	2.00	2.00	2.00	1.00	2.00	2.00	2.00
598	1.00	1.00	1.00	2.00	1.00	2.00	2.00	2.00
599	2.00	1.00	1.00	1.00	2.00	1.00	2.00	2.00
600	2.00	2.00	2.00	1.00	1.00	1.00	2.00	2.00
601	2.00	1.00	2.00	1.00	1.00	1.00	2.00	2.00
602	1.00	1.00	2.00	2.00	1.00	1.00	2.00	2.00
603	1.00	1.00	1.00	1.00	1.00	1.00	2.00	2.00
604	1.00	2.00	2.00	2.00	1.00	2.00	2.00	2.00
605	1.00	1.00	1.00	1.00	1.00	1.00	1.00	1.00
606	2.00	2.00	1.00	1.00	1.00	1.00	1.00	1.00
607	1.00	1.00	2.00	2.00	1.00	2.00	2.00	2.00
608	2.00	1.00	2.00	1.00	2.00	1.00	2.00	1.00
609	1.00	1.00	1.00	2.00	1.00	2.00	1.00	1.00
610	1.00	1.00	1.00	1.00	1.00	2.00	2.00	2.00
611	2.00	1.00	2.00	1.00	1.00	2.00	2.00	2.00
612	1.00	1.00	2.00	2.00	1.00	2.00	2.00	2.00
613	1.00	2.00	2.00	2.00	1.00	2.00	2.00	2.00
614	1.00	1.00	2.00	1.00	2.00	2.00	2.00	2.00
615	1.00	1.00	1.00	2.00	1.00	2.00	2.00	2.00
616	1.00	2.00	1.00	2.00	1.00	2.00	1.00	1.00
617	1.00	1.00	1.00	1.00	1.00	1.00	1.00	1.00
618	1.00	2.00	2.00	2.00	1.00	2.00	1.00	1.00
619	1.00	1.00	2.00	2.00	1.00	1.00	2.00	1.00
620	1.00	2.00	2.00	1.00	2.00	2.00	2.00	2.00
621	2.00	1.00	1.00	1.00	1.00	1.00	1.00	1.00
622	1.00	1.00	1.00	1.00	1.00	1.00	1.00	1.00
623	1.00	1.00	2.00	2.00	1.00	2.00	1.00	1.00
624	1.00	2.00	1.00	2.00	2.00	2.00	1.00	1.00
625	2.00	1.00	1.00	1.00	1.00	2.00	2.00	2.00
626	1.00	1.00	2.00	1.00	1.00	2.00	1.00	1.00
627	1.00	1.00	2.00	2.00	1.00	2.00	2.00	2.00
628	1.00	1.00	1.00	2.00	1.00	1.00	1.00	1.00
629	1.00	2.00	1.00	1.00	1.00	1.00	1.00	1.00
630	2.00	2.00	1.00	1.00	1.00	2.00	2.00	2.00

	B8	B9	B10	B11	B12	B13	B14	W1
589	2.00	1.00	1.00	1.00	2.00	2.00	1.00	1.00
590	1.00	2.00	2.00	1.00	2.00	2.00	2.00	2.00
591	1.00	1.00	1.00	1.00	1.00	1.00	1.00	1.00
592	2.00	2.00	2.00	2.00	2.00	2.00	2.00	1.00
593	1.00	2.00	1.00	1.00	2.00	2.00	2.00	1.00
594	1.00	1.00	1.00	1.00	1.00	1.00	1.00	1.00
595	2.00	1.00	2.00	2.00	2.00	2.00	1.00	2.00
596	2.00	2.00	1.00	1.00	2.00	2.00	1.00	1.00
597	2.00	2.00	2.00	1.00	2.00	2.00	2.00	1.00
598	2.00	1.00	1.00	1.00	2.00	1.00	2.00	1.00
599	2.00	2.00	2.00	2.00	2.00	2.00	2.00	2.00
600	2.00	2.00	1.00	1.00	2.00	2.00	2.00	1.00
601	2.00	2.00	2.00	2.00	2.00	2.00	2.00	1.00
602	2.00	2.00	1.00	1.00	2.00	1.00	1.00	1.00
603	2.00	2.00	1.00	1.00	1.00	2.00	2.00	1.00
604	2.00	2.00	2.00	1.00	2.00	2.00	2.00	1.00
605	1.00	1.00	1.00	1.00	1.00	1.00	1.00	1.00
606	2.00	2.00	1.00	1.00	1.00	2.00	2.00	1.00
607	2.00	1.00	1.00	1.00	2.00	2.00	2.00	1.00
608	2.00	1.00	1.00	2.00	1.00	1.00	2.00	1.00
609	2.00	2.00	2.00	1.00	2.00	2.00	1.00	2.00
610	2.00	2.00	1.00	1.00	1.00	2.00	2.00	1.00
611	2.00	2.00	1.00	2.00	2.00	2.00	2.00	2.00
612	2.00	1.00	1.00	1.00	2.00	2.00	2.00	1.00
613	2.00	1.00	1.00	1.00	2.00	1.00	1.00	2.00
614	2.00	2.00	2.00	1.00	2.00	2.00	2.00	1.00
615	2.00	2.00	1.00	1.00	2.00	2.00	2.00	2.00
616	1.00	1.00	1.00	1.00	2.00	2.00	1.00	1.00
617	1.00	1.00	1.00	2.00	2.00	2.00	2.00	1.00
618	2.00	2.00	2.00	2.00	2.00	1.00	2.00	2.00
619	1.00	1.00	1.00	1.00	2.00	2.00	2.00	1.00
620	2.00	1.00	1.00	1.00	2.00	2.00	1.00	1.00
621	1.00	1.00	1.00	1.00	1.00	1.00	1.00	1.00
622	2.00	1.00	1.00	2.00	1.00	1.00	1.00	1.00
623	2.00	1.00	1.00	2.00	2.00	1.00	1.00	1.00
624	1.00	1.00	1.00	1.00	2.00	1.00	2.00	1.00
625	1.00	1.00	1.00	1.00	2.00	1.00	1.00	2.00
626	1.00	1.00	1.00	1.00	1.00	2.00	2.00	2.00
627	2.00	2.00	2.00	2.00	2.00	2.00	1.00	2.00
628	2.00	1.00	1.00	1.00	2.00	1.00	1.00	1.00
629	1.00	1.00	1.00	1.00	1.00	1.00	1.00	1.00
630	1.00	1.00	2.00	2.00	1.00	2.00	1.00	1.00

	W2	W3	W4	W5	W6	E1	V1	V2
589	1.00	2.00	1.00	1.00	2.00	2.00	.	.
590	2.00	1.00	1.00	1.00	2.00	2.00	.	.
591	1.00	1.00	1.00	1.00	1.00	2.00	.	.
592	1.00	1.00	1.00	1.00	1.00	2.00	.	.
593	2.00	1.00	2.00	2.00	2.00	2.00	.	.
594	1.00	1.00	1.00	1.00	2.00	2.00	.	.
595	2.00	2.00	2.00	1.00	1.00	2.00	.	.
596	1.00	1.00	2.00	1.00	1.00	2.00	.	.
597	1.00	1.00	1.00	1.00	2.00	2.00	.	.
598	2.00	1.00	2.00	1.00	2.00	2.00	.	.
599	2.00	1.00	2.00	2.00	2.00	2.00	.	.
600	1.00	1.00	1.00	1.00	1.00	2.00	.	.
601	1.00	2.00	2.00	2.00	2.00	2.00	.	.
602	1.00	2.00	2.00	1.00	1.00	2.00	.	.
603	2.00	1.00	1.00	1.00	2.00	2.00	.	.
604	1.00	1.00	1.00	1.00	2.00	2.00	.	.
605	1.00	1.00	1.00	1.00	1.00	2.00	.	.
606	1.00	1.00	1.00	1.00	1.00	2.00	.	.
607	1.00	1.00	1.00	1.00	1.00	2.00	.	.
608	1.00	2.00	1.00	1.00	1.00	2.00	.	.
609	2.00	1.00	2.00	1.00	2.00	2.00	.	.
610	1.00	2.00	1.00	2.00	2.00	2.00	.	.
611	2.00	1.00	2.00	1.00	2.00	2.00	.	.
612	1.00	1.00	1.00	1.00	2.00	2.00	.	.
613	2.00	1.00	1.00	1.00	1.00	2.00	.	.
614	1.00	2.00	2.00	2.00	2.00	2.00	.	.
615	2.00	1.00	1.00	1.00	2.00	2.00	.	.
616	1.00	1.00	1.00	1.00	1.00	2.00	.	.
617	2.00	1.00	2.00	1.00	2.00	2.00	.	.
618	2.00	2.00	1.00	1.00	1.00	2.00	.	.
619	1.00	1.00	1.00	1.00	1.00	2.00	.	.
620	1.00	1.00	1.00	1.00	1.00	2.00	.	.
621	1.00	1.00	2.00	2.00	2.00	2.00	.	.
622	2.00	1.00	1.00	1.00	1.00	2.00	.	.
623	1.00	2.00	1.00	1.00	2.00	2.00	.	.
624	1.00	2.00	1.00	1.00	2.00	2.00	.	.
625	2.00	1.00	1.00	1.00	2.00	2.00	.	.
626	1.00	1.00	1.00	1.00	2.00	2.00	.	.
627	2.00	2.00	2.00	2.00	2.00	2.00	.	.
628	1.00	1.00	2.00	1.00	1.00	2.00	.	.
629	2.00	1.00	1.00	1.00	2.00	2.00	.	.
630	1.00	1.00	1.00	1.00	1.00	2.00	.	.

	V3	V4	V5	V6	E2	E3	E4	C1
589	.	.	.	.	.	.	.	.
590	.	.	.	.	.	.	.	.
591	.	.	.	.	.	.	.	.
592	.	.	.	.	.	.	.	.
593	.	.	.	.	.	.	.	.
594	.	.	.	.	.	.	.	.
595	.	.	.	.	.	.	.	.
596	.	.	.	.	.	.	.	.
597	.	.	.	.	.	.	.	.
598	.	.	.	.	.	.	.	.
599	.	.	.	.	.	.	.	.
600	.	.	.	.	.	.	.	.
601	.	.	.	.	.	.	.	.
602	.	.	.	.	.	.	.	.
603	.	.	.	.	.	.	.	.
604	.	.	.	.	.	.	.	.
605	.	.	.	.	.	.	.	.
606	.	.	.	.	.	.	.	.
607	.	.	.	.	.	.	.	.
608	.	.	.	.	.	.	.	.
609	.	.	.	.	.	.	.	.
610	.	.	.	.	.	.	.	.
611	.	.	.	.	.	.	.	.
612	.	.	.	.	.	.	.	.
613	.	.	.	.	.	.	.	.
614	.	.	.	.	.	.	.	.
615	.	.	.	.	.	.	.	.
616	.	.	.	.	.	.	.	.
617	.	.	.	.	.	.	.	.
618	.	.	.	.	.	.	.	.
619	.	.	.	.	.	.	.	.
620	.	.	.	.	.	.	.	.
621	.	.	.	.	.	.	.	.
622	.	.	.	.	.	.	.	.
623	.	.	.	.	.	.	.	.
624	.	.	.	.	.	.	.	.
625	.	.	.	.	.	.	.	.
626	.	.	.	.	.	.	.	.
627	.	.	.	.	.	.	.	.
628	.	.	.	.	.	.	.	.
629	.	.	.	.	.	.	.	.
630	.	.	.	.	.	.	.	.

	C2	C3	C4	C5	C6	C7	filter_$
589	.	.	.	.	.	.	0
590	.	.	.	.	.	.	0
591	.	.	.	.	.	.	0
592	.	.	.	.	.	.	0
593	.	.	.	.	.	.	0
594	.	.	.	.	.	.	0
595	.	.	.	.	.	.	0
596	.	.	.	.	.	.	0
597	.	.	.	.	.	.	0
598	.	.	.	.	.	.	0
599	.	.	.	.	.	.	0
600	.	.	.	.	.	.	0
601	.	.	.	.	.	.	0
602	.	.	.	.	.	.	0
603	.	.	.	.	.	.	0
604	.	.	.	.	.	.	0
605	.	.	.	.	.	.	0
606	.	.	.	.	.	.	0
607	.	.	.	.	.	.	0
608	.	.	.	.	.	.	0
609	.	.	.	.	.	.	0
610	.	.	.	.	.	.	0
611	.	.	.	.	.	.	0
612	.	.	.	.	.	.	0
613	.	.	.	.	.	.	0
614	.	.	.	.	.	.	0
615	.	.	.	.	.	.	0
616	.	.	.	.	.	.	0
617	.	.	.	.	.	.	0
618	.	.	.	.	.	.	0
619	.	.	.	.	.	.	0
620	.	.	.	.	.	.	0
621	.	.	.	.	.	.	0
622	.	.	.	.	.	.	0
623	.	.	.	.	.	.	0
624	.	.	.	.	.	.	0
625	.	.	.	.	.	.	0
626	.	.	.	.	.	.	0
627	.	.	.	.	.	.	0
628	.	.	.	.	.	.	0
629	.	.	.	.	.	.	0
630	.	.	.	.	.	.	0

	SN	Group	Gender	Age	Work	Work2	Work3	Sector
631	569.00	2.00	2.00	21.00	4.00	1.00	1.00	1.00
632	571.00	2.00	2.00	20.00	3.00	1.00	1.00	1.00
633	574.00	2.00	2.00	20.00	3.00	1.00	1.00	1.00
634	578.00	2.00	2.00	20.00	3.00	1.00	1.00	1.00
635	585.00	2.00	2.00	21.00	3.00	1.00	1.00	1.00
636	586.00	2.00	2.00	20.00	3.00	1.00	1.00	1.00
637	588.00	2.00	2.00	22.00	4.00	1.00	1.00	1.00
638	595.00	2.00	2.00	21.00	4.00	1.00	1.00	1.00
639	602.00	2.00	2.00	23.00	6.00	1.00	1.00	1.00
640	605.00	2.00	2.00	23.00	6.00	1.00	1.00	1.00
641	610.00	2.00	2.00	20.00	3.00	1.00	1.00	1.00
642	613.00	2.00	2.00	20.00	3.00	1.00	1.00	1.00
643	618.00	2.00	2.00	23.00	5.00	1.00	1.00	1.00
644	619.00	2.00	2.00	21.00	3.00	1.00	1.00	1.00
645	621.00	2.00	2.00	23.00	5.00	1.00	1.00	1.00
646	624.00	2.00	2.00	20.00	3.00	1.00	1.00	1.00
647	625.00	2.00	2.00	20.00	3.00	1.00	1.00	1.00
648	629.00	2.00	2.00	35.00	2.00	1.00	1.00	2.00
649	638.00	2.00	2.00	20.00	3.00	1.00	1.00	1.00
650	639.00	2.00	2.00	22.00	3.00	1.00	1.00	1.00
651	640.00	2.00	2.00	33.00	9.00	3.00	2.00	2.00
652	641.00	2.00	2.00	20.00	3.00	1.00	1.00	1.00
653	642.00	2.00	2.00	20.00	3.00	1.00	1.00	1.00
654	643.00	2.00	2.00	19.00	2.00	1.00	1.00	1.00
655	645.00	2.00	2.00	21.00	5.00	1.00	1.00	2.00

	Region	Nationality	M1	M2	M3	M4	M5	M6
631	1.00	1.00	2.00	1.00	1.00	2.00	2.00	1.00
632	1.00	1.00	1.00	1.00	1.00	1.00	1.00	2.00
633	3.00	1.00	1.00	1.00	1.00	1.00	1.00	1.00
634	3.00	1.00	1.00	1.00	1.00	1.00	1.00	1.00
635	3.00	1.00	1.00	1.00	1.00	1.00	1.00	2.00
636	3.00	1.00	1.00	1.00	1.00	1.00	1.00	1.00
637	4.00	1.00	1.00	1.00	1.00	1.00	1.00	1.00
638	4.00	1.00	1.00	1.00	2.00	1.00	1.00	1.00
639	1.00	1.00	1.00	1.00	1.00	1.00	1.00	1.00
640	1.00	1.00	1.00	1.00	2.00	1.00	1.00	2.00
641	1.00	1.00	1.00	1.00	2.00	1.00	1.00	1.00
642	1.00	1.00	2.00	1.00	1.00	1.00	1.00	1.00
643	3.00	1.00	1.00	2.00	2.00	2.00	2.00	1.00
644	4.00	1.00	1.00	2.00	2.00	1.00	1.00	2.00
645	3.00	1.00	1.00	1.00	2.00	1.00	1.00	1.00
646	1.00	1.00	2.00	1.00	1.00	1.00	1.00	2.00
647	1.00	1.00	1.00	1.00	1.00	1.00	2.00	2.00
648	4.00	1.00	1.00	1.00	1.00	1.00	1.00	1.00
649	5.00	1.00	1.00	1.00	1.00	1.00	1.00	1.00
650	3.00	1.00	1.00	2.00	2.00	2.00	1.00	1.00
651	2.00	2.00	1.00	1.00	2.00	1.00	1.00	2.00
652	5.00	1.00	1.00	1.00	1.00	1.00	1.00	1.00
653	5.00	1.00	1.00	1.00	2.00	2.00	1.00	1.00
654	5.00	1.00	1.00	1.00	1.00	1.00	1.00	1.00
655	2.00	2.00	1.00	1.00	2.00	1.00	1.00	2.00

	M7	M8	M9	M10	M11	M12	M13	M14
631	2.00	2.00	2.00	2.00	1.00	2.00	2.00	1.00
632	1.00	1.00	2.00	2.00	2.00	1.00	1.00	1.00
633	1.00	1.00	2.00	1.00	2.00	2.00	2.00	1.00
634	2.00	1.00	2.00	1.00	2.00	1.00	2.00	1.00
635	1.00	1.00	1.00	1.00	1.00	1.00	1.00	1.00
636	1.00	1.00	1.00	1.00	1.00	1.00	1.00	1.00
637	1.00	1.00	1.00	1.00	2.00	1.00	1.00	1.00
638	1.00	1.00	1.00	1.00	1.00	1.00	1.00	1.00
639	1.00	1.00	1.00	1.00	2.00	1.00	2.00	1.00
640	1.00	1.00	1.00	1.00	1.00	1.00	2.00	1.00
641	1.00	1.00	1.00	1.00	2.00	1.00	1.00	1.00
642	2.00	1.00	2.00	2.00	2.00	1.00	1.00	1.00
643	1.00	1.00	2.00	1.00	2.00	2.00	2.00	1.00
644	1.00	2.00	1.00	2.00	1.00	2.00	1.00	2.00
645	2.00	1.00	2.00	1.00	1.00	1.00	1.00	2.00
646	2.00	1.00	2.00	1.00	1.00	1.00	1.00	2.00
647	1.00	2.00	1.00	2.00	2.00	1.00	2.00	1.00
648	1.00	1.00	1.00	1.00	2.00	1.00	1.00	1.00
649	1.00	1.00	1.00	1.00	2.00	1.00	1.00	1.00
650	1.00	1.00	1.00	2.00	2.00	2.00	1.00	2.00
651	2.00	2.00	2.00	2.00	2.00	2.00	2.00	2.00
652	1.00	1.00	1.00	1.00	1.00	1.00	1.00	1.00
653	1.00	1.00	2.00	1.00	1.00	2.00	1.00	1.00
654	1.00	1.00	1.00	1.00	1.00	1.00	1.00	1.00
655	1.00	1.00	2.00	1.00	1.00	1.00	1.00	1.00

	M15	B1	B2	B3	B4	B5	B6	B7
631	2.00	2.00	2.00	2.00	1.00	1.00	2.00	2.00
632	1.00	1.00	1.00	2.00	2.00	2.00	2.00	2.00
633	1.00	2.00	2.00	2.00	1.00	2.00	1.00	1.00
634	1.00	1.00	2.00	2.00	2.00	2.00	1.00	2.00
635	1.00	1.00	2.00	1.00	1.00	1.00	1.00	1.00
636	1.00	1.00	1.00	1.00	1.00	1.00	1.00	1.00
637	1.00	2.00	2.00	2.00	1.00	2.00	1.00	1.00
638	1.00	1.00	2.00	2.00	1.00	1.00	1.00	1.00
639	1.00	2.00	1.00	2.00	2.00	2.00	1.00	1.00
640	2.00	2.00	2.00	2.00	1.00	2.00	1.00	1.00
641	1.00	1.00	2.00	2.00	1.00	2.00	2.00	2.00
642	1.00	1.00	2.00	1.00	1.00	1.00	1.00	1.00
643	2.00	1.00	2.00	1.00	1.00	1.00	2.00	2.00
644	1.00	1.00	1.00	2.00	1.00	2.00	1.00	1.00
645	2.00	1.00	2.00	2.00	1.00	1.00	1.00	1.00
646	1.00	1.00	2.00	2.00	1.00	1.00	1.00	1.00
647	1.00	2.00	2.00	1.00	1.00	1.00	1.00	1.00
648	1.00	2.00	1.00	1.00	2.00	2.00	1.00	1.00
649	1.00	2.00	2.00	2.00	1.00	2.00	2.00	2.00
650	1.00	1.00	1.00	2.00	2.00	1.00	1.00	1.00
651	1.00	1.00	1.00	1.00	1.00	1.00	2.00	2.00
652	1.00	1.00	2.00	2.00	1.00	1.00	1.00	1.00
653	1.00	1.00	1.00	1.00	1.00	1.00	1.00	1.00
654	1.00	1.00	2.00	2.00	1.00	1.00	2.00	2.00
655	1.00	1.00	2.00	2.00	1.00	2.00	2.00	2.00

	B8	B9	B10	B11	B12	B13	B14	W1
631	1.00	2.00	1.00	1.00	2.00	2.00	2.00	2.00
632	2.00	2.00	1.00	1.00	2.00	2.00	2.00	1.00
633	2.00	1.00	2.00	2.00	2.00	2.00	2.00	2.00
634	2.00	2.00	2.00	1.00	2.00	1.00	1.00	2.00
635	1.00	1.00	1.00	1.00	1.00	1.00	1.00	1.00
636	1.00	1.00	1.00	1.00	1.00	1.00	1.00	1.00
637	2.00	2.00	1.00	1.00	2.00	2.00	2.00	1.00
638	2.00	1.00	1.00	1.00	2.00	2.00	1.00	2.00
639	1.00	1.00	1.00	1.00	2.00	1.00	2.00	1.00
640	2.00	1.00	1.00	1.00	2.00	2.00	1.00	1.00
641	2.00	2.00	1.00	1.00	2.00	2.00	2.00	1.00
642	1.00	2.00	2.00	1.00	2.00	1.00	1.00	2.00
643	2.00	1.00	1.00	2.00	2.00	2.00	1.00	2.00
644	1.00	2.00	1.00	1.00	2.00	1.00	2.00	1.00
645	1.00	1.00	1.00	2.00	1.00	1.00	2.00	1.00
646	2.00	1.00	1.00	1.00	2.00	2.00	1.00	1.00
647	2.00	1.00	1.00	2.00	1.00	1.00	2.00	1.00
648	1.00	1.00	1.00	1.00	1.00	1.00	2.00	1.00
649	2.00	2.00	2.00	2.00	2.00	2.00	2.00	1.00
650	2.00	2.00	2.00	1.00	1.00	1.00	1.00	1.00
651	2.00	2.00	2.00	2.00	2.00	2.00	2.00	1.00
652	1.00	1.00	1.00	1.00	2.00	1.00	1.00	1.00
653	1.00	1.00	1.00	1.00	1.00	1.00	1.00	1.00
654	2.00	2.00	1.00	1.00	1.00	1.00	1.00	2.00
655	2.00	2.00	2.00	2.00	2.00	2.00	2.00	1.00

	W2	W3	W4	W5	W6	E1	V1	V2
631	2.00	1.00	1.00	2.00	2.00	2.00	.	.
632	1.00	1.00	1.00	1.00	1.00	2.00	.	.
633	2.00	1.00	1.00	1.00	1.00	2.00	.	.
634	1.00	1.00	2.00	1.00	2.00	2.00	.	.
635	1.00	1.00	1.00	2.00	1.00	2.00	.	.
636	1.00	1.00	1.00	1.00	1.00	2.00	.	.
637	1.00	1.00	1.00	1.00	1.00	2.00	.	.
638	2.00	1.00	1.00	1.00	2.00	2.00	.	.
639	2.00	1.00	1.00	2.00	1.00	2.00	.	.
640	1.00	1.00	1.00	1.00	2.00	2.00	.	.
641	1.00	1.00	1.00	1.00	1.00	2.00	.	.
642	1.00	1.00	2.00	1.00	2.00	2.00	.	.
643	1.00	2.00	1.00	1.00	1.00	2.00	.	.
644	2.00	2.00	1.00	2.00	2.00	2.00	.	.
645	1.00	1.00	1.00	1.00	1.00	2.00	.	.
646	2.00	1.00	2.00	1.00	1.00	2.00	.	.
647	1.00	2.00	1.00	1.00	1.00	2.00	.	.
648	1.00	1.00	1.00	1.00	1.00	2.00	.	.
649	1.00	1.00	1.00	1.00	2.00	2.00	.	.
650	2.00	2.00	2.00	2.00	2.00	2.00	.	.
651	1.00	2.00	2.00	2.00	2.00	2.00	.	.
652	1.00	1.00	1.00	1.00	1.00	2.00	.	.
653	2.00	1.00	2.00	1.00	2.00	2.00	.	.
654	2.00	1.00	1.00	1.00	1.00	2.00	.	.
655	1.00	1.00	1.00	1.00	2.00	2.00	.	.

	V3	V4	V5	V6	E2	E3	E4	C1
631	.	.	.	.	.	.	.	.
632	.	.	.	.	.	.	.	.
633	.	.	.	.	.	.	.	.
634	.	.	.	.	.	.	.	.
635	.	.	.	.	.	.	.	.
636	.	.	.	.	.	.	.	.
637	.	.	.	.	.	.	.	.
638	.	.	.	.	.	.	.	.
639	.	.	.	.	.	.	.	.
640	.	.	.	.	.	.	.	.
641	.	.	.	.	.	.	.	.
642	.	.	.	.	.	.	.	.
643	.	.	.	.	.	.	.	.
644	.	.	.	.	.	.	.	.
645	.	.	.	.	.	.	.	.
646	.	.	.	.	.	.	.	.
647	.	.	.	.	.	.	.	.
648	.	.	.	.	.	.	.	.
649	.	.	.	.	.	.	.	.
650	.	.	.	.	.	.	.	.
651	.	.	.	.	.	.	.	.
652	.	.	.	.	.	.	.	.
653	.	.	.	.	.	.	.	.
654	.	.	.	.	.	.	.	.
655	.	.	.	.	.	.	.	.

	C2	C3	C4	C5	C6	C7	filter_$
631	.	.	.	.	.	.	0
632	.	.	.	.	.	.	0
633	.	.	.	.	.	.	0
634	.	.	.	.	.	.	0
635	.	.	.	.	.	.	0
636	.	.	.	.	.	.	0
637	.	.	.	.	.	.	0
638	.	.	.	.	.	.	0
639	.	.	.	.	.	.	0
640	.	.	.	.	.	.	0
641	.	.	.	.	.	.	0
642	.	.	.	.	.	.	0
643	.	.	.	.	.	.	0
644	.	.	.	.	.	.	0
645	.	.	.	.	.	.	0
646	.	.	.	.	.	.	0
647	.	.	.	.	.	.	0
648	.	.	.	.	.	.	0
649	.	.	.	.	.	.	0
650	.	.	.	.	.	.	0
651	.	.	.	.	.	.	0
652	.	.	.	.	.	.	0
653	.	.	.	.	.	.	0
654	.	.	.	.	.	.	0
655	.	.	.	.	.	.	0
